# Fully Biocatalytic Rearrangement of Furans to Spirolactones

**DOI:** 10.1021/acscatal.3c00132

**Published:** 2023-05-15

**Authors:** Yu-Chang Liu, J. D. Rolfes, Joel Björklund, Jan Deska

**Affiliations:** † Department of Chemistry, University of Helsinki, A.I. Virtasen aukio 1, 00560 Helsinki, Finland; ‡ Department of Chemistry, Aalto University, Kemistintie 1, 02150 Espoo, Finland; § Albert Hofmann Institute for Physiochemical Sustainability, Albert-Schweitzer-Street 22, 32602 Vlotho, Germany

**Keywords:** biocatalytic, rearrangement, multienzymatic, cascade catalysis, furans, spirolactone, cyclization

## Abstract

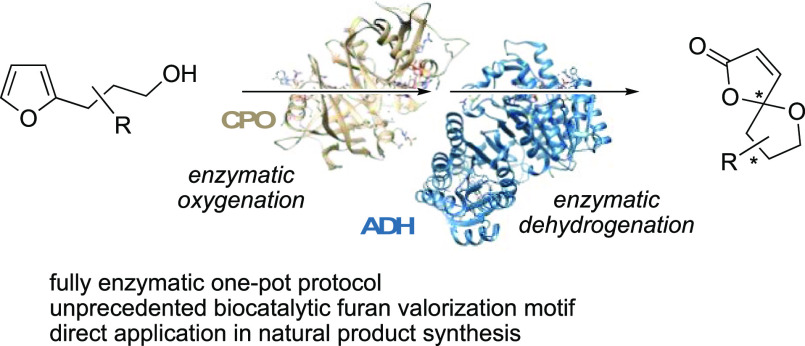

A multienzymatic pathway enables the preparation of optically pure spirolactone building blocks. In a streamlined one-pot reaction cascade, the combination of chloroperoxidase, an oxidase, and an alcohol dehydrogenase renders an efficient reaction cascade for the conversion of hydroxy-functionalized furans to the spirocyclic products. The fully biocatalytic method is successfully employed in the total synthesis of the bioactive natural product (+)-crassalactone D, and as the key module in a chemoenzymatic route yielding lanceolactone A.

## Introduction

**Figure 1:**
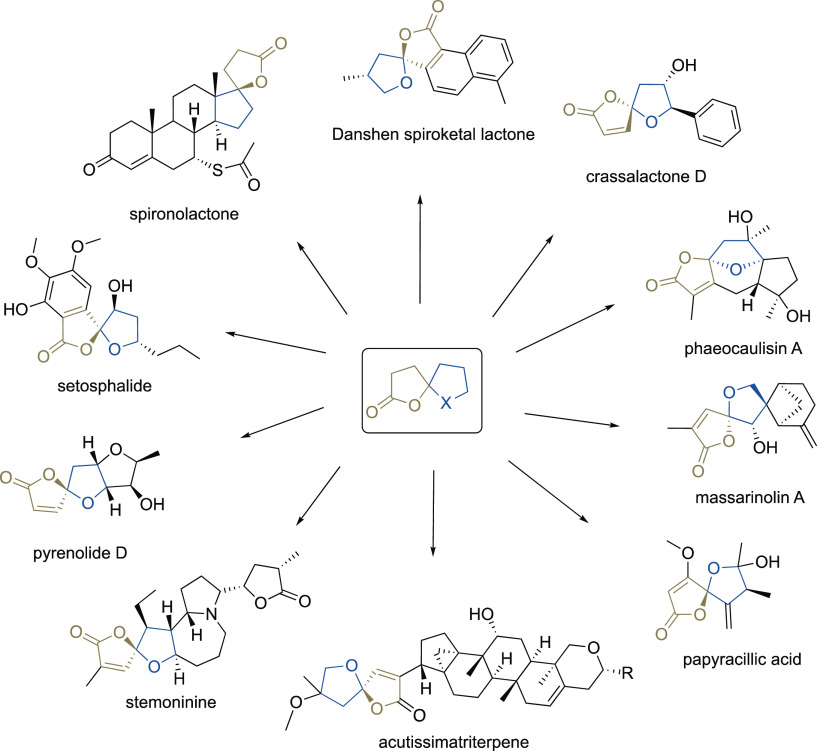
Biologically active spirolactone metabolites.

Oxa-spirolactones, with their unique framework comprising a [4.4] spirocyclic ketal motif and a γ-butenolide unit, are important building blocks that are found in a broad range of biologically active natural products and pharmaceuticals (Figure 1). Spironolactone (aldactone) is a well-known synthetic steroid used in the treatment of patients with high blood pressure, heart failure, hypokalemia, liver scarring, and kidney problems.^1^ The seco-abietane diterpenoid Danshen spiroketal lactone is the main active ingredient in Chinese medical herb *Salvia prionitis*, which is used to treat cardiovascular diseases, especially angina pectoris and myocardial infarction.^2^ Acutissimatriterpene and its analogues were found to have cytotoxic and anti-HIV-1 activities.^3^ Crassalactone D, sequoiamonascins, and phaeocaulisin A are typical representatives of secondary metabolites with antitumor activities.^4^ Massarinolin A, pyrenolide D, papyracillic acid, and setosphalide show potent antibacterial or antifungal activities, respectively,^5^ whereas stemoninine, isolated from the roots of *Stemona tuberosa*, exhibits dose-dependent inhibition of citric acid-induced coughing.^6^ With such a plethora of bioactivities, methodologies that address the preparation of this unique structural motif have received substantial attention over the years in order to facilitate an effective synthesis of these natural products and structural analogues thereof.^7^

**Scheme 1:**
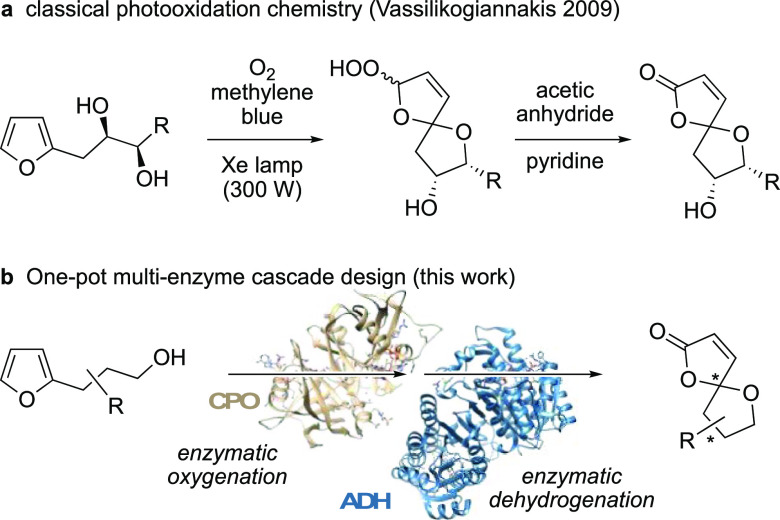
Furan Spirocyclizations: (a) Photochemical Peroxidation and Elimination by Methylene Blue and Acetic Anhydride; (b) Fully Biological Strategy Utilizing Oxygenase and Dehydrogenase Enzyme Modules

Among the various starting materials for the synthesis of spirolactones, furans represent a particularly attractive resource. As a substantial side stream of lignocellulosic biorefinery, furanoic building blocks such as furfural are nowadays considered as sustainable platform chemicals,^8^ and broader application of these heterocycles should be highly encouraged in light of green chemistry developments. Furans allow for a rich follow-up chemistry, and in 2009, Vassilikogiannakis and co-workers illustrated a photochemical reaction sequence for the preparation of γ-oxaspiro-γ-lactone. Employing singlet oxygen for the furan oxidation via [4 + 2] cycloaddition followed by an acetic anhydride-induced dehydration of the intermediate spirocyclic hydroperoxide furnished functionalized oxa-spirolactones in an elegant fashion (Scheme 1a).^9^ While deviating from the original Diels-Alder cycloaddition pathway, oxygenative furan rearrangements like the Achmatowicz oxidation proceed via similar unsaturated carbonyl intermediates.^10^ Hence, we envisaged that based on our previous work on biocatalytic Achmatowicz-type furan oxidations,^11^ we would be able to design an interrupted Achmatowicz pathway that would lead to oxo-spirolactols instead, which could be further derivatized/modified by auxiliary enzyme modules. Based on this consideration, we herein present a fully biological route to access γ-oxaspiro-γ-lactones from 2-(γ-hydroxyalkyl)furans via spirocyclic acetals in a one-pot fashion, combining peroxidase-induced furan oxygenations with ketoreductase-catalyzed dehydrogenations (Scheme 1b). The in-depth method development is supplemented by synthetic applications in more complex cascade designs for the total synthesis of the natural spirocyclic lactones lanceolactone A and crassalactone D.

## Results and Discussion

We commenced our study with the adaptation of a previously developed biocatalytic module for the oxygenative ring cleavage of furans. Chloroperoxidase (CPO) from *Caldariomyces fumago* was employed as a biocatalyst thanks to its oxygenase activities on many kinds of aromatic compounds,^12^ and it served us well in the development of an enzyme-driven Achmatowicz oxidation protocol. Gratifyingly, CPO in combination with glucose oxidase (GOx, for the in situ generation of hydrogen peroxide from glucose and air) was able to smoothly transform model substrate **1a** into desired spirocyclic ketal **3a**. In fact, in comparison with the peroxidase-catalyzed ring expansion of furyl alcohols, the oxa-spirocyclic rearrangement proceeded with much higher rates, and greater than 99% conversion was reached within only 20 min.

**Scheme 2:**
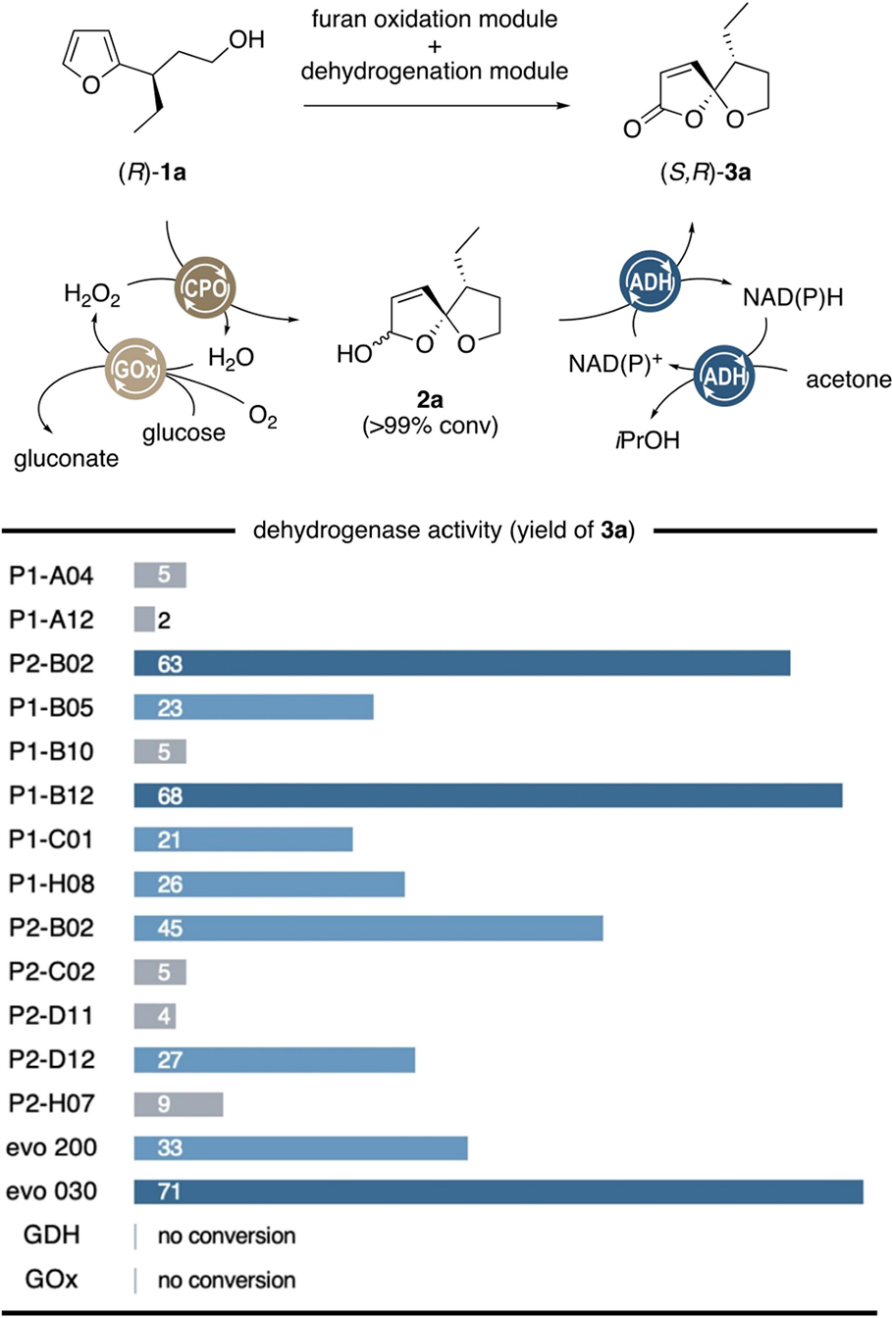
Evaluation of Biocatalysts for the Dehydrogenation of **2a**, Following CPO/GOx-Mediated Oxygenation in One Pot Reaction conditions: (i) **1a** (6.5 mM), chloroperoxidase (10 U), glucose oxidase (1 U), and glucose (10 mM) in citrate buffer (1 mL, 100 mM, pH 6.0) at 30 °C for 30 min; (ii) alcohol dehydrogenase evo_030_ (2 mg), NAD^+^ (2 mM), and acetone (50 mM) at 30 °C for 20 h.

With the initial key biotransformation operating without any deviation from the original protocol, we were encouraged to focus on the biocatalyst screening for a subsequent dehydrogenation of intermediate **2a** to the targeted spirolactone **3a** en route to achieve a true multienzymatic cascade. In our previous work, ketoreductases (KREDs, a.k.a. alcohol dehydrogenases) were designed to serve as isomerases bearing isomerizations of Achmatowicz-type pyranones to give enantiopure δ-lactones in good yield, taking advantage of an irreversible dehydrogenation of the cyclic ketal as a driving force.^13^ Thus, KREDs were also considered as the biocatalysts of choice as they would likely engage in a similar, irreversible lactol dehydrogenation of **2a**. In order to simplify the overall process, only KREDs with substrate-coupled NAD(P)-recycling (i.e., using acetone as a sacrificial electron acceptor) were chosen for the evaluation of the dehydrogenation performance. Moreover, in order to identify the optimal KREDs, compatible with the CPO/GOx couple, the screening was directly conducted in the same reaction mixture (in the presence of CPO, GOx, glucose and in citrate buffer) following the spirocyclic rearrangement of **1a** in the preceding reaction step. From a wider selection of commercial KREDs (from Codexic Inc. and evoCatal GmbH), a total of 15 different dehydrogenases showed significant conversion of spirolactol **2a**, giving rise to the desired γ-oxaspiro-γ-lactone **3a** in a diastereomeric ratio of 5:1, with only marginal variation in diastereoselectivity between the experiments (Scheme 2). Here, yields varied strongly, from lower single digits to greater 60% in case of Codexis’ NADP-dependent P_1_-B_02_ and P_1_-B_12_ (Scheme 2). NAD-dependent KRED evo_030_ was identified to pair optimally with the CPO/GOx system, leading to an overall yield of spirolactone **3a** of 71% over the two reaction steps. Thus, KRED evo_030_ was chosen to couple with CPO/GOx in our further investigation. Variation of pH and temperature showed little to moderate influence. A slightly acidic medium (pH 5–6) appeared to be optimal for formation of **2a**, while changes in temperature (25–40 °C) only resulted in marginal effects on diastereomeric ratios (Figure S1). To rule out any participation of sugar-active oxidoreductases, taking into account the sugar-like structure of hemiacetal **2a**, also glucose dehydrogenase and glucose oxidase were tested, yet neither production of **3a** nor consumption of **2a** was detected.

**Scheme 3:**
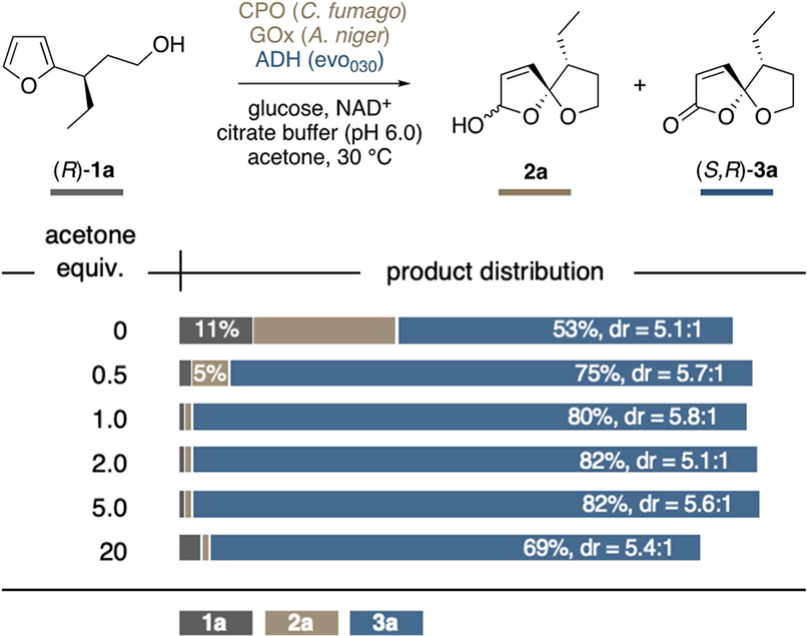
Influence of Acetone on the Concurrent System Product distributions and diastereomeric ratios are reported as average of duplicates.

After the stepwise cascade had been successfully established, we turned our attention to the actual goal, that is, the challenge to bring a direct, nonsequential conversion of furan **1** to the spirocyclic lactone (**3**) to fruition. Especially dehydrogenase activity on the primary alcohol of **1a** was considered a competitive pathway. However, in contrast to the hemiacetal dehydrogenation, the corresponding aldehyde would potentially also serve as the KRED substrate and could thus be reversibly reduced back to the starting material **1a**. We therefore set out to investigate the influence of the redox environment, in particular the effect of acetone as the terminal electron acceptor. Employing the enzyme triplet consisting of CPO, GOx, and evo_030_ in a nonsequential one-pot biotransformation of **1a**, the optimal yield of **3a** was achieved with 5 equiv of acetone, reaching an excellent 82%. More surprisingly, still with sub-stoichiometric amounts of acetone, in the absence of any other obvious electron acceptor, significant concentrations of the oxa-spirolactone **3a** could be observed. Most strikingly, even with no added acetone, 53% of the spirocyclic product was obtained, which exceeds the theoretical maximum yield relative to the added NAD^+^ (Scheme 3).

**Scheme 4:**
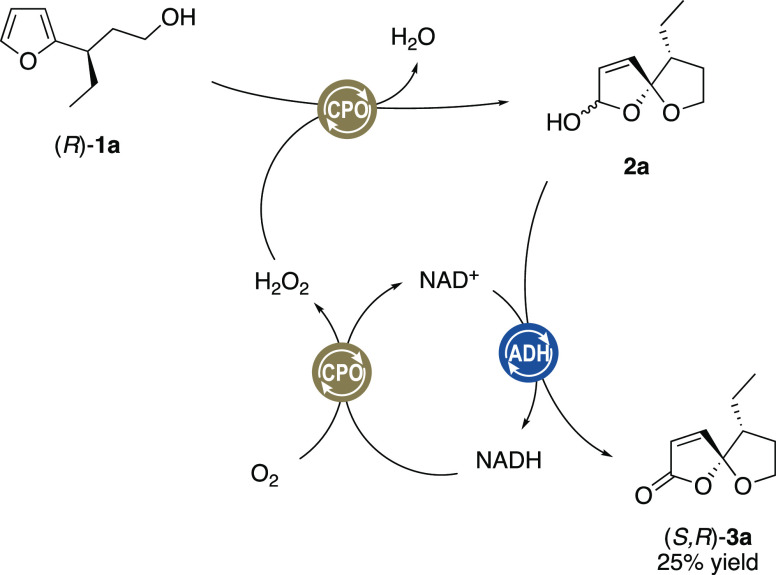
Redox Self-Sufficient System for the Spirolactonization of **1a** by CPO and evo_030_

We therefore suspected that CPO may act in some form or fashion as the regenerator to provide evo030 with more NAD^+^ by means of transferring electrons from reduced nicotinamide (NADH) to molecular oxygen meanwhile producing hydrogen peroxide. Indeed, NAD^+^ was detected when NADH was incubated with only CPO under air (Figure S2).^14^ Likewise, addition of catalase strongly inhibited the NADH-dependent conversion of **1a** to **3a** (Scheme 1). Consequently, based on this interesting discovery, an alternative enzyme cascade reaction was assembled as a nicotinamide self-sufficient system. In the absence of any oxidase and with 30 mol-% NADH, CPO and evo_030_ catalyzed the conversion of furyl alcohol **1a** to lactone **3a** (Scheme 4). The relatively low yield of 25% of **3a** probably has to be attributed to a rather low intrinsic NADH oxidase activity while also featuring a certain catalase side activity.^15^ Overall these parallel functions lead to a net loss of oxidation equivalents within the cascade. Further investigation on CPO’s use of nicotinamides as electron donors in biotransformation will be presented in our follow-up work.

**Scheme 5:**
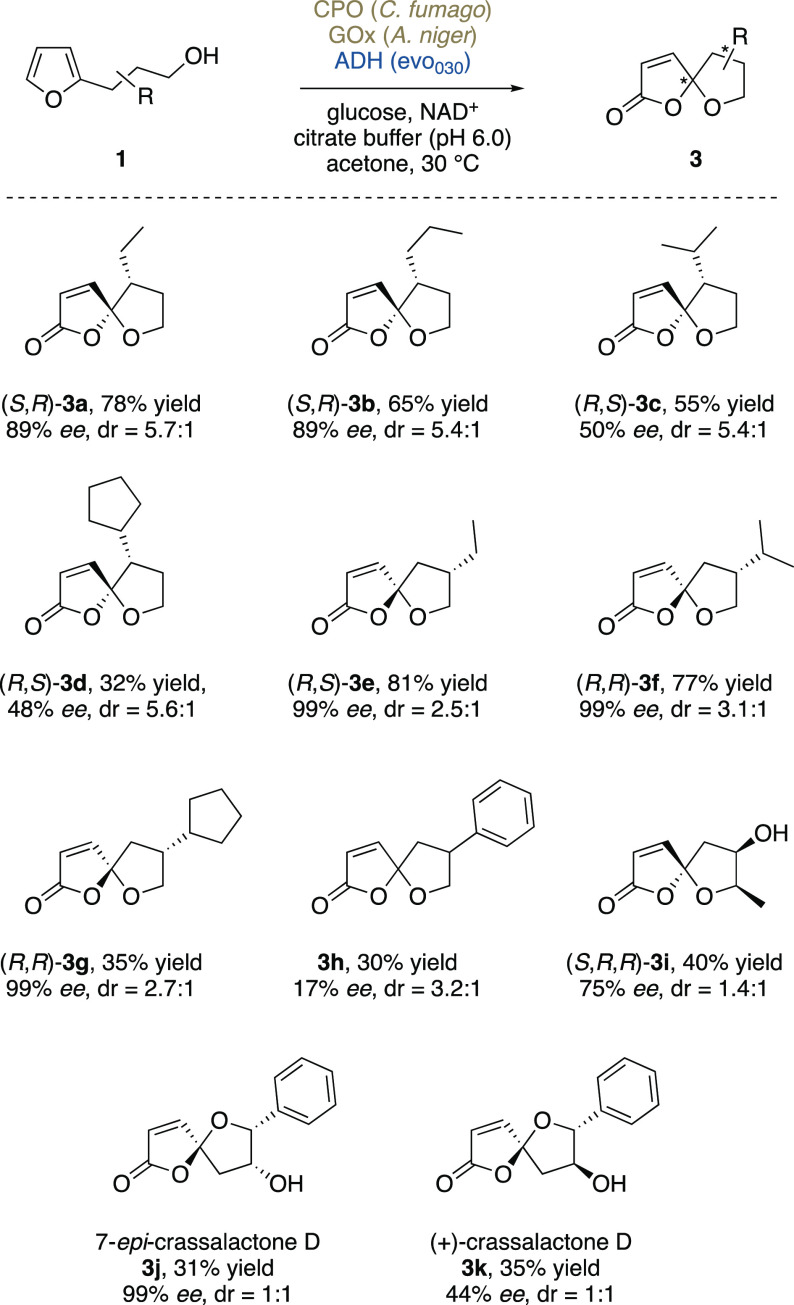
Substrate Scope of the Triple-Enzymatic System for the Conversion of 3-Furylpropanols to Spirolactones The depicted structures show the major diastereomers, whereas the minor isomers differ in their configurations at the spirocyclic center. Therein, reported diastereomeric ratios were determined by ^1^H NMR from the crude reaction mixtures; upon purification, sometimes further enrichment could be achieved. For assignment of configurations, see Supporting Information, Section 5).

With a promising, effective, new biocatalytic tool in hand, we next had a closer look on the wider scope of 2-(γ-hydroxyalkyl)furans, answering the questions of substrate tolerance of our methodology and the influence on different substitution patterns on the diastereoselectivity in the spirocyclization event. Optically enriched furan substrates were obtained through established asymmetric methods, mainly relying on enantioselective Michael additions (**1b**–**1d**) or lipase-mediated kinetic resolutions (**1e**–**1h**). To our delight, the multienzymatic one-pot protocol translated very well to a series of 2- and 3-substituted 3-furylpropanols (Scheme 5). 3-Substituted substrates delivered good to excellent conversions, with diastereoselectivities of **3a**–**3d** ranging above 5:1. Even more so, the spirocyclic products **3e**–**3h** from 2-substituted furylpropanols were obtained with generally very high conversions, though the isolated yield of the most lipophilic species was more modest. With the controlling stereogenic element further away from the newly forming spiro-center, also selectivities remained somewhat lower. Gratifyingly, despite the presence of the alcohol dehydrogenase evo_030_, a capable alcohol oxidizer, even secondary alcohol functions were well tolerated and the hydroxylated oxa-spirolactone **3i** could be obtained in decent yield. Even though the diastereoselectivity in the cyclization remained low, in contrast to most other spirocyclic products, the stereoisomers of **3i** (and **3h**) were easily separated by flash chromatography.

Thanks to the high tolerance on adjacent hydroxy functions, our triple-enzymatic spirolactonization method could also be directly applied in the total synthesis of γ-oxaspiro γ-lactone natural products. (+)-Crassalactone D (**3k**), a naturally occurring secondary metabolite from the leaves and twigs of *Polyalthia crassa* that exhibits antitumor properties, was originally synthesized by Tuchinda et al. in 2006.^4c^ The key intermediate **1k**, alongside its diastereomer **1j**, could be directly obtained through Sharpless dihydroxylation of the corresponding *trans*- and *cis*-styrylfurans, respectively. When subjected to the cyclization cascade mediated by CPO, GOx, and evo_030_, a separable mixture of 7-*epi*-crassalactone D (**3j**) and its 5,7-epimer (yield 31%, ratio of 1:1) was obtained from **1j**. With the same biotransformation process starting from **1k**, (+)-crassalactone D (**3k**) was directly obtained in an overall yield of 35% (as separable 1:1 mixture with 5-*epi*-crassalactone D). In all cases, the enantioselectivities of the starting materials were quantitatively transferred to the spirocyclic products. The alcohols (**1**) were synthesized by either copper-catalyzed asymmetric Michael addition, lipase-catalyzed kinetic resolution, or sharpless dihydroxylation, and the differences in optical purities reflect the efficacy of the methods that were used to prepare the enantioenriched furans.

The varying degree of diastereoselectivity of the enzymatic spirolactonizations raises the question to what extent the dehydrogenation biocatalyst would play a role in the selection process. In their very recent addition to methylene blue-induced furan oxidations, Kalaitzakis and Vassilikogiannakis also reported on the synthesis of spirolactones like **3a**, yet their method delivered close to equimolar mixtures of both diastereomers (dr = 1.4:1).^16^ At first glance, considering the similarities of the photochemical and biocatalytic oxygenation methods, it seemed plausible that a resolution-type selection of one diastereomer of hemiacetal **2** by the ADH evo_030_ could be taking place, potentially with concurrent epimerization of the spiro-center to enable full conversions and high yields. However, this hypothesis was quickly refuted as enzymatic furan oxidation followed by nonstereoselective oxidation with CrO_3_/H_2_SO_4_ provided **3a** with a diastereomeric ratio of 4.5:1, i.e., only marginally lower selectivity than in the bio-oxidation.

**Figure 2:**
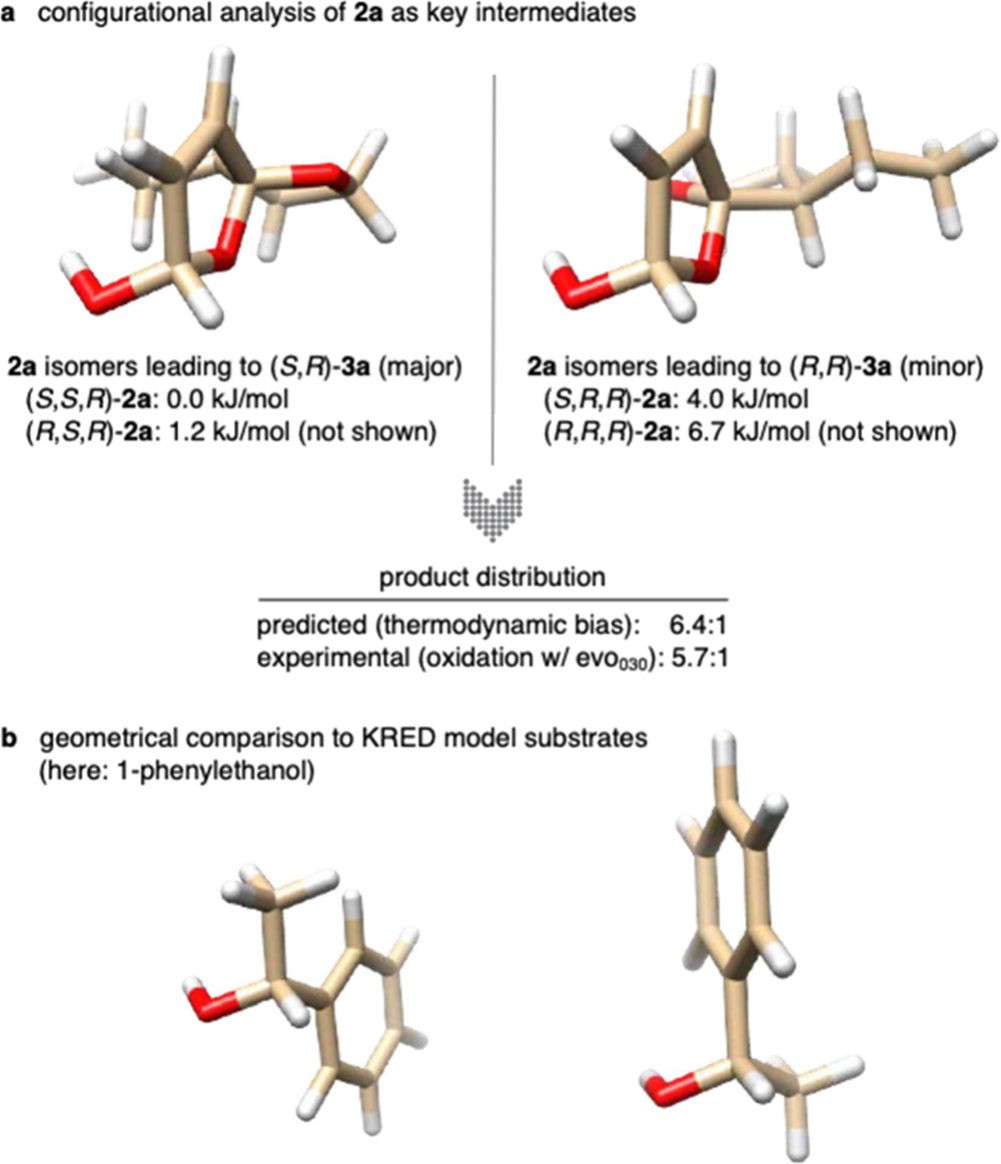
Configurational and geometrical analysis: (a) thermodynamic analysis of spirocyclic hemiacetal diastereomers and (b) comparison with (*S*)- and (*R*)-phenylethanol as the model substrate for the optimization of typical commercial KRED biocatalysts.

Quantum chemical analysis of all four possible diastereomers of **2a** and **2e**, respectively, by means of state-of-the-art ab initio calculations (SMD(H_2_O) DLPNO-CCSD(T)/def2-TZVPP//PBE0-D3/def2-TZVP), revealed a significant thermodynamic bias in favor of isomers carrying an (*S*)-configuration at the spiro-center (Figure S4). Gibbs free energies of spiro-(*S*) isomers of **2a** (0.0 and +1.2 kJ/mol, relative to the most stable isomer), which would lead to the formation of (*S*,*R*)-**3a**, were substantially lower than those of the spiro-(*R*) analogues (+4.0 and +6.7 kJ/mol, relative to the most stable isomer). Simple Boltzmann statistics would thus roughly suggest a 6.4:1 distribution of the hemiacetal isomers, if reaction rates would permit equilibration of the key intermediates prior to the irreversible dehydrogenation (Figure 2a). These results indicate that the oxidation by the alcohol dehydrogenase proceeds without significant stereodiscrimination and that the experimentally observed diastereoselectivity is mostly governed by the thermodynamics of the hemiacetals.

Several conclusions can be drawn from these stereochemical considerations. First, even though heavily inspired by Vassilikogiannakis’ photooxygenation methodology, it appears that the fully biocatalytic furan rearrangement differs significantly not just from the nature of the catalysts but also in regard to the underlying pathway and intermediates. While the commercial biocatalysts themselves cannot provide strong features of stereocontrol, the biocatalytic method still offers a considerably more diastereoselective route to oxa-spirolactones (photochemical 1.4:1 vs enzymatic 5.0:1) thanks to the alternative pathway that allows the thermodynamic bias to take full effect.^17^ That being said, the same is unfortunately also true for systems where little to no preference is dictating the stereochemical outcome, such as the crassalactone D examples. Yet, with the geometries of the hemiacetal isomers in hand, we can start examining possible reasons for the lack of stereocontrol. Comparison of the two **2a** diastereomers in Figure 2a shows clear differences in shape and sterics in different locations relative to the reactive hemiacetal center (see more projections in Figure S4). Those differences, however, are most significant toward the parts more distant from the reactive functional group, roughly 3.2–3.5 Å from the hemiacetal carbon. This stands in strong contrast to more typical KRED substrates such as 1-phenylethanol (Figure 2b). Many of these commercial dehydrogenases have been optimized to reduce ketones with high enantioselectivity, and they provide best results for substrates with major steric bias, i.e., ketones where the prochiral ketone carbon is decorated by one large and one small substituent (e.g., the acetophenone/phenylethanol couple). As such, the commercial KREDs’ active sites provide very distinct attractive and repulsive features very close to the substrate binding zinc ion and the bound nicotinamide cofactor. Dehydrogenases that would be capable of effectively discriminating between the spiro-(*S*) and spiro-(*R*) isomers of spiro-hemiacetals on the other hand required structural features further away from the catalytic center. While none of the commercial enzymes in this study provided this kind of recognition qualities, most likely due to the above-mentioned optimization criteria, the development of selective solutions through rational protein engineering appears to be a logical next step.

**Scheme 6:**
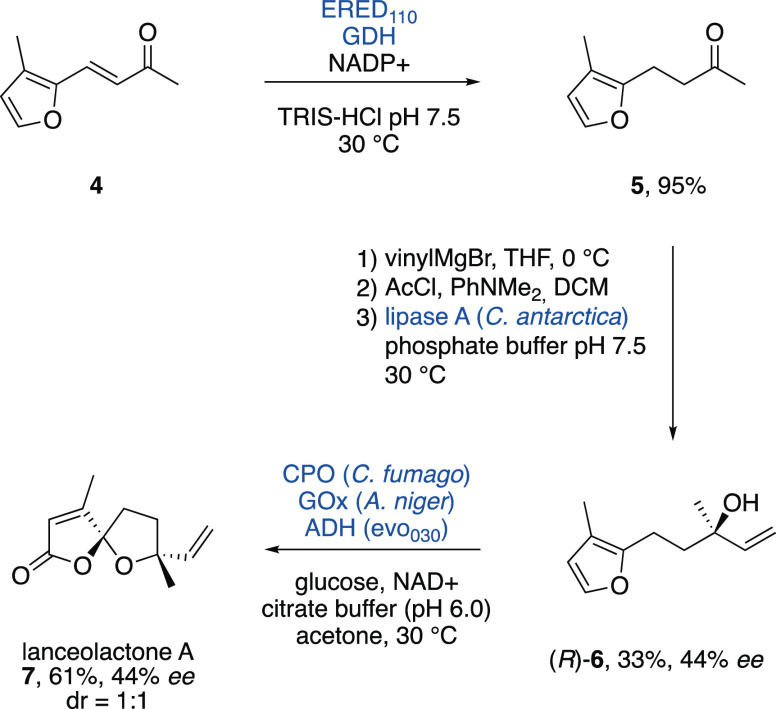
Chemoenzymatic Total Synthesis of Lanceolactone A (**7**)

Finally, we set out to implement the spirolactonization tool as the module in a multienzymatic retrosynthetic design (Scheme 6). Here, we developed a new chemoenzymatic route to the spirocyclic lanceolactone A, which was isolated from the traditional Chinese medicine plant *Illicium lanceolatum* and reported in 2015.^18^ We commenced our synthesis from enone **4** with a chemoselective reduction of the olefinic double bond with the commercial enoate reductase ERED_110_, coupled with glucose dehydrogenase (GDH) and glucose as cofactor regeneration system. The advantage of this biocatalytic approach lies in the specificity of the ERED for the olefin (95% yield of **5**), while chemical reduction with, e.g., Pd/C afforded almost exclusively the corresponding tetrahydrofuran through overreduction. In order to render our strategy enantioselective, we chose an enzymatic kinetic resolution of the tertiary allylic acetate intermediate in order to obtain optically enriched **6**. Thus, Grignard addition of vinyl magnesium bromide and subsequent acetylation gave rise to the necessary racemic ester **6**-OAc. Lipases and esterases with a conserved GGG(A)-X motif were discovered to show catalytic activities to esters of tertiary alcohol, even though high enantioselectivities are all but illusive for these lipase substrates.^19^ Thus, three enzymes, lipase A from *C. antarctica* (CALA), pig liver esterase (PLE), and lipase from *C. rugosa* (CLR), were tested in the hydrolytic kinetic resolution of *rac*-**6**-OAc (Figure S3). Lipase A managed to provide the highest selectivity in this very challenging resolution producing (*R*)-**5** in 33% yield with 44% *ee*. (*R*)-**6** was finally treated with CPO/GOx/evo_030_ and a separable mixture of the target product lanceolactone A (**7**) and its epimer (dr = 1:1) was obtained in an overall yield of 61%.

## Conclusions

In summary, we have presented a multienzymatic pathway for the direct oxidative rearrangement of furylpropanols to yield complex spirocyclic building blocks. The combination of an oxygenating peroxidase, an oxidase, and a ketoreductase provides a highly efficient one-pot system for the production of stereochemically defined oxa-spirolactones from the biorefinery-derived furan platform. The applicability of the method has been successfully demonstrated through its incorporation into more complex chemoenzymatic routes, including in the total syntheses of crassalactone D and lanceolactone A. Moreover, an alternative, oxidase-free pathway has been discovered, and implications of this peroxidase-nicotinamide cross-reactivity will be elucidated in depth in our future studies. While the observed diastereoselectivity was attributed to the thermodynamic bias rather than the commercial biocatalysts, the conformational analysis of key intermediates offers clear hints for the design and evolution of next-generation enzymes to address this synthetically appealing challenge.

## Supporting Information Available

Experimental procedures, configurational analysis, computational details and optimized geometries, spectral data for all new compounds, chromatographic traces, and supplementary tables and figures (PDF)
