# Supplementary material for: Fully Biocatalytic Rearrangement of Furans to Spirolactones
Source: ACS Catal. 2023 May 15;13(11):7256–62. doi: 10.1021/acscatal.3c00132 (PMC10242749; doi:10.1021/acscatal.3c00132)
Supplement: Supplementary file 1 — cs3c00132_si_001.pdf [file cs3c00132_si_001.pdf]

Fully Biocatalytic Oxidative Rearrangement of Furyl Alcohols to Spirolactones

Yu Chang Liu[a,b*], JD Rolfes,[c] Joel Björklund,[b] Jan Deska[a,b *]

(a) Department of Chemistry, University of Helsinki. A.I. Virtasen aukio 1, 00560 Helsinki, Finland

(b) Department of Chemistry, Aalto University, Kemistintie 1, 02150 Espoo, Finland

(c) Albert Hofmann Institute for Physiochemical Sustainability. Albert-Schweitzer-Str. 22, 32602 Vlotho, Germany.

Yu Chang Liu [a*] - yu-chang.liu@helsinki.fi.

Jan Deska [a*]- jan.deska@helsinki.fi

Table of contents

1. General remarks 2

2. Screening & optimization of conditions for the dehydrogenation of **3a** 3

3. Investigation on the oxidation of NADH by chloroperoxidase 4

4. Procedures & Analytical Data 5

4.1 Enzymatic preparation of spiro-lactones 5

4.2 Chemoenzymatic total synthesis of lanceolactone A 10

4.3. Preparation of substrates 12

5. Configurational analysis 20

5.1 Density functional theory 20

5.2 NMR spectroscopy 31

6. Spectra & chromatograms 36

6.1. NMR spectra of the products 36

6.2 NMR spectra of the substrates 54

6.3 HPLC traces 66

7. Supplementary references 61

1. General Remarks

Alcohol dehydrogenases and enoate reductase ERED-110 were purchased from Codexis (Codexis Screening Kit) and from evocatal GmbH (*evo* 1.1.030 and *evo* 1.1.200). Glucose dehydrogenase from *Pseudomonas* sp. (200 U/mg, Product No. 19359), glucose oxidase from *Aspergillus niger* (100 U/mg, Product No. G7141), chloroperoxidase from *Caldariomyces fumago* (10 U/µl, Product No. 25810), lipase A from *Candida antarctica* immobilized on Immobead 150 (2 U/mg, CALA, Product No. 41658), lipase from *Candida rugosa* (700 U/mg, CRL, Product No. L1754) and pig liver esterase immobilized Eupergit® C (0.2 U/mg, PLE, Product No. 46064) were purchased from Sigma Aldrich.

Enzymatic reactions were performed under non inert conditions on an orbital shaker in caped glass vials. All other reactions, unless stated otherwise were carried out under argon atmosphere with dry solvents using anhydrous conditions. Dry solvents were taken from a solvent drying system MB-SPS-800 from M-Braun. Commercially available reagents were used without further purification.

Column chromatography was performed with silica gel from Merck (Millipore 60, 40-60 µm, 240-400 mesh). Reactions were monitored by thin layer chromatograhy (TLC) carried out on Machery-Nagel precoated silica gel plates (TLC Silica gel 60 F254). Visualisation of the TLC plates was done by using UV light and staining with a basic potassium permanganate solution.

^{1}H- and ^{13}C-NMR spectra were recorded on a Bruker AV-400 instrument at 20 °C. Chemical shifts are reported in parts per million (ppm) calibrated using residual non-deuterated solvents as internal reference [$CHCl_3$ at δ = 7.26 ppm (^{1}H NMR) and 77.16 ppm (^{13}C NMR)]. Infrared spectra were recorded on a Bruker ALPHA Eco-ATR spectrometer, absorption bands are reported in wave numbers [cm^{-1}].

High resolution mass spectrometry was performed on an Agilent 6530 (Q-TOF) mass spectrometer. Optical rotations were measured on an Autopol VI – automatic polarimeter from Rudolph Research Analytical. High performance liquid chromatography analysis was performed on an Agilent 1100 system with a G1312A binary pump and a G1312B diode array detector using analytical Daicel Chiralpak column (250 mm x 4.6 mm; AS or OD-H).

Computational Methods:

All computations were performed with the ORCA program package.[1,2] Geometry optimizations were performed as density functional theory (DFT) calculations with the PBE0 functional[3] and the def2-TZVP basis set[4] including Grimme's D3 dispersion correction with the Becke–Johnson damping scheme (ORCA keyword "D3").[5,6] The resolution of identity approximation[7] was used for the Coulomb integrals with the def2/J auxiliary basis set[8] and for the Hartree-Fock exchange terms with the chain of spheres (COSX) approximation (ORCA keyword "RIJCOSX").[9] Starting geometries were built from scratch using the molecular builder in the Avogadro[10] program. Local minima were confirmed through frequency analysis. Self-consistent field (SCF) and optimization convergence criteria were set tightly (ORCA keywords "TightSCF" and "TightOpt").

For single point energies, domain-based pair natural orbital coupled cluster calculations with single and double, and perturbative triple excitations (DLPNO-CCSD(T))[11-15] were performed, with the def2-TZVPP basis sets[4] and def2-TZVPP/C auxiliary basis sets.[16] SCF convergence criteria were set tightly, and solvation in water was modelled with the solvation model based on density (SMD).[17]

Gibbs Free Energies were calculated by addition of the thermal corrections to Gibbs Free Energy from the DFT geometry optimization calculations to the electronic energies of the single point calculations.

2. Screening and optimization of conditions for the dehydrogenation of 3a

enzyme	yield (%)	enzyme	yield (%)
P1-A04	5	P2-C02	5
P1-A12	2	P2-D11	4
P2-B02	63	P2-D12	27
P1-B05	23	P2-H07	9
P1-B10	5	evo200	33
P1-B12	68	evo030	71
P1-C01	21	GDH	0
P1-H08	26	GOx	0
P2-B02	45		

Supplementary Table 1. Screening biocatalytic dehydrogenation of **2a.** Reaction condition: step 1, **1a** (6.5 mM), D-Glucose (10 mM), glucose oxidase (0.12 µM, 1 U), chloroperoxidase (0.14 µM, 10 U), citrate buffer (1.0 mL, 100 mM, pH 6.0), 30 °C for 1h; step 2, acetone (5 µl), NAD+ (2 mM), alcohol dehdyrogenase (2 mg) (glucose dehydrogenase 20U, or glucose oxidase 20U). Yield determined by NMR using methylsufonylmethane as standard.

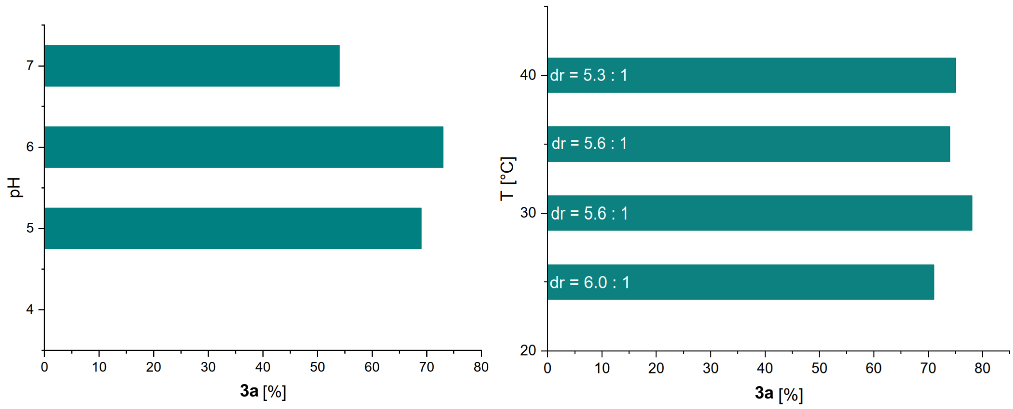

Supplementary Figure 1. Influence of pH and temperature. a) Influence of pH on production of **2a.** b) Influence of temperature on production of **3a.** Reaction condition: step 1, 1a (6.5 mM), D-Glucose (10 mM), glucose oxidase (0.12 µM, 1 U), chloroperoxidase (0.14 µM, 10 U), citrate buffer (1.0 mL, 100 mM, variable pH in Figure 1a), 30 °C (variable temperature in Figure 1b) for 1h; step 2, acetone (5 µl), NAD+ (2 mM), KRED evo_{030} (2 mg). Yield determined by NMR using methylsufonylmethane (MSM) as standard.

3. Investigation on the oxidation of NADH by chloroperoxidase

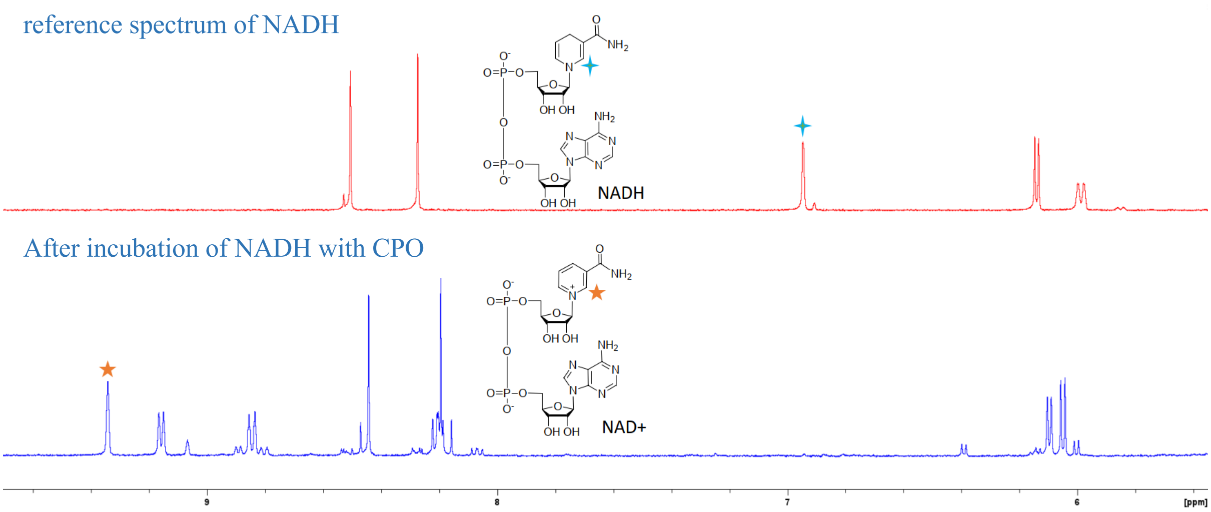

Supplementary Figure 2. Investigation on oxidation of NADH by chloroperoxidase. Aerobic oxidation of NADH by chloroperoxidase under substrate-free conditions. Reaction conditions: NADH (5mM) in deuterium oxide citrate buffer (2mL, 100mM, pH 6.0), chloroperoxidase (2 µl), 30 °C, 2h.

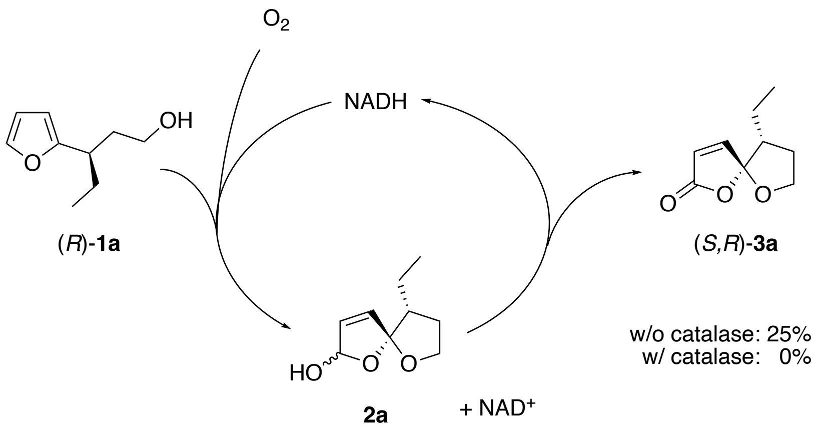

Supplementary Scheme 1. Investigations on a redox self-sufficient system. a) combination between CPO and evo030, b) Inhibition of oxidant-free spirolactonization by catalase. Reaction conditions: **1a** (1 mg) in deuterium oxide citrate buffer (1mL, 100 mM, pH 6.0), NADH (2 mM), dimethyl sulfone (0.5 mM, as internal standard), chloroperoxidase (1 µl) and alcohol dehydrogenase evo030 (2 mg), 30 °C, 20h. Quantitative ^{1}H NMR was employed to measure the yield relative to MSM.

4. Procedures & Analytical Data

4.1 Enzymatic preparation of spiro-lactones

9-Ethyl-1,6-dioxaspiro[4.4]non-3-en-2-one (3a)

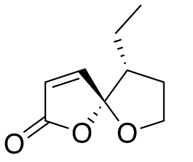

3a

In a 50 mL Erlenmeyer flask, substrate (*R*)-**1a** (15 mg, 97 µmol) was dissolved in a mixture of citrate buffer (15 mL, 100 mM, pH 5.5) and *t*-butanol (0.75 mL). Glucose (53 mg, 0.3 mmol), NAD^+ (9.9 mg, 15 µmmol), acetone (78 µl, 10 equiv.), chloroperoxidase (200 U), glucose oxidase (20 U) and alcohol dehydrogenase *evo* 1. 1. 030 (20 mg) were added and the solution was incubated at 30 °C for 20h. The reaction mixture was extracted with ethyl acetate (3×10 mL). The combined organic phases were dried over Na_2SO_4, concentrated under reduced pressure, and the residue was purified by column chromatography (*n*-hexane/ ethyl acetate 1/1) to afford **3a** (12.7 mg, 76 µmol, 78%, 5.7:1 dr) as a colorless oil. $\mathbf{R_f}$ (*n*-hexane/ethyl acetate 3/1): 0.33. Major diastereomer: **^{1}H-NMR** (400 MHz, $CDCl_3$): δ [ppm] = 7.05 (d, *J* = 5.6 Hz, 1H), 6.16 (d, *J* = 5.6 Hz, 1H), 4.24 (dq, *J* = 8.3 Hz, *J* = 2.3 Hz, 1H), 3.99-4.05 (m, 1H), 2.26-2.35 (m, 1H), 2.12-2.21 (m, 1H), 1.90-2.04 (m, 1H), 1.33-1.42 (m, 2H), 0.93 (t, *J* = 7.5 Hz, 3H). **^{13}C-NMR** (400 MHz, $CDCl_3$): δ [ppm] = 170.2, 151.9, 125.0, 115.1, 69.3, 48.6, 29.9, 20.7, 12.8. Minor diastereomer (selected signals): **^{1}H-NMR** (400 MHz, $CDCl_3$): δ [ppm] = 7.15 (d, *J* = 5.7 Hz, 1H), 6.17 (d, *J* = 5.7 Hz, 1H), 0.95 (t, *J* = 7.4 Hz, 3H). **FT-IR** (ATR): ν [cm^{-1}] = 3430 (s), 2955 (m), 1734 (s), 1167 (m), 1091 (m), 830 (m). HRMS (ESI+): m/z $[M+H]^+$ calcd for $C_9H_{12}O_3$: 168.0786; found: 168.0860.

9-Propyl-1,6-dioxaspiro[4.4]non-3-en-2-one (3b)

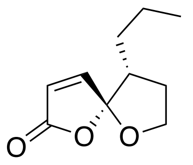

3b

In analogy to the synthesis of **3a**, **3b** (10.5 mg, 58 µmol, 65%, 5.4:1 dr) was obtained from **1b** (15 mg, 89 µmol) as a colorless oil. $\mathbf{R_f}$ (*n*-hexane/ethyl acetate 3/1): 0.33. Major diastereomer: **^{1}H-NMR** (400 MHz, $CDCl_3$): δ [ppm] = 7.04 (d, *J* = 5.5 Hz, 1H), 6.16 (d, *J* = 5.5 Hz, 1H), 4.21-4.26 (m, 1H), 3.98-4.05 (m, 1H), 2.19-2.33 (m, 2H), 1.91-1.99 (m, 1H), 1.20-1.43 (m, 4H), 0.89 (t, *J* = 7.1 Hz, 3H). **^{13}C-NMR** (400 MHz, $CDCl_3$): δ [ppm] =170.2, 151.8, 124.6, 115.2, 69.3, 46.6, 30.2, 29.6, 21.5, 14.2. Minor diastereomer (selected signals): **^{1}H-NMR** (400 MHz, $CDCl_3$): δ [ppm] = 7.15 (d, *J* = 5.7 Hz, 1H), 2.45-1.50 (m, 1H), 3.34-2.43 (m, 1H), 1.81-1.90 (m, 2H), 0.95 (t, *J* = 7.4 Hz, 3H). **FT-IR** (ATR): ν [cm^{-1}] = 3430 (s), 2955 (m), 1737 (s), 1167 (m), 1091 (m), 836 (m). HRMS (ESI+): m/z $[M+H]^+$ calcd for $C_{10}H_{14}O_3$: 182.0943; found: 182.1012.

9-Isopropyl-1,6-dioxaspiro[4.4]non-3-en-2-one (3c)

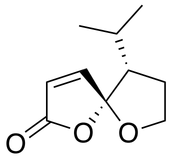

3c

In analogy to the synthesis of **3a**, **3c** (8.9 mg, 49 µmol, 55%, 5.4:1 dr) was obtained from **1c** (15 mg, 89 µmol) as a colorless oil. **R*f*** (*n*-hexane/ethyl acetate 3/1): 0.33. Major diastereomer: **^{1}H-NMR** (400 MHz, $CDCl_3$): δ [ppm] = 7.12 (d, *J* = 5.5 Hz, 1H), 6.13 (d, *J* = 5.5 Hz, 1H), 4.15-4.20 (m, 1H), 3.92-3.98 (m, 1H), 2.21-2.31 (m, 1H), 1.98-2.11 (m, 2H), 1.70-1.92 (m, 1H), 0.95 (t, *J* = 6.8 Hz, 3H), 0.81 (t, *J* = 6.6 Hz, 3H). **^{13}C-NMR** (400 MHz, $CDCl_3$): δ [ppm] = 170.6, 153.6, 124.3, 114.5, 68.4, 53.7, 29.4, 27.5, 22.4, 22.2, 12.8. Minor diastereomer (selected signals): **^{1}H-NMR** (400 MHz, $CDCl_3$): δ [ppm] = 7.19 (d, *J* = 5.7 Hz, 1H), 6.17 (d, *J* = 5.7 Hz, 1H), 0.93 (t, *J* = 6.8 Hz, 3H), 0.89 (t, *J* = 6.6 Hz, 3H). **FT-IR** (ATR): ν [cm^{-1}] = 3429 (s), 2955 (m), 1737 (s), 1167 (m), 1091 (m), 836 (m). HRMS (ESI+): m/z [M+H]$^+$ calcd for $C_{10}H_{14}O_3$: 182.0943; found: 182.1012.

9-Cyclopentyl-1,6-dioxaspiro[4.4]non-3-en-2-one (3d)

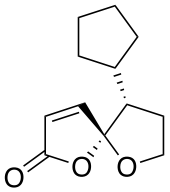

3d

In analogy to the synthesis of **3a**, **3d** (5.1 mg, 25 µmol, 32%, 5.6:1 dr) from **1d** (15 mg, 77 µmol) as a colorless oil. **R*f*** (*n*-hexane/ethyl acetate 3/1): 0.33. Major diastereomer: **^{1}H-NMR** (400 MHz, $CDCl_3$): δ [ppm] = 7.09 (d, *J* = 5.6 Hz, 1H), 6.11 (d, *J* = 5.6 Hz, 1H), 4.15-4.20 (m, 1H), 3.93-3.99 (m, 1H), 2.18-2.24 (m, 1H), 2.00-2.16 (m, 2H), 1.74-1.92 (m, 2H), 1.51-1.62 (m, 4H), 1.40-1.50 (m, 2H), 1.07-1.22 (m, 1H). **^{13}C-NMR** (400 MHz, $CDCl_3$): δ [ppm] = 170.5, 153.0, 123.7, 114.6, 68.9, 53.0, 39.4, 32.6, 32.0, 30.3, 25.4, 24.5. Minor diastereomer (selected signals): **^{1}H-NMR** (400 MHz, $CDCl_3$): δ [ppm] = 7.16 (d, *J* = 5.6 Hz, 1H), 6.11 (d, *J* = 5.6 Hz, 1H), 4.05-4.09 (m, 2H). **FT-IR** (ATR): ν [cm^{-1}] = 3429 (s), 2955 (m), 1735 (s), 1167 (m), 1091 (m), 830 (m). HRMS (ESI+): m/z [M+H]$^+$ calcd for $C_{12}H_{16}O_3$: 208.1099; found: 208.1174.

8-Ethyl-1,6-dioxaspiro[4.4]non-3-en-2-one (3e)

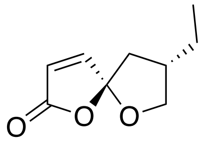

3e

In analogy to the synthesis of **3a**, **3e** (13.2 mg, 79 µmol, 81%, 2.5:1 dr) was obtained from **1e** (15 mg, 97 µmol) as a colorless oil. **R*f*** (*n*-hexane/ethyl acetate 3/1): 0.33. Major diastereomer: **^{1}H-NMR** (400 MHz, $CDCl_3$): δ [ppm] = 7.08 (d, *J* = 5.5 Hz, 1H), 6.10 (d, *J* = 5.5 Hz, 1H), 4.23 (t, *J* = 7.3 Hz, 1H), 3.67 (t, *J* = 8.3 Hz, 1H), 2.59-2.71 (m, 1H), 2.30 (dd, *J* = 12.9 Hz, *J* = 6.8 Hz, 1H), 1.82 (dd, *J* = 12.9 Hz, *J* = 10.7 Hz, 1H), 1.43-1.55 (m, 2H), 0.95 (t, *J* = 7.4 Hz, 3H). **^{13}C-NMR** (400 MHz, $CDCl_3$): δ [ppm] = 152.1, 124.3, 114.9, 75.8, 41.7, 39.8, 26.1, 12.8. Minor diastereomer (selected signals): **^{1}H-NMR** (400 MHz, $CDCl_3$): δ [ppm] = 7.10 (d, *J* = 5.5 Hz, 1H), 6.12 (d, *J* = 5.5 Hz, 1H), 4.34 (t, *J* = 8.1 Hz, 1H), 3.84 (dd, *J* = 8.4 Hz, *J* = 7.0 Hz, 1H), 0.93 (t, *J* = 7.4 Hz,

3H). **^{13}C-NMR** (400 MHz, CDCl3): δ [ppm] = 152.3, 123.6, 40.7, 40.4, 12.9. **FT-IR** (ATR): ν [cm^{-1}] = 3429 (s), 2955 (m), 1734 (s), 1167 (m), 1091 (m), 827 (m). HRMS (ESI+): m/z [M+H]$^+$ calcd for $C_9H_{12}O_3$: 168.0786; found: 168.0861.

8-Isopropyl-1,6-dioxaspiro[4.4]non-3-en-2-one (3f)

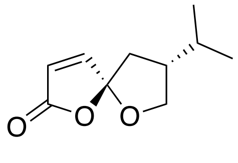

3f

In analogy to the synthesis of **3a**, **3f** (12.5 mg, 69 μmol, 77%, 3.1:1 dr) was obtained from **1f** (15 mg, 89 μmol) as a colorless oil. **R*f*** (*n*-hexane/ethyl acetate 3/1): 0.33. Major diastereomer: **^{1}H-NMR** (400 MHz, $CDCl_3$): δ [ppm] = 7.10 (d, *J* = 5.5 Hz, 1H), 6.12 (d, *J* = 5.5 Hz, 1H), 4.32 (t, *J* = 8.2 Hz, 1H), 3.73 (t, *J* = 8.5 Hz, 1H), 4.32 (t, *J* = 8.3 Hz, 1H), 3.73 (t, *J* = 8.6 Hz, 1H), 2.43-2.53 (m, 1H), 2.23 (dd, *J* = 12.7 Hz, *J* = 6.7 Hz, 1H), 1.86 (dd, *J* = 12.4 Hz, *J* = 11.6 Hz, 1H), 1.50-1.61 (m, 1H), 0.95 (d, *J* = 6.6 Hz, 3H), 0.95 (d, *J* = 6.6 Hz, 3H), 0.92 (d, *J* = 6.6 Hz, 3H). **^{13}C-NMR** (400 MHz, $CDCl_3$): δ [ppm] = 152.0, 124.4, 115.1, 74.9, 47.0, 45.4, 40.6, 39.2, 32.1, 31.3, 21.5, 21.4. Minor diastereomer (selected signals): **^{1}H-NMR** (400 MHz, $CDCl_3$): δ [ppm] = 7.08 (d, *J* = 5.5 Hz, 1H), 6.09 (d, *J* = 5.5 Hz, 1H), 4.19 (t, *J* = 7.3 Hz, 1H), 3.86 (t, *J* = 8.8 Hz, 1H). **FT-IR** (ATR): ν [cm^{-1}] = 3429 (s), 2955 (m), 1734 (s), 1167 (m), 1091 (m), 827 (m). HRMS (ESI+): m/z [M+H]$^+$ calcd for $C_{10}H_{14}O_3$: 182.0943; found: 182.1016.

8-Cyclopentyl-1,6-dioxaspiro[4.4]non-3-en-2-one (3g)

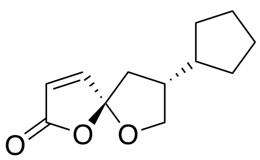

3g

In analogy to the synthesis of **3a**, **3g** (5.6 mg, 27 μmol, 35%, 2.7:1 dr) was obtained from **1g** (15 mg, 77 μmol) as a colorless oil. **R*f*** (*n*-hexane/ethyl acetate 3/1): 0.33. Major diastereomer: **^{1}H-NMR** (400 MHz, $CDCl_3$): δ [ppm] = 7.10 (d, *J* = 5.5 Hz, 1H), 6.12 (d, *J* = 5.5 Hz, 1H), 4.31 (t, *J* = 8.2 Hz, 1H), 3.72 (t, *J* = 8.5 Hz, 1H), 2.52-2.63 (m, 1H), 2.26 (dd, *J* = 12.7 Hz, *J* = 6.7 Hz, 1H), 1.88 (dd, *J* = 12.8 Hz, *J* = 11.1 Hz, 1H), 1.51-1.79 (m, 8H), 1.12-1.27 (m, 1H). **^{13}C-NMR** (400 MHz, $CDCl_3$): δ [ppm] = 152.1, 124.3. 115.0, 75.3, 43.8, 43.7, 41.4, 31.7, 31.5, 25.3, 25.1. Minor diastereomer (selected signals): **^{1}H-NMR** (400 MHz, $CDCl_3$): δ [ppm] = 7.08 (d, *J* = 5.5 Hz, 1H), 6.10 (d, *J* = 5.5 Hz, 1H), 4.19 (t, *J* = 7.6 Hz, 1H), 3.89 (t, *J* = 8.2 Hz, 1H), 2.39 (dd, *J* = 13.5 Hz, *J* = 9.4 Hz, 1H), 2.10 (dd, *J* = 13.5 Hz, *J* = 5.8 Hz, 1H). **FT-IR** (ATR): ν [cm^{-1}] = 3429 (s), 2955 (m), 1734 (s), 1167 (m), 1091 (m), 830 (m). HRMS (ESI+): m/z [M+H]$^+$ calcd for $C_{12}H_{16}O_3$: 208.1099; found: 208.1172.

8-Phenyl-1,6-dioxaspiro[4.4]non-3-en-2-one (3h)

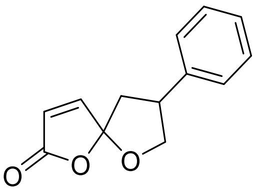

3h

In analogy to the synthesis of **3a**, **3h** (4.8 mg, 22 µmol, 30%, 3.2:1 dr) was obtained from **1h** (15 mg, 74 µmol) as a colorless oil. $\mathbf{R}_f$ (*n*-hexane/ethyl acetate 3/1): 0.32. Major diastereomer: **^{1}H-NMR** (400 MHz, $CDCl_3$): δ [ppm] = 7.24-7.37 (m, 5H), 7.21 (d, *J* = 5.5 Hz, 1H), 6.19 (d, *J* = 5.5 Hz, 1H), 4.60 (t, *J* = 8.2 Hz, 1H), 4.06 (t, *J* = 8.3 Hz, 1H), 3.94-4.01 (m, 1H), 2.57 (dd, *J* = 13.0 Hz, *J* = 6.9 Hz, 1H), 2.38 (dd, *J* = 12.9 Hz, *J* = 11.1 Hz, 1H). **^{13}C-NMR** (400 MHz, $CDCl_3$): δ [ppm] = 151.6, 139.7, 129.0, 127.4, 127.2, 126.8, 114.7, 76.6, 43.7, 43.4. Minor diastereomer (selected signals): **^{1}H-NMR** (400 MHz, $CDCl_3$): δ [ppm] = 7.18 (d, *J* = 5.5 Hz, 1H), 6.17 (d, *J* = 5.5 Hz, 1H), 4.46 (t, *J* = 8.3 Hz, 1H), 3.62-3.71 (m, 1H), 2.80 (dd, *J* = 14.3 Hz, *J* = 10.6 Hz, 1H), 2.48 (dd, *J* = 14.3 Hz, *J* = 7.4 Hz, 1H). **FT-IR** (ATR): ν [cm^{-1}] = 3430 (s), 2955 (m), 1734 (s), 1167 (m), 1091 (m), 832 (m). HRMS (ESI+): m/z $[M+Na]^+$ calcd for $C_{13}H_{12}O_3$: 216.0786; found: 216.0680.

8-Hydroxy-7-methyl-1,6-dioxaspiro[4.4]non-3-en-2-one (3i)

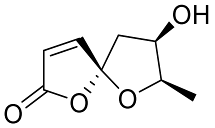

3i

In analogy to the synthesis of **3a**, **3i** (6.5 mg, 38 µmol, 40%, 1.4:1 dr) was obtained from **1i** (15 mg, 96 µmol) as a colorless oil. $\mathbf{R}_f$ (*n*-hexane/ethyl acetate 1/1): 0.21. Major diastereomer: **^{1}H-NMR** (400 MHz, $CDCl_3$): δ [ppm] = 7.20 (d, *J* = 5.6 Hz, 1H), 6.09 (d, *J* = 5.5 Hz, 1H), 4.48 (br, 1H), 4.39-4.45 (m, 1H), 2.68 (d, *J* = 5.8 Hz, 1H), 2.37 (d, *J* = 2.0 Hz, 1H), 1.95 (bs, 1H), 1.33 (d, *J* = 6.4 Hz, 1H). **^{13}C-NMR** (400 MHz, $CDCl_3$): δ [ppm] =170.3, 153.1, 123.0, 113.5, 81.1, 73.1, 44.5, 13.7. Minor diastereomer (selected signals): **^{1}H-NMR** (400 MHz, $CDCl_3$): δ [ppm] = 2.72 (d, *J* = 5.8 Hz, 1H), 2.33 (d, *J* = 2.0 Hz, 1H). **FT-IR** (ATR): ν [cm^{-1}] = 3430 (s), 2955 (m), 1734 (s), 1167 (m), 1091 (m), 832 (m). HRMS (ESI+): m/z $[M+H]^+$ calcd for $C_8H_{10}O_4$: 170.0579; found: 170.0535.

7-epi-Crassalactone D (3j)

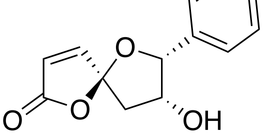

3j

In analogy to the synthesis of **3a, 1j** (15 mg, 69 µmol) was converted affording a separable mixture of **3j** and epi-**3j** (3.2 mg, 14 µmol, 20%, 1:1 dr) as a white solid. **3j**: $[\alpha]_{20}^{D}$: -62.8 (c 0.29, ethanol). {lit. $[\alpha]_{20}^{D}$: -64 (0.3, ethanol)}.[18] $\mathbf{R}_f$ (*n*-hexane/ethyl acetate 1/1) 0.36. **^{1}H-NMR** (400 MHz, $CDCl_3$): δ [ppm] = 7.35-7.45 (m, 6H), 6.16 (d, *J* = 5.5 Hz, 1H), 5.43 (d, *J* = 3.1 Hz, 1H), 4.61-4.65 (m, 1H), 2.83 (ddd, *J* = 14.8 Hz, *J* = 13.3 Hz, *J* = 1.5 Hz, 1H), 2.54 (dd, *J* = 14.7 Hz, *J* = 1.3 Hz), 1.30 (t, *J* = 1.8 Hz, 1H). **^{13}C-NMR** (100 MHz, $CDCl_3$): δ [ppm] = 152.9, 128.9, 128.8, 126.7, 122.9, 86.7, 73.8, 43.2.

epi-**3j**: $[\alpha]_{20}^{D}$: -33.9 (c 0.28, ethanol). {lit. $[\alpha]_{20}^{D}$: -48.2 (c 0.2, ethanol)}.[18] **R*f*** (*n*-hexane/ethyl acetate 1/1): 0.16. **^{1}H-NMR** (400 MHz, $CDCl_3$): δ [ppm] = 7.40 (d, *J* = 4.5 Hz, 4H), 7.32-7.38 (m, 1H), 7.20 (d, *J* = 5.6 Hz, 1H), 6.24 (d, *J* = 5.6 Hz, 1H), 5.39 (d, *J* = 4.1 Hz, 1H), 4.57-4.59 (m, 1H), 2.63 (dd, *J* = 14.0 Hz, *J* = 4.9 Hz, 1H), 2.53 (d, *J* = 14.0 Hz, 1H), 1.99 (d, *J* = 7.8 Hz, 1H). **^{13}C-NMR** (100 MHz, $CDCl_3$): δ [ppm] = 169.3, 151.8, 135.2, 128.7, 128.5, 126.8, 124.6, 114.2, 89.0, 72.9, 44.4.

Crassalactone D (3k)

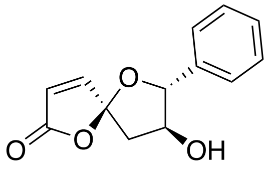

3k

In analogy to the synthesis of **2a**, **1k** (15 mg, 69 µmol) was converted affording a separable mixture of **3k** and epi-**3k** (4.1 mg, 18 µmol, 26%, 1:1 dr) as a white solid. **3k**: $[\alpha]_{20}^{D}$: 67.3 (c 0.25, ethanol). {lit. $[\alpha]_{20}^{D}$: 13.6 (0.2, ethanol)}.[18] **R*f*** (*n*-hexane/ethyl acetate 1/1): 0.36. Major diastereomer: **^{1}H-NMR** (400 MHz, $CDCl_3$): δ [ppm] = 7.37-7.40 (m, 2H), 7.30-7.34 (m, 3H), 7.29 (d, *J* = 5.6 Hz, 1H), 6.28 (d, *J* = 5.6 Hz, 1H), 5.40 (d, *J* = 2.2 Hz, 1H), 4.39-4.44 (m, 1H), 2.76 (d, *J* = 10.1 Hz, 1H), 2.56 (dd, *J* = 14.2 Hz, *J* = 6.3 Hz, 1H), 2.30 (dd, *J* = 14.2 Hz, *J* = 1.6 Hz, 1H). **^{13}C-NMR** (100 MHz, $CDCl_3$): δ [ppm] = 169.1, 151.0, 138.5, 128.8, 128.3, 125.1, 125.0, 114.4. 91.5, 78.3, 42.5.

epi-**3k**: $[\alpha]_{20}^{D}$: 20.4 (c 0.23, ethanol). {lit. $[\alpha]_{24}^{D}$: 28 (0.5, ethanol)}.[19] **R*f*** (*n*-hexane/ethyl acetate 1/1): 0.34. **^{1}H-NMR** (400 MHz, $CDCl_3$): δ [ppm] = 7.30-7.40 (m, 5H), 7.27 (d, *J* = 5.5 Hz, 1H), 6.17 (d, *J* = 5.5 Hz, 1H), 4.96 (d, *J* = 6.3 Hz, 1H), 4.61-4.65 (m, 1H), 2.58 (dd, *J* = 13.0 Hz, *J* = 6.0 Hz, 1H), 2.58 (dd, *J* = 13.0 Hz, *J* = 6.0 Hz, 1H), 2.45 (dd, *J* = 13.0 Hz, *J* = 8.4 Hz, 1H), 2.26 (dd, *J* = 7.5 Hz, *J* = 4.6 Hz, 1H). **^{13}C-NMR** (100 MHz, $CDCl_3$): δ [ppm] = 152.4, 138.9, 128.7, 128.4, 126.2, 125.9, 123.8, 113.2. 89.1, 77.5, 42.8.

4.2 Chemoenzymatic total synthesis of lanceolactone A

4-(3-Methylfuran-2-yl)butan-2-one (5)

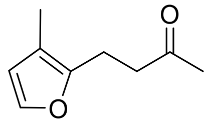

5

In a 100 mL Erlenmeyer flask, substrate enone **4** (50mg, 0.33 mmol, in isopropanol (5%, w/v)) was dissolved in phosphate buffer (50mL, 100mM, pH 7.0), and then $NADP^+$ (2 mM), glucose (297 mg, 1.65 mmol), glucose dehydrogenase (25 mg) and ERED-110 (30 mg) were added in the mixture and the solution was incubated at 30 °C for 20h. The reaction mixture was extracted with ethyl acetate (3×15 mL). The combined organic phase was dried over Na_2SO_4, concentrated under reduced pressure, and the residue was purified by column chromatography (*n*-hexane/ethyl acetate = 10/1). 4-(3-methylfuran-2-yl)butan-2-one (**4**) (47.1 mg, 0.31 mmol, 95%) was obtained as colorless oil. $\mathbf{R_f}$ (*n*-hexane/ethyl acetate 7/1): 0.33. **^{1}H-NMR** (400 MHz, $CDCl_3$): δ [ppm] = 7.20 (d, *J* = 1.8 Hz, 1H), 6.14 (d, *J* = 1.7 Hz, 1H), 2.81-2.85 (m, 2H), 2.72-2.76 (m, 2H), 2.14 (s, 3H), 1.96 (s, 3H). **^{13}C-NMR** (400 MHz, $CDCl_3$): δ [ppm] = 207.8, 149.4, 140.1, 114.3, 112.9, 41.9, 30.1, 20.2, 9.8.

(*R*)-3-Methyl-5-(3-methylfuran-2-yl)pent-1-en-3-ol (6)

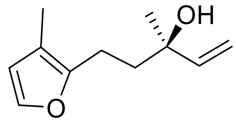

6

To a solution of **5** (300 mg, 1.97 mmol) in anhydrous THF (10 mL) was added a solution of vinylmagnesium bromide (2.95 mmol, 1.5 equiv., 1.0 M in THF) at 0 °C under argon. The mixture was stirred at the same temperature for 2h, then quenched with saturated aqueous NH_4Cl solution, extracted with ethyl acetate (3 x 15 mL). The combined organic phase was washed with brine and then dried over anhydrous Na_2SO_4 and concentrated under reduced pressure to afford crude *rac*-alcohol (**6**), which was utilized in the next step without purification. *rac*-**6** and N,N-dimethyl aniline (1 mL, 7.9 mmol) in dry dichloromethane (5 mL) was added acetyl chloride (914 mg, 11.8 mmol) at 0 °C under argon, then stirred at room temperature. After 24 h, the resulting mixture was diluted with ethyl acetate, washed with chloric acid (1.0 M) and brine, dried over anhydrous Na_2SO_4 and concentrated under reduced pressure. The residue was purified by column chromatography (*n*-hexane/ethyl acetate = 30/1), and the acetylated *rac*-**6-OAc** (323.6 mg, 1.46 mmol, 74%) was isolated as colorless oil. $\mathbf{R_f}$ (*n*-hexane/ethyl acetate 7/1): 0.41. **^{1}H-NMR** (400 MHz, $CDCl_3$): δ [ppm] = 7.20 (d, *J* = 1.8 Hz, 1H), 6.14 (d, *J* = 1.7 Hz, 1H), 5.97 (dd, *J* = 17.5 Hz, *J* = 10.9 Hz, 1H), 5.16 (dd, *J* = 17.5 Hz, *J* = 15.5 Hz, 2H), 2.58 (t, *J* = 8.2 Hz, 1H), 2.12-2.20 (m, 1H), 1.99-2.07 (m, 4H), 1.94 (s, 3H), 1.57 (s, 3H). **^{13}C-NMR** (400 MHz, $CDCl_3$): δ [ppm] = 170.0, 150.5, 141.5, 139.9, 113.8, 113.6, 112.9, 82.6, 38.3, 23.7, 22.3, 20.6, 9.9.

In a 25 mL Erlenmeyer flask, *rac*-**6-OAc** (50mg, 0.22 mmol, in DMSO (200μl)) was dissolved in phosphate buffer (2 mL, 100mM, pH 7.5), and lipase A from *Candida antartica* (CALA, 30 mg) was added, and the reaction mixture was incubated at 30 °C. The reaction was monitored by HPLC. The reaction mixture was extracted with ethyl acetate (4×5 mL). The combined organic phase was dried over anhydrous Na_2SO_4 and concentrated under reduced pressure. The residue was purified by flash column chromatography (SiO_2, n-hexane/ethyl acetate, 5/1) to give pure (*R*)-**6** (19 mg, 0.1 mmol, 45%, 44% ee) as colorless oil. $[\alpha]_{24}^{D}$: +10.9 (c 0.5, CH_3Cl). $\mathbf{R_f}$ (*n*-hexane/ethyl acetate 7/1): 0.35. **^{1}H-NMR** (400 MHz, $CDCl_3$): δ [ppm] = 7.20 (d, *J* = 1.8 Hz, 1H), 6.14 (d, *J* = 1.7 Hz, 1H), 5.92 (dd, *J* = 17.3 Hz, *J* = 10.6 Hz, 1H), 5.25 (dd, *J* = 17.3 Hz, *J* = 1.2 Hz, 1H), 5.09 (dd, *J* = 10.7 Hz, *J* = 1.2 Hz, 1H), 2.55-2.68 (m, 2H), 1.94 (s, 3H), 1.81-1.87 (m, 2H), 1.31 (s, 3H). **^{13}C-NMR** (400 MHz, $CDCl_3$): δ [ppm] = 150.9, 144.6, 139.9, 113.7, 112.9, 112.2, 73.1, 40.3, 28.1, 20.7, 9.9. **HPLC**

(column OD-H, 0.5 mL·min^{-1}, *n*-hexane/2-propanol 98/2): t_R ((*S*)-**6**) = 19.1 min (minor), t_R ((*R*)-**6**) = 20.0 min (major).

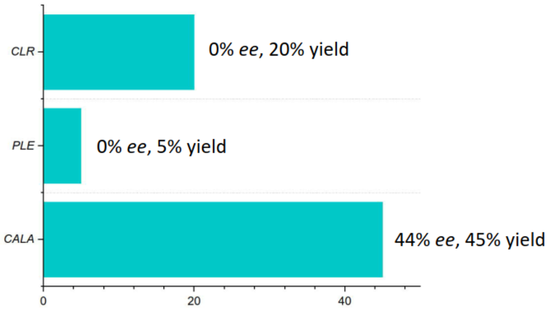

Supplementary Figure 3: Screening for enantioselective biocatalyst for the kinetic resolution of acetate *rac*-**6a**. In a 25 mL Erlenmeyer flask, *rac*-**6-OAc** (50mg, 0.22 mmol, in DMSO (200ul)) was dissolved in phosphate buffer (2 mL, 100mM, pH 7.5), and lipase A from *Candida antartica* (CALA, 30 mg), lipase from *Candida rugosa* (CRL, 30 mg), or pig liver esterase (PLE, 30 mg), was added respectively, and incubated at 30 °C. The reaction was monitored by HPLC and conversions and optical purities were determined.

Lanceolactone A (7)

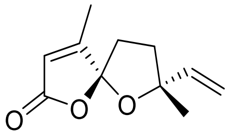

7

In a 50 mL Erlenmeyer flask, substrate (*R*)-**6** (15 mg, 83 µmol) were dissolved in a mixture of citrate buffer (15mL, 100mM, pH 6.0) and *t*-butanol (0.75 mL). Chloroperoxidase (200 U), H_2O_2 (19.5 mM, 3 equiv.), NAD^+ (2mM), acetone (78 µl, 10 equiv.) and alcohol dehydrogenase *evo* 1. 1. 030 (20 mg) were added and the solution was incubated at 30 °C for 20h. The reaction mixture was extracted with ethyl acetate (3×10 mL). The combined organic phases were dried over Na_2SO_4, concentrated under reduced pressure, and the residue was purified by column chromatography (*n*-hexane/ethyl acetate, 1/1) to afford a separable mixture of **lanceolactone A** (**7**) and epi-**7** (10 mg, 51 µmol, 61%, **7**:epi-**7** = 1:1) as a colorless oil. **7**: $[\alpha]_{24}^{D}$: +33.7 (c 0.25, $CHCl_3$). {lit. **7** $[\alpha]_{25}^{D}$: +46.4 (c 0.03, $CHCl_3$)}.[20] **R*f*** (*n*-hexane/ethyl acetate 5/1): 0.34. **^{1}H-NMR** (400 MHz, $CDCl_3$): δ [ppm] = 5.91 (dd, *J* = 17.2 Hz, *J* = 10.8 Hz, 1H), 5.84 (q, *J* = 1.6 Hz, 1H), 5.24 (dd, *J* = 17.3 Hz, *J* = 1.2 Hz, 1H), 5.06 (dd, *J* = 10.8 Hz, *J* = 1.2 Hz, 1H), 2.07-2.88 (m, 4H), 2.04 (d, *J* = 1.6 Hz, 3H), 1.51 (s, 3H). **^{13}C-NMR** (400 MHz, $CDCl_3$): δ [ppm] = 164.0, 142.3, 119.5, 115.2, 112.3, 87.6, 36.2, 34.4, 27.8, 12.7. **FT-IR** (ATR): ν [cm^{-1}] = 3430 (s), 2955 (m), 1734 (s).

epi-**7**: $[\alpha]_{24}^{D}$: -12.9 (c 0.17, $CHCl_3$). **R*f*** (*n*-hexane/ethyl acetate 5/1): 0.31. **^{1}H-NMR** (400 MHz, $CDCl_3$): δ [ppm] = 6.07 (dd, *J* = 17.2 Hz, *J* = 10.8 Hz, 1H), 5.83 (q, *J* = 1.6 Hz, 1H), 5.29 (dd, *J* = 17.3 Hz, *J* = 1.0 Hz, 1H), 5.10 (dd, *J* = 10.8 Hz, *J* = 1.0 Hz, 1H), 2.26-2.36 (m, 2H), 2.15-2.22 (m, 1H), 2.04 (d, *J* = 1.6 Hz, 3H), 1.96-2.03 (m, 1H), 1.39 (s, 3H). **^{13}C-NMR** (400 MHz, $CDCl_3$): δ [ppm] = 164.0, 142.6, 119.2, 115.2, 113.3, 87.5, 36.6, 34.3, 25.3, 12.3. **FT-IR** (ATR): ν [cm^{-1}] = 3430 (s), 2955 (m), 1734 (s).

4.3 Preparation of substrates

General procedures A for preparation of 1a, 1b, 1c and 1d

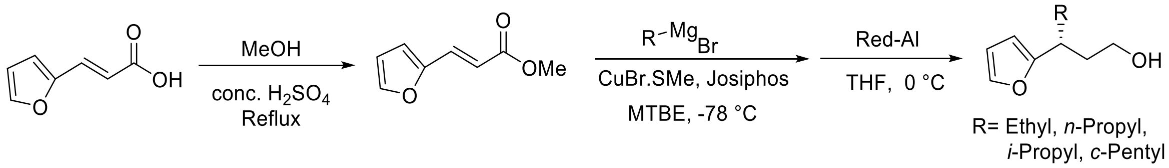

To the solution of *E*-3-(2-furyl)acrylic acid (5 g, 36.2 mmol) in methanol (50 mL), was added conc. H_2SO_4 (0.5 mL), and then the mixture was refluxed. After complete disappearance of the starting material monitored by TLC, the solvent was evaporated under reduced pressure. Water (20 mL) was added and the mixture was extracted with diethyl ether (3 × 15 mL). The combined organic layers were dried over anhydrous Na_2SO_4 and concentrated under reduced pressure. The residue was purified by flash column chromatography (SiO_2, pentane/diethyl ether, 5/1) to give pure methyl 3-(2-furyl)acrylate (4.39 g, 28.9 mmol, 80%) as white solid. **^{1}H-NMR** (400 MHz, $CDCl_3$): δ [ppm] = 7.48 (d, *J* = 1.6 Hz, 1H), 7.43 (d, *J* = 15.8 Hz, 1H), 6.60 (d, *J* = 3.4 Hz, 1H), 6.46 (dd, *J* = 3.4 Hz, *J* = 1.8 Hz, 1H), 6.31 (d, *J* = 15.8 Hz, 1H), 3.78 (s, 3H). **^{13}C-NMR** (400 MHz, $CDCl_3$): δ [ppm] = 168.3, 151.7, 145.6, 132.0, 116.3, 115.6, 113.1, 52.5.

According to a literature procedure,[21] to a Schlenk tube was added CuBr•SMe_2 (7.5µmol, 1.54 mg, 0.1% mol) and ligand (*R*)-1-(S_P)-2-(dicyclohexylphosphino)ferrocenylethyldiphenylphosphine (Josiphos SL-J004-1) (9.0 µmol, 5.4 mg, 0.12% equiv.) in tBuOMe (1.2 mL) and stirred under argon at room temperature for 30 min. The mixture was cooled to −78 °C and Grignard reagent (1.87 mmol, 2.5 equiv., 3.0 M in diethyl ether) was added dropwise. After stirring for 10 min at the same temperature, 3-(2-furyl)acrylate [0.75 mmol, 114mg in tBuOMe (1 mL)] was added dropwise sequentially. After 3h, the reaction was quenched by saturated aqueous NH_4Cl solution, extracted with diethyl ether (3 × 5mL). The combined organic phases were dried over anhydrous Na_2SO_4 and concentrated under reduced pressure, giving 1,4-addition product, which was used in the next step without purification. To a solution of crude methyl 3-methylfuran-2-carboxylate in anhydrous THF (10 mL) was added sodium dihydrido-bis(2-methoxyethoxy)aluminate (Red-Al) (1.5 equiv.) at 0 °C under argon, and the reaction mixture was stirred for 1 h. The reaction was then quenched with saturated aqueous NH_4Cl solution, extracted with diethyl ether (3 x 10 mL), dried over Na_2SO_4, and concentrated under reduced pressure. The residue was purified by column chromatography (SiO_2, pentane/ethyl ether, 3/1) to yield alcohol **1a-1d**.

(*R*)-3-(Furan-2-yl)-3-ethylpropan-1-ol (1a)

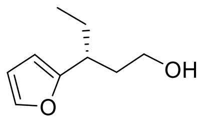

1a

According to the general procedure A, (*R*)-3-(furan-2-yl)-3-ethylpropan-1-ol (**1a,** 53.1 mg, 0.35 mmol, 46%, 89.1% *ee*) was obtained as colorless oil. $[\alpha]_{20}^{D}$: −2.7 (c 0.73, CH_2Cl_2). **R_f** (*n*-hexane/ethyl acetate, 3/1): 0.30. **^{1}H-NMR** (400 MHz, $CDCl_3$): δ [ppm] = 7.30 (dd, *J* = 1.8 Hz, *J* = 0.8 Hz, 1H), 6.27 (dd, *J* = 3.1 Hz, *J* = 1.9 Hz, 1H), 6.01 (d, *J* = 3.1 Hz, 1H), 3.57-3.59(m, 1H), 3.48-3.54 (m, 1H), 2.73-2.80 (m, 1H), 1.78-1.91 (m, 2H), 1.58-1.69 (m, 2H), 0.83 (t, *J* = 7.4 Hz, 3H). **^{13}C-NMR** (400 MHz, $CDCl_3$): δ [ppm]= 158.2, 140.9, 109.9, 105.3, 61.1, 37.3, 36.9, 27.3, 11.8. **HPLC** (column AS, 0.5 mL·min^{-1}, *n*-hexane/2-propanol, 97/3): t_R ((*R*)-**1a**) = 19.1 min (major), t_R ((*S*)-**1a**) = 22.3 min (minor).

(*R*)-3-(Furan-2-yl)-3-propylpropan-1-ol (1b)

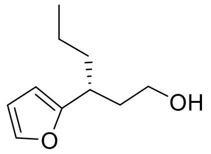

1b

According to the general procedure A, (*R*)-3-(furan-2-yl)-3-propylpropan-1-ol **(1b)** (51.7 mg, 0.31 mmol, 41%, 89.4% *ee*) was obtained as colorless oil. $[\alpha]_{20}^{D}$: –6.2 (c 0.81, CH_2Cl_2). {lit. (*R*)-**1b** $[\alpha]_{20}^{D}$: –6.5 (c 1.16, CH_2Cl_2)}.[22] $\mathbf{R}_f$ (*n*-hexane/ethyl acetate, 3/1): 0.33. **^{1}H-NMR** (400 MHz, $CDCl_3$): δ [ppm] = 7.30 (dd, *J* = 1.8 Hz, *J* = 0.7 Hz, 1H), 6.27 (dd, *J* = 3.1 Hz, *J* = 1.8 Hz, 1H), 6.00 (d, *J* = 3.1 Hz, 1H), 3.57-3.63(m, 1H), 3.48-3.54 (m, 1H), 2.83-2.90 (m, 1H), 1.78-1.91 (m, 2H), 1.50-1.68 (m, 2H), 1.35 (bs, 1H), 1.19-1.35 (m, 2H), 0.87 (t, *J* = 7.4 Hz, 3H). **^{13}C-NMR** (400 MHz, $CDCl_3$): δ [ppm]= 158.4, 141.0, 110.0, 105.2, 61.1, 37.3, 36.7, 35.5, 20.5, 14.1. **HPLC** (column AS, 0.5 mL·min^{-1}, n-hexane/2-propanol 97/3): t_R ((*R*)-**1b**) = 23.1 min (major), t_R ((*S*)-**1b**) = 25.3 min (minor).

(*R*)-3-(Furan-2-yl)-3-isopropylpropan-1-ol (1c)

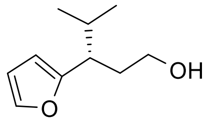

1c

According to the general procedure A, (*R*)-3-(furan-2-yl)-3-isopropylpropan-1-ol (**1c**, 41.6 mg, 0.15 mmol, 33%, 50% *ee*) was obtained as colorless oil. $[\alpha]_{20}^{D}$: -2.2 (c 0.33, CH_2Cl_2). $\mathbf{R}_f$ (*n*-hexane/ethyl acetate, 3/1): 0.33. **^{1}H-NMR** (400 MHz, $CDCl_3$): δ [ppm] = 7.31 (dd, *J* = 1.8 Hz, *J* = 0.8 Hz, 1H), 6.27 (dd, *J* = 3.1 Hz, *J* = 1.8 Hz, 1H), 6.00 (d, *J* = 3.1 Hz, 1H), 3.54-3.60(m, 1H), 3.42-3.48 (m, 1H), 2.61-2.67 (m, 1H), 1.81-1.95 (m, 3H), 1.40 (bs, 1H), 0.92 (d, *J* = 6.7 Hz, 3H), 0.81 (d, *J* = 6.7 Hz, 3H). **^{13}C-NMR** (400 MHz, $CDCl_3$): δ [ppm]= 157.3, 140.9, 109.9, 106.2, 61.5, 42.4, 34.1, 32.2, 20.8, 19.9. **HPLC** (column AS, 0.5 mL·min^{-1}, *n*-hexane/2-propanol 97/3): t_R ((*R*)-**1c**) = 20.4 min (major), t_R ((*S*)-**1c**) = 26.4 min (minor).

(*R*)-3-(Furan-2-yl)-3-cyclopropylpropan-1-ol (1d)

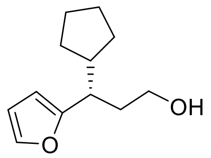

1d

According to the general procedure A, (*R*)-3-(furan-2-yl)-3-cyclopentylpropan-1-ol (**1d**, 33.5 mg, 0.17 mmol, 23%, 48% *ee*) was obtained as colorless oil. $[\alpha]_{20}^{D}$: –2.4 (c 0.14, CH_2Cl_2). $\mathbf{R}_f$ (*n*-hexane/ethyl acetate, 3/1): 0.33. **^{1}H-NMR** (400 MHz, $CDCl_3$): δ [ppm] = 7.31 (dd, *J* = 1.8 Hz, *J* = 0.7 Hz, 1H), 6.27 (dd, *J* = 3.1 Hz, *J* = 1.8 Hz, 1H), 6.00 (dd, *J* = 3.1 Hz, *J* = 0.6 Hz, 1H), 3.53-3.59(m, 1H), 3.42-3.48 (m, 1H), 2.62-2.68 (m, 1H), 2.01-2.12 (m, 1H), 1.92-2.00 (m, 1H),1.80-1.88(m, 2H), 1.42-1.63 (m, 5H), 1.09-1.26 (m, 3H). **^{13}C-NMR** (400 MHz, $CDCl_3$): δ [ppm]= 158.1, 140.9, 109.9, 105.7, 61.4, 44.5, 41.3, 36.2, 31.3, 31.0, 25.4, 25.1. **HPLC** (column AS, 0.5 mL·min^{-1}, *n*-hexane/2-propanol 97/3): t_R ((*R*)-**1d**) = 21.3 min (major), t_R ((*S*)-**1d**) = 25.5 min (minor).

General procedure B for preparation of 1e, 1f and 1g

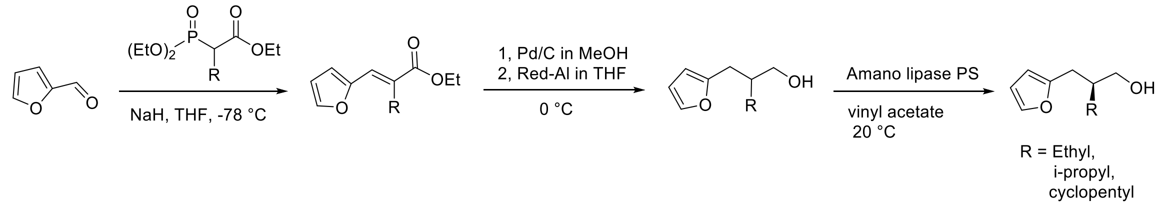

To a solution of substituted triethyl 2-phosphonoacetate (15.6 mmol, 1.5 equiv.) in THF (20 mL), was added sodium hydride (60% in mineral oil, 15.6 mmol, 1.5 equiv.) slowly at 0 °C for 1h. The reaction was cooled to –78 °C and a solution of aldehyde (1g, 10.4 mmol, 1 equiv.) in THF was added dropwise. The mixture was stirred for another 2h at the same temperature. The reaction was quenched with aqueous saturated NH_4Cl solution, extracted with diethyl ether (3 x 15 mL). The combined organic phases were washed with brine, dried over anhydrous Na_2SO_4, and concentrated in vacuo. The residue was purified by column chromatography (SiO_2, pentane/diethyl ether, 50/1) to give corresponding trans-acrylate.

To a solution of acrylate (500 mg) in methanol (20 mL) was added Palladium on charchoal (10 wt. %, 50 mg) at 0 °C with a balloon of hydrogen, the reaction was stirred for 1.5 h. The reaction was filtered through a pad of celite, then concentrated under reduced pressure to give ethyl 3-(furan-2-yl)propanoate, which was used in the next step without purification. To a solution of ethyl 3-(furan-2-yl)propanoate in anhydrous THF was added Red-Al (1.5 equiv.) at 0 °C under argon, and the reaction mixture was stirred for 1h. The reaction was then quenched with saturated aqueous NH_4Cl solution, extracted with diethyl ether (3 x 10 mL), dried over Na_2SO_4, and concentrated under reduced pressure. The residue was purified by column chromatography (SiO_2, pentane/ethyl ether, 3/1) to yield racemic 3-(furan-2-yl) propanol as colorless liquid.

According to a literature procedure,[23] to a solution of *rac*-alcohol (0.3 mmol) and vinyl acetate (0.3 mmol) in dry toluene (4 mL) was added Amano lipase PS (5 mg), the reaction was stirred at 20 °C, and the reaction progress was monitored by HPLC. The reaction mixture was filtered through a pad of Celite, concentrated under reduced pressure. The residue was purified by column chromatography (SiO_2, pentane/ethyl acetate, 5/1) to yield **1e**, **1f** and **1g** as colorless liquid

(*S*)-3-(Furan-2-yl)-2-ethylpropan-1-ol (1e)

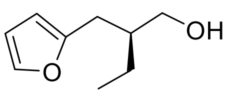

1e

According to the general procedure B, ethyl (*E*)-3-(furan-2-yl)-2-ethylacrylate (1.82g, 9.37 mmol, 90%) was obtained as colorless oil. **^{1}H-NMR** (400 MHz, $CDCl_3$): δ [ppm] = 7.50 (d, *J* = 1.7 Hz, 1H), 7.36 (s, 1H), 6.58 (d, *J* = 3.4 Hz, 1H), 6.46 (dd, *J* = 3.4 Hz, *J* = 1.8 Hz, 1H), 4.23 (q, *J* = 7.1 Hz, 2H), 2.71 (q, *J* = 7.4 Hz, 2H), 1.12 (t, *J* = 7.4 Hz, 3H), 0.86 (t, *J* = 6.9 Hz, 3H). **^{13}C-NMR** (400 MHz, $CDCl_3$): δ [ppm] = 168.3, 151.7, 144.1, 131.5, 124.9, 114.6, 112.0, 60.7, 14.3, 14.1, 13.5.

Ethyl 3-(furan-2-yl)-2-ethylpropanoate was obtained as colorless oil. **^{1}H-NMR** (400 MHz, $CDCl_3$): δ [ppm] = 7.28 (dd, *J* = 1.8 Hz, *J* = 0.8 Hz, 1H), 6.25 (dd, *J* = 3.1 Hz, *J* = 1.8 Hz, 1H), 6.00 (dd, *J* = 3.1 Hz, *J* = 0.7 Hz, 1H), 4.11 (q, *J* = 7.1 Hz, 1H), 2.95 (dd, *J* = 15.0 Hz, *J* = 8.3 Hz, 1H), 2.77 (dd, *J* = 15.0 Hz, *J* = 6.3 Hz, 1H), 2.61-2.69 (m, 1H), 1.53-1.69 (m, 2H), 1.21 (t, *J* = 7.1 Hz, 3H), 0.91 (t, *J* = 7.4 Hz, 3H). **^{13}C-NMR** (400 MHz, $CDCl_3$): δ [ppm] = 175.3, 153.5, 141.3, 110.2, 106.2, 60.3, 46.3, 30.3, 25.1, 14.3, 11.6.

(*R*)-3-(furan-2-yl)-2-ethylpropan-1-ol **(1e)** (15.2 mg, 0.1 mmol, 33%, >99% *ee*) was obtained as colorless oil. $[\alpha]_{20}^{D}$: –4.3 (c 0.24, CH_2Cl_2). **R*f*** (*n*-hexane/ethyl acetate, 3/1): 0.33. **^{1}H-NMR** (400 MHz, $CDCl_3$): δ [ppm] = 7.30 (dd, *J* = 1.8 Hz, *J* = 0.8 Hz, 1H), 6.28 (dd, *J* = 3.1 Hz, *J* = 1.8 Hz, 1H), 6.02 (dd, *J* = 3.2 Hz, *J* = 0.7 Hz, 1H), 3.50-3.58 (m, 2H), 2.68 (d, *J* = 6.7 Hz, 1H), 1.76-1.82 (m, 1H), 1.50 (bs, 1H), 1.29-1.44 (m, 2H), 0.94 (t, *J* = 7.4

Hz, 3H). **^{13}C-NMR** (400 MHz, $CDCl_3$): δ [ppm] = 154.8, 141.1, 110.2, 106.2, 64.8, 42.0, 29.3, 23.5, 11.4. **HPLC** (column AS, 0.5 mL·min^{-1}, *n*-hexane/2-propanol 97/3): t_R (minor) = 17.7 min, t_R (major) = 21.3 min.

(*R*)-3-(Furan-2-yl)-2-isopropylpropan-1-ol (1f)

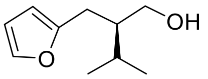

1f

To a solution of ethyl 2-(diethoxyphosphoryl) acetate (11.2 g, 50 mmol) in 40 mL DMSO, Potassium tert-butoxide (6.16g, 55 mmol) was added at 0 °C and the mixture was stirred for 30 min. After potassium tert-butoxide dissolved, 2-iodopropane (9.35 g, 55 mmol) was added, and then the mixture was stirred at 60 °C until reaction complete by TLC. The reaction was quenched with aqueous saturated NH_4Cl solution, extracted with diethyl ether three times. The combined organic phases were washed with brine, dried over Na_2SO_4, and concentrated under reduced pressure. The residue was purified by column chromatography (SiO_2, n-hexane/ethyl acetate, 1/1) to give pure ethyl 2-isopropyl-2-(diethoxyphosphoryl)acetate (12.2 g, 46 mmol, 92%) as a colourless oil. **^{1}H-NMR** (400 MHz, $CDCl_3$): δ [ppm] = 4.07-4.20 (m, 6H), 2.69 (dd, *J* = 20.2, *J* = 9.3, 1H), 2.30-2.38 (m, 1H), 1.24-1.31 (m, 9H), 1.11 (d, *J* = 6.7, 1H), 0.98 (d, *J* = 6.7, 1H). **^{13}C-NMR** (400 MHz, $CDCl_3$): δ [ppm] = 169.4, 62.5, 61.2, 54.2, 52.8, 28.4, 21.7, 21.6, 16.4, 14.2.

According to the general procedure B, ethyl (*E*)-3-(furan-2-yl)-2-isopropylacrylate (1.67g, 8.01 mmol, 77%) was obtained as a colourless oil. **^{1}H-NMR** (400 MHz, $CDCl_3$): δ [ppm] = 7.32 (dd, *J* = 1.8, *J* = 0.6, 1H), 6.42 (d, *J* = 3.4, 1H), 6.35 (dd, *J* = 3.4, *J* = 1.8, 1H), 6.24 (d, *J* = 1.2, 1H), 4.30 (q, *J* = 7.1, 2H), 2.68-2.75 (m, 1H), 1.31 (t, *J* = 7.0, 3H), 1.15 (d, *J* = 6.8, 6H). **^{13}C-NMR** (400 MHz, $CDCl_3$): δ [ppm] = 169.9, 150.9, 142.5, 138.3, 116.2, 111.5, 110.4, 60.8, 33.0, 20.5, 14.3.

Ethyl 3-(furan-2-yl)-2-isopropylpropanoate was obtained as colorless oil. **^{1}H-NMR** (400 MHz, $CDCl_3$): δ [ppm] = 7.27 (dd, *J* = 1.7 Hz, *J* = 0.7 Hz, 1H), 6.23 (dd, *J* = 2.9 Hz, *J* = 1.9 Hz, 1H), 5.98 (d, *J* = 3.1 Hz, 1H), 4.08 (q, *J* = 7.1 Hz, 1H), 2.93 (dd, *J* = 15.0 Hz, *J* = 10.2 Hz, 1H), 2.81 (dd, *J* = 15.0 Hz, *J* = 4.7 Hz, 1H), 2.51-2.56 (m, 1H), 2.11-2.23 (m, 1H), 1.18 (t, *J* = 7.1 Hz, 3H), 0.99 (d, *J* = 6.8 Hz, 3H), 0.95 (d, *J* = 6.7 Hz, 3H). **^{13}C-NMR** (400 MHz, $CDCl_3$): δ [ppm] = 174.7, 153.9, 141.2, 110.2, 105.9, 60.1, 51.5, 30.5, 28.3, 20.3, 20.1, 14.3.

3-(furan-2-yl)-2-isopropylpropan-1-ol **(1f)** (23.2 mg, 0.14 mmol, 46%, 99% *ee*) was obtained as colorless oil. $[\alpha]_{20}^{D}$: -2.3 (c 0.27, CH2Cl2). **R*f*** (*n*-hexane/ethyl acetate, 3/1): 0.36. **^{1}H-NMR** (400 MHz, $CDCl_3$): δ [ppm] = 7.30 (dd, *J* = 1.8, *J* = 0.8, 1H), 6.28 (dd, *J* = 3.1, *J* = 1.8, 1H), 6.02 (dd, *J* = 3.1, *J* = 0.7, 1H), 3.54-3.72 (m, 2H), 2.73 (dd, *J* = 15.2, *J* = 5.3, 1H), 2.73 (dd, *J* = 15.2, *J* = 8.3, 1H), 1.69-1.84 (m, 2H), 1.46 (bs, 1H), 0.94 (dd, *J* = 6.7, *J* = 5.8, 6H). **^{13}C-NMR** (400 MHz, $CDCl_3$): δ [ppm] = 155.2, 141.0, 110.3, 106.1, 63.5, 46.4, 28.1, 26.9, 19.9, 19.6. **HPLC** (column AS, 0.5 mL·min^{-1}, n-hexane/2-propanol 97/3): t_R (minor) = 16.1 min, t_R (major) = 22.9 min.

(*R*)-3-(furan-2-yl)-2-cyclopentanylpropan-1-ol (1g)

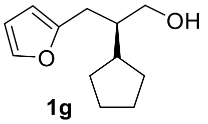

1g

In analogy to the synthesis of ethyl 2-isopropyl-2-(diethoxyphosphoryl)acetate starting from 2-iodopropane replaced by iodocyclopentane (10.8 g, 55 mmol), ethyl 2-cyclopentyl-2-(diethoxyphosphoryl)acetate (13.14 g, 45 mmol, 90%) was obtained as a colorless oil. **^{1}H-NMR** (400 MHz, $CDCl_3$): δ [ppm] = 4.08-4.16 (m, 6H), 2.77 (dd, *J* = 20.0, *J* = 10.6, 1H), 2.38-2.42 (m, 1H), 1.94-2.00 (m, 1H), 1.76-1.81 (m, 1H), 1.49-1.67 (m, 4H), 1.24-1.34 (m, 10H), 1.16-1.21 (m, 1H). **^{13}C-NMR** (400 MHz, $CDCl_3$): δ [ppm] = 169.5, 62.5, 61.2, 52.1, 50.8, 39.0, 32.0, 31.5, 25.1, 24.3, 16.5, 14.2.

According to the general procedure B, ethyl (*E*)-3-(furan-2-yl)-2-cyclopentylacrylate (1.73 g, 7.39 mmol, 71%) was obtained as colorless oil. **^{1}H-NMR** (400 MHz, $CDCl_3$): δ [ppm] = 7.32 (dd, *J* = 1.7, *J* = 0.5, 1H), 6.40 (d, *J* = 3.4, 1H), 6.35 (dd, *J* = 3.4, *J* = 1.8, 1H), 6.26 (d, *J* = 1.4, 1H), 4.29 (q, *J* = 7.1, 2H), 2.74-2.82 (m, 1H), 1.85-1.91 (m, 2H), 1.68-1.74 (m, 2H), 1.51-1.63 (m, 4H), 1.31 (t, *J* = 7.1, 3H). **^{13}C-NMR** (400 MHz, $CDCl_3$): δ [ppm] = 170.1, 151.0, 142.5, 135.9, 116.4, 111.5, 110.3, 60.8, 34.2, 31.5, 24.9, 22.4, 14.1.

Ethyl 3-(furan-2-yl)-2-cyclopentylpropanoate was obtained as colorless oil. **^{1}H-NMR** (400 MHz, $CDCl_3$): δ [ppm] = 7.27 (s, 1H), 6.23 (dd, *J* = 3.1 Hz, *J* = 1.5 Hz, 1H), 5.98 (d, *J* = 3.1 Hz, 1H), 4.07 (q, *J* = 7.3 Hz, 2H), 2.82-2.98 (m, 2H), 2.51-2.57 (m, 1H), 1.99-2.06 (m,1H), 1.80-1.88 (m, 3H), 1.48-1.73 (m, 5H), 1.17 (t, *J* = 7.3 Hz, 3H). **^{13}C-NMR** (400 MHz, $CDCl_3$): δ [ppm] = 175.1, 153.7, 141.2, 110.2, 105.9, 60.2, 50.5, 42.6, 30.8, 30.6, 30.2, 25.2, 25.1, 14.3.

3-(furan-2-yl)-2-cyclopentanylpropan-1-ol (**1g**) (27.9 mg, 0.14 mmol, 48%, >99% *ee*) was obtained as colorless oil. $[\alpha]_{20}^{D}$: −16.5 (c 0.21, CH_2Cl_2). **R_f** (*n*-hexane/ethyl acetate, 3/1): 0.36. **^{1}H-NMR** (400 MHz, $CDCl_3$): δ [ppm] = 7.30 (dd, *J* = 1.8 Hz, *J* = 0.8 Hz, 1H), 6.28 (dd, *J* = 3.1 Hz, *J* = 1.9 Hz, 1H), 6.03 (dd, *J* = 3.1 Hz, *J* = 0.6 Hz, 1H), 3.51-3.61 (m, 2H), 2.83 (dd, *J* = 15.1 Hz, *J* = 7.9 Hz, 1H), 1.48-1.87 (m, 10H), 1.11-1.27 (m, 1H). **^{13}C-NMR** (400 MHz, $CDCl_3$): δ [ppm] = 154.9, 141.0, 110.3, 106.3, 64.2, 46.0, 41.9, 30.9, 30.8, 28.5, 25.3, 25.1. **HPLC** (column AS, 0.5 mL·min^{-1}, n-hexane/2-propanol 97/3): t_R (major) = 17.8 min, t_R (minor) = 19.4 min.

Preparation of 1h

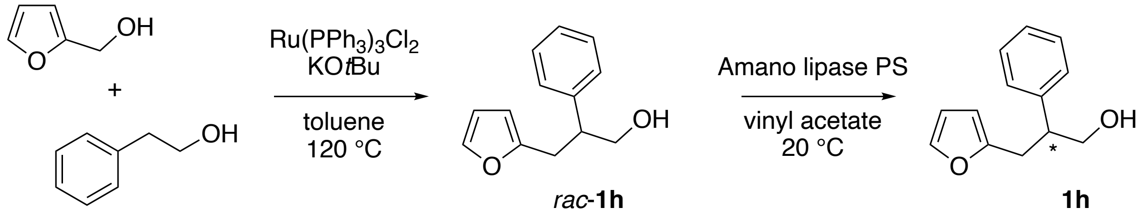

According to a literature procedure,[24] to a solution of $Ru(PPh_3)_3Cl_2$ (191.8 mg, 0.2 mmol) and KO*t*Bu (960 mg, 8.5 mmol) in toluene (50 mL) was added furfuryl alcohol (980 mg, 10 mmol) and 2-phenylethanol (3.66 g, 30 mmol) and the mixture was heated to reflux. After 24h, the mixture was cooled to room temperature, quenched with H_2O (20 mL), extracted by ethyl acetate (3 × 15 mL). The combined organic phases were dried over anhydrous Na_2SO_4 and concentrated *in vacuo*. The residue was purified by flash column chromatography (SiO_2, heptane/ethyl acetate, 2/1) to afford pure alcohol *rac*-**1h** (1.15 g, 5.7 mmol, 57%) as a yellow oil.

To a solution of *rac*-**1h** (0.3 mmol) and vinyl acetate (0.3 mmol) in dry toluene (4 mL) was added Amano lipase PS (5 mg), the reaction was stirred at 20 °C, which was monitored by HPLC. The reaction mixture was filtered through a pad of Celite, concentrated under reduced pressure. The residue was purified by column chromatography (SiO_2, pentane:ethyl ether, 5:1) to yield enantioenriched **1h** (39.4 mg, 0.19 mmol, 65%, 17% *ee*) as colorless liquid. $[\alpha]_{20}^{D}$: −7.8 (c 0.4, CH_2Cl_2). **R_f** (*n*-hexane/ethyl acetate, 3/1): 0.3. **^{1}H-NMR** (400 MHz, $CDCl_3$): δ [ppm] = 7.25-7.36 (m, 4H), 7.21-24 (m, 2H), 6.22 (dd, *J* = 3.1 Hz, *J* = 1.8 Hz, 1H), 5.91 (dd, *J* = 3.1 Hz, *J* = 0.8 Hz, 1H), 3.78-3.82 (m, 2H), 3.21 (p, *J* = 6.5 Hz, 1H), 3.07 (dd, *J* = 7.5 Hz, *J* = 15.1 Hz, 1H), 2.95 (dd, *J* = 7.4 Hz, *J* = 15.0 Hz, 1H), 1.43 (br, 1H). **^{13}C-NMR** (400 MHz, $CDCl_3$): δ [ppm] = 153.9, 141.7, 141.2, 128.8, 128.0, 127.1, 110.3, 106.5, 66.6, 47.4, 30.9. **HPLC** (column AS, 0.5 mL·min^{-1}, n-hexane/2-propanol 97/3): t_R (major) = 34.4 min, t_R (minor) = 37.4 min.

General procedure C for preparation of 1i, 1j and 1k

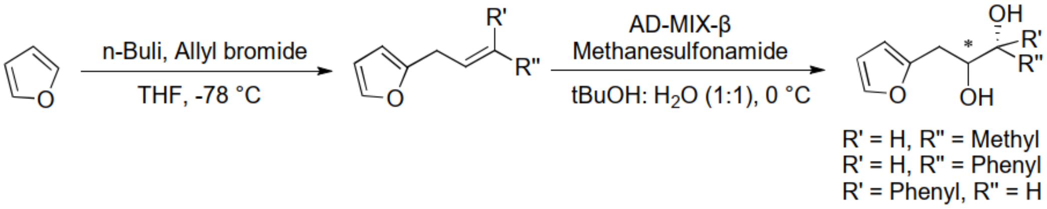

To a solution of furan (3 equiv) in anhydrous THF was added a solution of n-BuLi (2.0 equiv, 2.0 M in hexane). The solution was stirred under argon at –78 °C for 4h. A solution of allyl bromide (1 equiv) in anhydrous THF was added dropwise and further stirring for 10h at the same temperature. The reaction mixture was quenched with saturated aqueous NH_4Cl solution, extracted with diethyl ether three times. The combined organic phases dried over anhydrous Na_2SO_4 and concentrated under reduced pressure. The residual was purified by flash column chromatography (SiO_2, *n*-pentane) to give pure allylfuran.

To a solution of allylfuran (1 equiv) in *tert*-butanol and H_2O (1/1 v/v) was added AD-mix-β (1 equiv) and methanesulfonamide (1 equiv) at 0 °C. The reaction mixture was stirred for 24h at the same temperature until complete consumption of substrate was indicated by TLC. The mixture was stirred for 30 min after addition of solid Na_2SO_3 (10 equiv), and then extracted with ethyl acetate three times. The combined organic phases were dried over anhydrous Na_2SO_4 and concentrated under reduced pressure. The residual was purified by flash column chromatography (SiO_2, heptane/ethyl acetate, 2/1) to give alcohols **1i, 1j, 1k.**

(2*R*,3*R*)-1-(Furan-2-yl)butane-2,3-diol (1i)

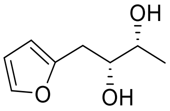

1i

According to the general procedure C from (*E*)-crotyl bromide (940 mg, 7 mmol), *E*-2-crotonylfuran (597 mg, 4.9 mmol, 70 %) was obtained as colorless oil. **^{1}H-NMR** (400 MHz, $CDCl_3$): δ [ppm] =7.31 (dd, *J* = 1.8 Hz, *J* = 0.7 Hz, 1H), 6.28 (dd, *J* = 3.1 Hz, *J* = 1.8 Hz, 1H), 5.98-5.99 (m, 1H), 5.51-5.65 (m, 2H), 3.63-3.76 (m, 2H), 3.31-3.40 (m, 2H), 1.68 (m, 3H). **^{13}C-NMR** (400 MHz, $CDCl_3$): δ [ppm] = 152.2, 141.8, 110.5, 107.4, 74.5, 70.1, 32.6, 19.4.

(2*R*,3*R*)-1-(furan-2-yl)butane-2,3-diol (**1i**) (151.3 mg, 0.97 mmol, 65%, 75% *ee*) was prepared as colorless oil. $[\alpha]_{20}^{D}$: 21.4 (c 0.35, CH_2Cl_2). **R_f** (heptane/ethyl acetate 1/1): 0.2. **^{1}H-NMR** (400 MHz, $CDCl_3$): δ [ppm] = 7.34 (dd, *J* = 1.8 Hz, *J* = 0.8 Hz, 1H), 6.31 (dd, *J* = 3.1 Hz, *J* = 1.9 Hz, 1H), 6.13 (dd, *J* = 3.2 Hz, *J* = 0.8 Hz, 1H), 3.61-3.88 (m, 2H), 2.76-2.92 (m, 2H), 2.36 (br, 2H), 1.23 (d, *J* = 6.0 Hz, 3H). **^{13}C-NMR** (400 MHz, $CDCl_3$): δ [ppm] = 152.3, 141.8, 110.5, 107.4, 74.5, 70.1, 32.6, 19.5. **HPLC** (column OD-H, 0.5 mL·min^{-1}, *n*-hexane/2-propanol 90/10): t_R ((1*S*,2*S*)-**1j**) = 16.4 min, t_R ((1*R*,2*R*)-**1j**) = 17.9 min.

(1*R*,2*R*)-3-(Furan-2-yl)-1-phenylpropane-1,2-diol (1j)

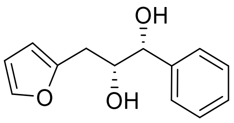

1j

According to the general procedure C starting from *E*-cinnamyl bromide (871 mg, 4.4 mmol), cinnamyl furan (597 mg, 3.2 mmol, 73%) was obtained as colorless oil. **^{1}H-NMR** (400 MHz, $CDCl_3$): δ [ppm] = 7.20-

7.38 (m, 6H), 6.49 (d, J = 15.8 Hz, 1H), 6.28-6.35 (m, 2H), 6.07 (d, J = 3.1 Hz, 1H), 3.56 (d, J = 6.7 Hz, 2H). **^{13}C-NMR** (400 MHz, $CDCl_3$): δ [ppm] = 154.0, 141.4, 137.3, 132.1, 128.6, 127.4, 126.3, 125.7, 110.4, 105.7, 31.8.

(1*R*,2*R*)-3-(furan-2-yl)-1-phenylpropane-1,2-diol (**1j**) (152 mg, 0.69 mmol, 65%, >99% *ee*) was prepared as colorless oil. $[\alpha]_{20}^{D}$: –2.5 (c 0.41, CH_2Cl_2). **R_f**(heptane/ethyl acetate 1/1): 0.30. **^{1}H-NMR** (400 MHz, $CDCl_3$): δ [ppm] = 7.30-7.40 (m, 6H), 6.30 (dd, *J* = 3.2 Hz, *J* = 1.9 Hz, 1H), 6.10 (dd, *J* = 3.1 Hz, *J* = 0.7 Hz, 1 H), 4.54 (dd, *J* = 6.4 Hz, *J* = 3.6 Hz, 1H), 3.99-4.05 (m, 1H), 2.70-2.81 (m, 3H), 2.47 (br, 1H). **^{13}C-NMR** (400 MHz, $CDCl_3$): δ [ppm] = 152.2, 141.6, 140.7, 128.6, 128.2, 126.9, 110.4, 107.3, 76.7, 74.4, 31.8. **HPLC** (column OD-H, 0.5 mL·min^{-1}, *n*-hexane/2-propanol 90/10): t_R ((1*S*,2*S*)-**1j**) = 23.7 min, t_R ((1*R*,2*R*)-**1j**) = 24.9 min.

(1*R*,2*S*)-3-(Furan-2-yl)-1-phenylpropane-1,2-diol (1k)

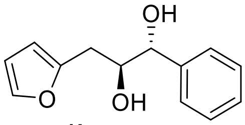

1k

To a solution of 3-phenylprop-2-yn-1-ol (2.0 g, 15.1 mmol) and quinoline (4 mL) in dry ethyl acetate (20 mL) was added poisoned Lindlar's catalyst (400 mg). Under a H_2 atmosphere (balloon), the reaction was stirred for 24h at room temperature. The reaction mixture was filtered through a pad of celite, diluted with ethyl acetate (50 mL), washed with 2% H_2SO_4 (3 × 15 mL) and brine (3 × 15 mL), dried over anhydrous Na_2SO_4 and concentrated under reduced pressure. The residue was purified by flash column chromatography (SiO_2, heptane/ethyl acetate, 3/1) to afford the pure (*Z*)-cinnamyl alcohol (1.76 g, 13.1 mmol, 87%, >99% *Z*/*E*) as pale yellow oil. **^{1}H-NMR** (400 MHz, $CDCl_3$): *δ* [ppm] = 7.20-7.37 (m, 5 H), 6.57 (d, *J* = 11.8 Hz, 1 H), 5.87 (td, *J* = 11.7 Hz, *J* = 6.5 Hz, 1 H), 4.45 (d, *J* = 6.4 Hz, 2 H), 1.54 (br, 1H) ppm. **^{13}C-NMR** (400 MHz, $CDCl_3$): *δ* [ppm] = 136.4, 131.2, 128.9, 128.4, 127.4, 59.8.

To a solution of *N*-bromosuccinimide (NBS) (1.5 g, 3.6 mmol, 1.5 equiv.) in dichloromethane (40 mL) was added Me_2S (1.6 mL, 22.4 mmol, 2.0 equiv.) dropwise at –20 °C, then followed by addition of a solution of (*Z*)-cinnamyl alcohol (1.5 g, 11.2 mmol) in dichloromethane (2 mL). The mixture was stirred for 2h at the same temperature. The reaction was quenched with cold water (10 mL), diluted with diethyl ether (80 mL), and washed with brine (3 × 20 mL). The organic phase was dried over anhydrous Na_2SO_4 and concentrated under reduced pressure. The residue was purified by flash column chromatography (SiO_2, pentane) to afford pure (*Z*)-cinnamyl bromide (1.27 g, 6.5 mmol, 58%, >99% *Z*/*E*) as colorless oil. **^{1}H-NMR** (400 MHz, $CDCl_3$): *δ* [ppm] = 7.27-7.41 (m, 6 H), 6.61 (d, *J* = 11.3 Hz, 1 H), 5.99 (td, *J* = 11.2 Hz, *J* = 8.6 Hz, 1 H), 4.17 (dd, *J* = 8.7 Hz, *J* = 0.7 Hz, 2 H). **^{13}C-NMR** (400 MHz, $CDCl_3$): *δ* [ppm] = 235.7, 133.6, 128.8, 128.7, 127.8, 127.1, 126.9, 29.0.

According to the general procedure C starting from (*Z*)-cinnamyl bromide (1 g, 5.1 mmol), *Z*-2-cinnamyl furan (638 mg, 3.5 mmol, 68%) was obtained as colorless oil. **^{1}H-NMR** (400 MHz, $CDCl_3$): δ [ppm] = 7.20-7.41 (m, 6H), 6.61 (d, *J* = 11.5 Hz, 1H), 6.31 (dd, *J* = 3.1 Hz, *J* = 1.9 Hz, 1H), 6.08 (dd, *J* = 3.1 Hz, *J* = 0.9 Hz, 1H), 5.83-5.90 (td, , *J* = 11.4 Hz, *J* = 7.5 Hz, 1H), 3.65 (d, *J* = 7.5 Hz, 2H). **^{13}C-NMR** (400 MHz, $CDCl_3$): δ [ppm] = 154.3, 141.4, 136.9, 131.0, 128.8, 128.4, 127.1, 127.0, 110.4, 105.4, 27.8.

(1*R*,2*S*)-3-(furan-2-yl)-1-phenylpropane-1,2-diol (**1k**) (124.5 mg, 57.1 mmol, 51 %, 43% *ee*) was obtained as colorless oil. $[\alpha]_{20}^{D}$: –8.2 (c 0.35, CH_2Cl_2). **R_f** (heptane/ethyl acetate, 1/1): 0.33. **^{1}H-NMR** (400 MHz, $CDCl_3$): δ [ppm] = 7.29-7.41 (m, 6H), 6.29 (dd, *J* = 3.1 Hz, *J* = 1.9 Hz, 1H), 6.08 (dd, *J* = 3.1 Hz, *J* = 0.7 Hz, 1H), 4.79 (d, *J* = 4.4 Hz, 1H), 4.10-4.13 (m, 1H), 2.70-2,81 (m, 2H), 2.61 (brs, 1H), 2.26 (brs, 1H). **^{13}C-NMR** (100 MHz, $CDCl_3$): δ [ppm] = 152.6, 141.7, 140.0, 128.6, 128.0, 126.7, 110.5, 107.3, 76.0, 73.9, 30. **HPLC** (column OD-H, 0.5 mL·min^{-1}, *n*-hexane/2-propanol 90/10): t_R ((1*S*,2*R*)-**1k**) = 25.8 min, t_R ((1*R*,2*S*)-**1k**) = 28.0 min.

(*E*)-4-(3-methylfuran-2-yl)but-3-en-2-one (4)

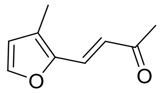

To a solution of methyl 3-methyl-2-furoate 1 (2 g, 14.3 mmol) in anhydrous THF (20 mL) was added bis(2-methoxyethoxy)aluminum hydride (Red-Al) (21.4 mmol, 1.5 equiv., 60 wt.% in toluene) at 0 °C under argon, and the reaction mixture was stirred for 2 h. The reaction was then quenched with saturated aqueous NH_4Cl solution, extracted with diethyl ether (3 x 15 mL), dried over Na_2SO_4, and concentrated under reduced pressure. The residue was purified by flash column chromatography (SiO_2, pentane/ethyl ether, 3/1) to yield 3-methylfurfuryl alcohol (1.32 g, 11.8 mmol, 83%) as a pale yellow liquid. **^{1}H-NMR** (400 MHz, $CDCl_3$): δ [ppm] = 7.30 (d, *J* = 1.8 Hz, 1H), 6.21 (d, *J* = 1.6 Hz, 1H), 4.57 (d, *J* = 5.8 Hz, 2H), 2.05 (s, 3H). **^{13}C-NMR** (400 MHz, $CDCl_3$): δ [ppm] = 149.5, 141.7, 117.8, 113.1, 55.4, 9.8.

To a solution of the alcohol (1.0 g, 8.9 mmol) in CH_2Cl_2 (20 mL) was added MnO_2 (7.7g, 89.2 mmol, 10 equiv. activated 85%) and 1-(triphenylphosphoranylidene)-2-propanone (4.25 g, 12.4 mmol, 1.5 equiv.) at room temperature. After 20 h, the reaction mixture was filtered through a pad of celite, concentrated under reduced pressure and purified by column chromatography (SiO_2, pentane/ethyl ether, 5/1) to afford pure enone **4** (1.0 g, 6.8 mmol, 77%) as pale yellow oil. **^{1}H-NMR** (400 MHz, $CDCl_3$): δ [ppm] = 7.38 (dd, *J* = 1.7 Hz, *J* = 0.4 Hz, 1H), 7.31 (d, *J* = 15.7 Hz, 1H), 6.55 (d, *J* = 15.7 Hz, 1H), 6.32 (d, *J* = 1.5 Hz, 1H), 2.30 (s, 3H), 2.15 (s, 3H). **^{13}C-NMR** (400 MHz, $CDCl_3$): δ [ppm] = 197.9, 147.1, 144.4, 127.3, 127.1, 122.4, 115.1, 28.3, 10.4.

5. Configurational analysis

5.1 Density functional theory

(*S*,*S*,*R*)-**2a**

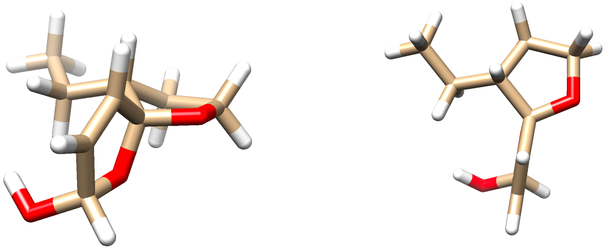

(*R*,*S*,*R*)-**2a**

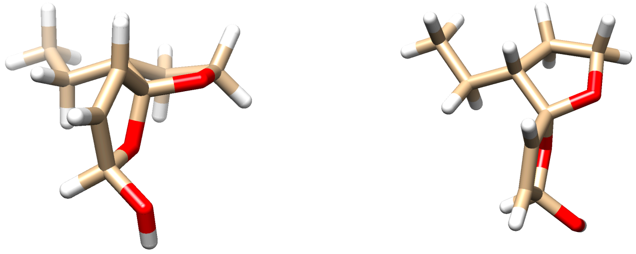

(*S*,*R*,*R*)-**2a**

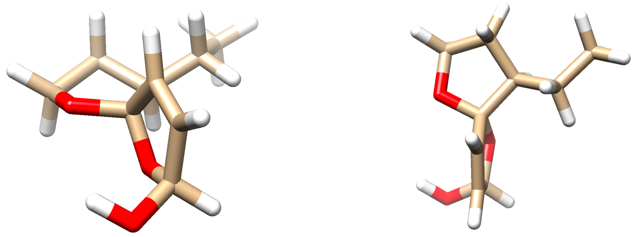

(*R*,*R*,*R*)-**2a**

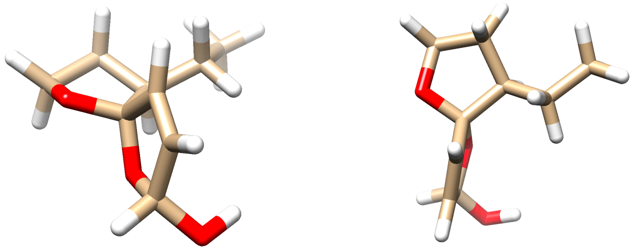

(*S*,*R*)-**3a**

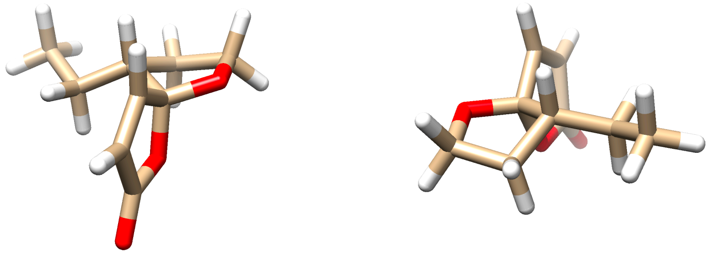

(*R*,*R*)-**3a**

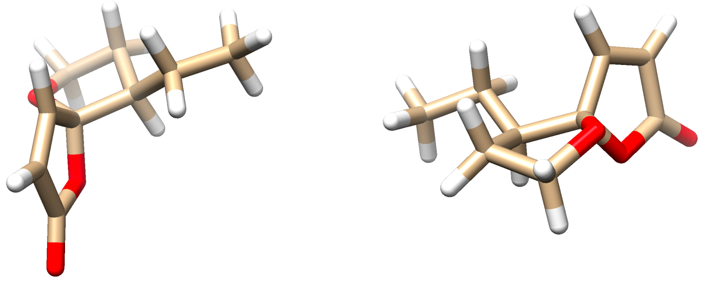

Supplementary Figure 4: Optimized geometries of **2a** and **3a** isomers.

Geometries & Energies:

(*S*,*R*,*R*)-**2a**

O	0.17261526254055	-0.82921600505434	-0.78616618966988
O	-0.69596986867786	-0.20711762391739	1.29617005691730
O	-2.79927834947829	-1.21986805924663	1.20263138460606
C	2.15797760461780	0.36220100806062	-0.65269627979477
C	1.57736445751020	-1.02256104655068	-0.84762086897851
C	1.21614049933637	0.93117021745073	0.39987900265055
C	-0.12609591423912	0.24562046197345	0.08358546095390
C	-1.22549083337056	1.04663973599309	-0.55337484136026
C	1.15240259117128	2.44347117248350	0.49894443118666
C	-2.09478525884294	-0.02026798585655	1.29735760641370
C	-2.34291268412239	0.89542020836592	0.13413426942770
C	2.45492435864016	3.05597969947276	0.98441229977268
H	-2.37743357534393	0.40235542969106	2.26489260096387
H	-2.57394396076453	-1.62001575162599	0.35501698456088
H	2.08948118324837	0.93636272420989	-1.58277911266555
H	3.20108416380208	0.34647874411790	-0.33382462504008
H	1.90326334038269	-1.70476322031018	-0.05139553528728
H	1.80999936931960	-1.47495686815122	-1.81250513697451
H	1.50951785486309	0.53195723124398	1.37754230365171
H	-3.32477050947413	1.28447167780801	-0.09849410725012
H	-1.08833867057901	1.57872712721295	-1.48442637880653
H	0.88992090740702	2.86338548112138	-0.47855161192157
H	0.34007910346255	2.71784874454570	1.17964287260261
H	2.72926448467818	2.66175626231169	1.96672312462471
H	2.37373508876103	4.14128201921856	1.07229830502370
H	3.27880528935178	2.84090831273178	0.29936444559305

Electronic Energy:

-576.121315738476

Correction to Gibbs Free Energy:
0.18701762

(*R*,*R*,*R*)-**2a**

O	-2.48454369328204	0.40610671413844	2.37677635517960
O	0.24613251146342	-0.74312726992006	-0.96574484049005
O	-0.67228188368191	-0.28045307741834	1.12615149465361
C	2.26081576919965	0.34219214407958	-0.61835955304068
C	1.63696236550648	-1.00845869620523	-0.89855402383077
C	1.25308836320469	0.94296992411812	0.35488585443329

C -0.08064882699947 0.26678289513079 -0.03655841570769
C -1.17785337516340 1.09717055417377 -0.63952331028299
C 1.21096443933180 2.45916293135192 0.40254193274928
C -2.07012961949823 -0.07393866045223 1.13905214901793
C -2.31208076818328 0.86659622345811 -0.00461184117313
C 2.49894276786476 3.06652011295720 0.93081614901927
H -2.05592597398366 1.25755592030672 2.51673114605617
H -2.59166517637082 -1.02962414565345 1.01739914578306
H 2.30704130915607 0.93459044127063 -1.53822392257940
H 3.26778955794718 0.27082638294855 -0.20470095277668
H 1.85264772670002 -1.72048153848528 -0.09068301455798
H 1.93692037938797 -1.45454194795763 -1.84738931242195
H 1.47292713360647 0.56990550776640 1.36107632540696
H -3.29349286081079 1.26100721403641 -0.23027374966704
H -1.02294099184475 1.71590352099013 -1.51216854458797
H 1.00590505967284 2.85002211998802 -0.60037929466788
H 0.37134737208546 2.77422809764404 1.03134454477904
H 2.71725260897179 2.70431478852122 1.93912569629029
H 2.43536675088919 4.15573656713176 0.97378250722695
H 3.35049516413056 2.81003635218040 0.29587966088875

Electronic Energy: -576.120076166015

Correction to Gibbs Free Energy: 0.18680629

(*R*,*S*,*R*)-**2a**

O 0.78401009293412 0.92328017043919 -0.29231478227093
O -0.25347444253353 -0.26973232390384 1.41835199616197
O -1.60209012355792 1.23934312448998 2.58559842702072
C 2.10969800983087 0.42627686734615 -0.47021693972226
C 2.03860900008678 -1.07811568728396 -0.25470016060458
C 0.57634028298391 -1.37447789518774 -0.56433263091224
C -0.09242425785912 -0.12178025853918 0.01150148106725
C -1.47000370456212 0.19818076190613 -0.47586107285241
C -1.55906033237490 0.07412241436844 1.81797635346538
C -2.30415586763099 0.30140467613788 0.54183022849865
C 0.03203821956752 -2.69075152819388 -0.05040027701096
C 0.65704441506808 -3.89641148722003 -0.73204774124494
H -1.13080502431350 1.07209490368333 3.40671205517949
H -1.95004780301613 -0.77274548915892 2.40035283045787
H 2.43313334660071 0.67534921472353 -1.48638867777179
H 2.77536049266542 0.92840447598104 0.23599073877684

H	2.24868352033678	-1.33504550697830	0.78709526809040
H	2.73710255974343	-1.62188842093677	-0.89124961030434
H	-3.35421755634255	0.55599627808724	0.51886379544625
H	-1.68416661356739	0.34777189189742	-1.52499148396991
H	0.43283677807636	-1.32449206102128	-1.65198540399778
H	0.19499846519623	-2.74007045967617	1.02935034185116
H	-1.05337744955597	-2.70561016902531	-0.19879765019752
H	1.73497506009316	-3.94004323256088	-0.55764889267738
H	0.49845306342196	-3.86694323709948	-1.81383875589264
H	0.22623035660874	-4.82814669597459	-0.35960715158633

Electronic Energy: -576.121815425914

Correction to Gibbs Free Energy: 0.18642679

(*S*,*S*,*R*)-**2a**

O	-2.06751128397825	-0.92409815957553	2.48015451710989
O	0.83642039954182	0.95309349666995	-0.40399575935735
O	-0.20061473369691	-0.14992127472241	1.34503647484728
C	2.15821587626274	0.43427445811831	-0.54914377334665
C	2.06441094629748	-1.06429511218449	-0.29994903550410
C	0.59866726077340	-1.34883381762955	-0.60492306010573
C	-0.05288786134331	-0.07174415265086	-0.06411986223748
C	-1.42770741318714	0.25461216663493	-0.55854705570095
C	-1.52304797133869	0.12963239776668	1.74597179346081
C	-2.25142414333419	0.40257919690599	0.46288306088995
C	0.03715827979899	-2.64063848715612	-0.04844310963650
C	0.65875146850750	-3.87783488172655	-0.67384077577744
H	-2.11613442466524	-1.69073159846164	1.89843787298526
H	-1.52354140513206	0.97962516548896	2.43563698525299
H	2.49941475580707	0.65698094936234	-1.56541631266989
H	2.82000045236680	0.94227353117777	0.15641027926170
H	2.26886136703694	-1.29958893961291	0.74787685020370
H	2.75607737987127	-1.63226956441048	-0.92254737899139
H	-3.30288210263105	0.65430229486059	0.43777432824509
H	-1.64718069375653	0.36423886930933	-1.61172481464493
H	0.45524060023963	-1.33101079642856	-1.69340983625627
H	0.18666906327666	-2.64947359282832	1.03487520527073
H	-1.04677874352366	-2.65405164212061	-0.21792505935365
H	1.73326462221591	-3.92337796516336	-0.48120609984917
H	0.51616371535676	-3.88861364215415	-1.75806533365754
H	0.21418584683408	-4.79037380816928	-0.27147420993837

Electronic Energy: -576.122696700026

Correction to Gibbs Free Energy: 0.18685658

(*R*,*R*)-**3a**

O	-2.64870376429575	-0.66592285556018	1.92742418668838
O	-0.63419912086191	-0.54702079342134	0.94729315927049
O	0.36391483390850	-0.76349360827829	-1.13622580094698
C	2.32443095129688	3.02030073952178	1.09691603458946
C	-2.26987575766331	0.75495036096963	0.00583859408709
C	-1.94593794256647	-0.21804943744748	1.07289580405103
C	1.08941354313622	2.38271126480800	0.48479618455766
C	-1.17537607779894	0.96959758023158	-0.70514046020950
C	-0.04421406344058	0.14370492752362	-0.16281352235056
C	1.22473416290005	0.87772990076148	0.33803196331686
C	1.77060740280059	-0.95633991390602	-1.03855142623901
C	2.29947973311579	0.40237769046871	-0.63542012732723
H	3.20713040773173	2.86703753981905	0.47135232621485
H	2.19146706071353	4.09639576134318	1.22258309912698
H	2.53648861358849	2.59323060810357	2.08076449977943
H	0.21653094703641	2.60516666458900	1.10659725558422
H	0.89240415239088	2.82974730689477	-0.49635105969633
H	-1.05391774548303	1.59804986225051	-1.57605133231692
H	-3.25991754915065	1.16586725395067	-0.12078070747726
H	1.43200787755010	0.45115315258798	1.32452708579171
H	2.11937563988133	-1.30863530266740	-2.00906688072689
H	1.99551112069833	-1.71818721105815	-0.28211643231801
H	3.29087748707266	0.35696126832431	-0.18340597511138
H	2.34774876253913	1.06284611349098	-1.50720930223809

Electronic Energy: -574.930896507969

Correction to Gibbs Free Energy: 0.16436825

(*S*,*R*)-**3a**

O	-1.87269954054104	0.15049184063742	2.79090780488107
O	-0.19005151035134	-0.03577320878601	1.31778444538773
O	0.83921102349917	0.97695192624845	-0.48984114192396
C	0.61987756847314	-3.86021170814850	-0.51796967786984
C	0.02937341240790	-2.58896604734297	0.06773000769126
C	-2.26103716052953	0.35478898330841	0.41237997541781

C	-1.48939064663736	0.15879424040057	1.66052784222766
C	-1.42547258227514	0.26635979659266	-0.60982533448107
C	-0.04312315387159	-0.01904064406437	-0.11157758604050
C	0.58922871834867	-1.33556707580097	-0.57352806995436
C	2.06095277269338	-1.03868977541681	-0.30311191565110
C	2.16243927571942	0.44385340721833	-0.62508058952702
H	0.17998299276587	-4.74562849166374	-0.05535915493516
H	0.43879281247649	-3.92320327882031	-1.59468364196233
H	1.69989235696446	-3.90946140897752	-0.35971736380925
H	-1.05872223753486	-2.59872338575093	-0.05802317596238
H	0.21396223269991	-2.54917795393281	1.14424680199698
H	0.43193556138788	-1.38167886030769	-1.65918342175456
H	-1.63605853717100	0.37319695624633	-1.66501380023381
H	-3.32354506234360	0.54467770041374	0.41431836815679
H	2.73750773547818	-1.64091331343110	-0.90934102042527
H	2.28162531221180	-1.22621522873559	0.75072709972720
H	2.82857389344100	0.98503718025391	0.04951180977382
H	2.48777051958820	0.62064084075951	-1.65440730192972

Electronic Energy: -574.933606321719

Correction to Gibbs Free Energy: 0.16442719

(*S*,*R*,*S*)-**2e**

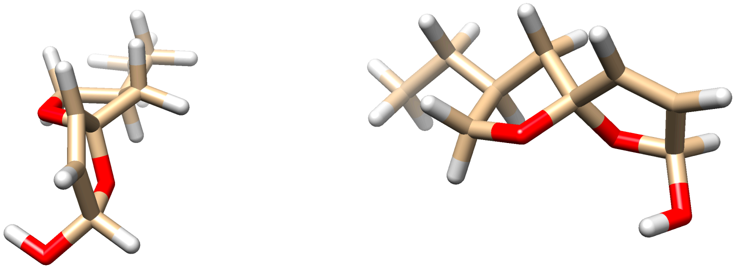

(*R*,*R*,*S*)-**2e**

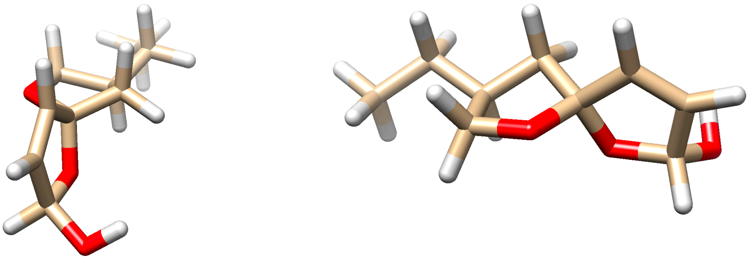

(*R*,*S*,*S*)-**2e**

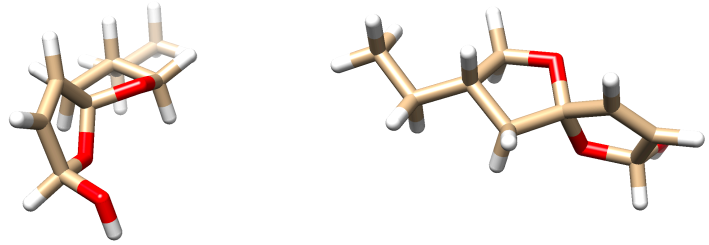

(*S*,*S*,*S*)-**2e**

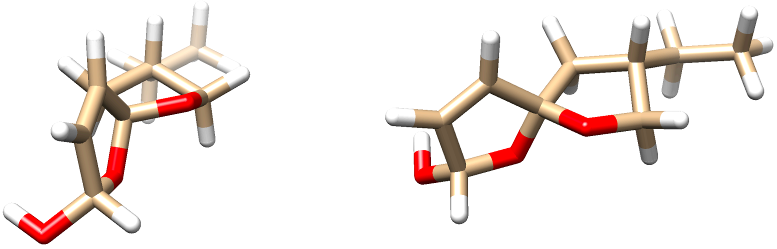

(*R*,*S*)-**3e**

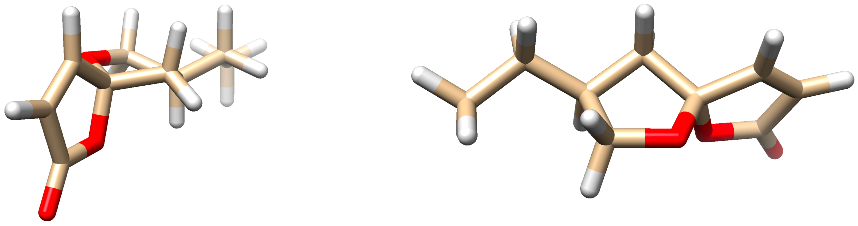

(*S*,*S*)-**3e**

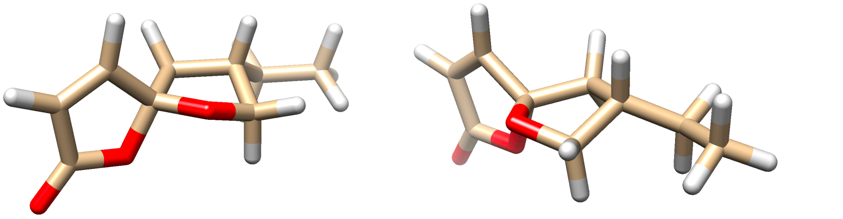

Supplementary Figure 5: Optimized geometries of **2e** and **3e** isomers.

Geometries & Energies

(*S*,*R*,*S*)-**2e**

O	-1.60833304256633	-2.29538235554458	1.52283156602826
O	0.01223866840141	-0.64572802902578	1.21257559301053
O	0.30648160170984	-0.53686186605313	-1.09658307058066
C	4.63142472331428	0.64768696969526	-1.21317835598666
C	-2.18113387232228	-0.03329565501312	0.89581224893399
C	-1.28412487951954	-0.94812914668960	1.67826505906272
C	3.41225228792484	1.42230389803326	-0.73979045996190
C	-1.47596461665174	0.59852847286673	-0.02526359725586
C	-0.04013335124806	0.18413129190293	0.06227158515375
C	1.02204294566897	1.26366246414487	0.17578187695984
C	1.68303556242293	-0.34176740421432	-1.40797884431072
C	2.26830996308185	0.52892159369094	-0.29906989253452
H	3.69469913886936	2.07392022874110	0.09363309563642
H	3.05836892565107	2.08384342107891	-1.53996683079641
H	2.61063026758321	-0.12144856375282	0.51341438009469
H	1.09193557095389	1.65622697637673	1.18896773797072
H	0.78264480195936	2.08349086016176	-0.50962726805339
H	4.40354024415947	0.03791771899498	-2.09129495483861
H	5.44831309183277	1.31992600665900	-1.48343637039980
H	4.99670478981639	-0.02317750044283	-0.43089727854573
H	-1.83141939956219	1.27727769530949	-0.78831804139328
H	-3.24776179123835	0.01671826414012	1.06664415844565
H	1.75232672082605	0.16416984049809	-2.37988666583296
H	2.16840142090982	-1.31679131030991	-1.49076123378978
H	-1.59194437875092	-2.48786092507461	0.57847179968219
H	-1.31070118752610	-0.77243593867346	2.75731322140151

Electronic Energy: -576.122303391922

Correction to Gibbs Free Energy: 0.18682596

(*R*,*R*,*S*)-**2e**

O	-1.26545051633831	-0.59662953695446	0.05174277429545
O	0.68471359771426	-0.02692944341652	-1.06930512021133
O	-3.22887058530959	-0.02796641825899	1.13501557552176
C	3.14085377694193	-3.70944460113087	-0.33623698996740
C	-1.56938776472400	1.63438692614264	0.52528755616671
C	-2.29144006334844	0.36418091280910	0.18187942641785
C	2.65300822098138	-2.64411411018935	0.63152728332191
C	-0.26798915918938	1.41381818890165	0.55578445028666
C	0.01778935796052	-0.00520984639049	0.16954659983482
C	0.89190645519642	-0.83923045997397	1.09228107172863
C	1.59133925844382	-1.12227233105463	-1.12963535195427
C	1.40523055439174	-1.92362095435777	0.15529341969172
H	0.60237796306333	-2.65307555389871	0.00147029508209
H	2.44995858138181	-3.10103577840837	1.60571843981253
H	3.44484795525701	-1.90383561111995	0.79939462553664

H	0.52258941507734	2.11919607990405	0.77230679211912
H	0.33278483986464	-1.21572600844559	1.94771987873758
H	1.72575042236324	-0.22789623021555	1.45380955749380
H	4.01981856022173	-4.22690735637387	0.05351123723737
H	2.36607951768226	-4.45941013962051	-0.51731799359258
H	3.41462883045683	-3.27801539333432	-1.30247381283848
H	-2.09187083420747	2.56117950399248	0.71939604337426
H	2.61402486075470	-0.72722612073567	-1.19643710752029
H	1.38586189970924	-1.70158056581702	-2.03254797018497
H	-2.85652742518165	0.43289091759423	-0.75373763393604
H	-2.78096679416337	-0.06512704374752	1.98696689944646

Electronic Energy: -576.121284669625

Correction to Gibbs Free Energy: 0.18658150

(*R*,*S*,*S*)-**2e**

O	-0.55002128484553	2.35631662605119	1.86759829515715
O	-0.44671542071923	0.17625988555560	1.01703895224838
O	1.03585717943503	0.38518923369722	-0.78337301396393
C	2.62852752579782	-3.62092520126567	-1.93376774731853
C	1.32455697801357	-3.27498405742553	-1.23357079542743
C	-1.79794507618691	1.71965100664492	-0.02653616655862
C	-1.25669034819967	1.29364217410481	1.30104603169533
C	-1.23653030239122	0.99732542646296	-0.97742915817139
C	-0.26277804474361	0.02405144775527	-0.38450933640765
C	-0.41637709408516	-1.46184667693280	-0.74934573493818
C	0.88561813775603	-1.83829537003408	-1.45136688136035
C	1.82542970633482	-0.78422486774774	-0.88433353553917
H	0.53257600561857	-3.94638675065098	-1.58157185411205
H	1.42224766197742	-3.45251000856660	-0.15589527731510
H	0.78459222183255	-1.65260740024744	-2.52746081755692
H	-1.30208670435193	-1.63786918651789	-1.36053902680733
H	-0.53202639700117	-2.02517000581398	0.17887941664869
H	2.89088049794027	-4.67082474831652	-1.78814418904686
H	2.55622640603254	-3.44269850251702	-3.01026485681190
H	3.45873968567674	-3.01961248941240	-1.55493241201810
H	-1.37534811115592	1.08551901026502	-2.04618431855201
H	-2.50190247112258	2.53244186193850	-0.13481338720291
H	2.19677384219408	-1.08792295230170	0.10609902269354
H	2.67914459438414	-0.55291889178203	-1.52187461181359
H	-2.03514525351591	0.94901137275115	1.99607567434938
H	-0.21745763537473	2.06910259710578	2.72272156942956

Electronic Energy: -576.120067210506

Correction to Gibbs Free Energy: 0.18630408

(*S*,*S*,*S*)-**2e**

O	-0.39913079579175	0.17429193533865	0.99464759343370
O	0.97802732103754	0.31395004549545	-0.87771205419867
O	-2.09972182675689	1.07632785864939	2.26354965293246
C	2.65670807403804	-3.69437385652428	-1.87453668100580
C	1.37133682585026	-3.35620950126929	-1.13694308449305
C	-1.78602747739402	1.66461070497666	-0.07605085840109
C	-1.17707513357601	1.32570260412037	1.25414100193429
C	-1.31488925875239	0.84841180699829	-1.00057988296365
C	-0.29231877236300	-0.06753732559084	-0.39511872324454
C	-0.40774177696303	-1.57407885955840	-0.66837702826773
C	0.87518054157271	-1.94739781481057	-1.40659304702822
C	1.80117277210451	-0.83529757042208	-0.93589854934163
H	0.58930652621530	-4.06932188430030	-1.41823519335186
H	1.51885944870238	-3.47645303488382	-0.05711784351538
H	0.72226917839410	-1.82034082782113	-2.48514661875483
H	-1.31293916519783	-1.81363964713707	-1.22802652639534
H	-0.46181541065766	-2.08552109735395	0.29507990066844
H	2.95996790691025	-4.72697790069468	-1.69038185085453
H	2.53472184069394	-3.57133471774101	-2.95430052450227
H	3.48081401172994	-3.04937769213495	-1.55980643523565
H	-1.54411349416482	0.83356437197026	-2.05747704707297
H	-2.49895530749478	2.46888273957825	-0.19634058830504
H	2.21198820865877	-1.06817099327071	0.05775614154322
H	2.62542996904530	-0.61856526288415	-1.61572631815935
H	-0.54213197174583	2.12925832497372	1.64336656784778
H	-2.68640311279504	0.37727276849623	1.95468895223169

Electronic Energy: -576.120008368303

Correction to Gibbs Free Energy: 0.18660820

(*R*,*S*)-**3e**

O	0.04517909795505	-0.22323035118496	-1.34488965140631
O	-0.10853889176080	-1.02346360796777	0.82483148295251
O	-1.54867608722136	-1.86758926608270	2.32469086769192
C	2.26336057406954	0.06897033244991	-0.54996525579461
C	1.40976223979212	-0.27795138363351	-1.76751395492055
C	1.27271180761456	0.86929566997366	0.28696214519357
C	-0.02902715674746	0.14458403247226	-0.00905769184677
C	-1.30639661292090	0.87371443424563	0.27281315550599
C	3.55382637038213	0.79608674420250	-0.87868122973474
C	-1.26270324978702	-1.01430251485684	1.54046737829827
C	-2.01470992704659	0.20585619482774	1.16884385516428
C	4.50813618316772	-0.03444193530192	-1.72077867714083
H	2.49682699376068	-0.85537782036892	-0.01105184491895
H	4.04595850965214	1.08524173545384	0.05548090722014
H	3.31142116416634	1.72957746878039	-1.40084604879424
H	1.49836335144140	0.88846376526159	1.35208705787450
H	1.20265623106299	1.89734150728564	-0.08263491804368

H	1.61217587363702	-1.27359772289855	-2.16633167941877
H	1.54556118666288	0.45561225967817	-2.57112353718678
H	-2.97790510551471	0.44264968713892	1.59446587530389
H	-1.55374722445866	1.79865314934528	-0.22981092920470
H	4.76193148697237	-0.97075867452839	-1.21678233527004
H	5.43859903414136	0.50359442413923	-1.91184661713829
H	4.07066047907917	-0.28890407553120	-2.68960632218580

Electronic Energy: -574.932758416653

Correction to Gibbs Free Energy: 0.16438437

(*S*,*S*)-**3e**

O	1.24934061665651	0.74676142748563	-0.23838515515305
O	-0.70488741466689	0.02340470887514	0.77189891176495
O	-2.68000275515119	0.80726583579405	1.49294706706342
C	2.24645686624139	-0.20006171809077	0.11969621791747
C	1.96747635513212	-1.40533333855156	-0.76687793170899
C	0.43836444620008	-1.37314369092245	-0.84771063349591
C	0.05756411566682	0.06129080156741	-0.44399652704493
C	-0.81609653075510	0.83100451854619	-1.38847929801586
C	-1.87679396456403	0.69282835336722	0.61765686036640
C	-1.93138196252333	1.19138844980986	-0.77556639373052
C	2.53707595222393	-2.71696147208640	-0.25659549084822
C	4.05538168274045	-2.72703677624720	-0.18983223648292
H	2.38273918368705	-1.19596126623973	-1.75924127760909
H	2.19336050709647	-3.52978671461336	-0.90449023659517
H	2.11746319419268	-2.91874006273635	0.73584464736816
H	0.06481300969098	-1.62210245093287	-1.84122564733935
H	-0.01269694550646	-2.06557485714892	-0.13546672658783
H	3.21530508727636	0.26925785683870	-0.04741261465639
H	2.15568047570859	-0.46355205763876	1.18229007818458
H	-2.76891495786176	1.75687542010995	-1.15445819732673
H	-0.50506153087178	1.03793529640131	-2.40338722580383
H	4.43229471195230	-1.97315330798874	0.50582531124487
H	4.49305895265464	-2.52027078400117	-1.17036815202604
H	4.42996965808014	-3.69668546529717	0.14357663961498

Electronic Energy: -574.930981763600

Correction to Gibbs Free Energy: 0.16409042

5.2 NMR spectroscopy

NOE spectra of compound 3c

major diastereomer

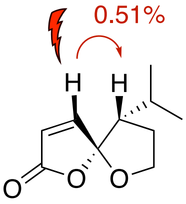

minor diastereomer

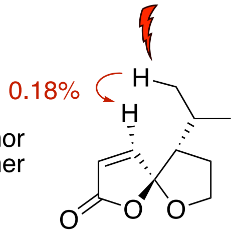

^{1}H NMR

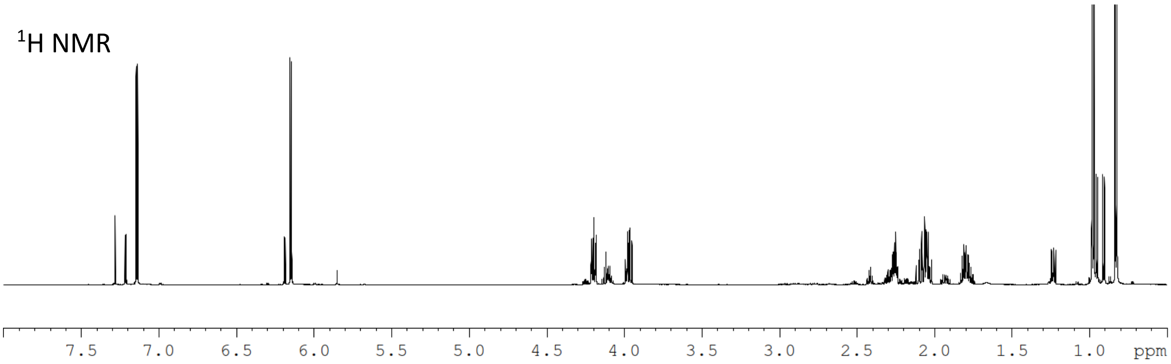

1D NOE (selective excitation: 7.12 ppm)

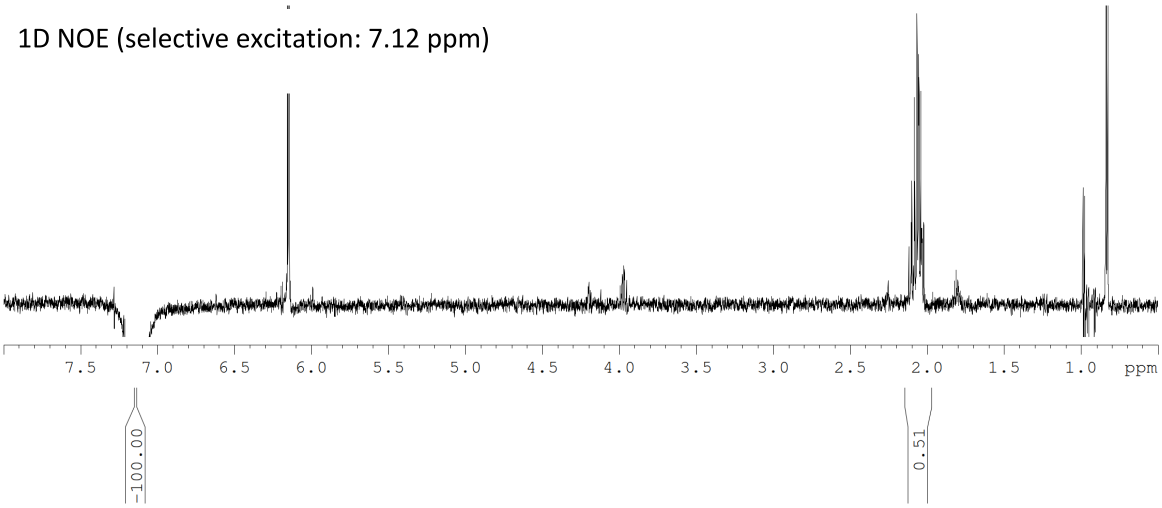

1D NOE (selective excitation: 0.95 ppm)

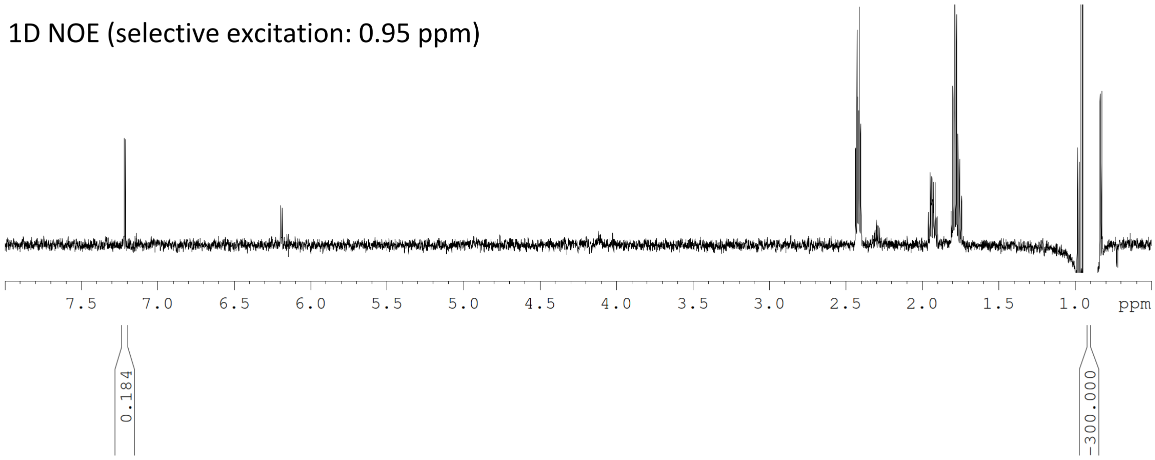

2D NOESY

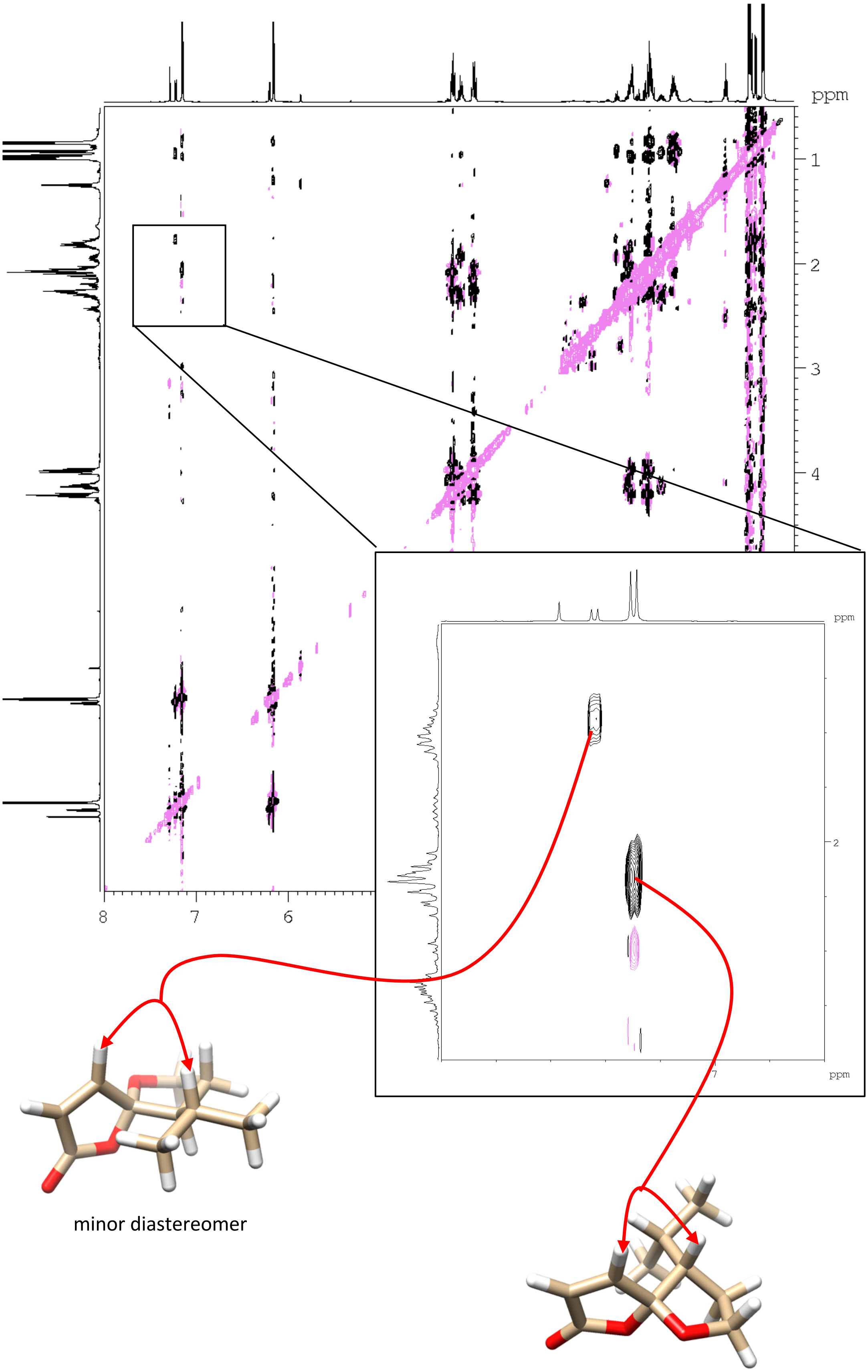

minor diastereomer

major diastereomer

NOE spectra of compound 3f

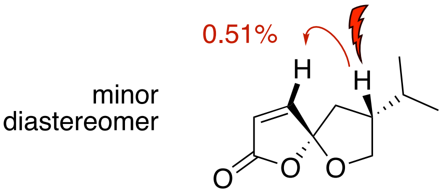

^{1}H NMR

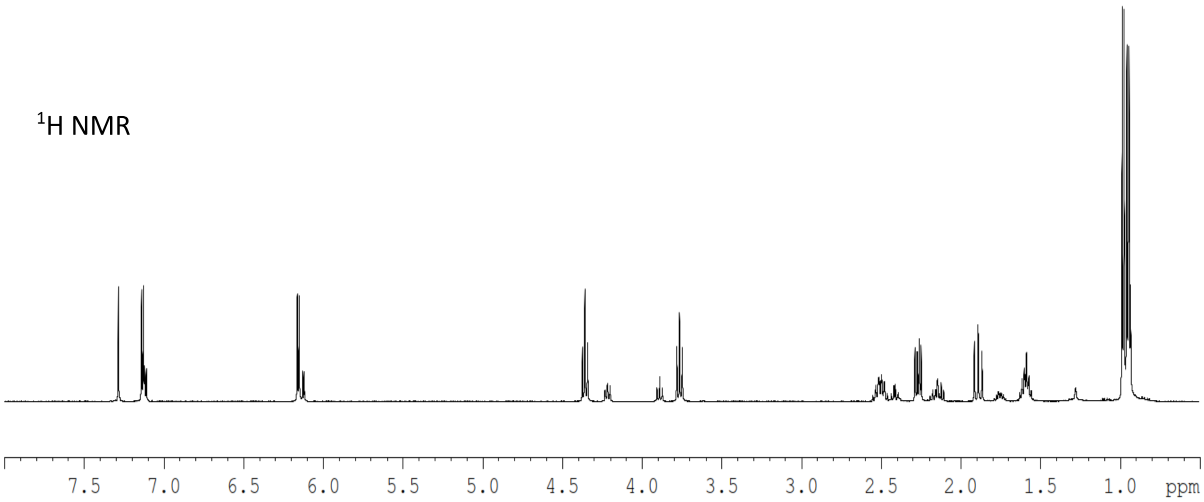

1D NOE (selective excitation: 2.44 ppm)

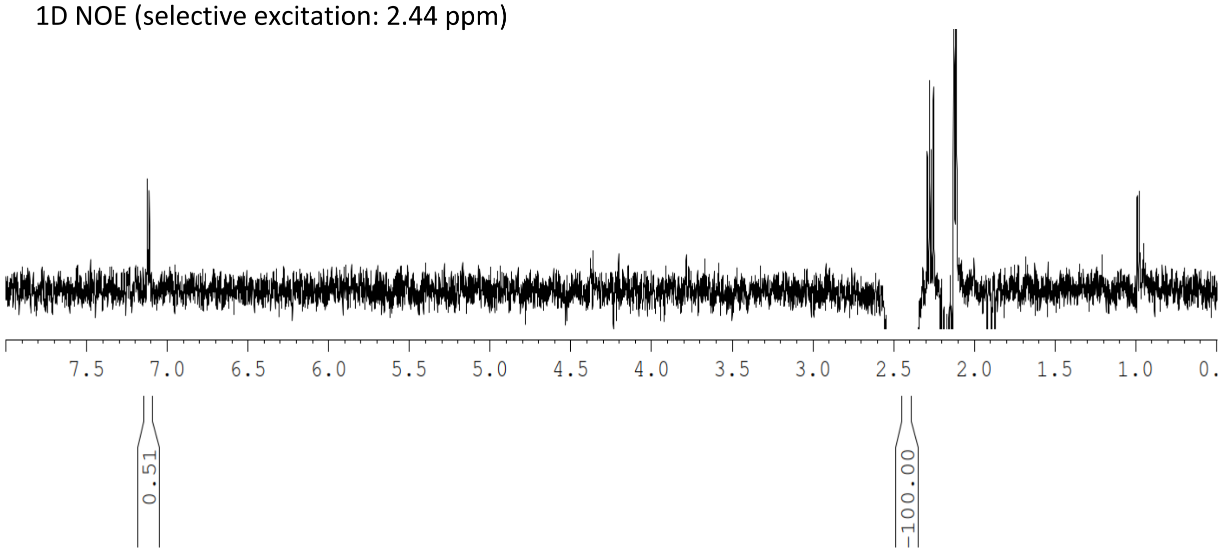

2D NOESY

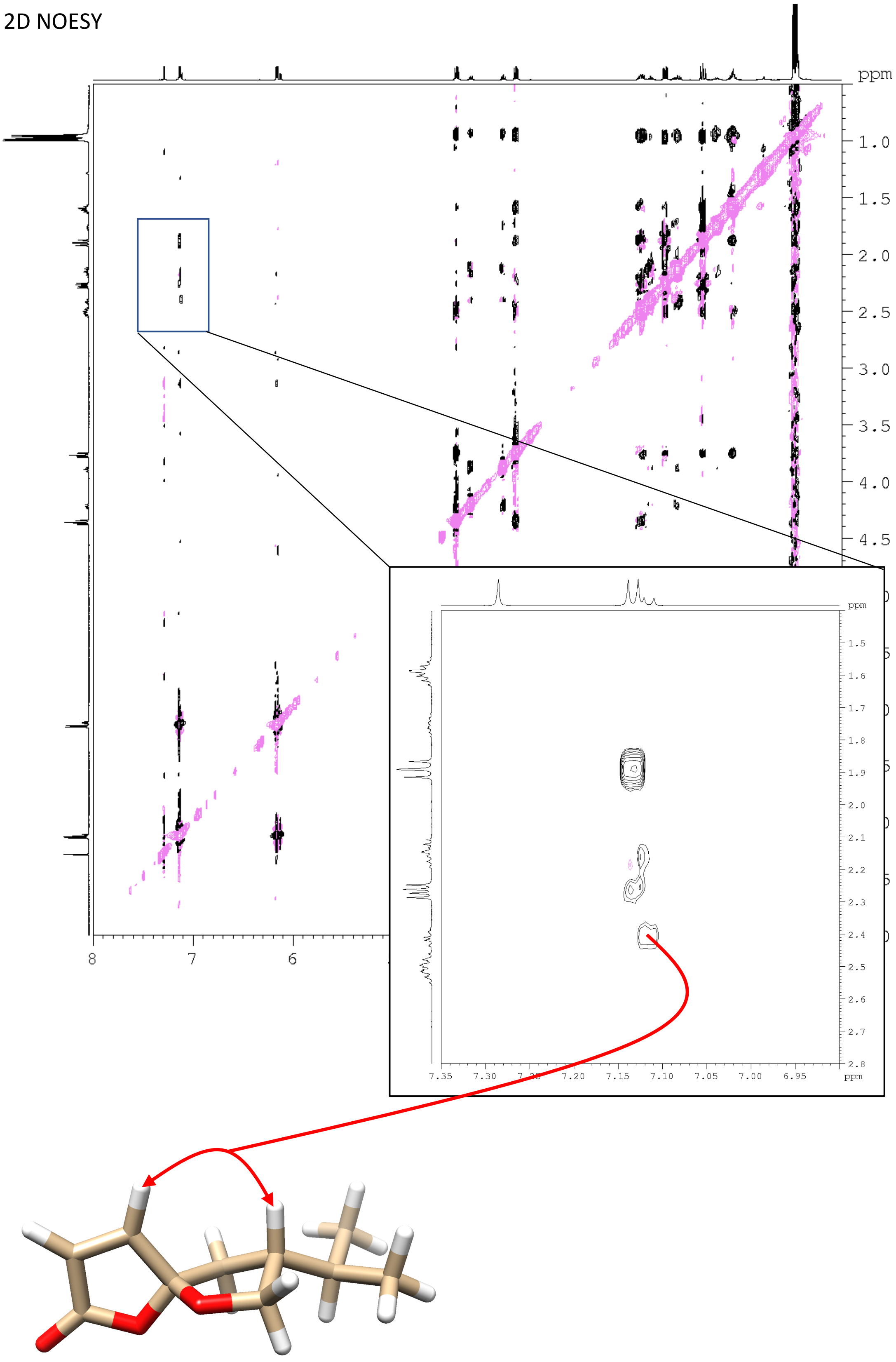

minor diastereomer

NOE spectra of compound 3i

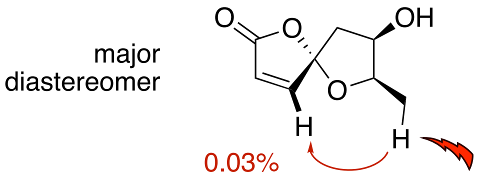

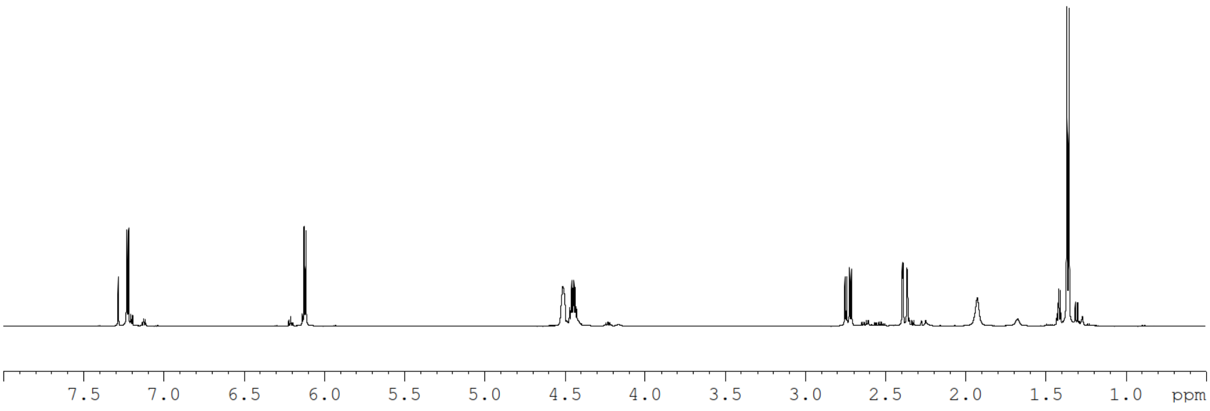

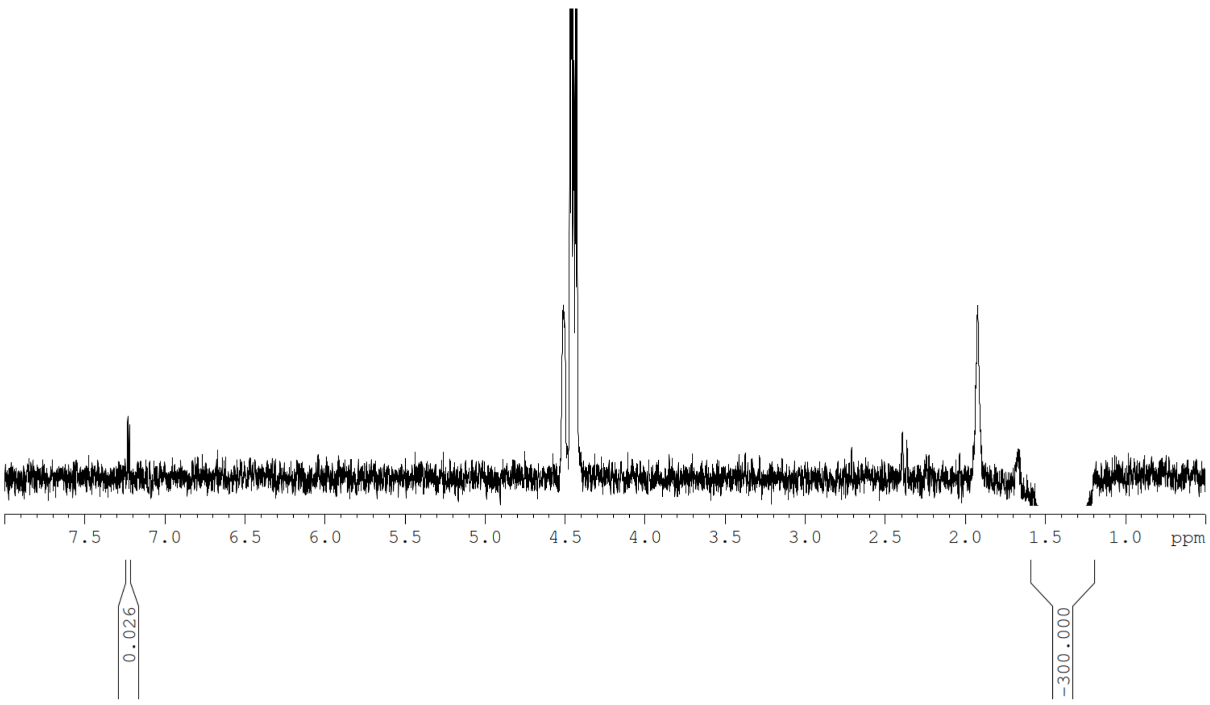

6. Spectra & Chromatograms

6.1 NMR spectra of the products

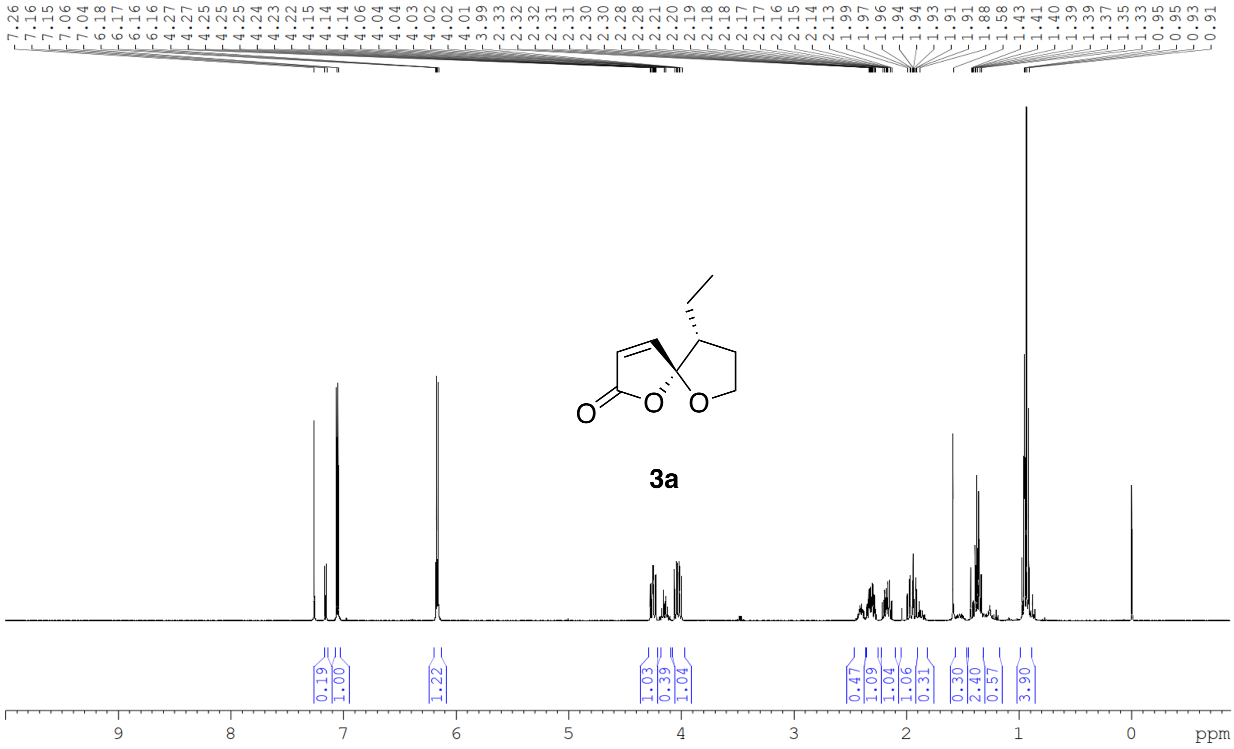

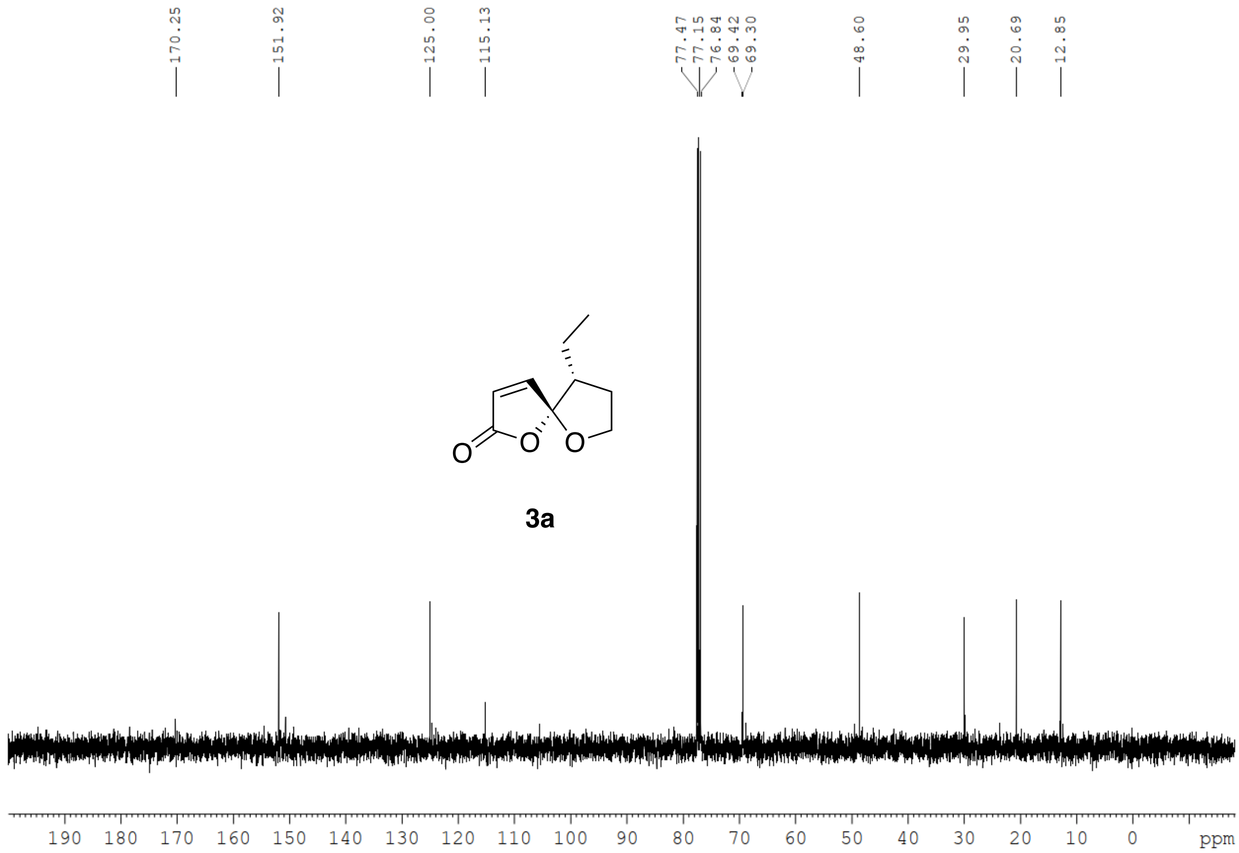

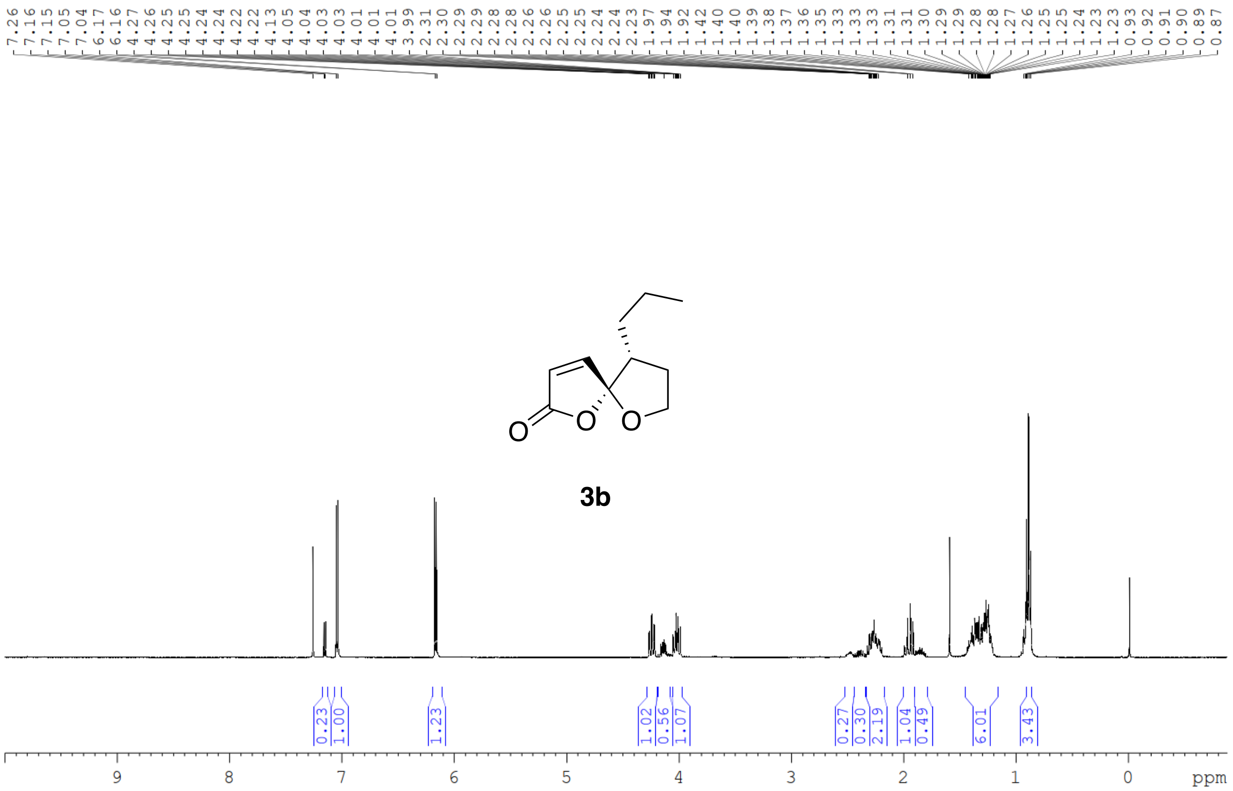
7.26
7.16
7.15
7.05
7.04
6.17
6.16
4.27
4.26
4.25
4.25
4.24
4.24
4.22
4.22
4.13
4.05
4.04
4.03
4.03
4.01
4.01
4.01
3.99
2.31
2.30
2.29
2.29
2.28
2.28
2.26
2.26
2.25
2.25
2.24
2.24
2.24
2.23
1.97
1.94
1.92
1.42
1.40
1.40
1.39
1.38
1.37
1.36
1.35
1.33
1.33
1.33
1.31
1.30
1.29
1.29
1.28
1.28
1.27
1.26
1.25
1.25
1.24
1.23
1.23
0.93
0.92
0.91
0.90
0.89
0.87
O
O
O
3b
0.23
1.00
1.23
1.02
0.56
1.07
0.27
0.30
2.19
1.04
0.49
6.01
3.43
9
8
7
6
5
4
3
2
1
0
ppm

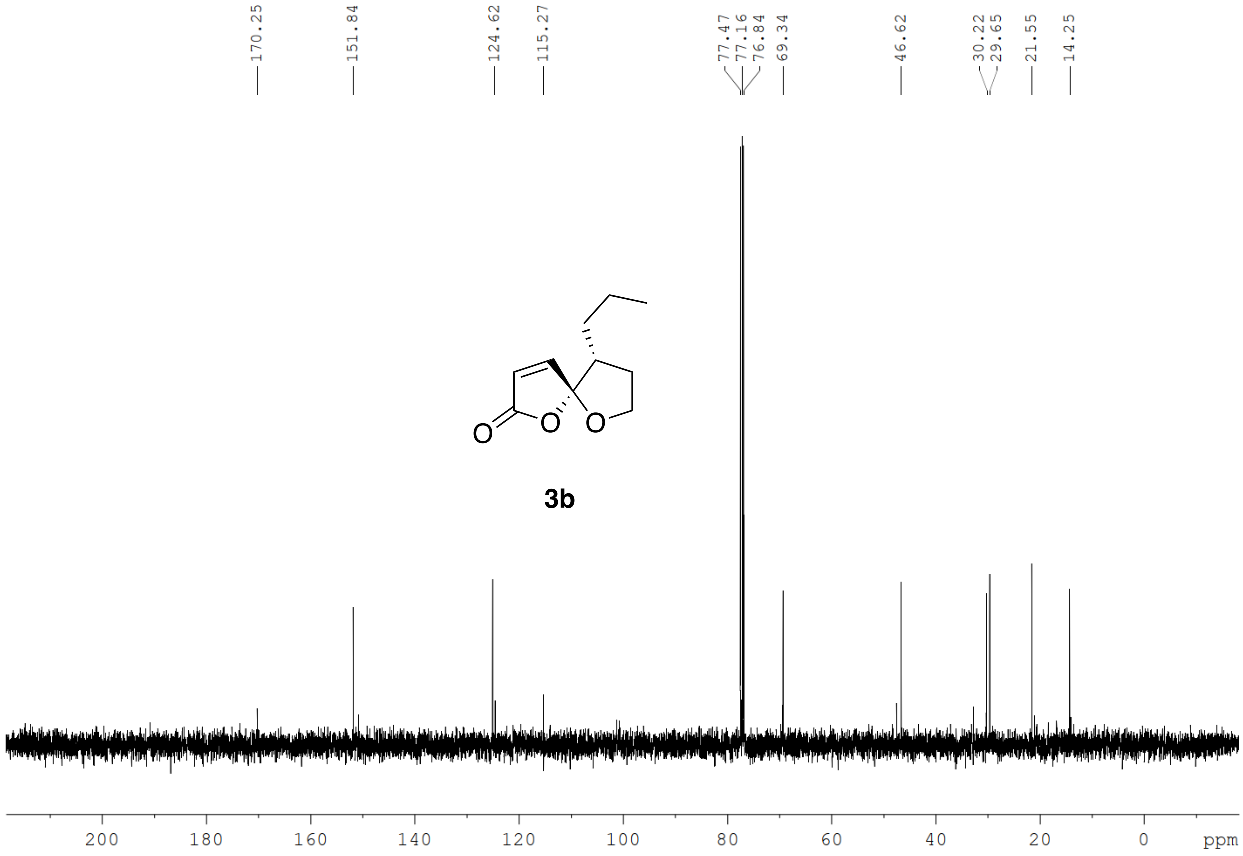
170.25
151.84
124.62
115.27
77.47
77.16
76.84
69.34
46.62
30.22
29.65
21.55
14.25
O
O
O
3b
200
180
160
140
120
100
80
60
40
20
0
ppm

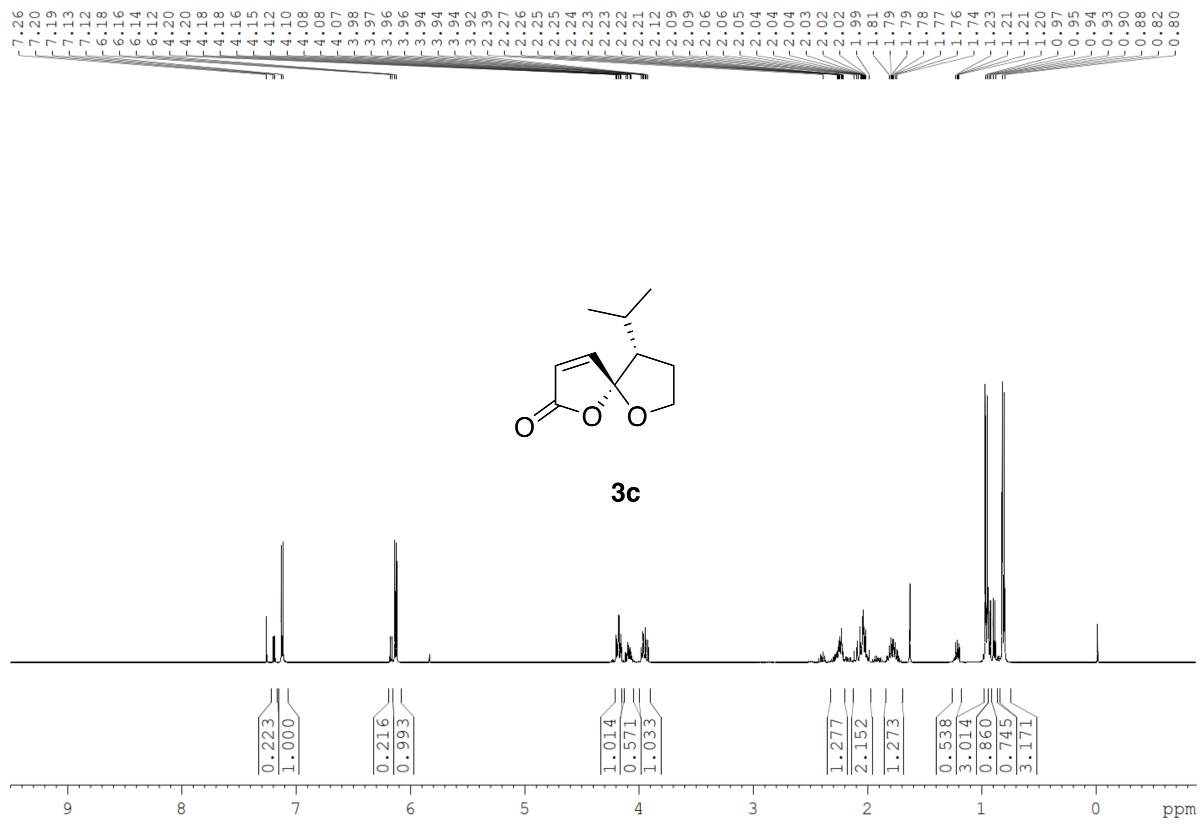

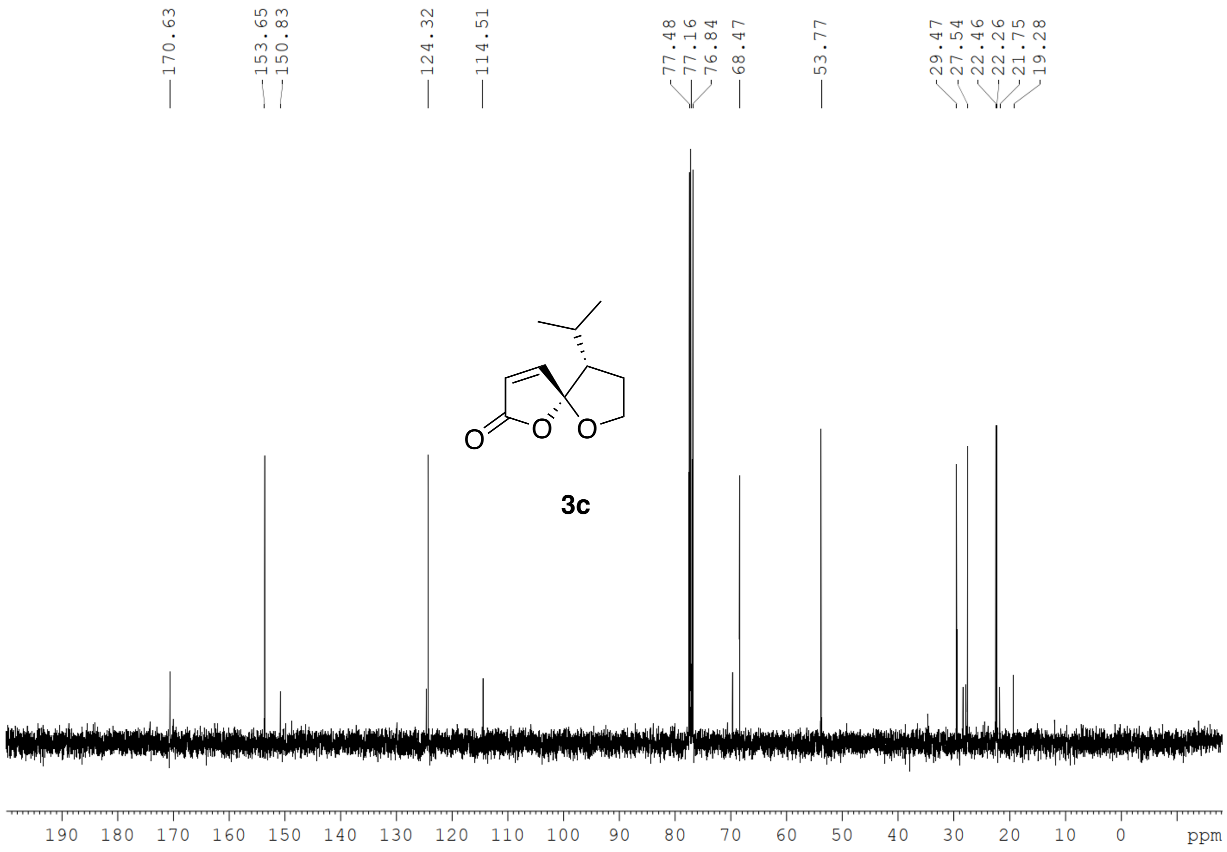

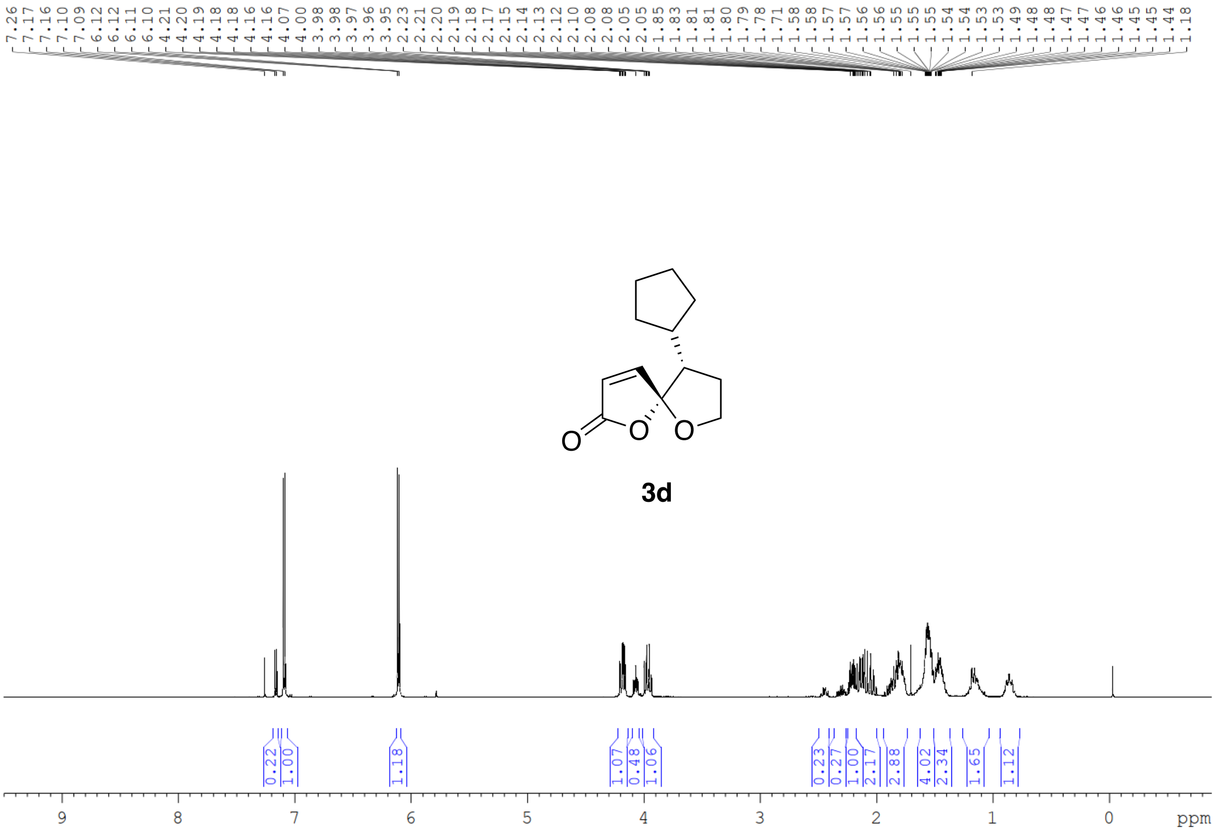
7.26
7.17
7.16
7.10
7.09
6.12
6.12
6.11
6.10
4.21
4.20
4.19
4.18
4.18
4.16
4.16
4.07
4.00
3.98
3.98
3.97
3.96
3.95
2.23
2.21
2.20
2.19
2.18
2.17
2.15
2.14
2.13
2.12
2.10
2.08
2.08
2.05
2.05
1.85
1.83
1.81
1.81
1.80
1.79
1.78
1.71
1.58
1.58
1.57
1.57
1.56
1.55
1.55
1.55
1.54
1.54
1.53
1.49
1.48
1.48
1.47
1.47
1.46
1.46
1.45
1.44
1.18
O
O
O
3d
0.22
1.00
1.18
1.07
0.48
1.06
0.23
0.27
1.00
2.17
2.88
4.02
2.34
1.65
1.12
9
8
7
6
5
4
3
2
1
0
ppm

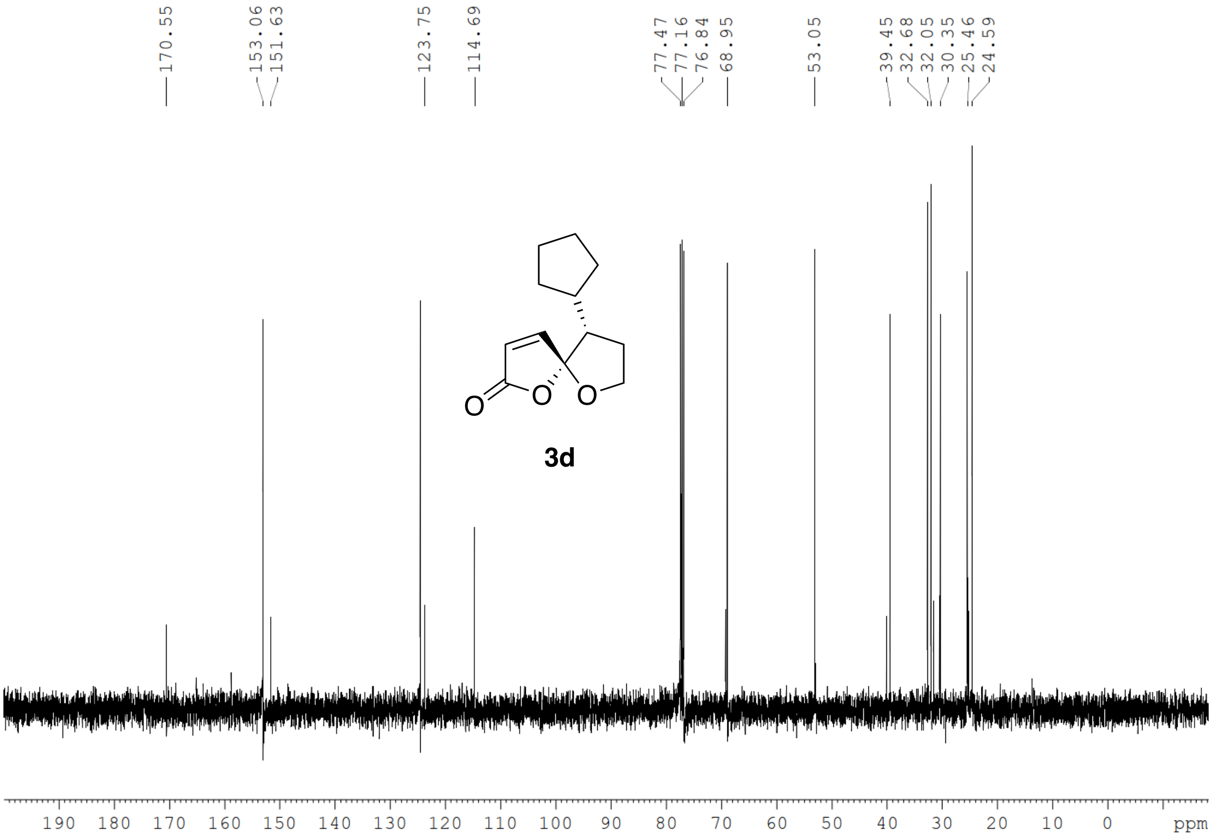
170.55
153.06
151.63
123.75
114.69
77.47
77.16
76.84
68.95
53.05
39.45
32.68
32.05
30.35
25.46
24.59
O
O
O
3d
190
180
170
160
150
140
130
120
110
100
90
80
70
60
50
40
30
20
10
0
ppm

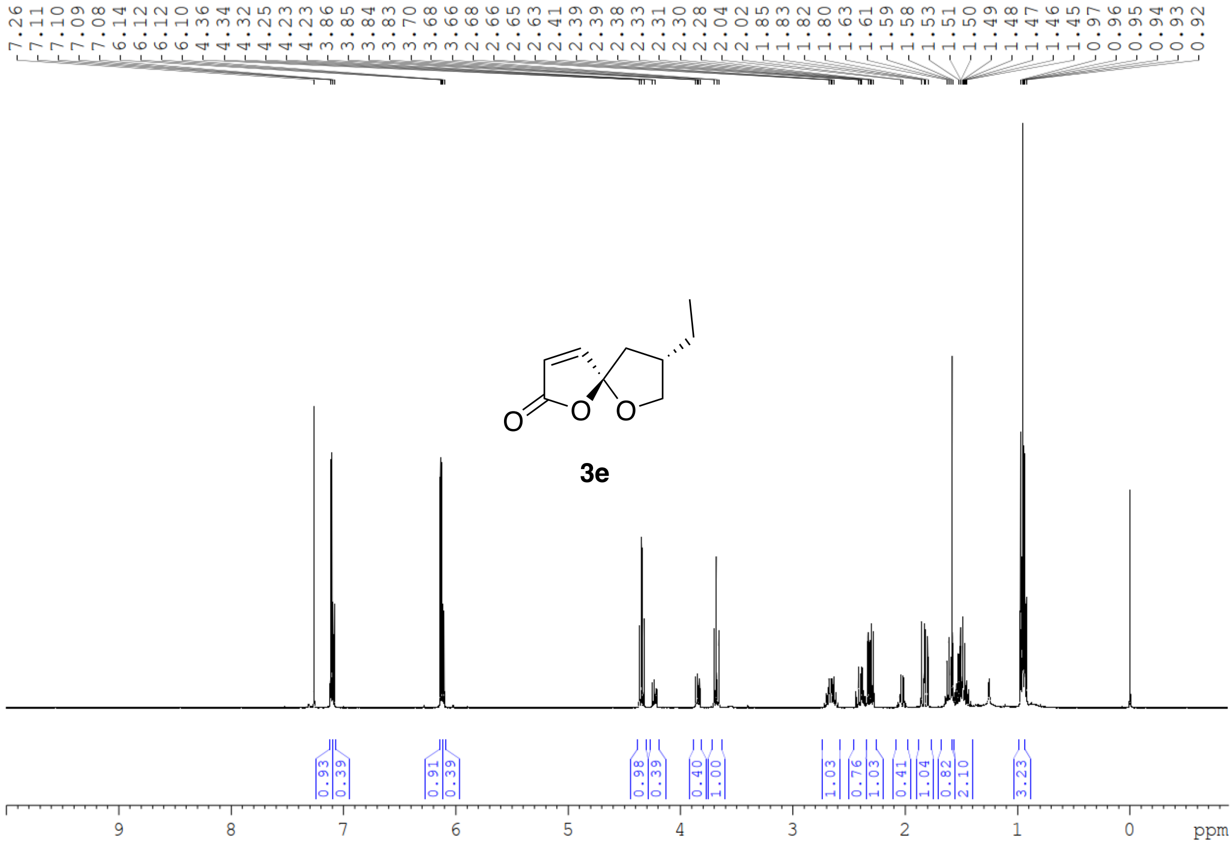
7.26 7.11 7.10 7.09 7.08 6.14 6.12 6.12 6.10 4.36 4.34 4.32 4.25 4.23 4.23 3.86 3.85 3.84 3.83 3.70 3.68 3.66 2.68 2.66 2.65 2.63 2.41 2.39 2.39 2.38 2.33 2.31 2.30 2.28 2.04 2.02 1.85 1.83 1.82 1.80 1.63 1.61 1.59 1.58 1.53 1.51 1.50 1.49 1.48 1.47 1.46 1.45 0.97 0.96 0.95 0.94 0.93 0.92
O
O
O
3e
0.93 0.39 0.91 0.39 0.98 0.39 0.40 1.00 1.03 0.76 1.03 0.41 1.04 0.82 2.10 3.23
9 8 7 6 5 4 3 2 1 0 ppm

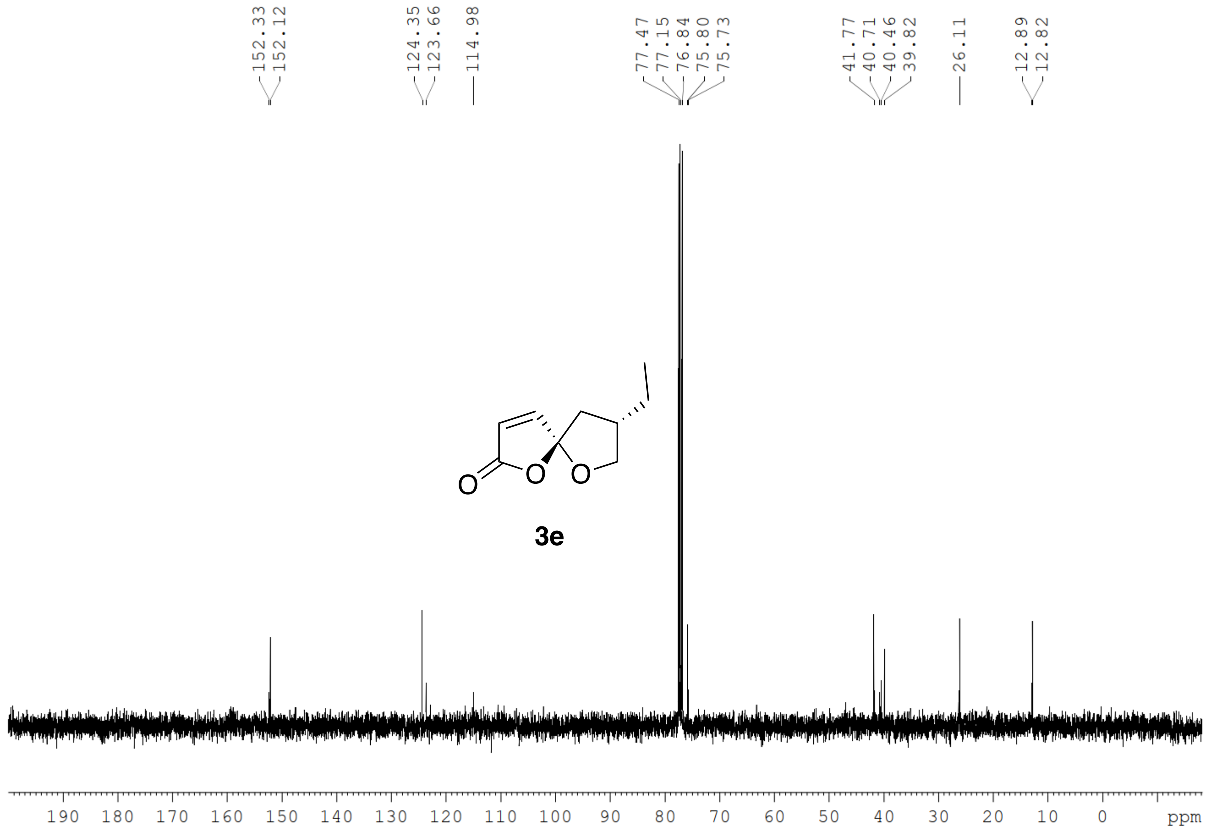
152.33 152.12 124.35 123.66 114.98 77.47 77.15 76.84 75.80 75.73 41.77 40.71 40.46 39.82 26.11 12.89 12.82
O
O
O
3e
190 180 170 160 150 140 130 120 110 100 90 80 70 60 50 40 30 20 10 0 ppm

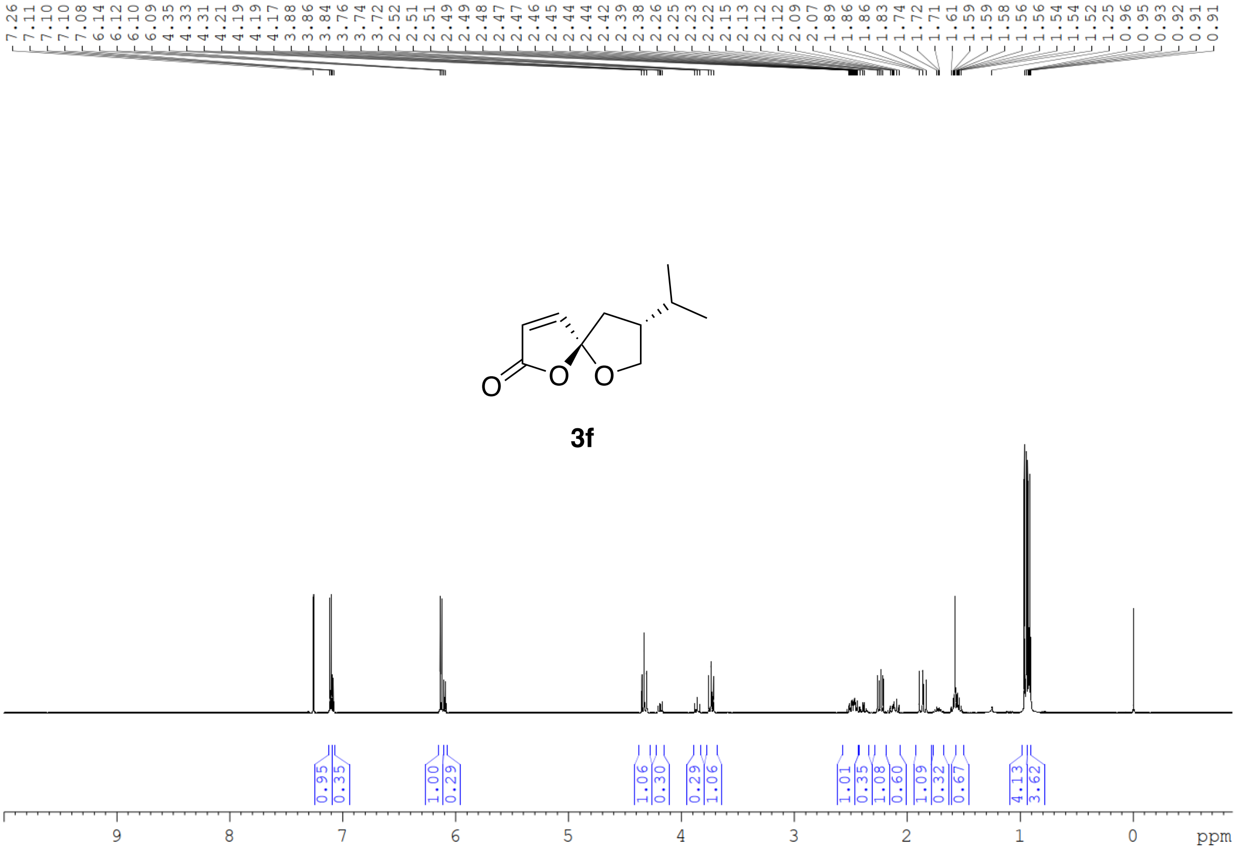
7.26 7.11 7.10 7.10 7.08 6.14 6.12 6.10 6.09 4.35 4.33 4.31 4.21 4.19 4.17 3.88 3.86 3.84 3.76 3.74 3.72 2.52 2.51 2.51 2.49 2.49 2.48 2.47 2.47 2.46 2.45 2.44 2.44 2.42 2.39 2.38 2.26 2.25 2.23 2.22 2.15 2.13 2.12 2.12 2.09 2.07 1.89 1.86 1.86 1.83 1.74 1.72 1.71 1.61 1.59 1.59 1.58 1.56 1.56 1.54 1.54 1.52 1.25 0.96 0.95 0.93 0.92 0.91 0.91
O
O
O
3f
0.95 0.35 1.00 0.29 1.06 0.30 0.29 1.06 1.01 0.35 1.08 0.60 1.09 0.32 0.67 4.13 3.62
9 8 7 6 5 4 3 2 1 0 ppm

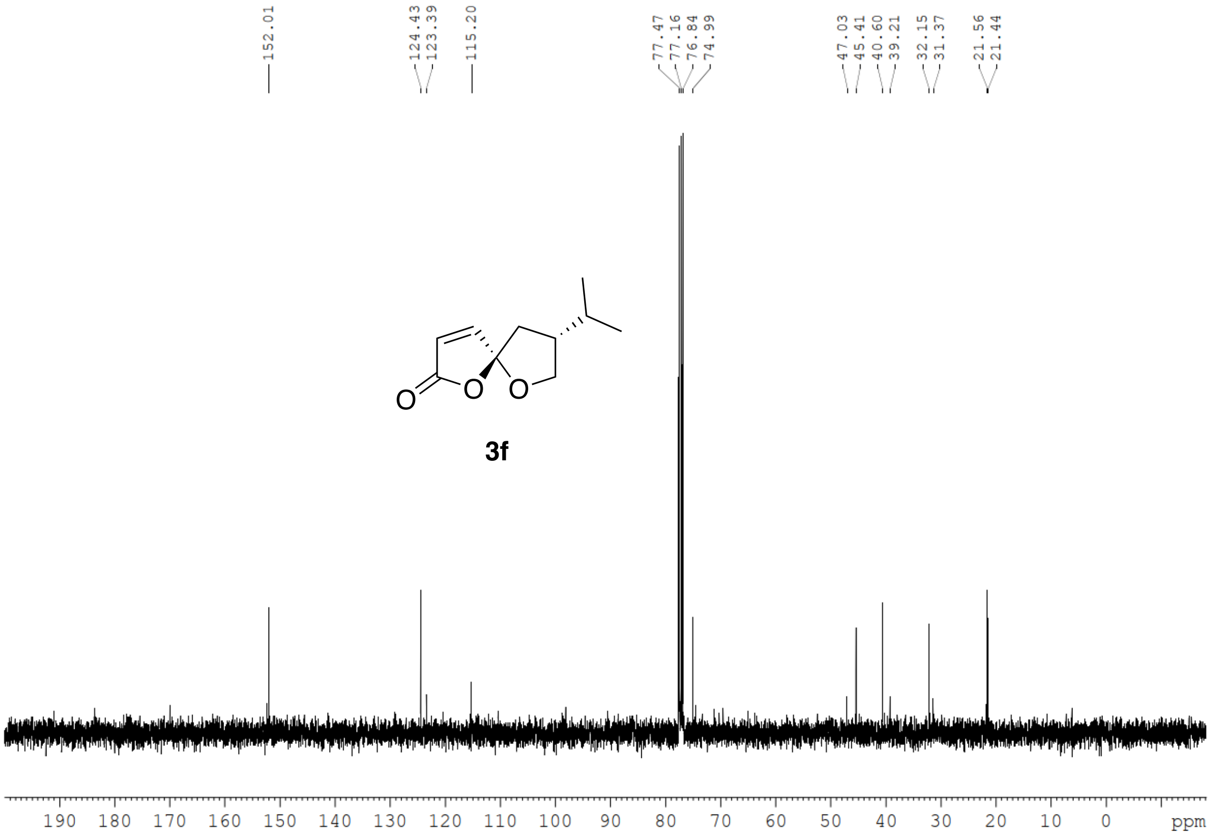
152.01 124.43 123.39 115.20 77.47 77.16 76.84 74.99 47.03 45.41 40.60 39.21 32.15 31.37 21.56 21.44
O
O
O
3f
190 180 170 160 150 140 130 120 110 100 90 80 70 60 50 40 30 20 10 0 ppm

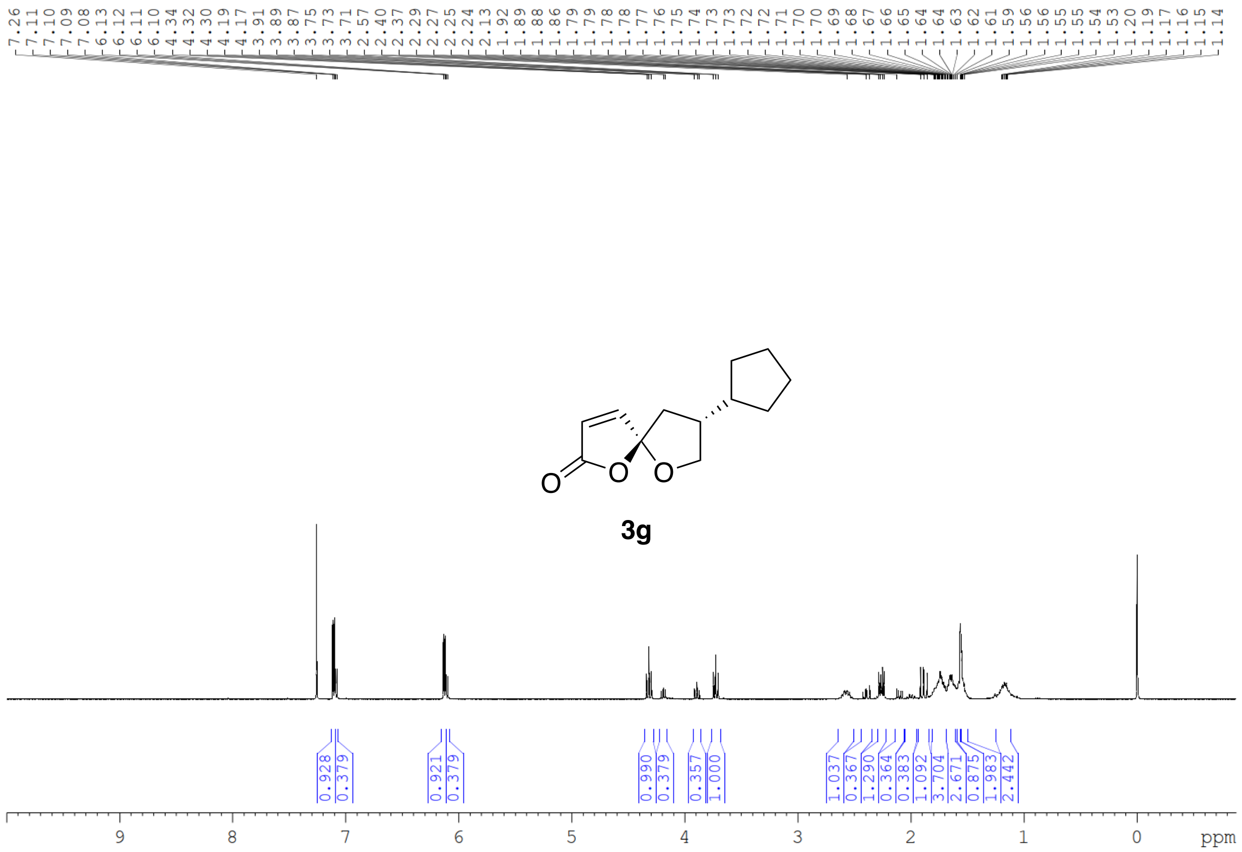
7.26 7.11 7.10 7.09 7.08 6.13 6.12 6.11 6.10 4.34 4.32 4.30 4.19 4.17 3.91 3.89 3.87 3.75 3.73 3.71 2.57 2.40 2.37 2.29 2.27 2.25 2.24 2.13 1.92 1.89 1.88 1.86 1.79 1.79 1.78 1.77 1.76 1.75 1.74 1.73 1.73 1.72 1.72 1.71 1.70 1.70 1.69 1.68 1.67 1.66 1.65 1.64 1.64 1.63 1.62 1.61 1.59 1.56 1.55 1.54 1.53 1.20 1.19 1.17 1.16 1.15 1.14
O
O
O
3g
0.928 0.379 0.921 0.379 0.990 0.379 0.357 1.000 1.037 0.367 1.290 0.364 0.383 1.092 3.704 2.671 0.875 1.983 2.442
9 8 7 6 5 4 3 2 1 0 ppm

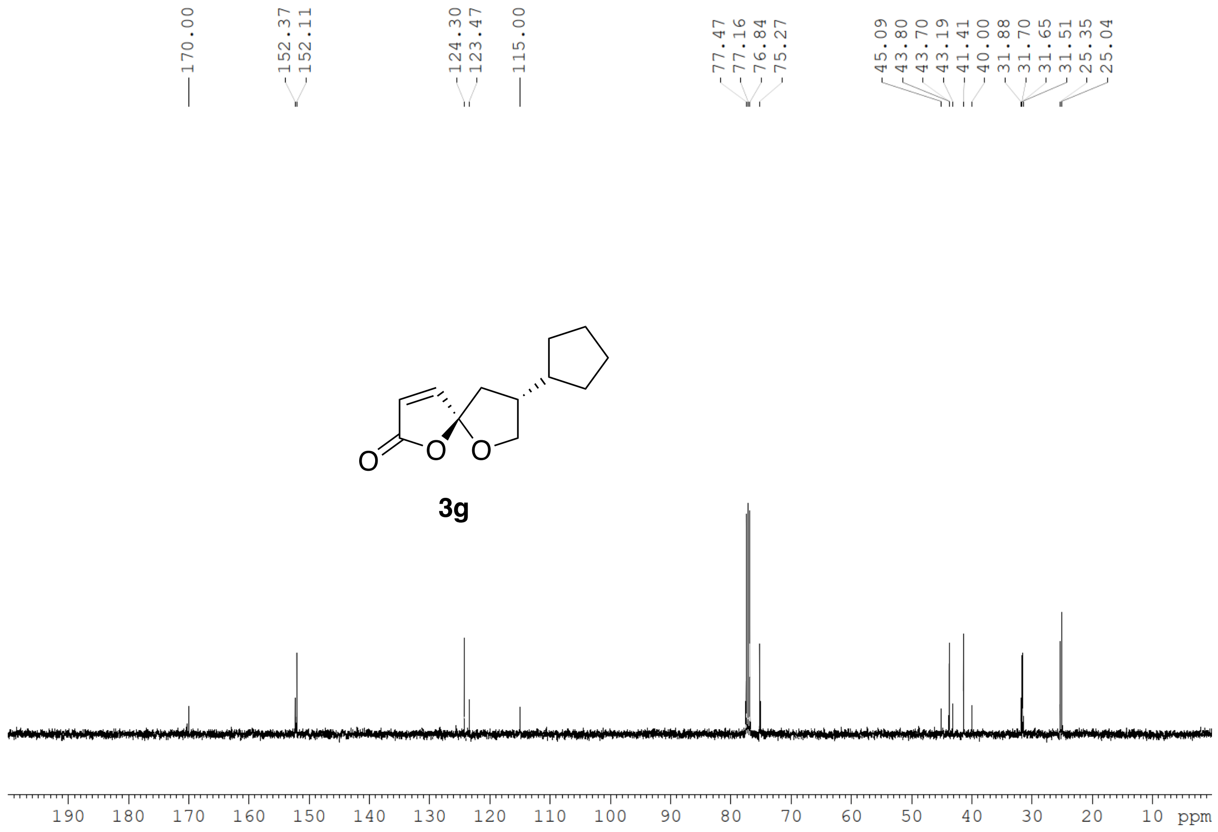
170.00 152.37 152.11 124.30 123.47 115.00 77.47 77.16 76.84 75.27 45.09 43.80 43.70 43.19 41.41 40.00 31.88 31.70 31.65 31.51 25.35 25.04
O
O
O
3g
190 180 170 160 150 140 130 120 110 100 90 80 70 60 50 40 30 20 10 ppm

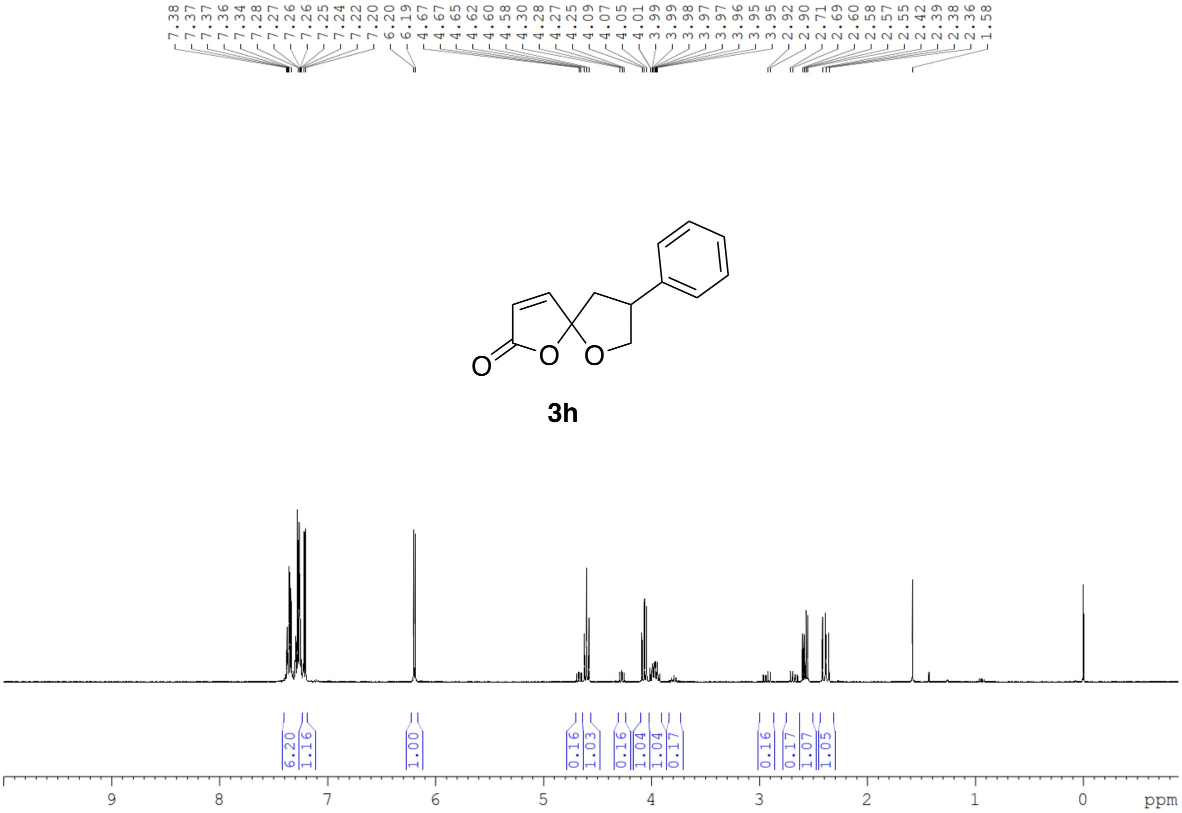
7.38 7.37 7.37 7.36 7.34 7.28 7.27 7.26 7.26 7.25 7.24 7.22 7.20 6.20 6.19 4.67 4.65 4.62 4.60 4.58 4.30 4.28 4.27 4.25 4.09 4.07 4.05 4.01 3.99 3.99 3.98 3.97 3.96 3.95 3.95 2.92 2.90 2.71 2.69 2.60 2.58 2.57 2.55 2.42 2.39 2.38 2.36 1.58
O
O
O
3h
6.20 1.16 1.00 0.16 1.03 0.16 1.04 1.04 0.17 0.16 0.17 1.07 1.05
9 8 7 6 5 4 3 2 1 0 ppm

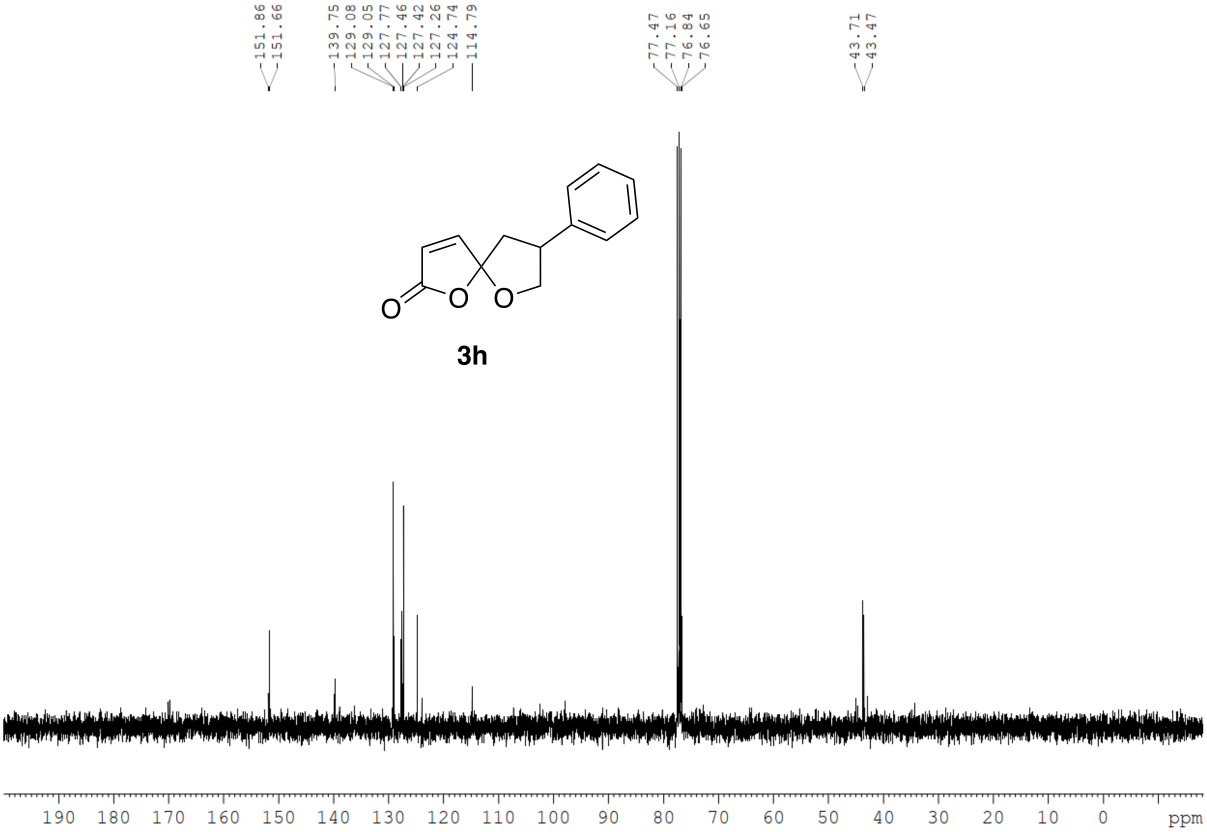
151.86 151.66 139.75 129.08 129.05 127.77 127.46 127.42 127.26 124.74 114.79 77.47 77.16 76.84 76.65 43.71 43.47
O
O
O
3h
190 180 170 160 150 140 130 120 110 100 90 80 70 60 50 40 30 20 10 0 ppm

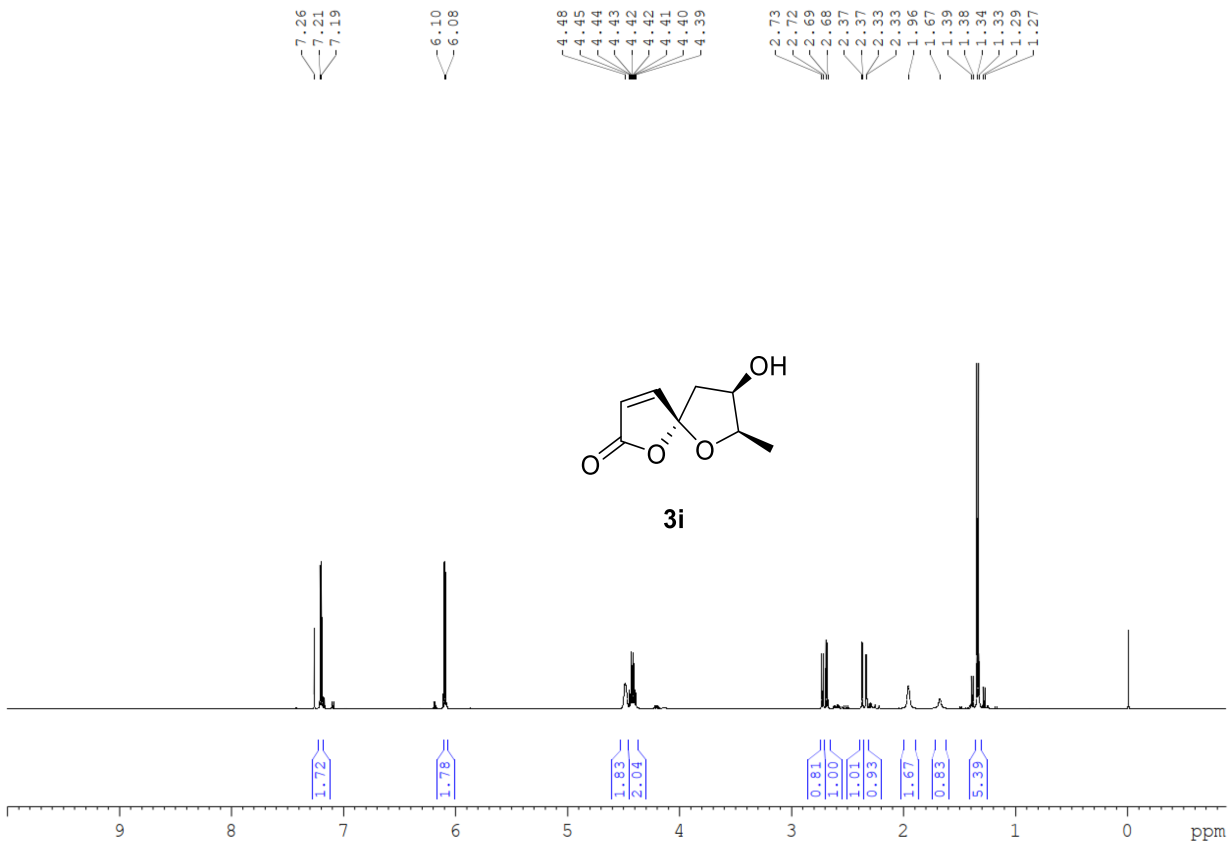
7.26
7.21
7.19
6.10
6.08
4.48
4.45
4.44
4.43
4.42
4.42
4.41
4.40
4.39
2.73
2.72
2.69
2.68
2.37
2.37
2.33
2.33
1.96
1.67
1.39
1.38
1.34
1.33
1.29
1.27
OH
O
O
O
3i
1.72
1.78
1.83
2.04
0.81
1.00
1.01
0.93
1.67
0.83
5.39
9
8
7
6
5
4
3
2
1
0
ppm

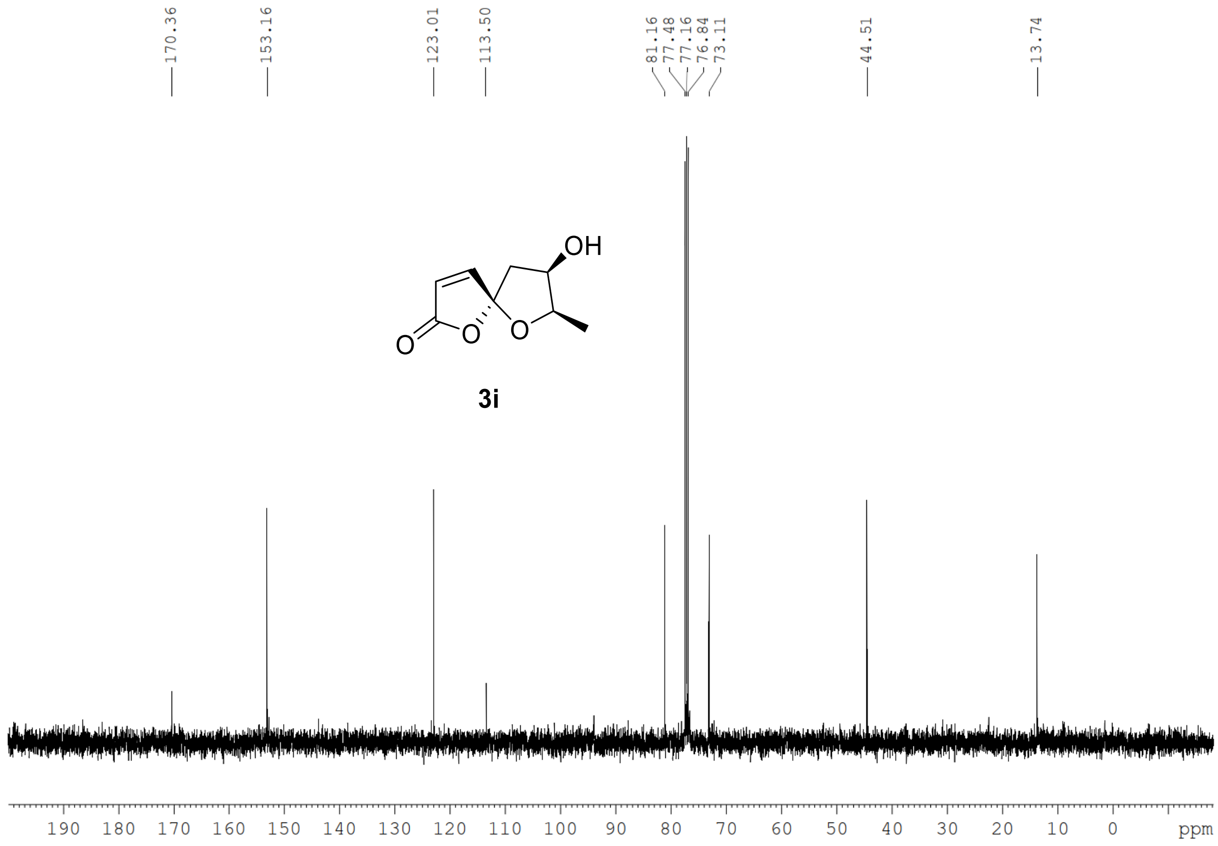
170.36
153.16
123.01
113.50
81.16
77.48
77.16
76.84
73.11
44.51
13.74
OH
O
O
O
3i
190
180
170
160
150
140
130
120
110
100
90
80
70
60
50
40
30
20
10
0
ppm

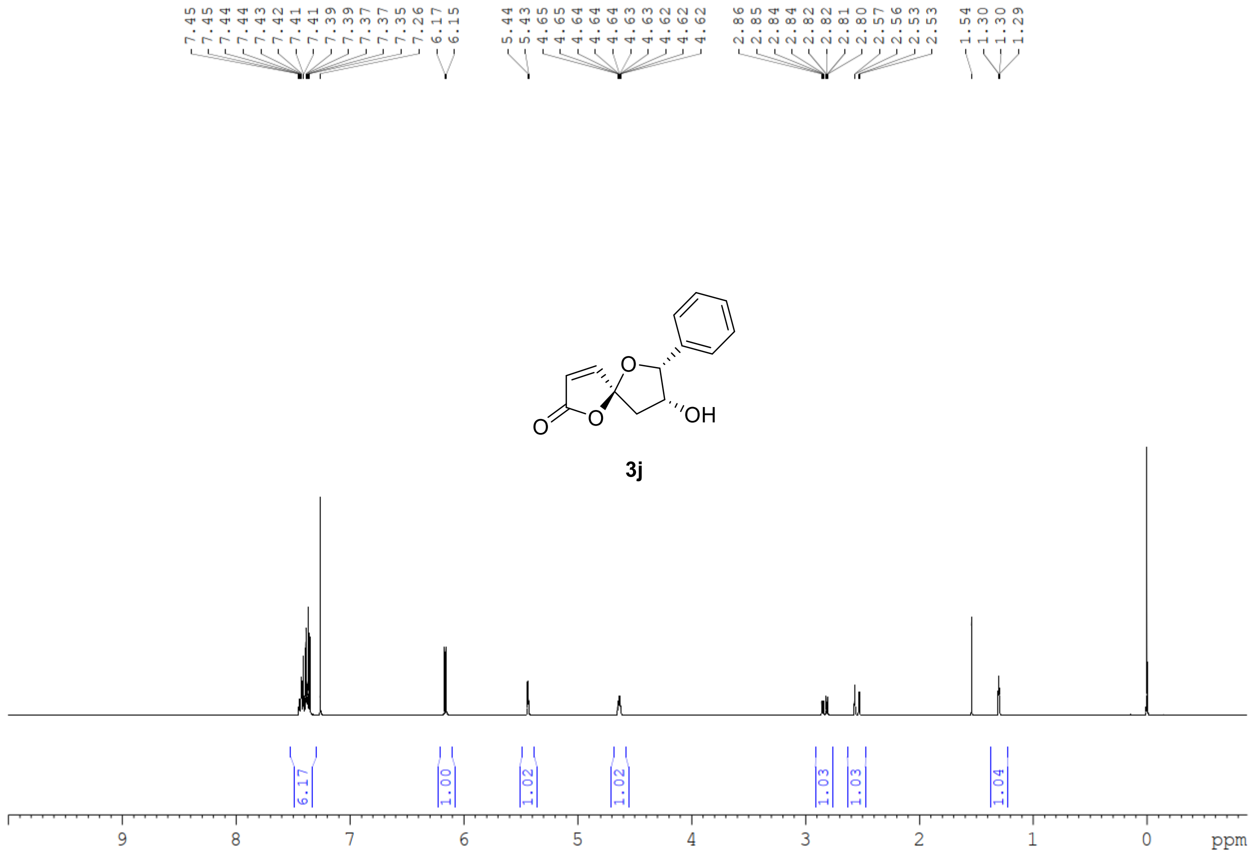
7.45 7.45 7.44 7.44 7.43 7.42 7.41 7.41 7.39 7.39 7.37 7.37 7.35 7.26 6.17 6.15
5.44 5.43 4.65 4.64 4.64 4.64 4.63 4.63 4.62 4.62 4.62
2.86 2.85 2.84 2.84 2.82 2.82 2.81 2.80 2.57 2.56 2.53 2.53
1.54 1.30 1.30 1.29
O
O
O
OH
3j
6.17 1.00 1.02 1.02 1.03 1.03 1.04
9 8 7 6 5 4 3 2 1 0 ppm

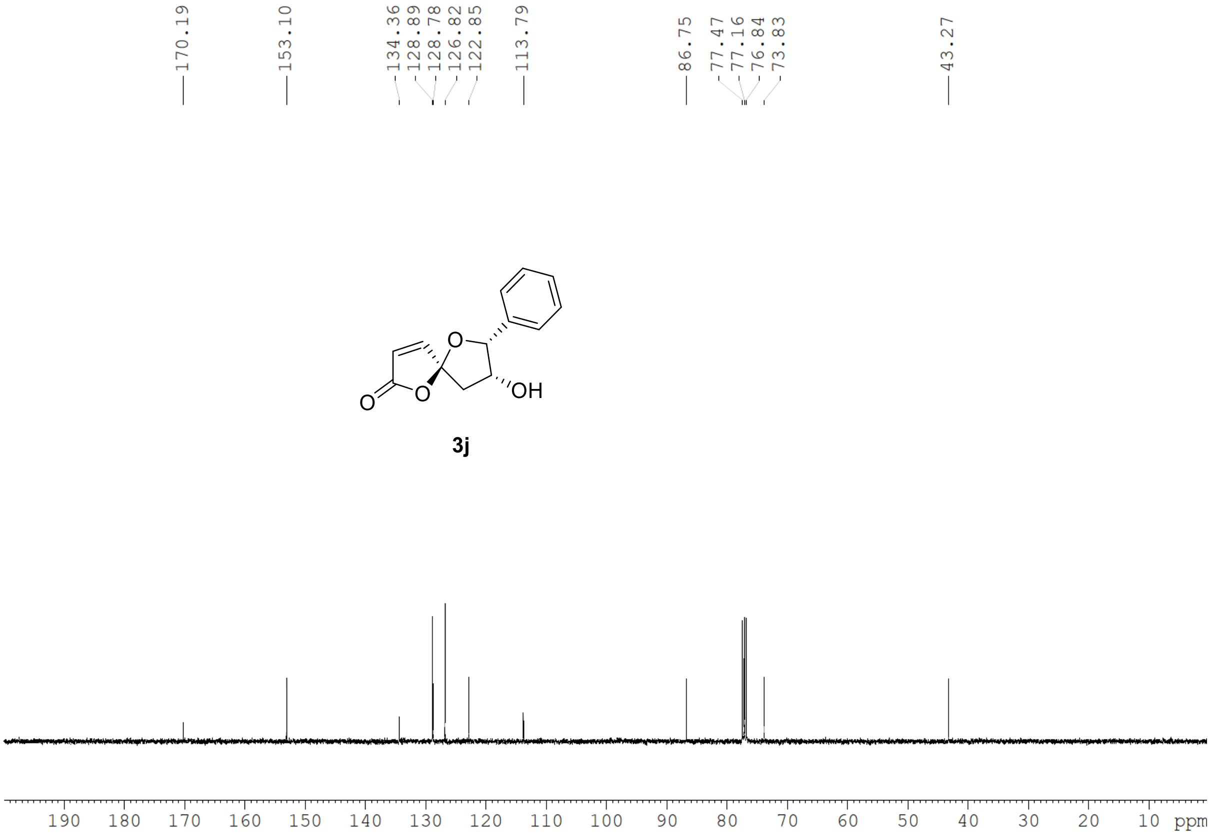
170.19
153.10
134.36 128.89 128.78 126.82 122.85
113.79
86.75
77.47 77.16 76.84 73.83
43.27
O
O
O
OH
3j
190 180 170 160 150 140 130 120 110 100 90 80 70 60 50 40 30 20 10 ppm

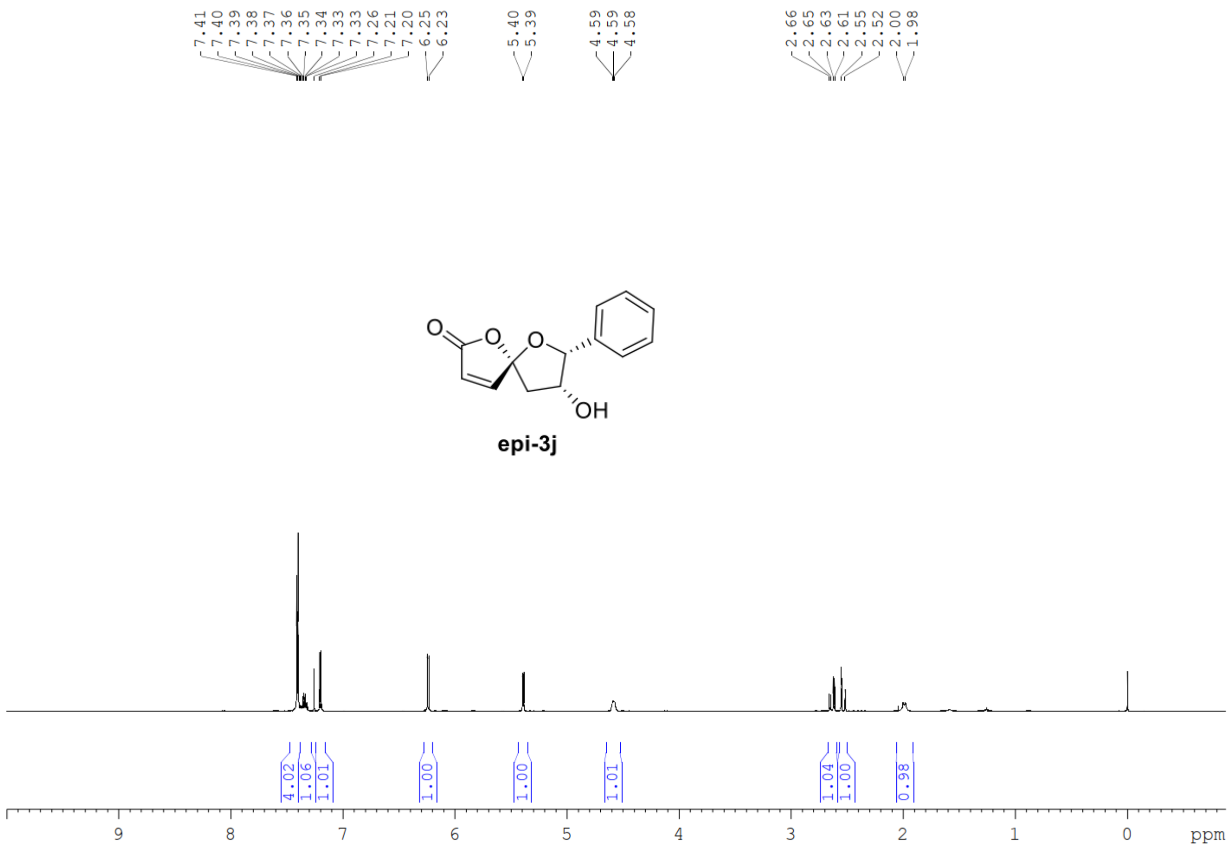
7.41 7.40 7.39 7.38 7.37 7.36 7.35 7.34 7.33 7.33 7.26 7.21 7.20 6.25 6.23
5.40 5.39
4.59 4.59 4.58
2.66 2.65 2.63 2.61 2.55 2.52 2.00 1.98
O O O OH
4.02 1.06 1.01 1.00 1.00 1.01 1.04 1.00 0.98
9 8 7 6 5 4 3 2 1 0 ppm

epi-3j

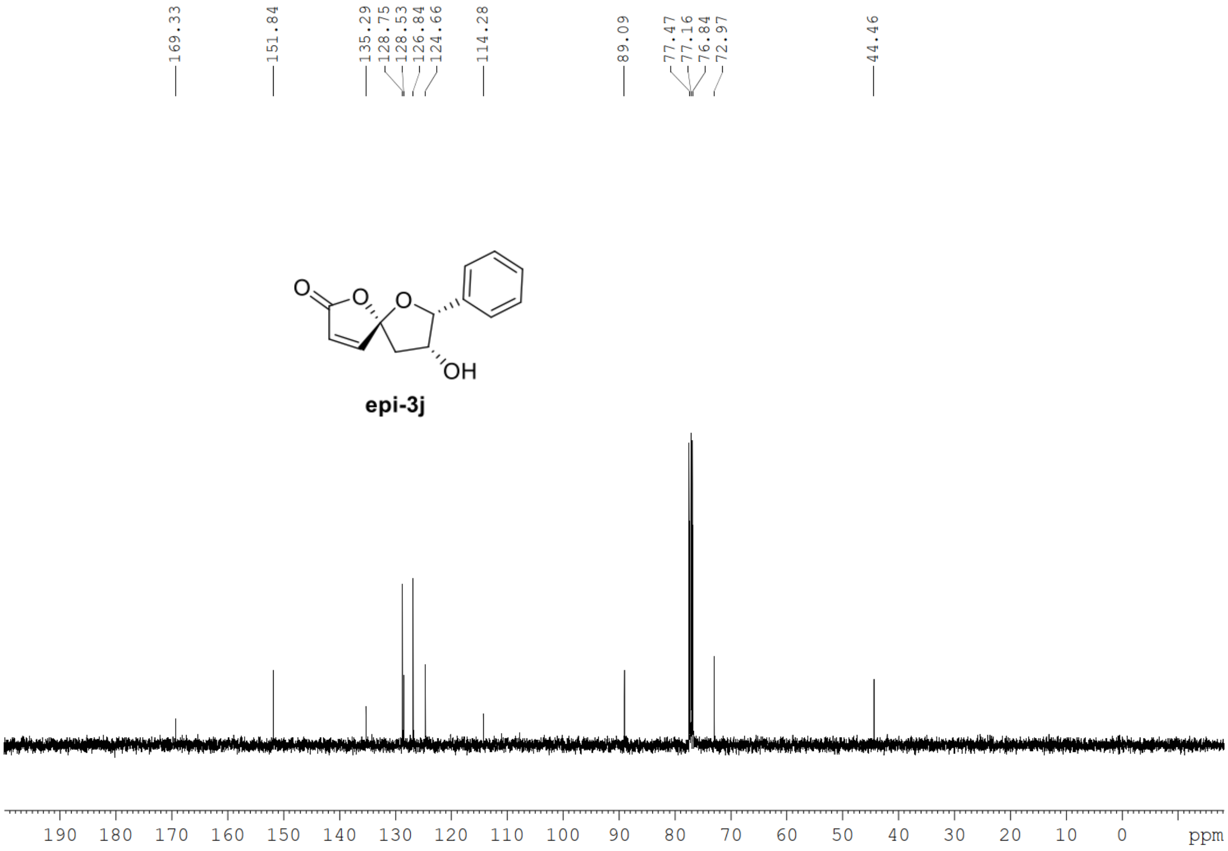
169.33
151.84
135.29 128.75 128.53 126.84 124.66
114.28
89.09
77.47 77.16 76.84 72.97
44.46
O O O OH
190 180 170 160 150 140 130 120 110 100 90 80 70 60 50 40 30 20 10 0 ppm

epi-3j

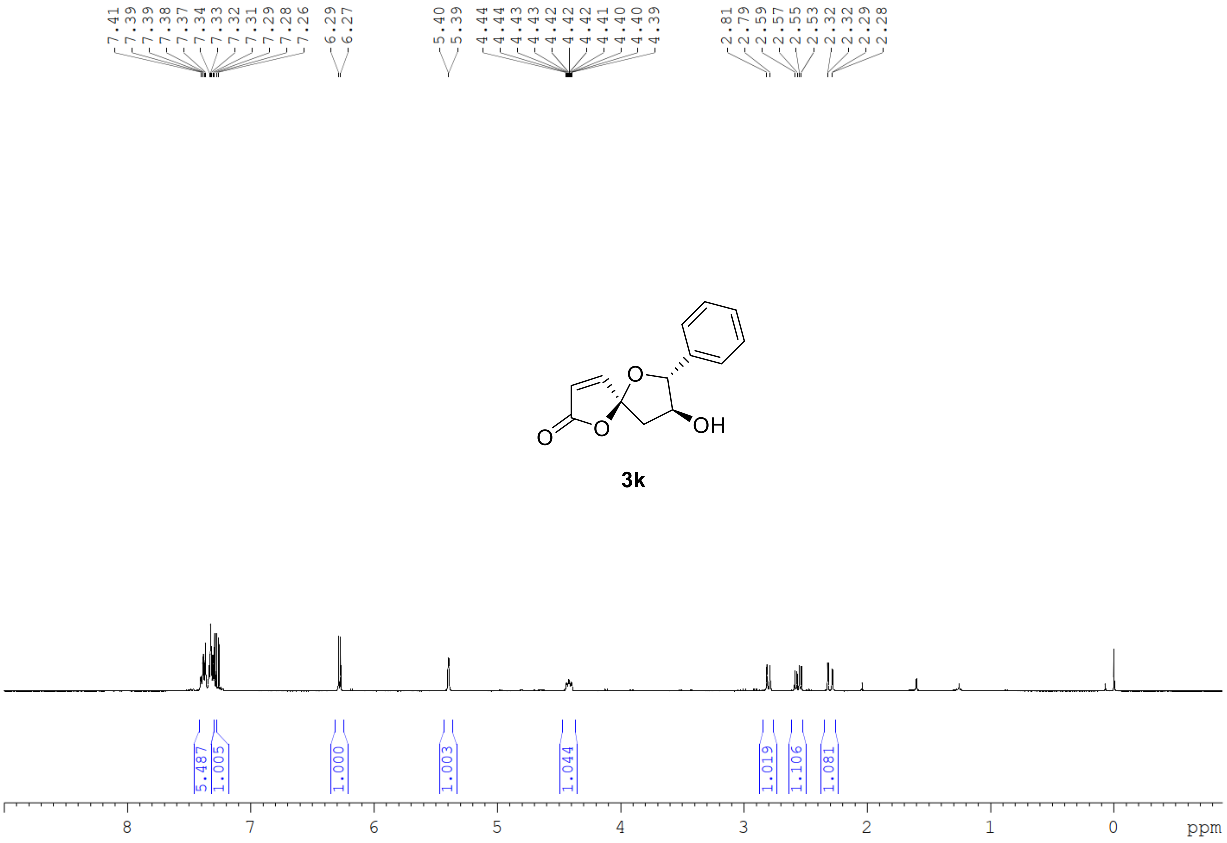
7.41
7.39
7.39
7.38
7.37
7.34
7.33
7.32
7.31
7.29
7.28
7.26
6.29
6.27
5.40
5.39
4.44
4.44
4.43
4.43
4.42
4.42
4.42
4.41
4.40
4.40
4.39
2.81
2.79
2.59
2.57
2.55
2.53
2.32
2.32
2.29
2.28
O
O
O
OH
3k
5.487
1.005
1.000
1.003
1.044
1.019
1.106
1.081
8
7
6
5
4
3
2
1
0
ppm

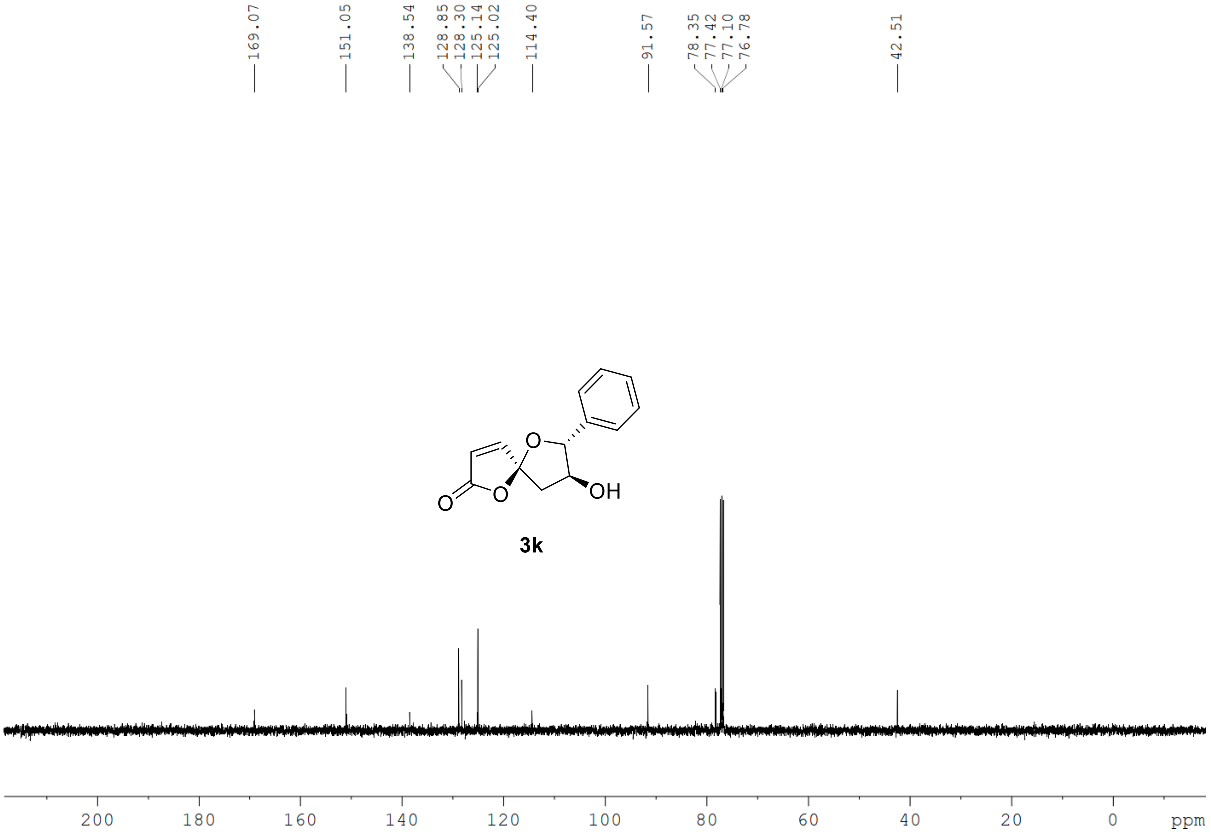
169.07
151.05
138.54
128.85
128.30
125.14
125.02
114.40
91.57
78.35
77.42
77.10
76.78
42.51
O
O
O
OH
3k
200
180
160
140
120
100
80
60
40
20
0
ppm

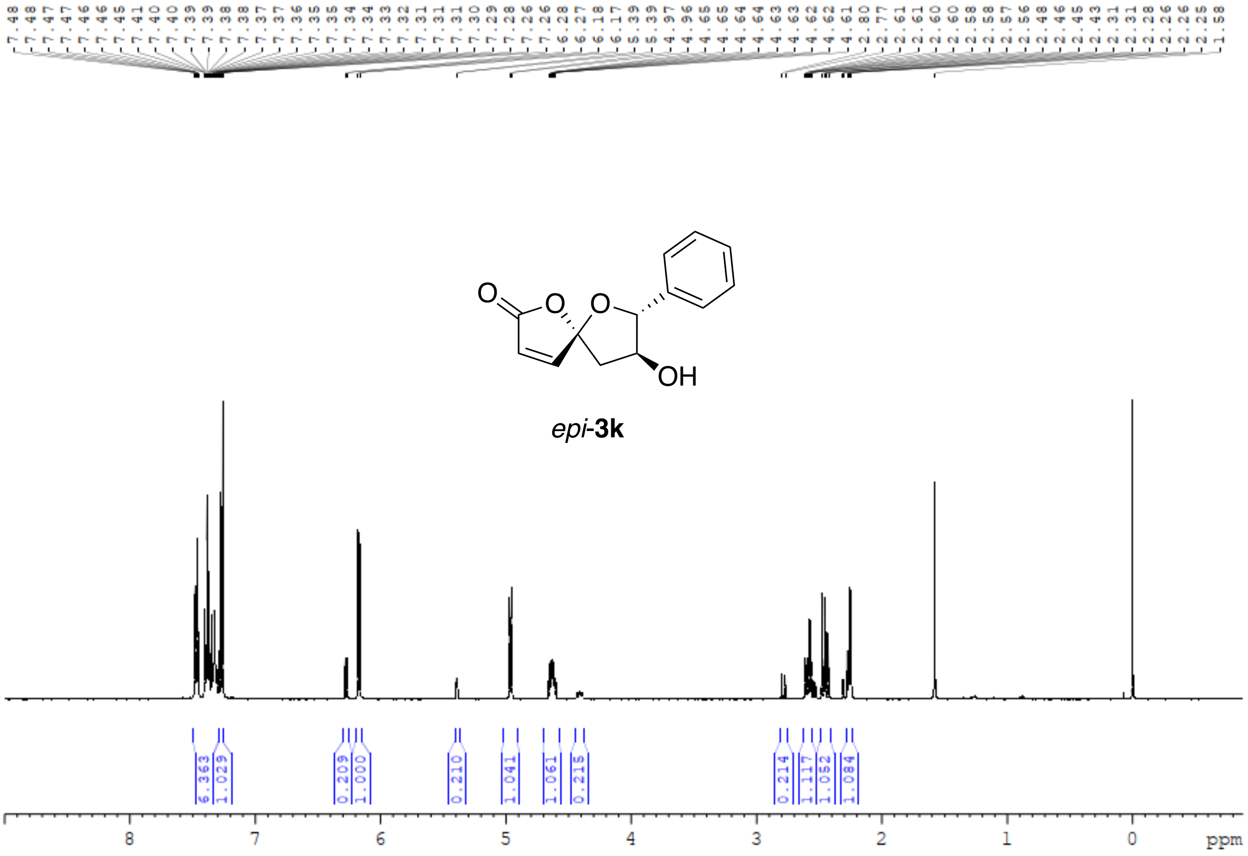
7.48 7.48 7.47 7.47 7.46 7.46 7.45 7.41 7.40 7.40 7.39 7.39 7.38 7.38 7.37 7.37 7.36 7.35 7.35 7.34 7.34 7.33 7.32 7.31 7.31 7.31 7.30 7.29 7.28 7.26 7.26 6.28 6.27 6.18 6.17 5.39 5.39 4.97 4.96 4.65 4.65 4.64 4.63 4.63 4.62 4.62 4.61 2.80 2.77 2.61 2.61 2.60 2.60 2.58 2.58 2.57 2.56 2.48 2.46 2.45 2.43 2.31 2.28 2.26 2.26 2.25 1.58
O
O
O
OH
epi-3k
6.363
1.029
0.209
1.000
0.210
1.041
1.061
0.215
0.214
1.117
1.052
1.084
8 7 6 5 4 3 2 1 0 ppm

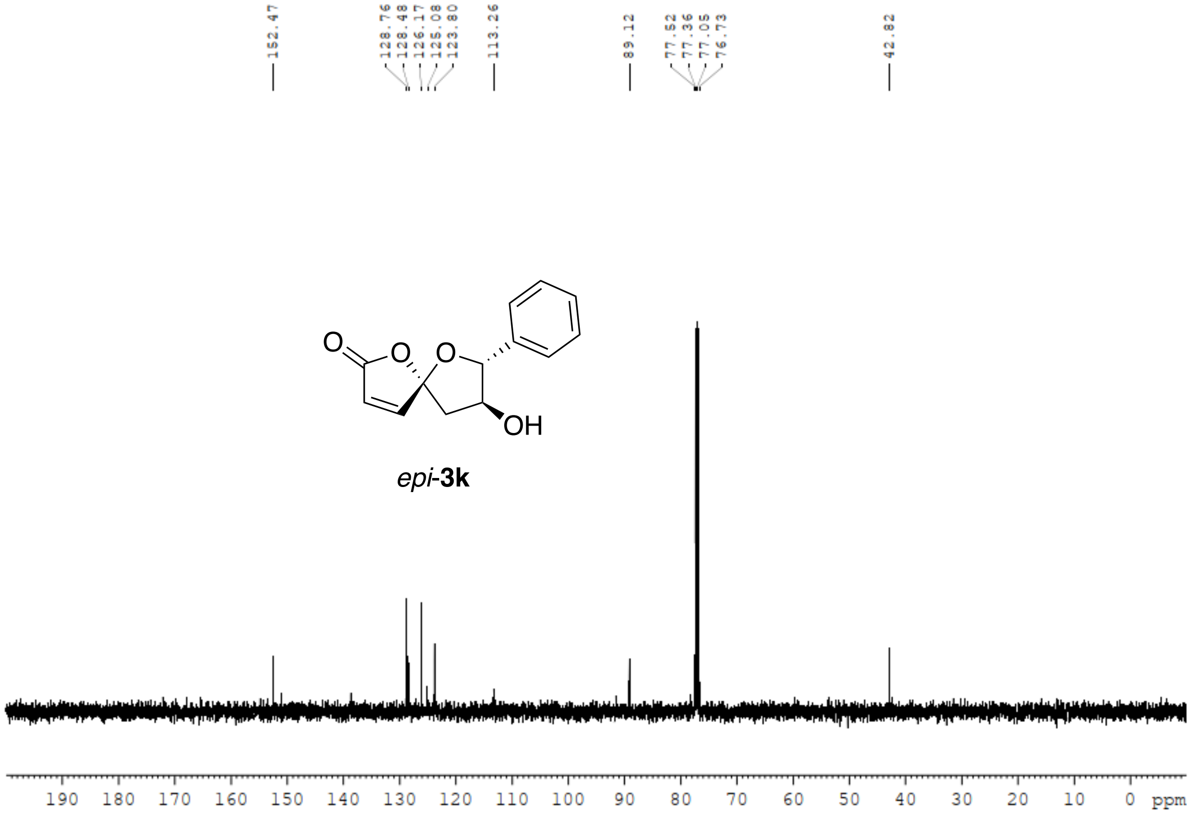
152.47
128.76
128.48
126.17
125.08
123.80
113.26
89.12
77.52
77.36
77.05
76.73
42.82
O
O
O
OH
epi-3k
190 180 170 160 150 140 130 120 110 100 90 80 70 60 50 40 30 20 10 0 ppm

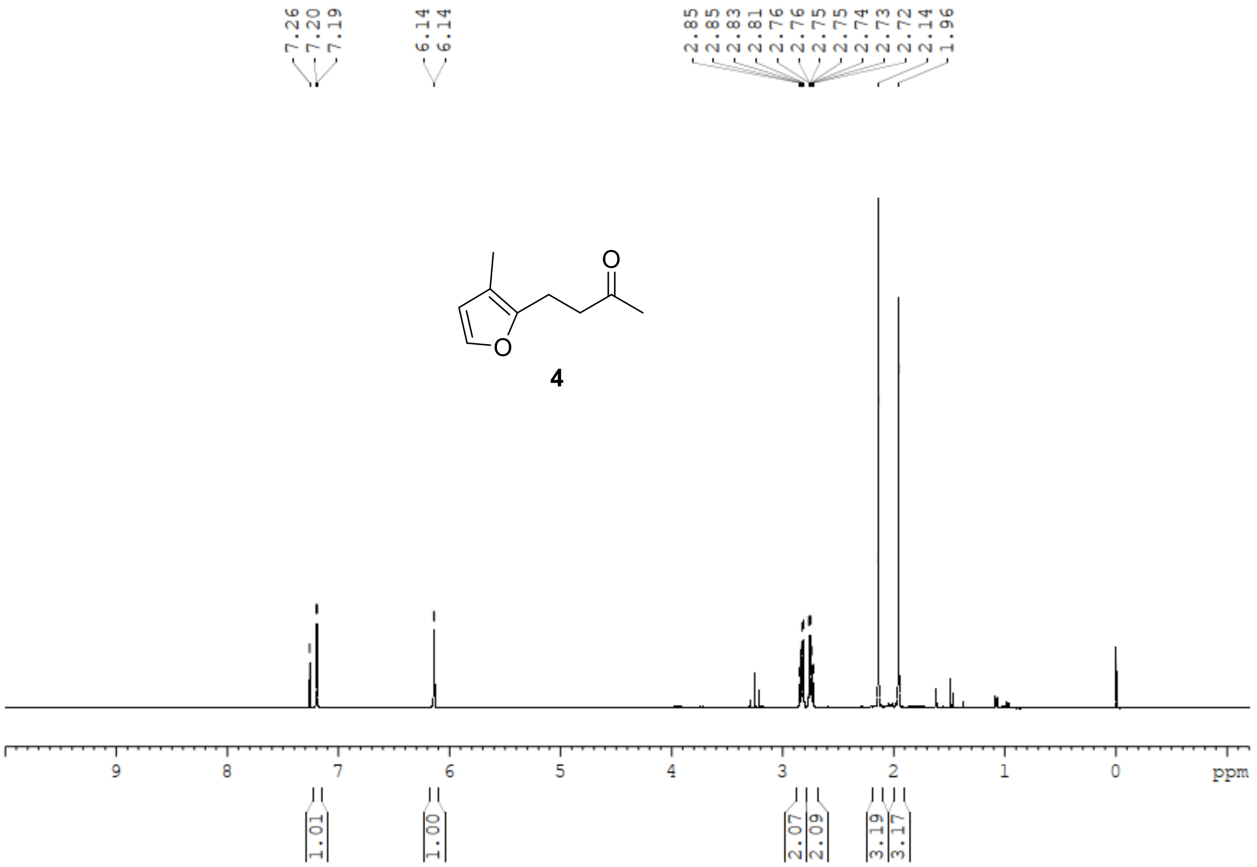
7.26
7.20
7.19
6.14
6.14
2.85
2.85
2.83
2.81
2.76
2.76
2.75
2.75
2.74
2.73
2.72
2.14
1.96
O
O
4
9
8
7
6
5
4
3
2
1
0
ppm
1.01
1.00
2.07
2.09
3.19
3.17

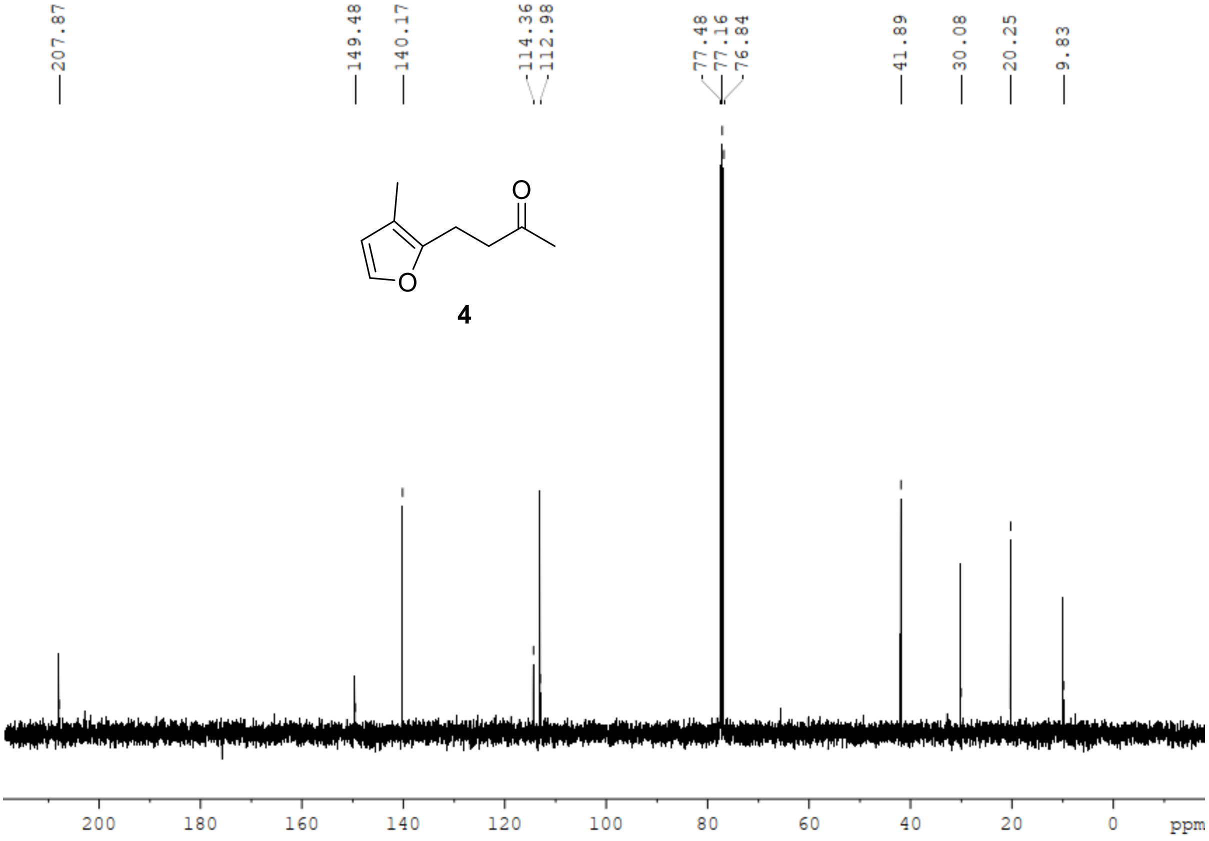
207.87
149.48
140.17
114.36
112.98
77.48
77.16
76.84
41.89
30.08
20.25
9.83
O
O
4
200
180
160
140
120
100
80
60
40
20
0
ppm

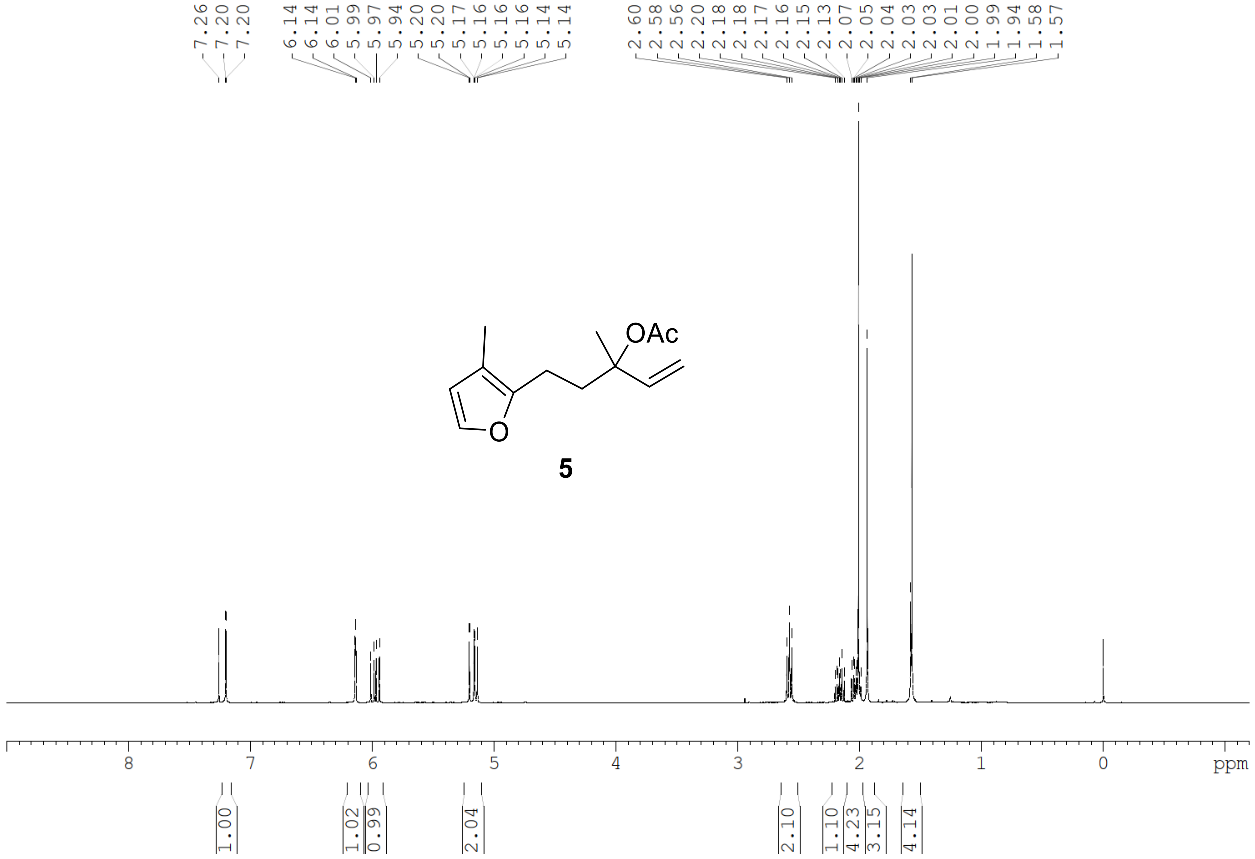
7.26
7.20
7.20
6.14
6.14
6.01
5.99
5.97
5.94
5.20
5.20
5.17
5.16
5.16
5.16
5.14
5.14
2.60
2.58
2.56
2.20
2.18
2.18
2.17
2.16
2.15
2.13
2.07
2.05
2.04
2.03
2.03
2.01
2.00
1.99
1.94
1.58
1.57
OAc
O
5
ppm
1.00
1.02
0.99
2.04
2.10
1.10
4.23
3.15
4.14

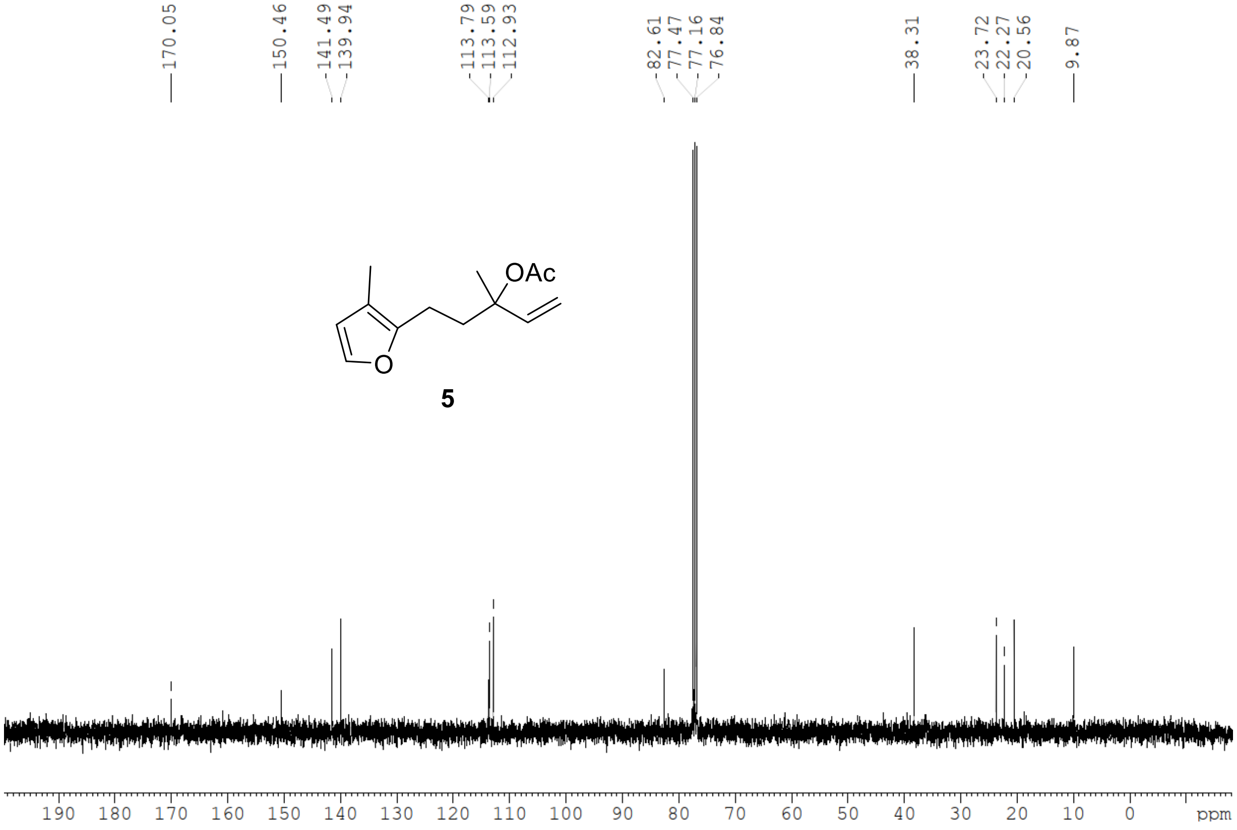
170.05
150.46
141.49
139.94
113.79
113.59
112.93
82.61
77.47
77.16
76.84
38.31
23.72
22.27
20.56
9.87
OAc
O
5
ppm

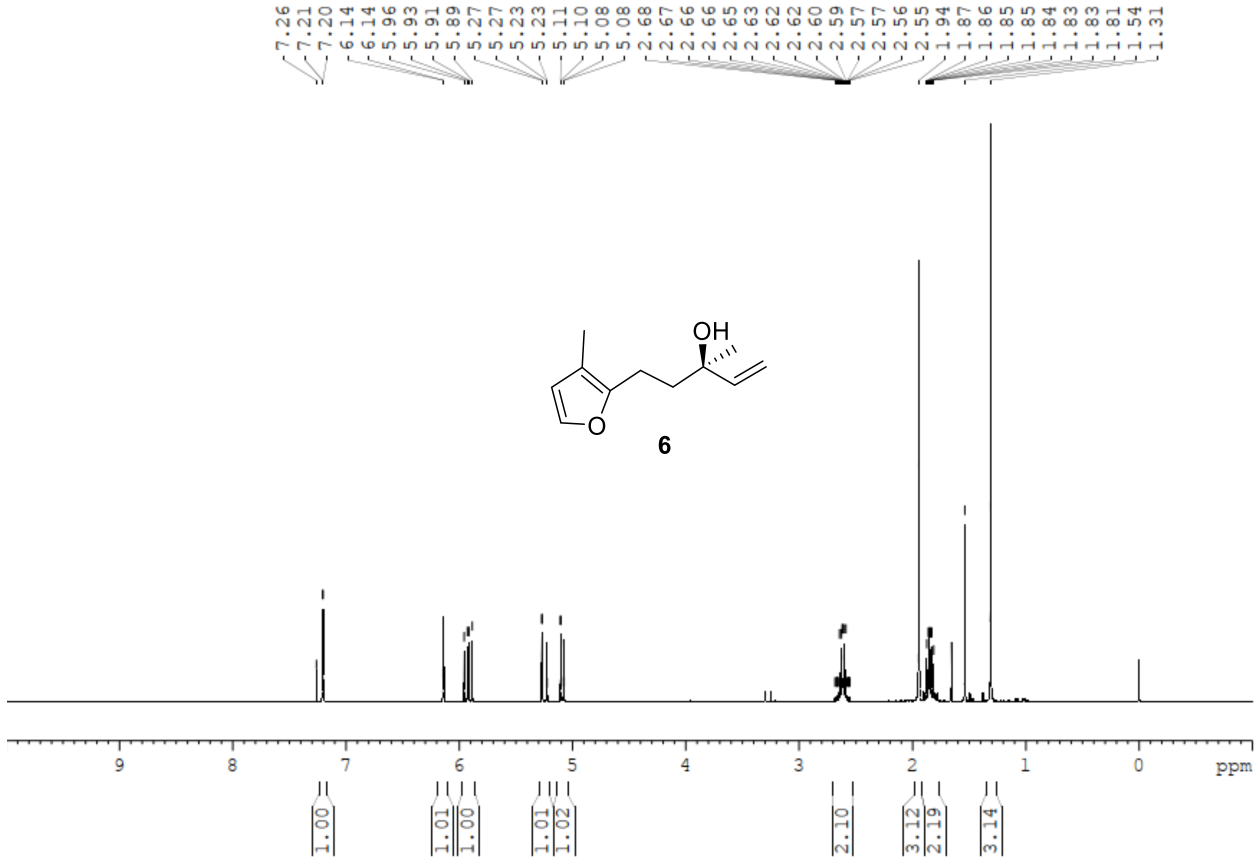
7.26 7.21 7.20 6.14 6.14 5.96 5.93 5.91 5.89 5.27 5.27 5.23 5.23 5.11 5.10 5.08 5.08 2.68 2.67 2.66 2.65 2.63 2.62 2.60 2.59 2.57 2.56 2.55 1.94 1.87 1.86 1.85 1.85 1.84 1.83 1.81 1.54 1.31
OH
O
6
9 8 7 6 5 4 3 2 1 0 ppm
1.00 1.01 1.00 1.01 1.02 2.10 3.12 2.19 3.14

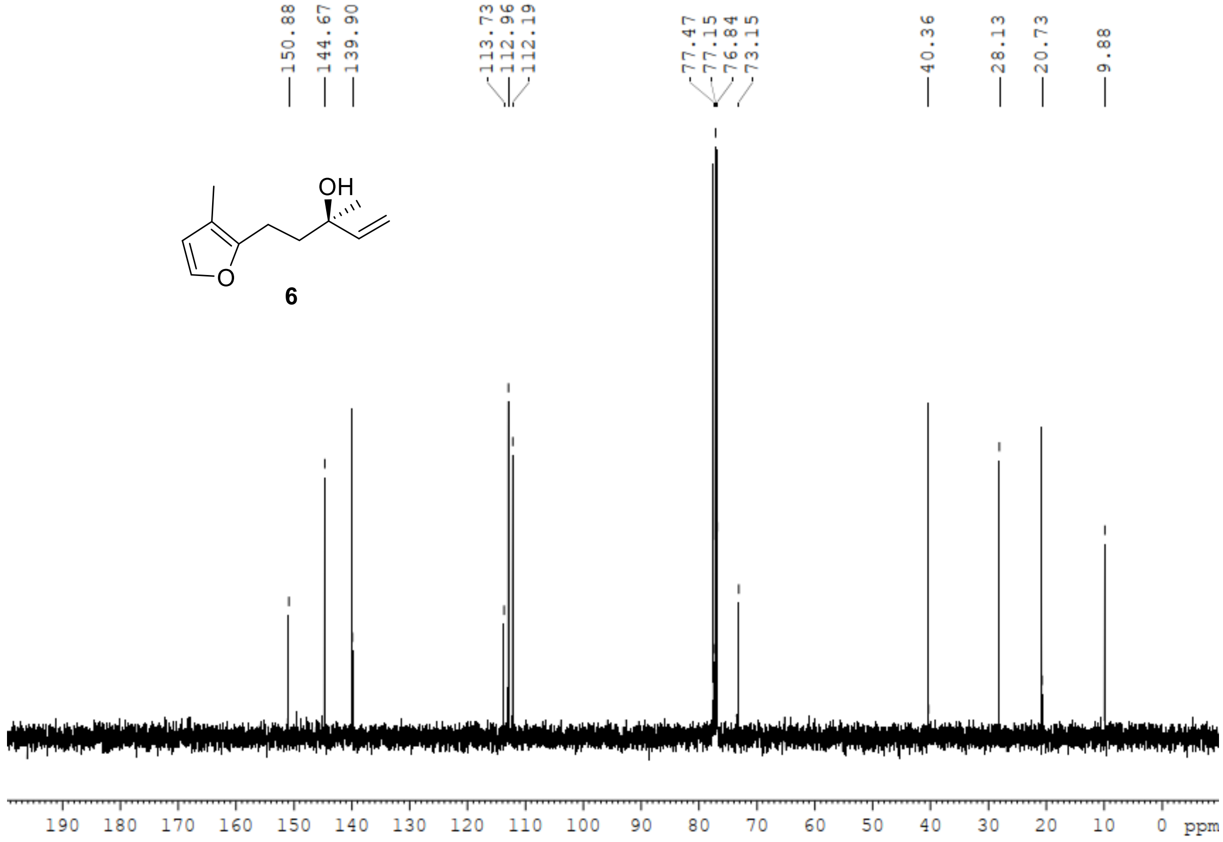
150.88 144.67 139.90 113.73 112.96 112.19 77.47 77.15 76.84 73.15 40.36 28.13 20.73 9.88
OH
O
6
190 180 170 160 150 140 130 120 110 100 90 80 70 60 50 40 30 20 10 0 ppm

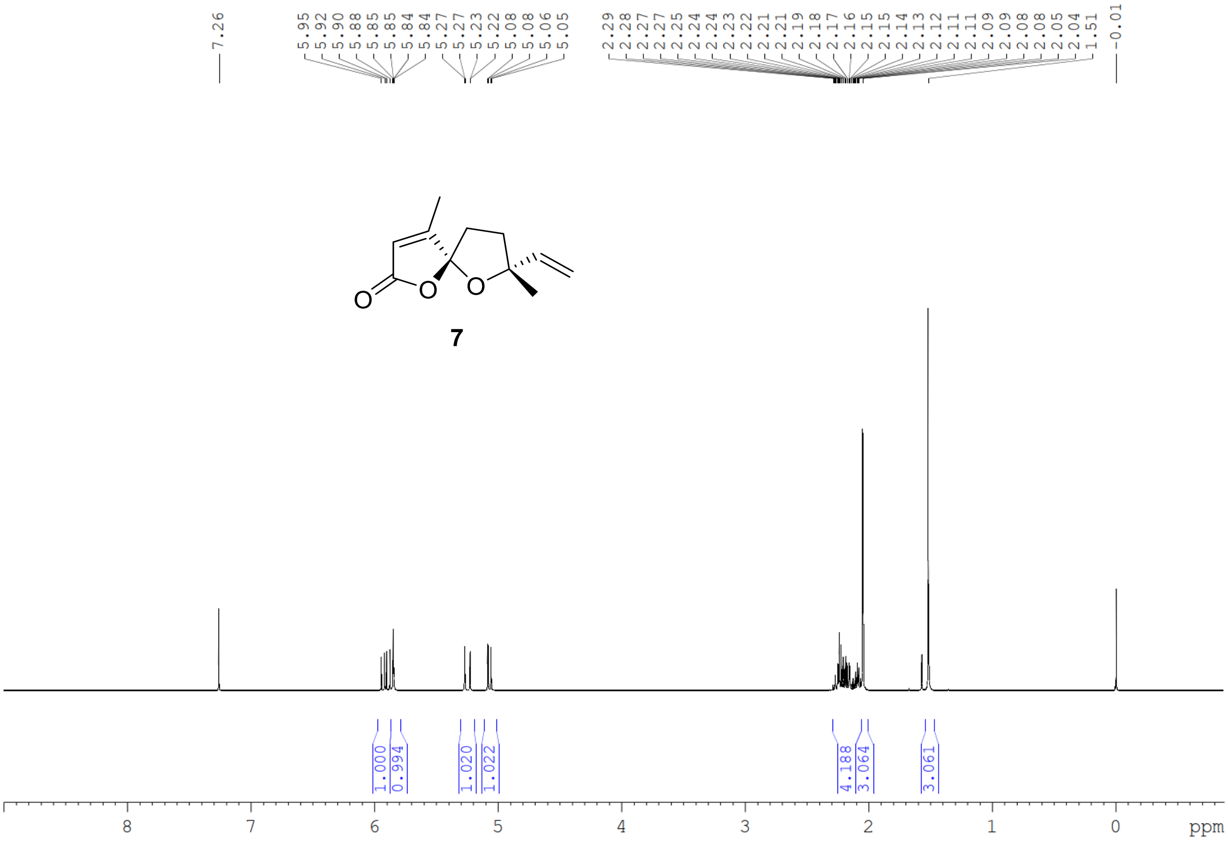
7.26
5.95 5.92 5.90 5.88 5.85 5.85 5.84 5.84 5.27 5.27 5.23 5.22 5.08 5.08 5.06 5.05
2.29 2.28 2.27 2.27 2.25 2.24 2.24 2.23 2.22 2.21 2.21 2.19 2.18 2.17 2.16 2.15 2.15 2.14 2.13 2.12 2.11 2.11 2.09 2.09 2.08 2.08 2.05 2.04 1.51
-0.01
O
O
O
7
1.000
0.994
1.020
1.022
4.188
3.064
3.061
8 7 6 5 4 3 2 1 0 ppm

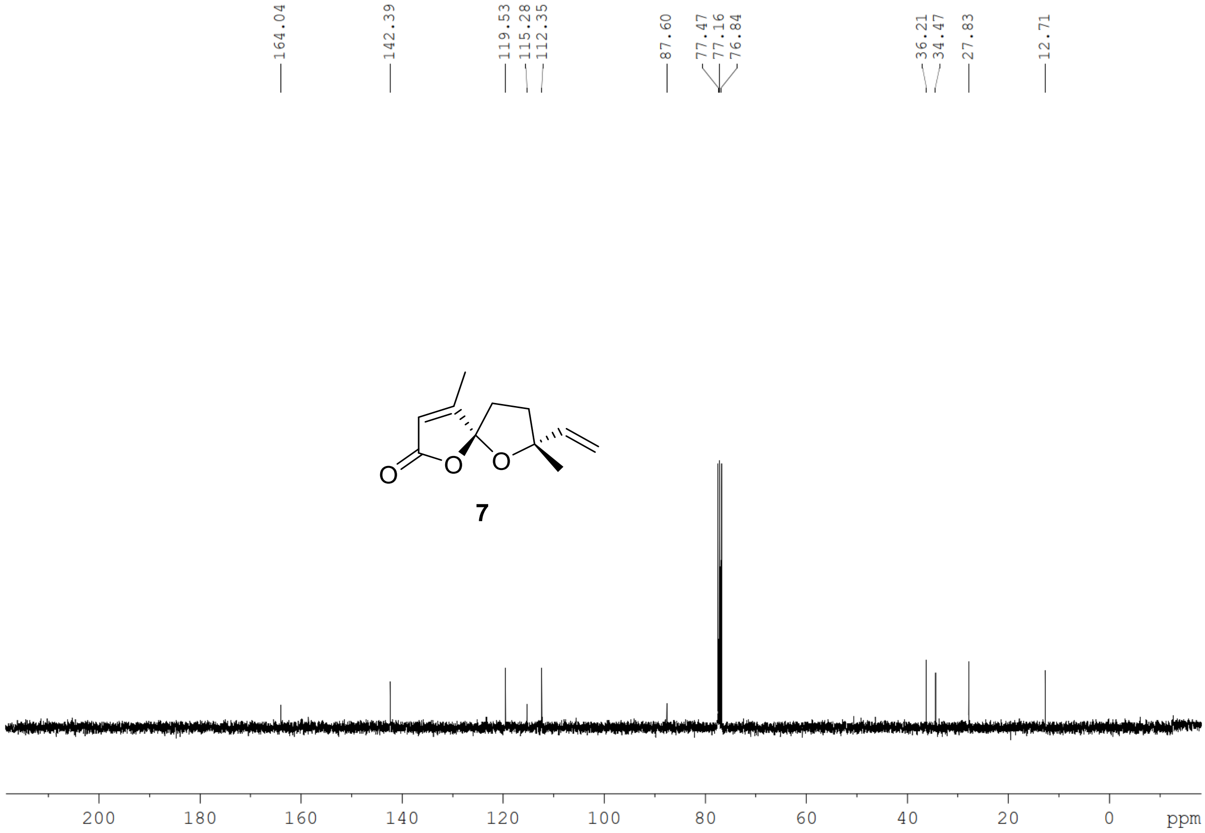
164.04
142.39
119.53 115.28 112.35
87.60
77.47 77.16 76.84
36.21 34.47
27.83
12.71
O
O
O
7
200 180 160 140 120 100 80 60 40 20 0 ppm

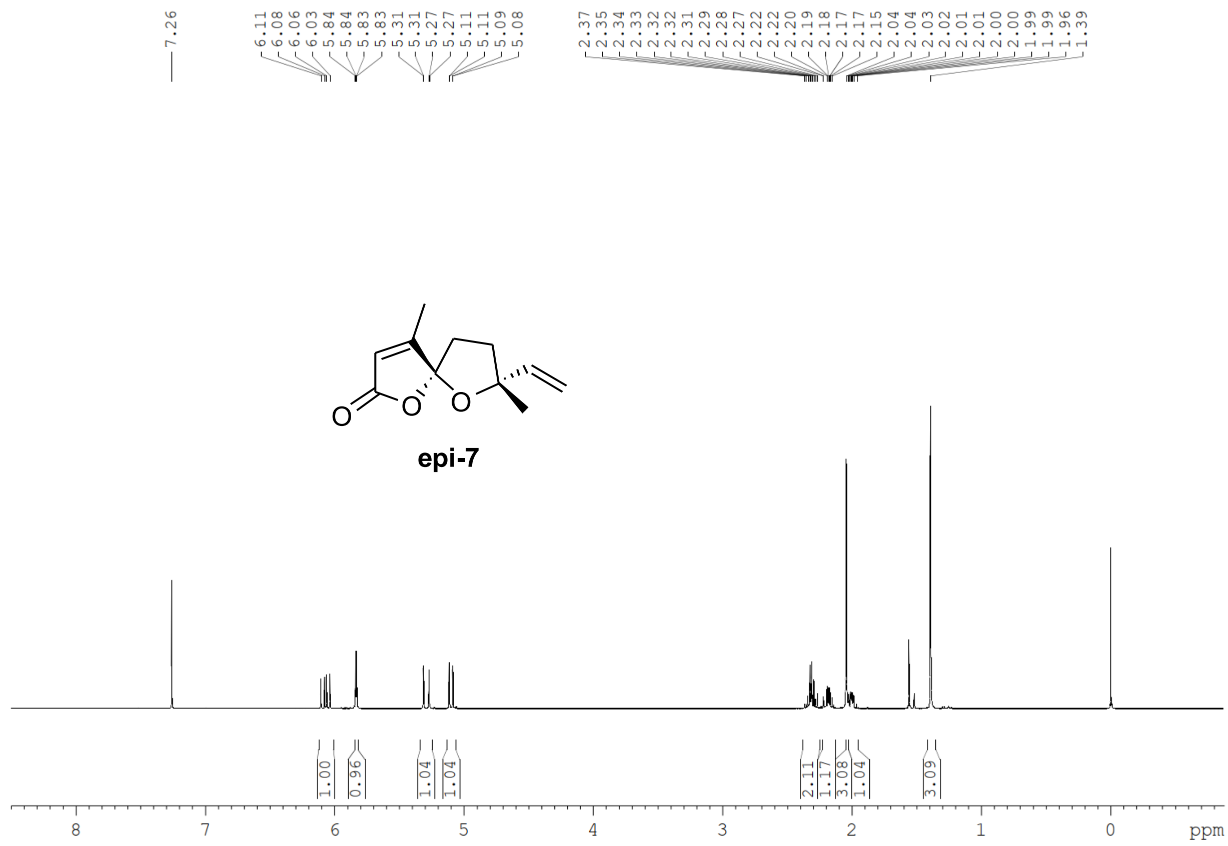
7.26
6.11 6.08 6.06 6.03 5.84 5.84 5.83 5.83 5.31 5.31 5.27 5.27 5.11 5.11 5.09 5.08
2.37 2.35 2.34 2.33 2.32 2.32 2.31 2.29 2.28 2.27 2.22 2.22 2.20 2.19 2.18 2.17 2.17 2.15 2.04 2.04 2.03 2.02 2.01 2.01 2.00 2.00 1.99 1.99 1.96 1.39
epi-7
1.00 0.96 1.04 1.04 2.11 1.17 3.08 1.04 3.09
8 7 6 5 4 3 2 1 0 ppm

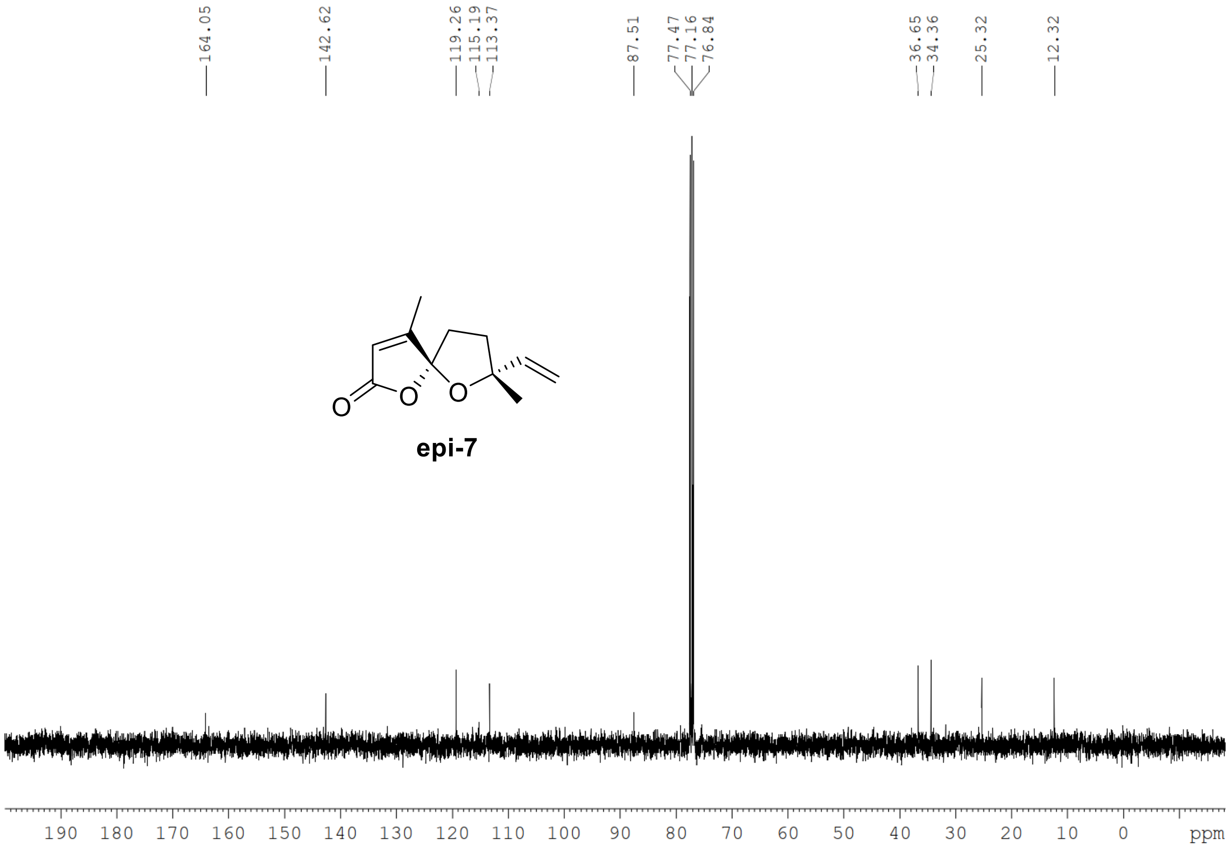
164.05
142.62
119.26 115.19 113.37
87.51
77.47 77.16 76.84
36.65 34.36
25.32
12.32
epi-7
190 180 170 160 150 140 130 120 110 100 90 80 70 60 50 40 30 20 10 0 ppm

6.2 NMR spectra of the substrates

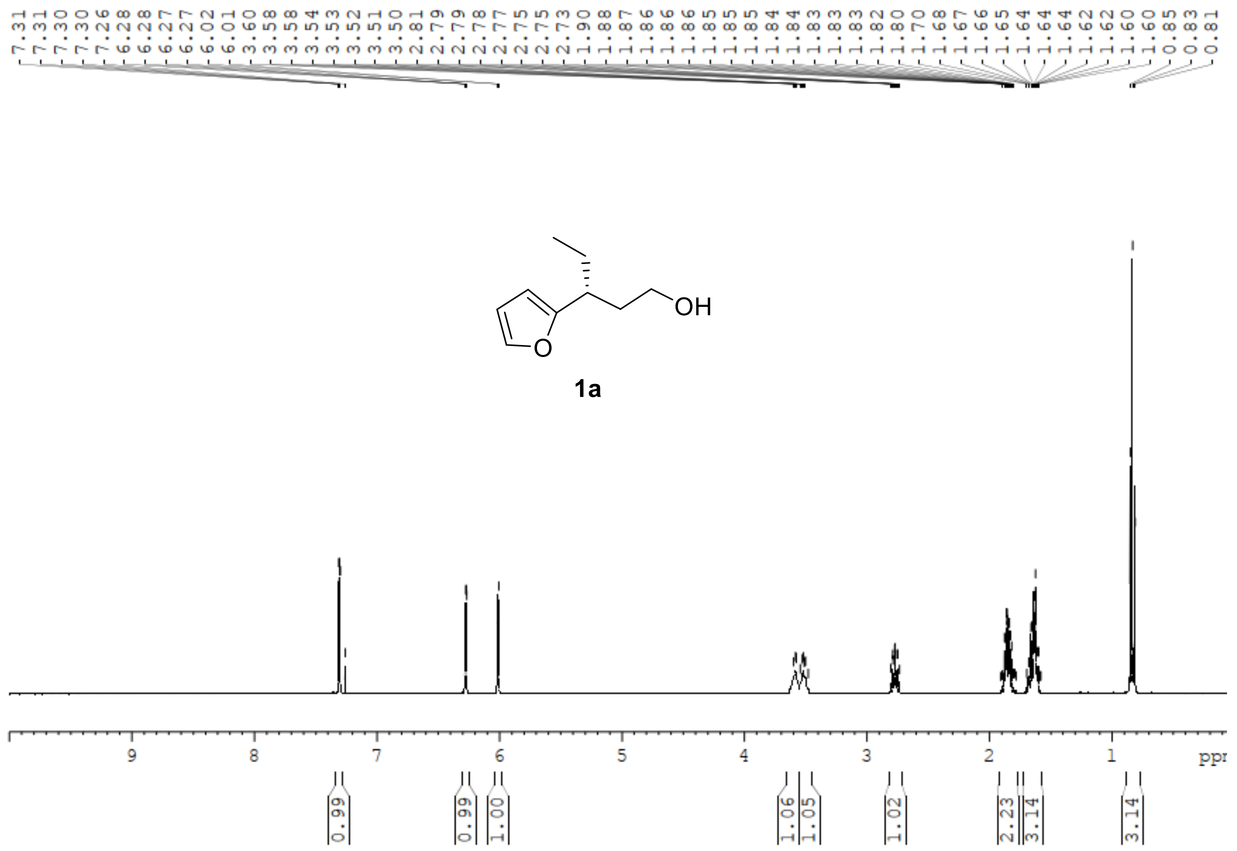

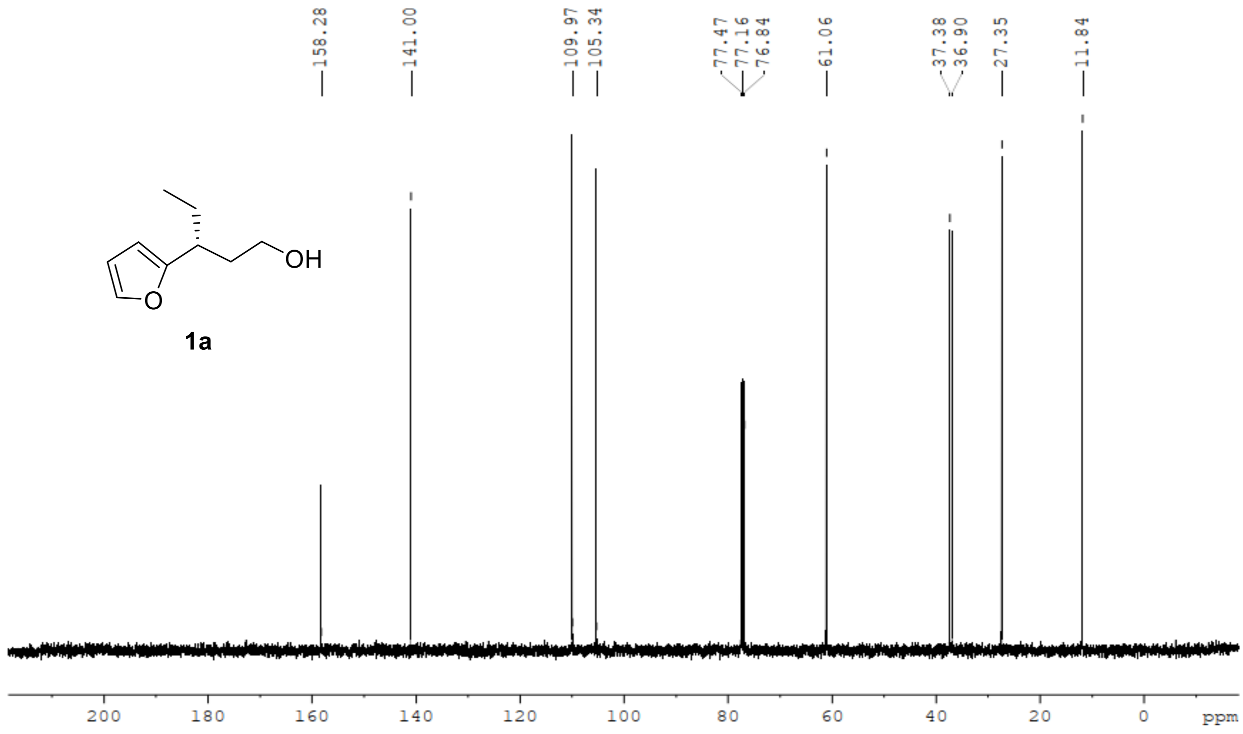

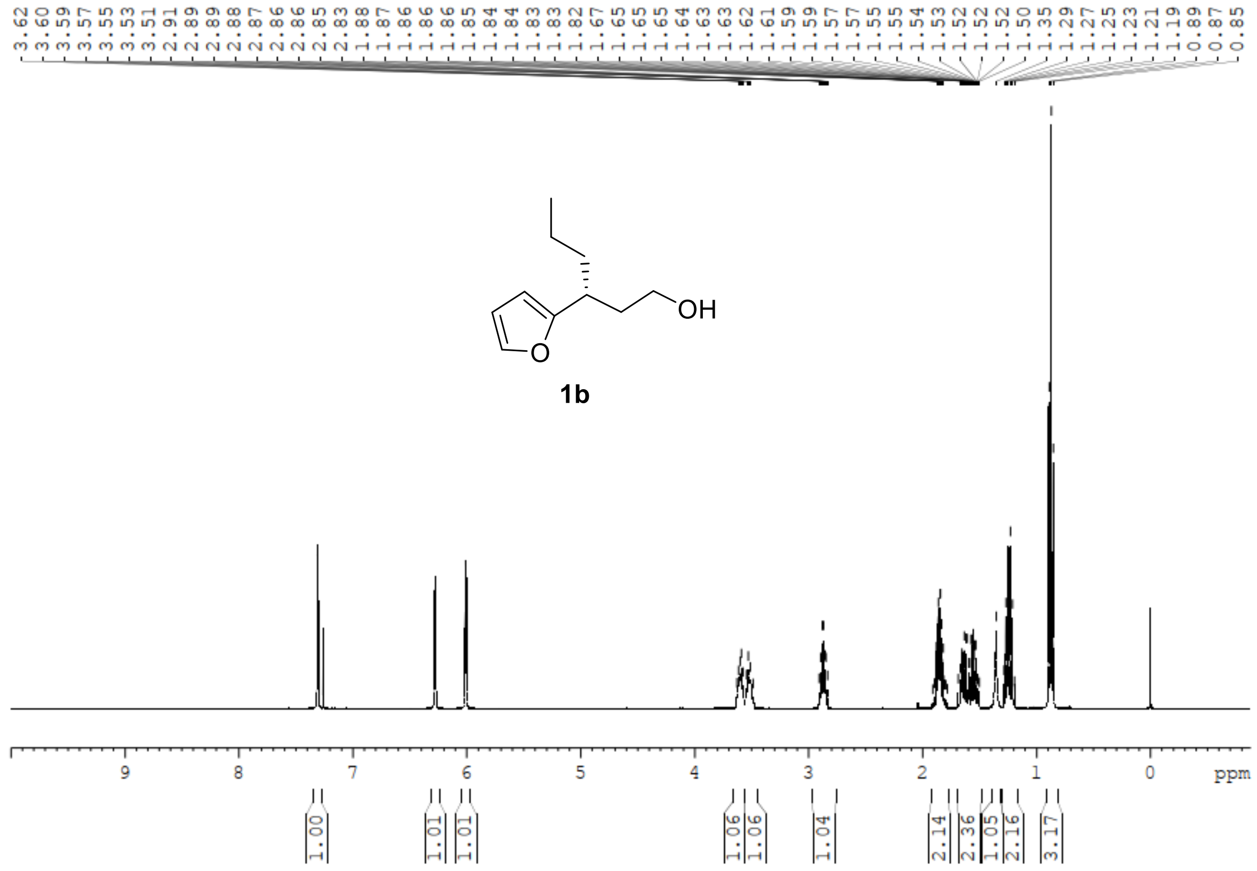
3.62 3.60 3.59 3.57 3.55 3.53 3.51 2.91 2.89 2.88 2.87 2.86 2.86 2.85 2.83 1.88 1.87 1.86 1.86 1.86 1.85 1.84 1.84 1.83 1.83 1.82 1.67 1.65 1.65 1.65 1.64 1.63 1.63 1.62 1.61 1.59 1.59 1.57 1.57 1.55 1.55 1.54 1.53 1.52 1.52 1.52 1.50 1.35 1.29 1.27 1.25 1.23 1.21 1.19 0.89 0.87 0.85
OH
O
1b
9 8 7 6 5 4 3 2 1 0 ppm
1.00 1.01 1.01 1.06 1.06 1.04 2.14 2.36 1.05 2.16 3.17

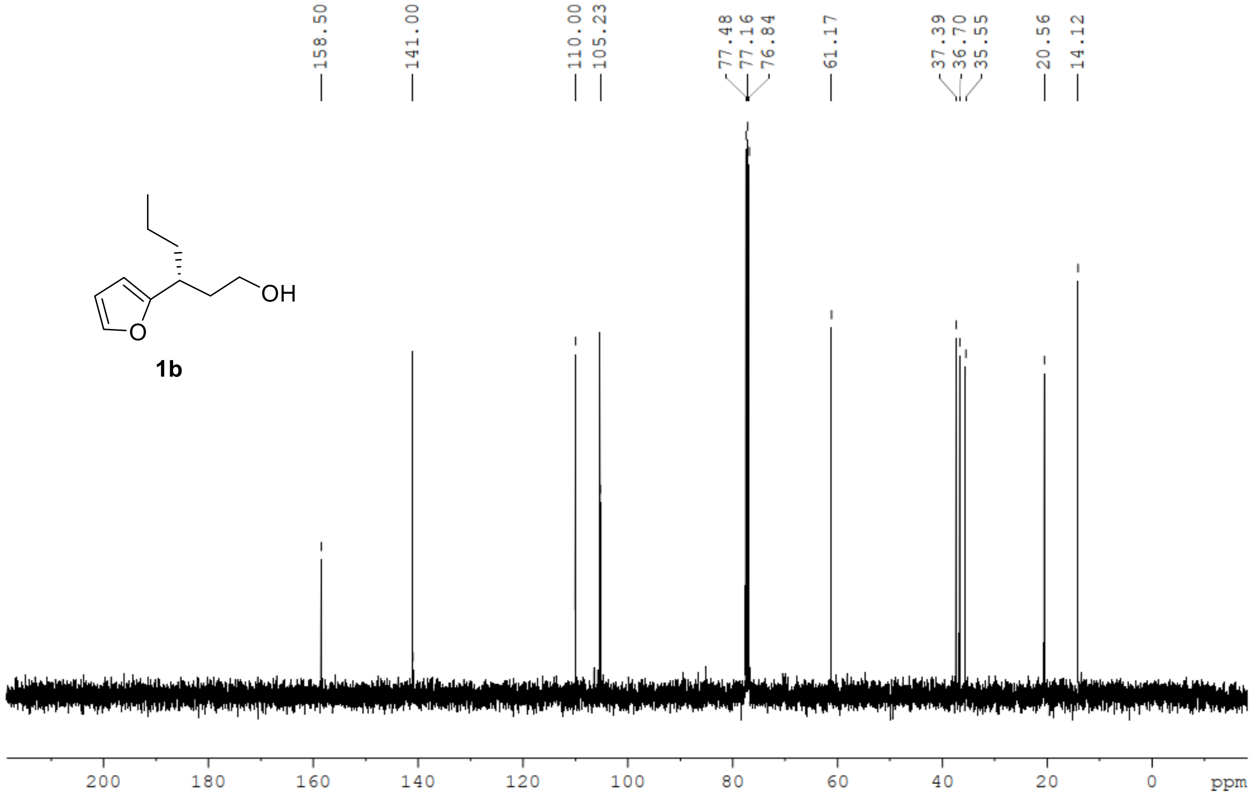
158.50 141.00 110.00 105.23 77.48 77.16 76.84 61.17 37.39 36.70 35.55 20.56 14.12
OH
O
1b
200 180 160 140 120 100 80 60 40 20 0 ppm

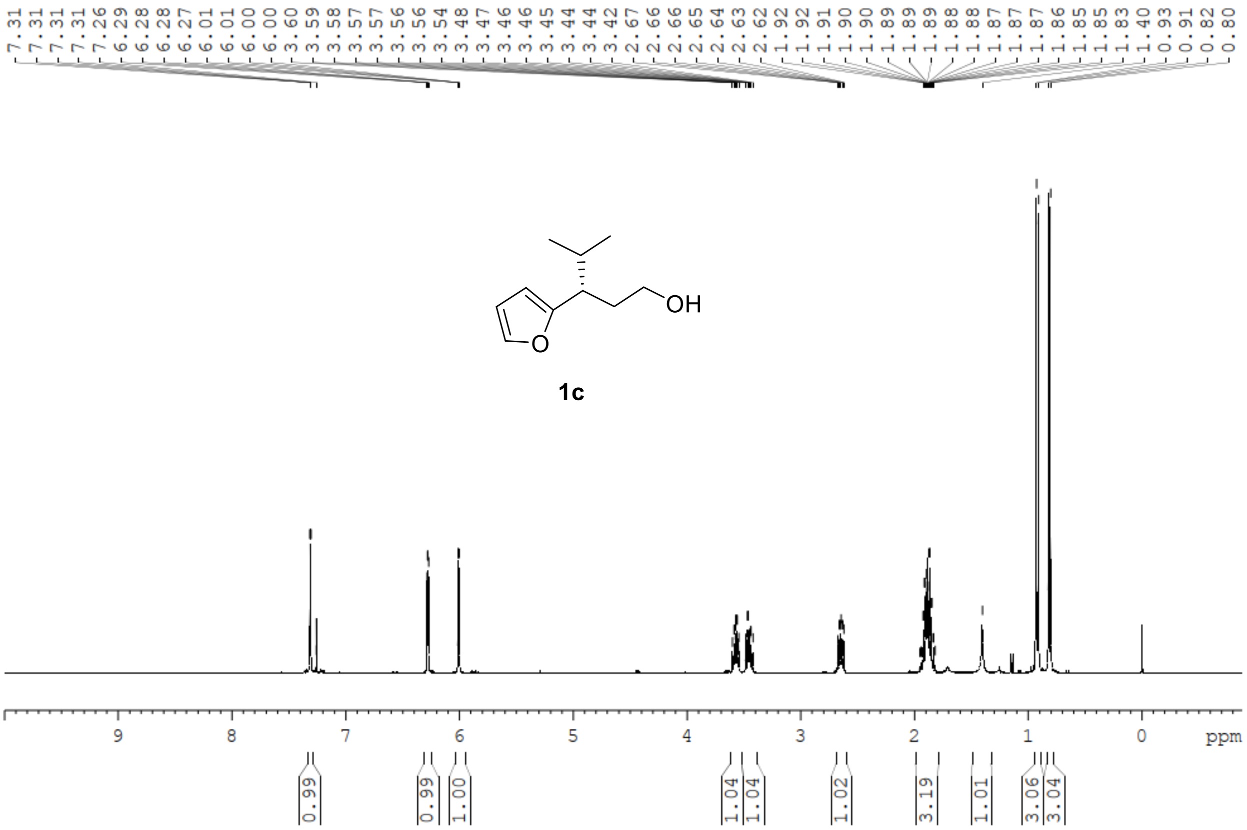
7.31
7.31
7.31
7.31
7.26
6.29
6.28
6.28
6.27
6.01
6.01
6.00
6.00
3.60
3.59
3.58
3.57
3.57
3.56
3.56
3.54
3.48
3.47
3.46
3.46
3.45
3.44
3.44
3.42
2.67
2.66
2.66
2.65
2.64
2.63
2.62
1.92
1.91
1.90
1.90
1.89
1.89
1.89
1.88
1.87
1.87
1.87
1.86
1.85
1.85
1.83
1.40
0.93
0.91
0.82
0.80
OH
O
1c
9
8
7
6
5
4
3
2
1
0
ppm
0.99
0.99
1.00
1.04
1.04
1.02
3.19
1.01
3.06
3.04

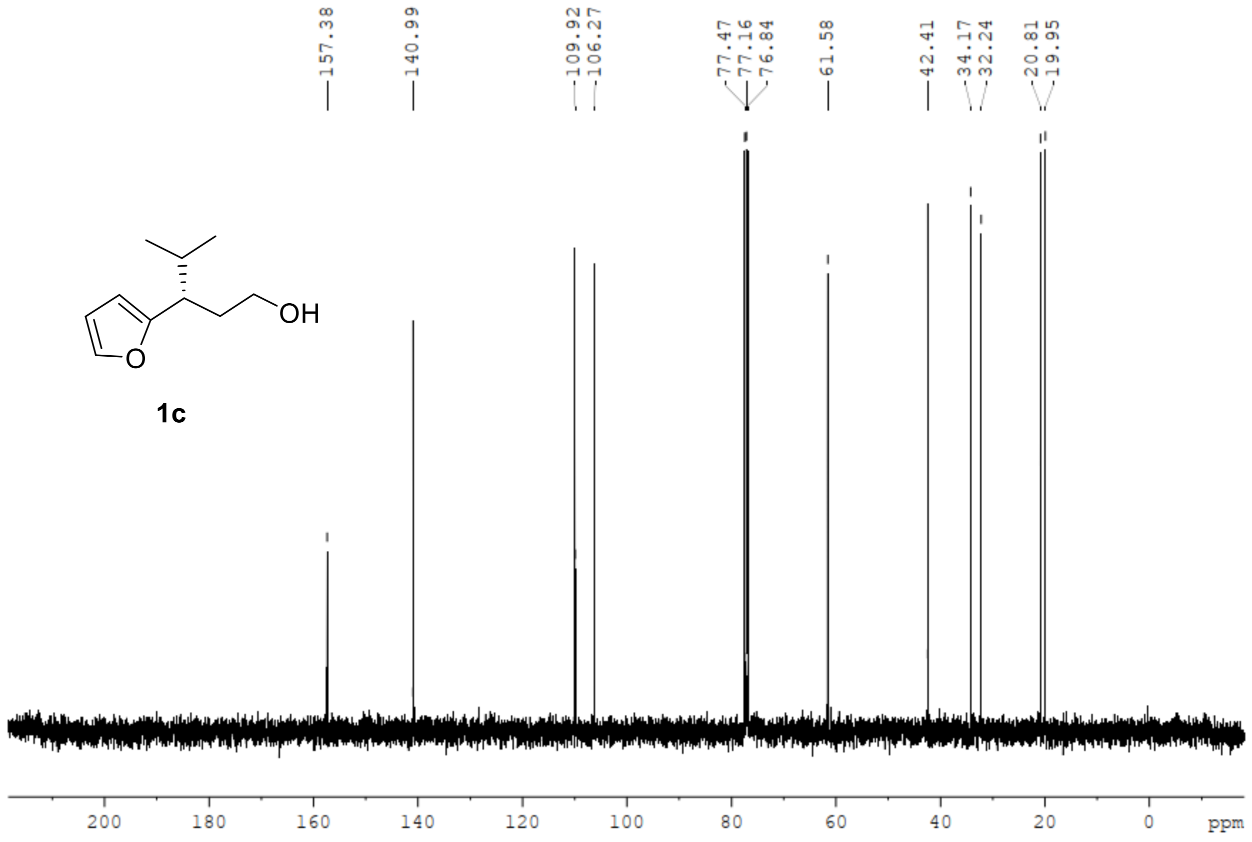
157.38
140.99
109.92
106.27
77.47
77.16
76.84
61.58
42.41
34.17
32.24
20.81
19.95
OH
O
1c
200
180
160
140
120
100
80
60
40
20
0
ppm

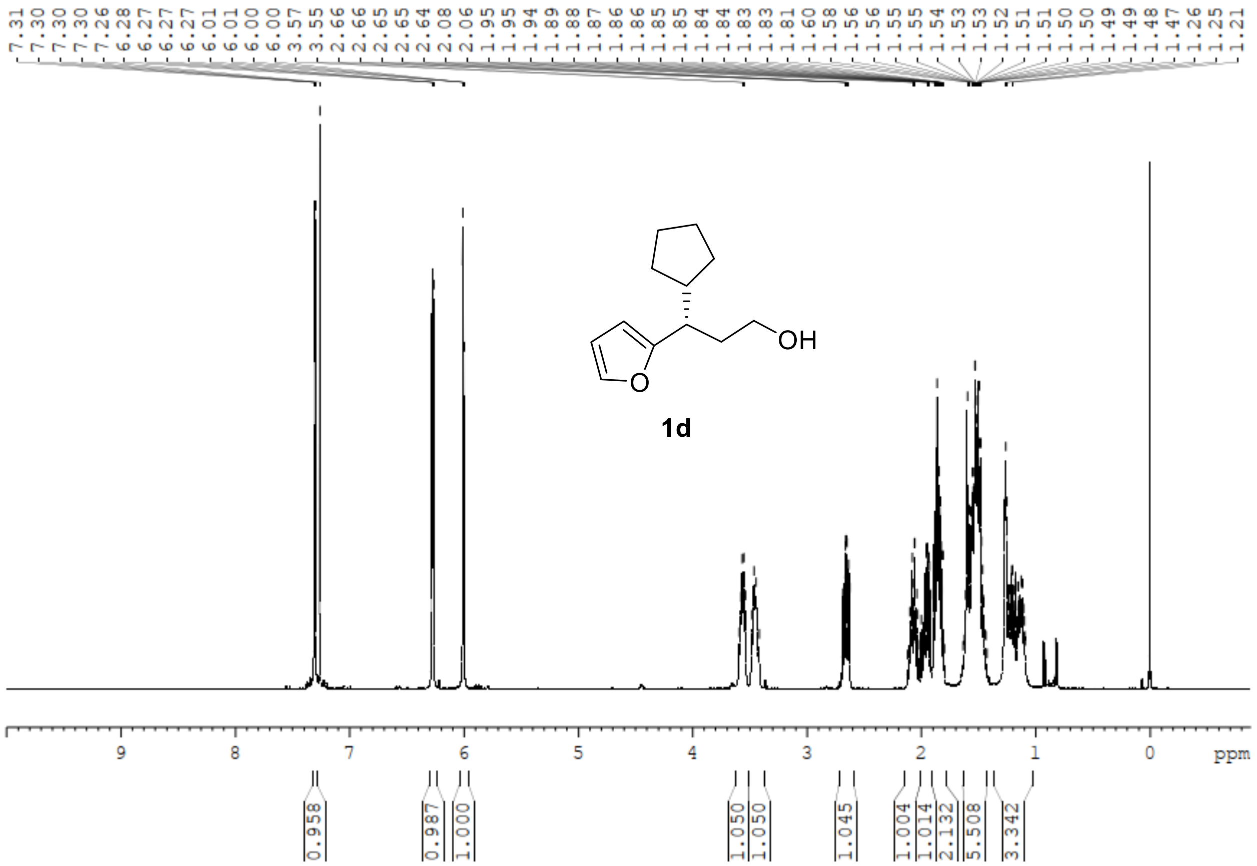
7.31 7.30 7.30 7.26 6.28 6.27 6.27 6.01 6.01 6.00 6.00 3.57 3.55 2.66 2.66 2.65 2.64 2.08 2.06 1.95 1.95 1.94 1.89 1.87 1.86 1.86 1.85 1.85 1.84 1.84 1.83 1.83 1.81 1.60 1.58 1.56 1.56 1.55 1.55 1.54 1.53 1.53 1.52 1.51 1.51 1.50 1.50 1.49 1.49 1.48 1.47 1.26 1.25 1.21
OH
O
1d
9 8 7 6 5 4 3 2 1 0 ppm
0.958 0.987 1.000 1.050 1.050 1.045 1.004 1.014 2.132 5.508 3.342

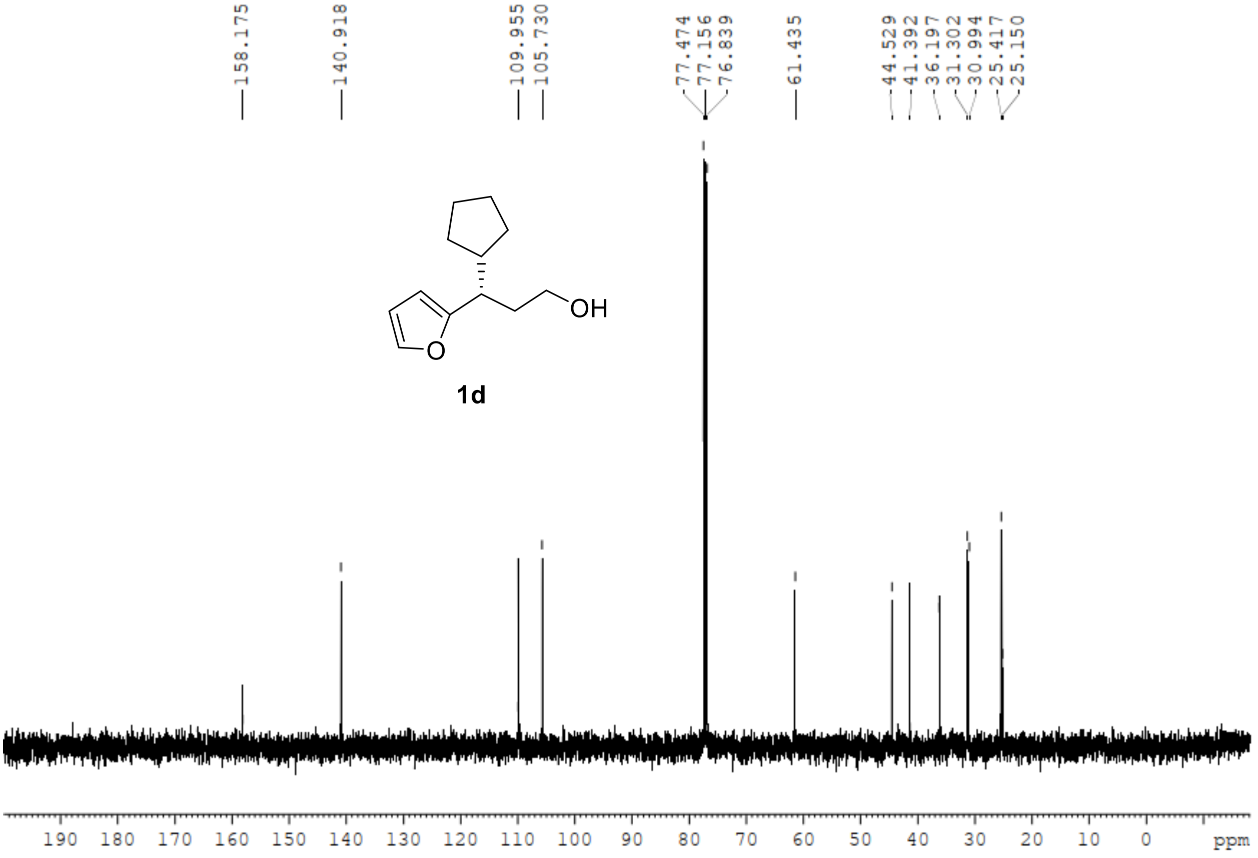
158.175 140.918 109.955 105.730 77.474 77.156 76.839 61.435 44.529 41.392 36.197 31.302 30.994 25.417 25.150
OH
O
1d
190 180 170 160 150 140 130 120 110 100 90 80 70 60 50 40 30 20 10 0 ppm

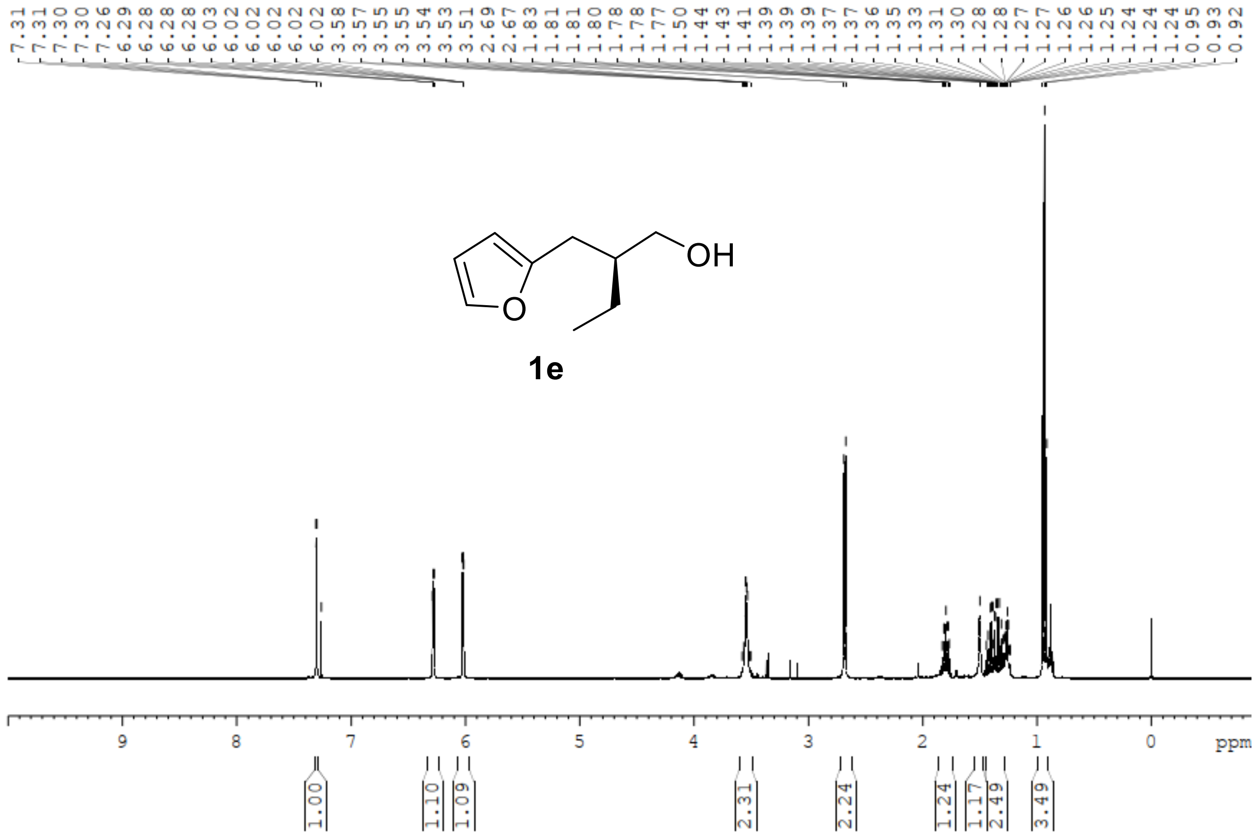
7.31 7.31 7.30 7.30 7.26 6.29 6.28 6.28 6.03 6.02 6.02 6.02 6.02 6.02 3.58 3.57 3.55 3.55 3.54 3.53 3.51 2.69 2.67 1.83 1.81 1.81 1.80 1.78 1.78 1.77 1.50 1.44 1.43 1.41 1.39 1.39 1.37 1.37 1.36 1.35 1.33 1.31 1.30 1.28 1.28 1.27 1.27 1.26 1.25 1.24 1.24 1.24 0.95 0.93 0.92
OH
O
1e
9 8 7 6 5 4 3 2 1 0 ppm
1.00 1.10 1.09 2.31 2.24 1.24 1.17 2.49 3.49

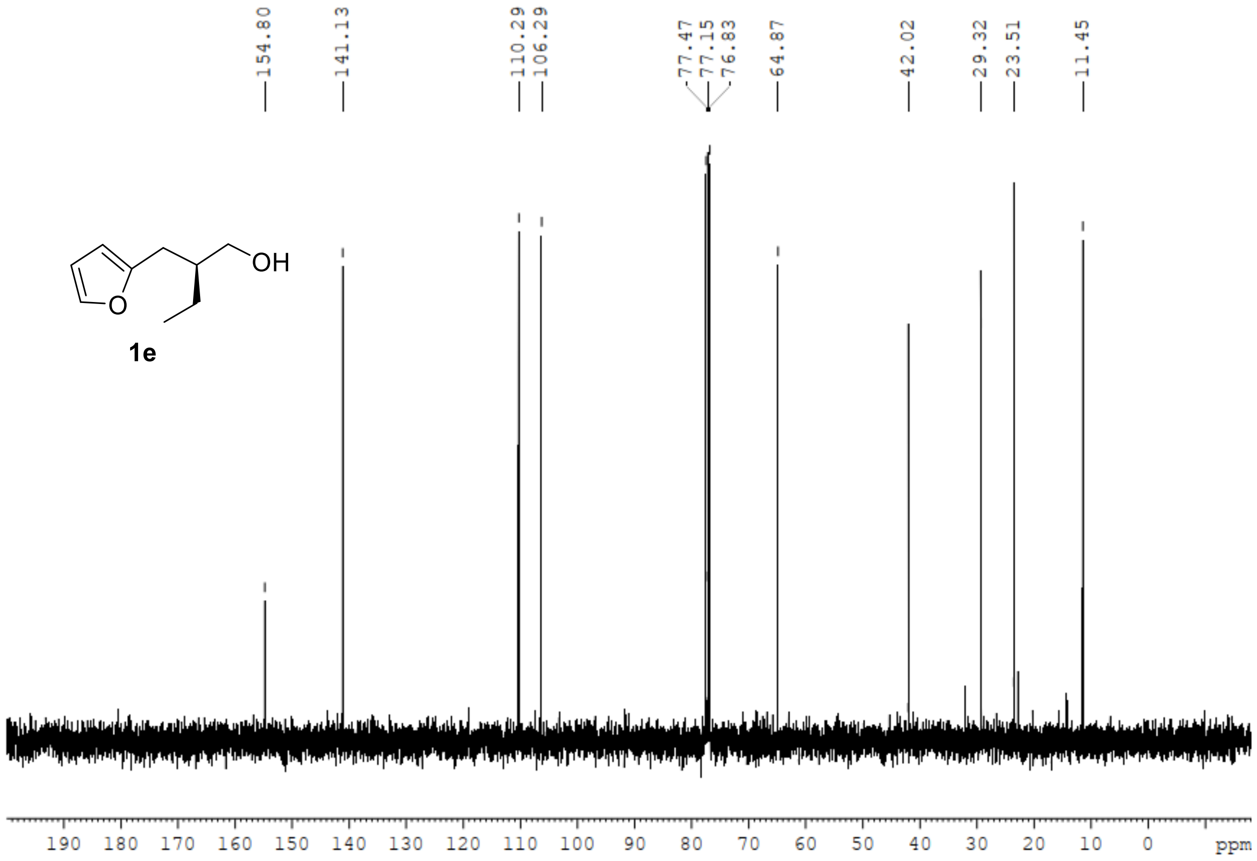
154.80 141.13 110.29 106.29 77.47 77.15 76.83 64.87 42.02 29.32 23.51 11.45
OH
O
1e
190 180 170 160 150 140 130 120 110 100 90 80 70 60 50 40 30 20 10 0 ppm

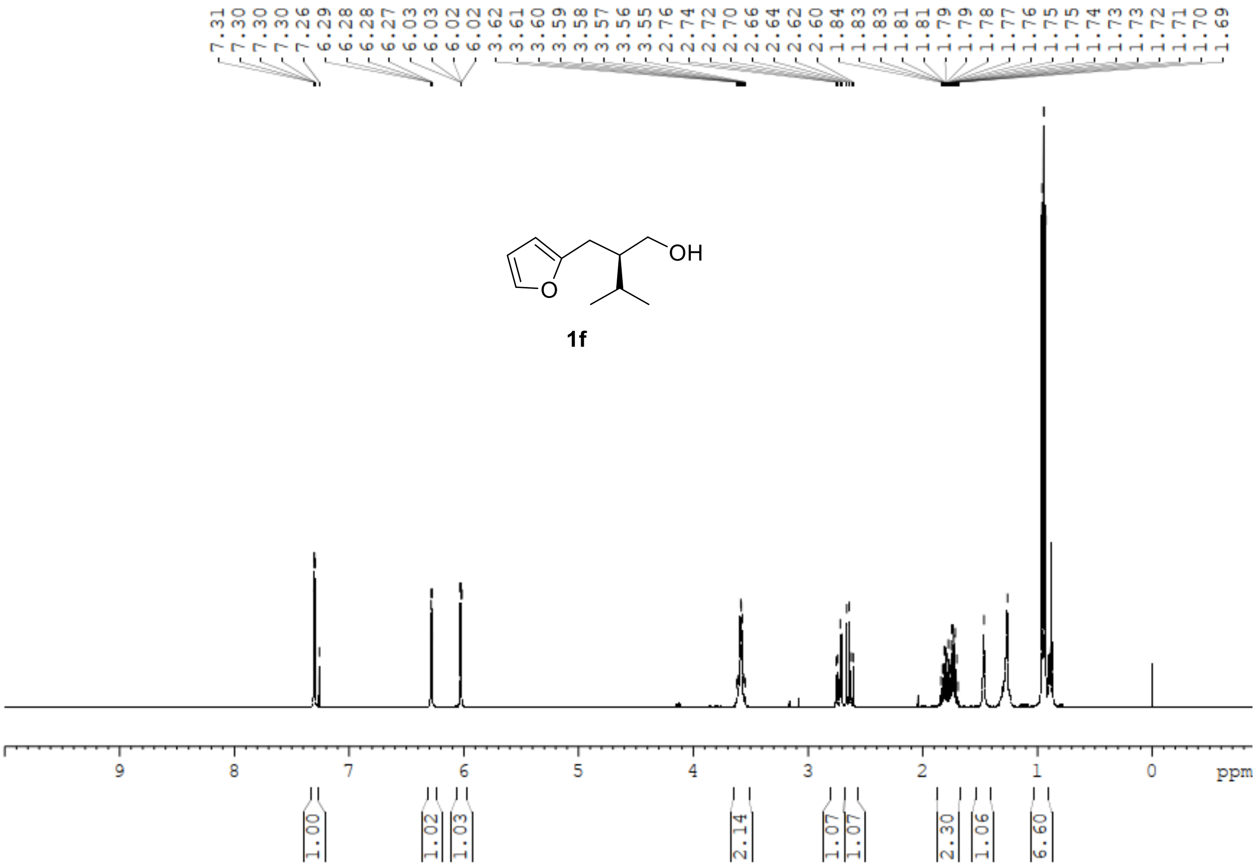
7.31 7.30 7.30 7.30 7.26 6.29 6.28 6.28 6.27 6.03 6.03 6.02 6.02 3.62 3.61 3.60 3.59 3.58 3.57 3.56 3.55 2.76 2.74 2.72 2.70 2.66 2.64 2.62 2.60 1.84 1.83 1.83 1.81 1.81 1.79 1.79 1.78 1.77 1.76 1.75 1.75 1.74 1.73 1.73 1.72 1.71 1.70 1.69
OH
O
1f
9 8 7 6 5 4 3 2 1 0 ppm
1.00 1.02 1.03 2.14 1.07 1.07 2.30 1.06 6.60

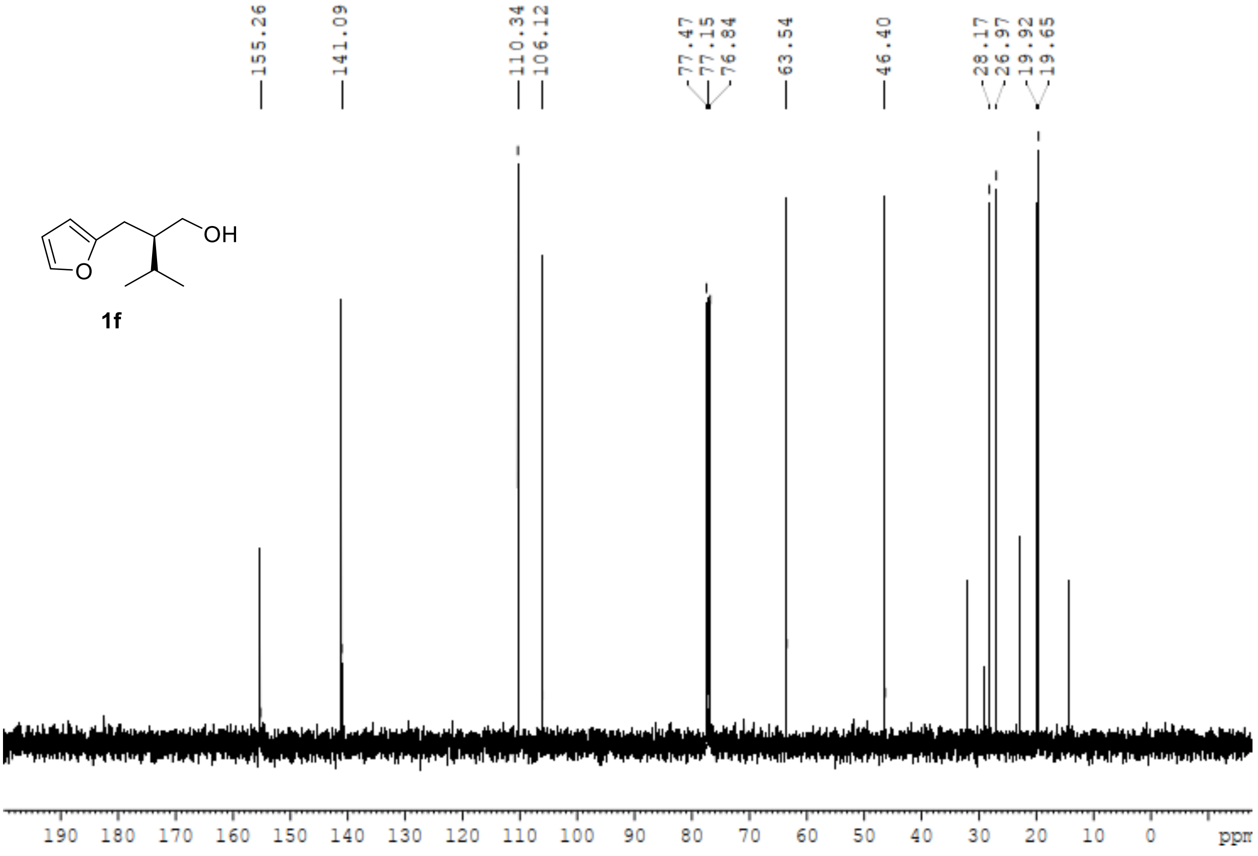
155.26 141.09 110.34 106.12 77.47 77.15 76.84 63.54 46.40 28.17 26.97 19.92 19.65
OH
O
1f
190 180 170 160 150 140 130 120 110 100 90 80 70 60 50 40 30 20 10 0 ppm

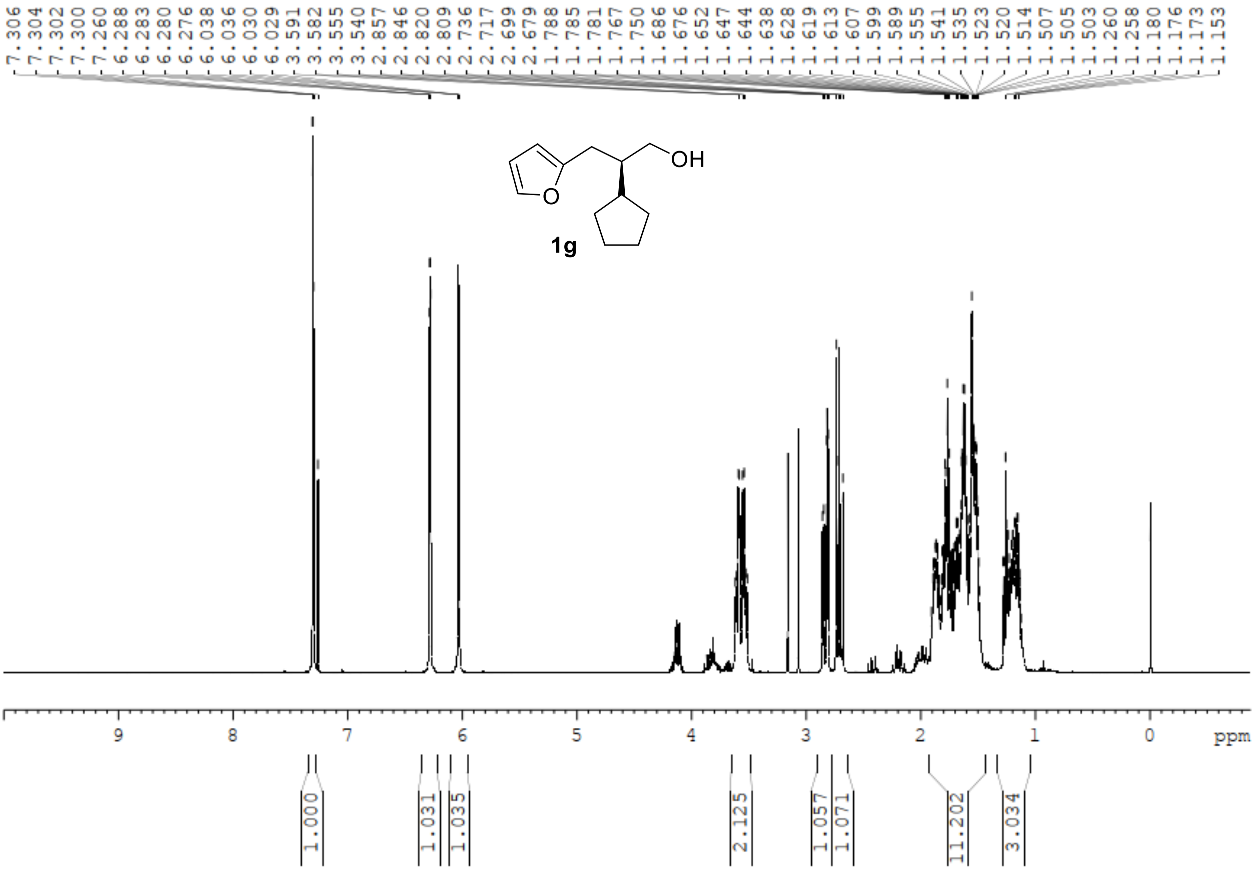
7.306
7.304
7.302
7.300
7.260
6.288
6.283
6.280
6.276
6.038
6.036
6.030
6.029
3.591
3.582
3.555
3.540
2.857
2.846
2.820
2.809
2.736
2.717
2.699
2.679
1.788
1.785
1.781
1.767
1.750
1.686
1.676
1.652
1.647
1.644
1.638
1.628
1.619
1.613
1.607
1.599
1.589
1.555
1.541
1.535
1.523
1.520
1.514
1.507
1.505
1.503
1.260
1.258
1.180
1.176
1.173
1.153
OH
O
1g
9
8
7
6
5
4
3
2
1
0
ppm
1.000
1.031
1.035
2.125
1.057
1.071
11.202
3.034

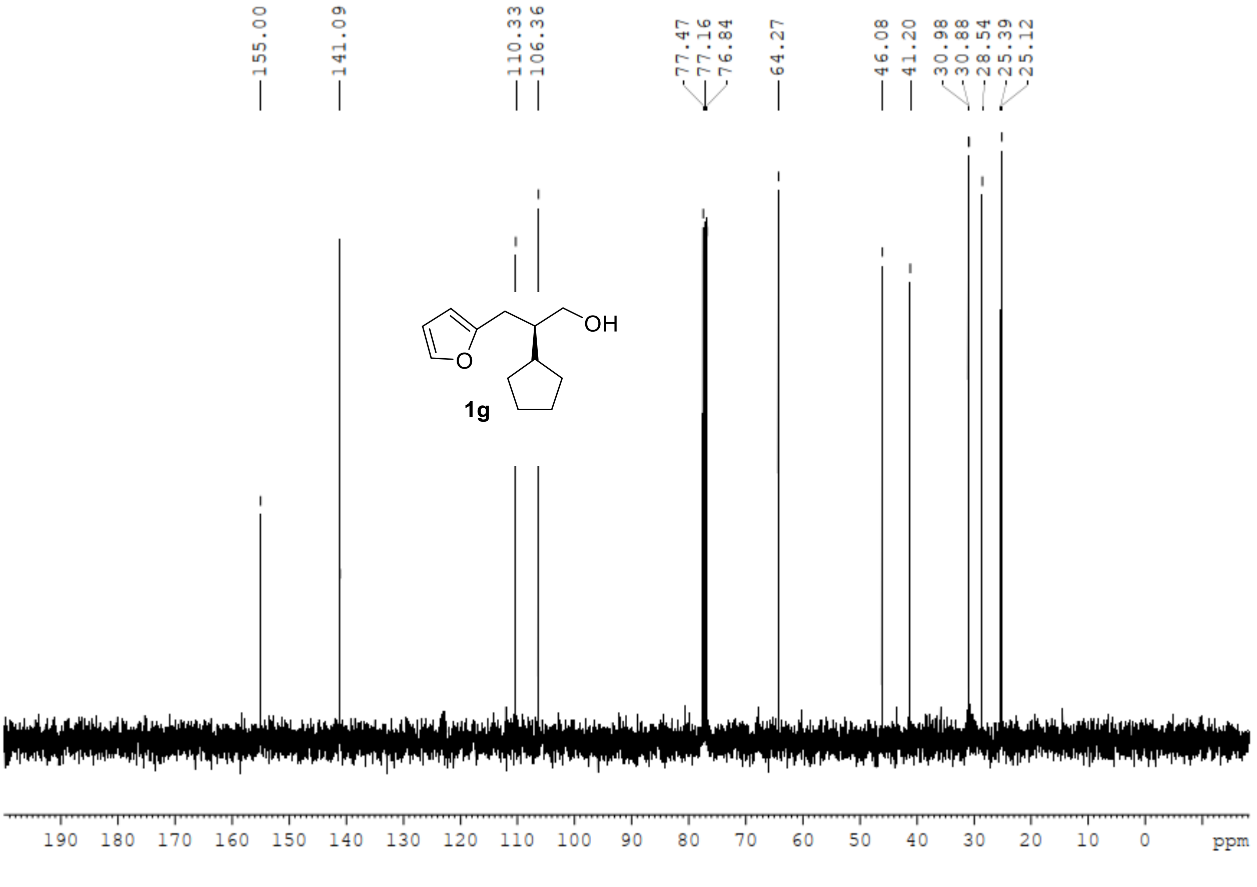
155.00
141.09
110.33
106.36
77.47
77.16
76.84
64.27
46.08
41.20
30.98
30.88
28.54
25.39
25.12
OH
O
1g
190
180
170
160
150
140
130
120
110
100
90
80
70
60
50
40
30
20
10
0
ppm

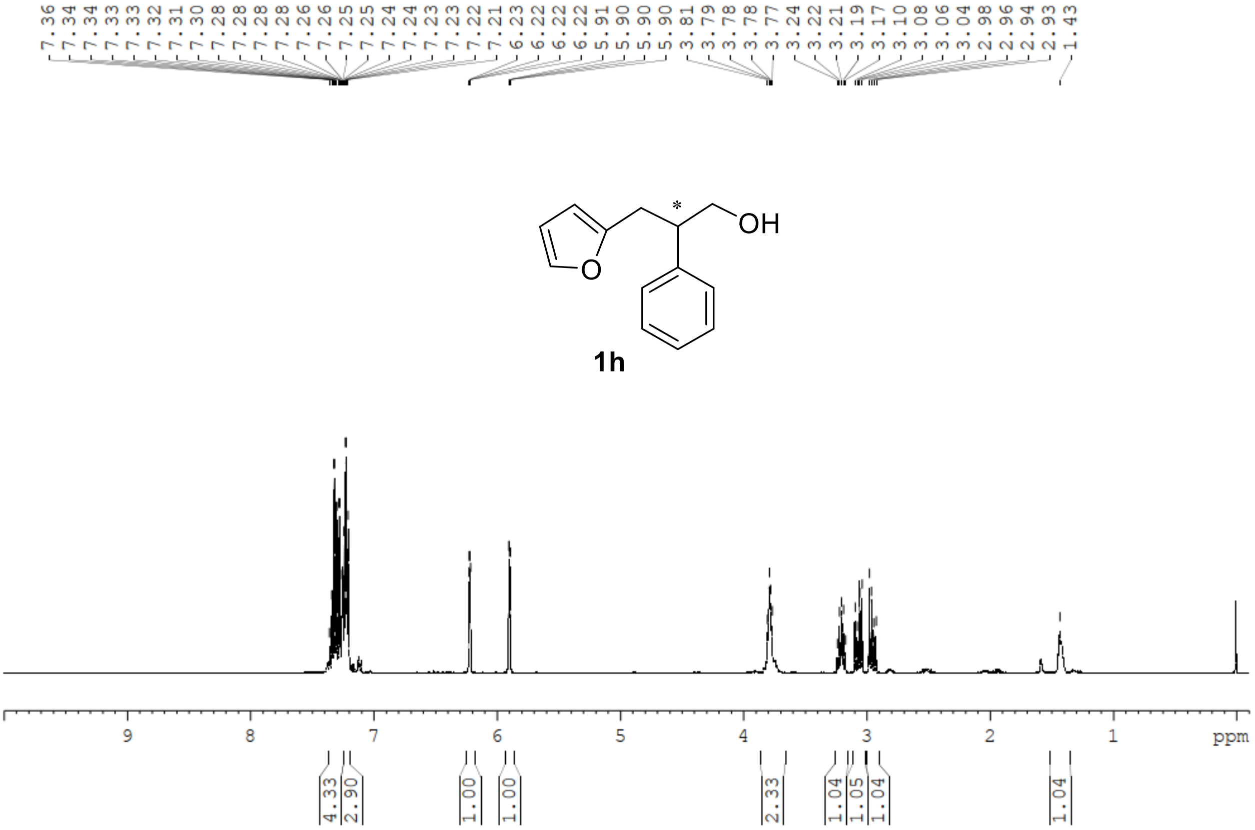
7.36 7.34 7.34 7.33 7.33 7.32 7.31 7.30 7.28 7.28 7.28 7.26 7.26 7.25 7.25 7.24 7.24 7.23 7.23 7.22 7.21 6.23 6.22 6.22 6.22 5.91 5.90 5.90 5.90 3.81 3.79 3.78 3.78 3.77 3.24 3.22 3.21 3.19 3.17 3.10 3.08 3.06 3.04 2.98 2.96 2.94 2.93 1.43
OH
O
1h
9 8 7 6 5 4 3 2 1 ppm
4.33 2.90 1.00 1.00 2.33 1.04 1.05 1.04 1.04

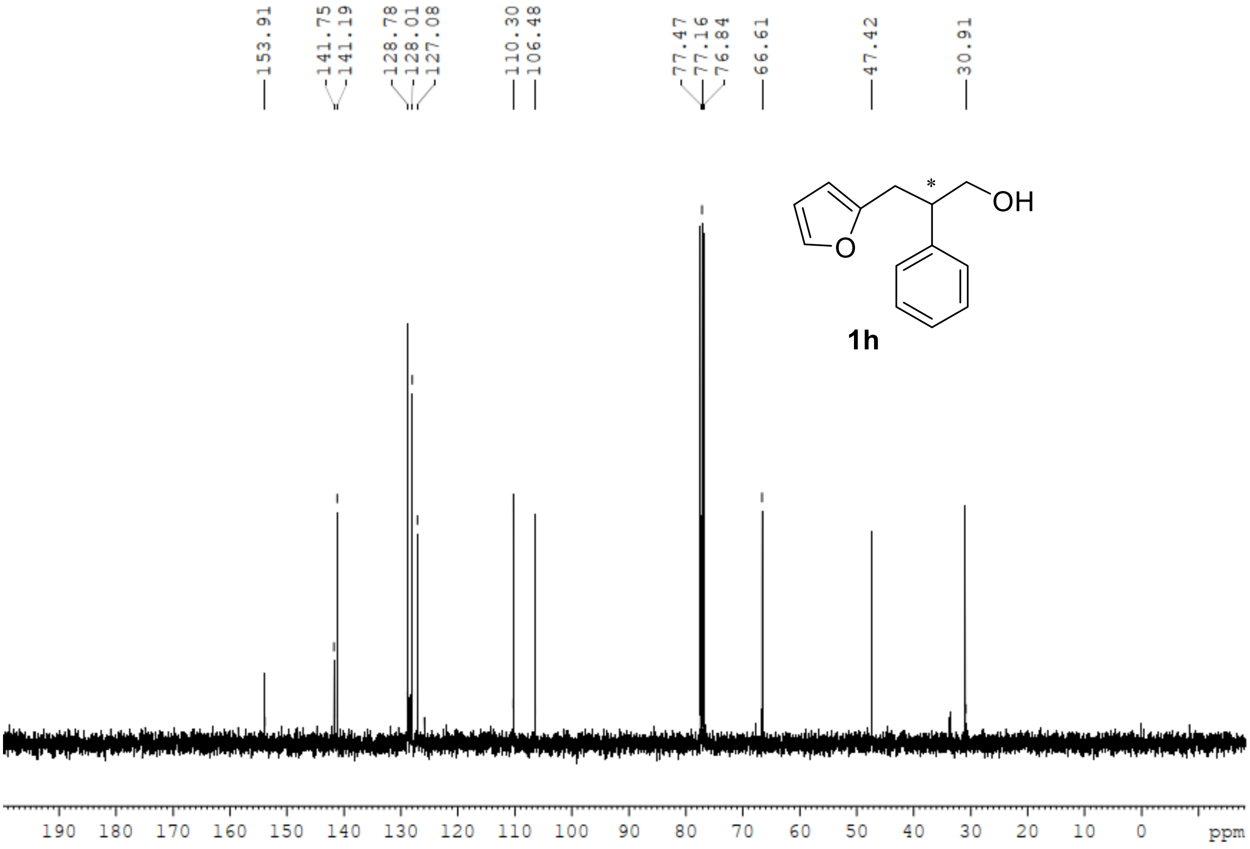
153.91 141.75 141.19 128.78 128.01 127.08 110.30 106.48 77.47 77.16 76.84 66.61 47.42 30.91
OH
O
1h
190 180 170 160 150 140 130 120 110 100 90 80 70 60 50 40 30 20 10 0 ppm

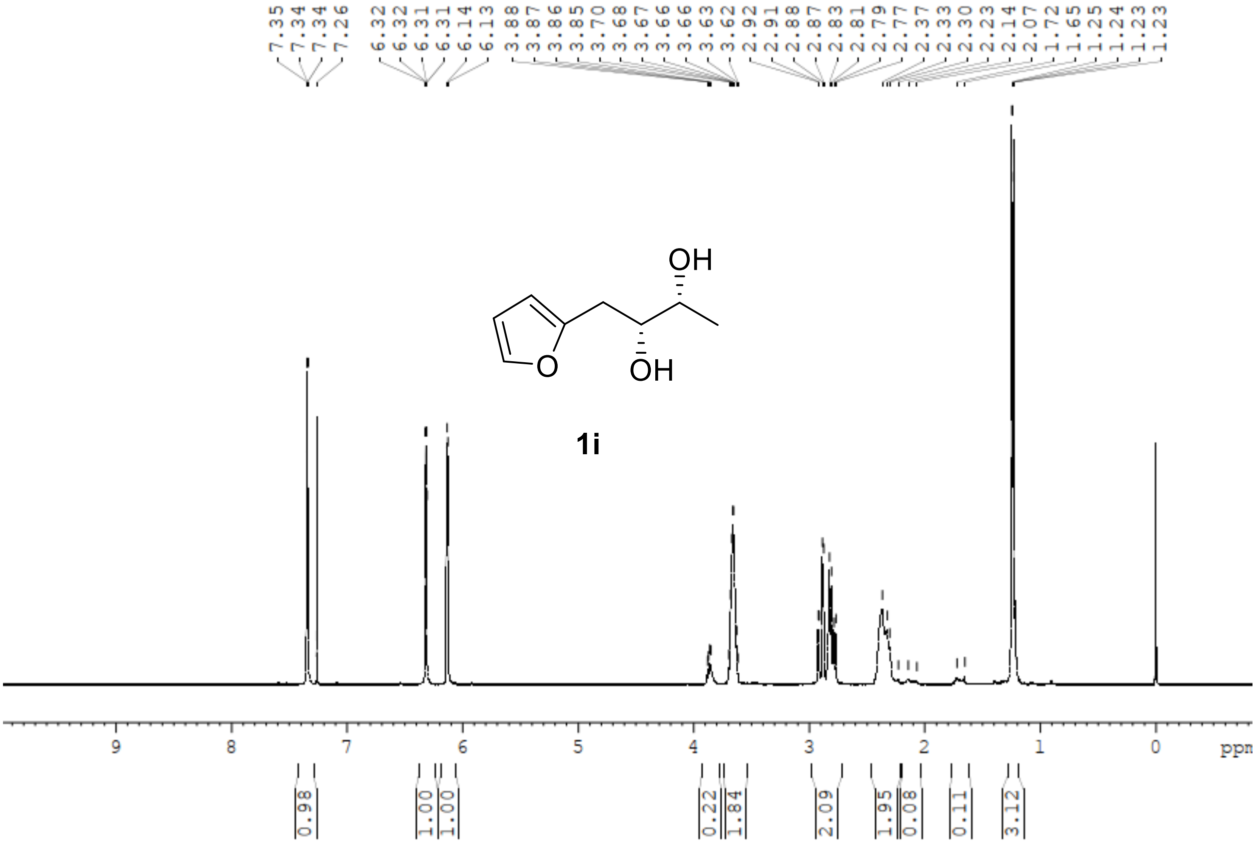
7.35
7.34
7.34
7.26
6.32
6.32
6.31
6.31
6.14
6.13
3.88
3.87
3.86
3.85
3.70
3.68
3.67
3.66
3.66
3.63
3.62
2.92
2.91
2.88
2.87
2.83
2.81
2.79
2.77
2.37
2.33
2.30
2.23
2.14
2.07
1.72
1.65
1.25
1.24
1.23
1.23
OH
O
OH
1i
ppm
0.98
1.00
1.00
0.22
1.84
2.09
1.95
0.08
0.11
3.12

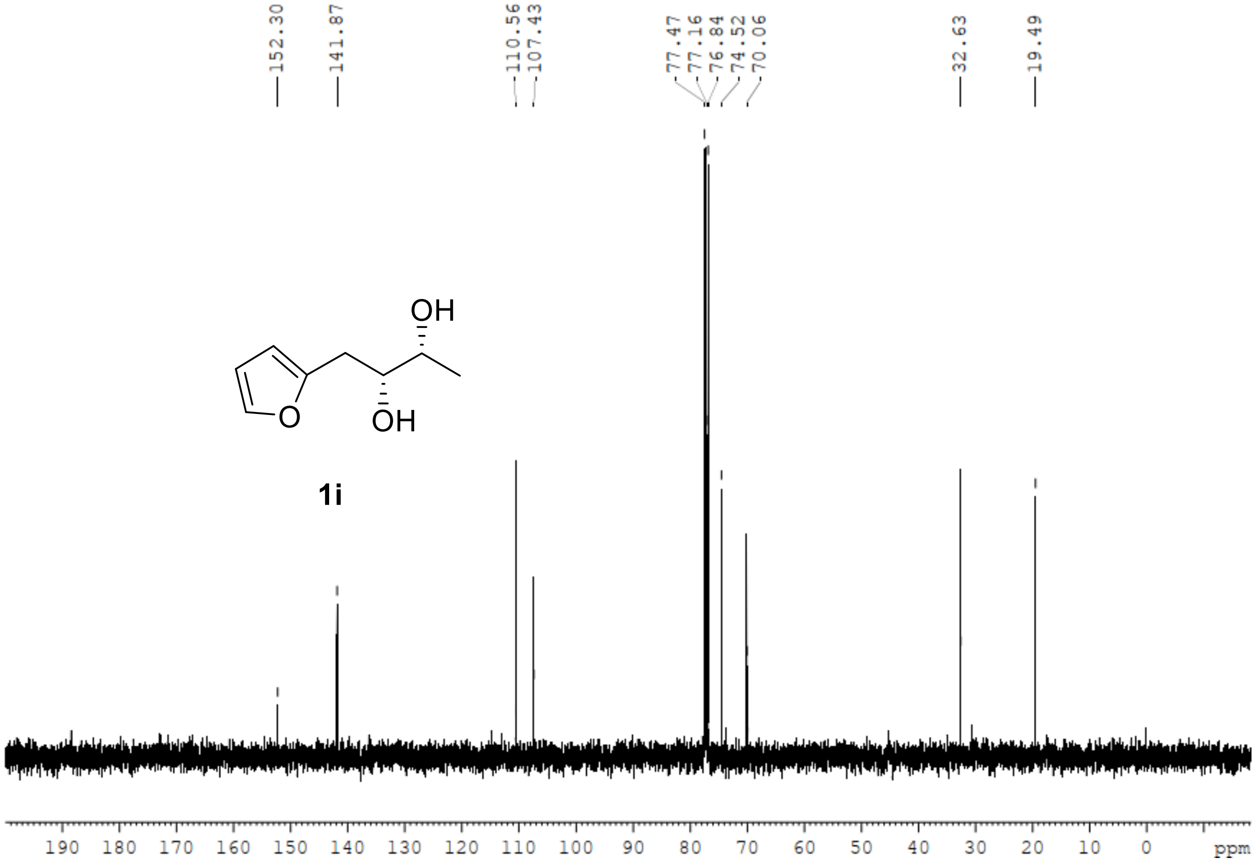
152.30
141.87
110.56
107.43
77.47
77.16
76.84
74.52
70.06
32.63
19.49
OH
O
OH
1i
ppm

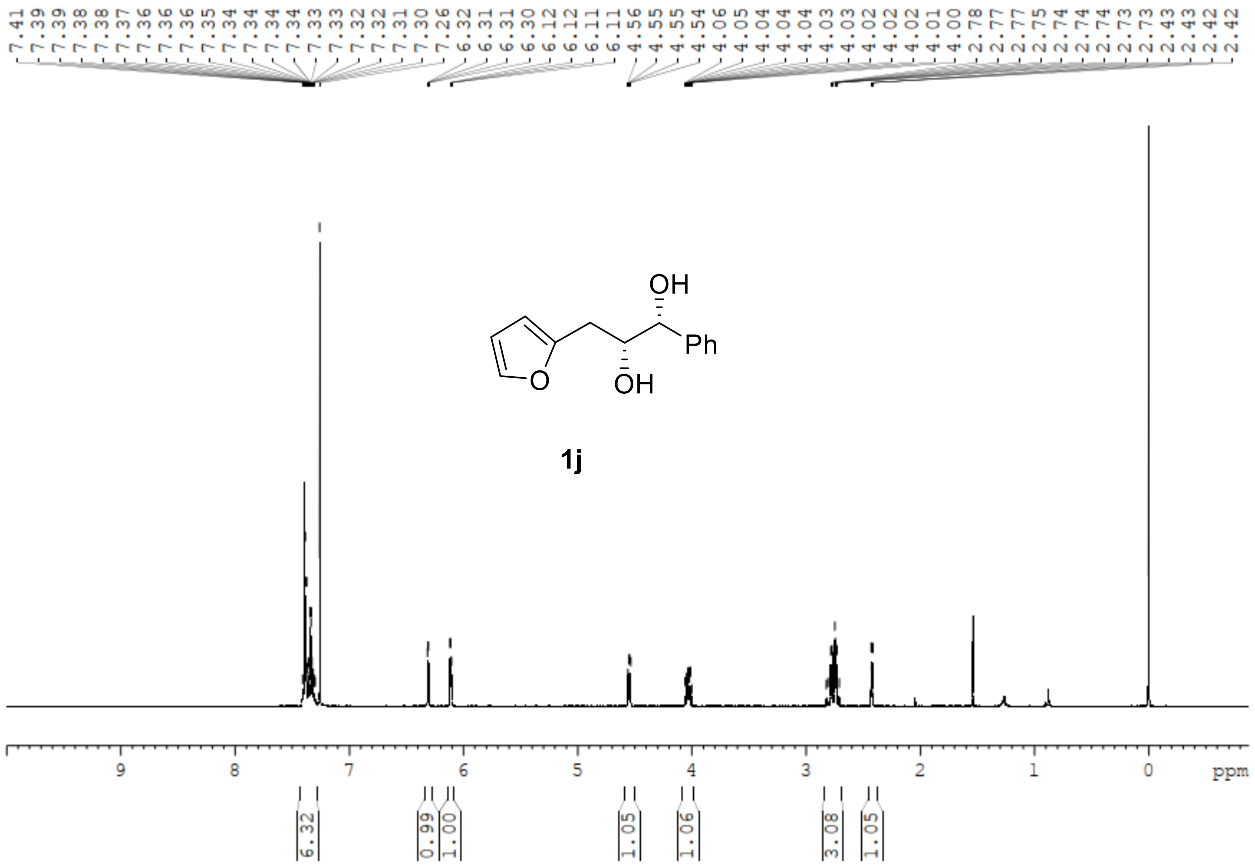
7.41 7.39 7.39 7.38 7.38 7.37 7.36 7.36 7.36 7.35 7.34 7.34 7.34 7.34 7.33 7.33 7.32 7.32 7.31 7.30 7.26 6.32 6.31 6.31 6.30 6.12 6.12 6.11 6.11 4.56 4.55 4.55 4.54 4.06 4.05 4.04 4.04 4.04 4.03 4.03 4.02 4.02 4.02 4.01 4.00 2.78 2.77 2.77 2.75 2.74 2.74 2.74 2.73 2.73 2.43 2.43 2.42 2.42
OH
Ph
O
OH
1j
9 8 7 6 5 4 3 2 1 0 ppm
6.32 0.99 1.00 1.05 1.06 3.08 1.05

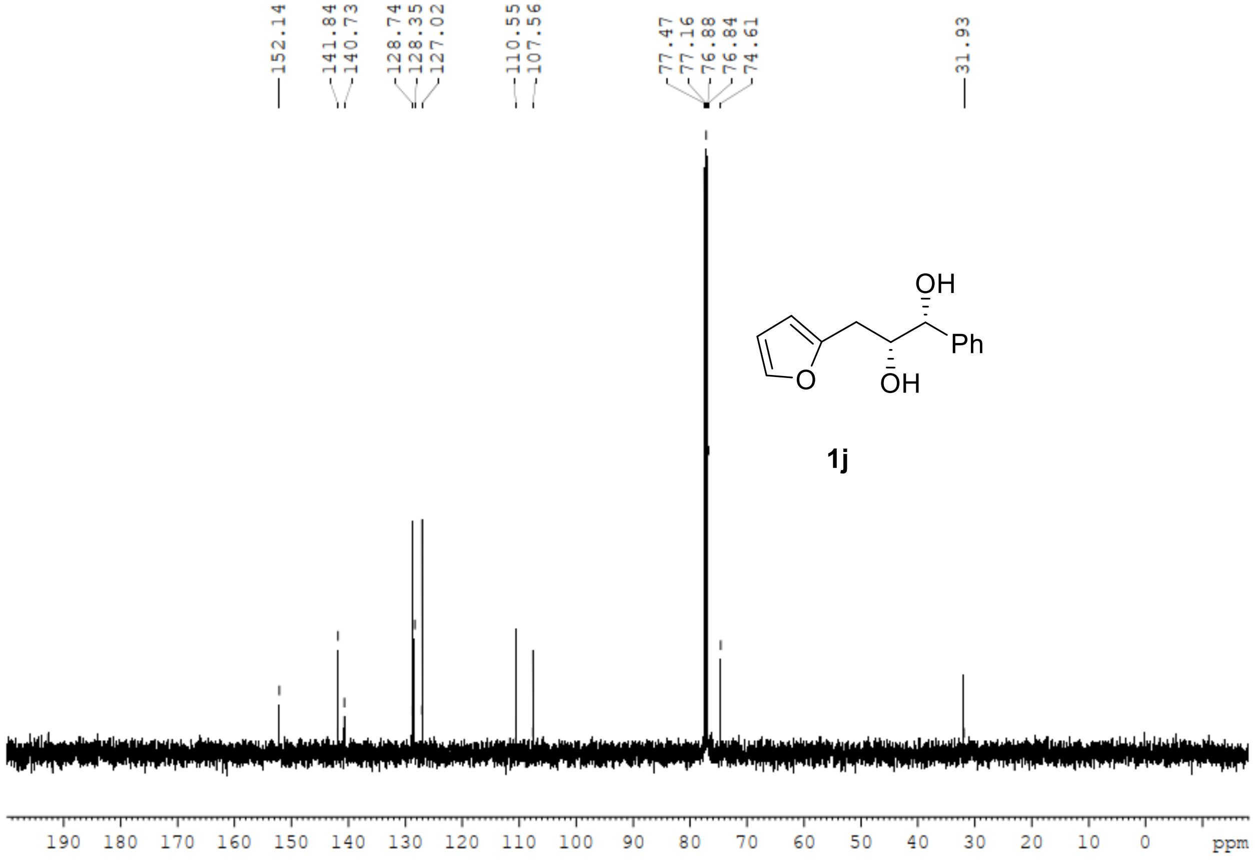
152.14 141.84 140.73 128.74 128.35 127.02 110.55 107.56 77.47 77.16 76.88 76.84 74.61 31.93
OH
Ph
O
OH
1j
190 180 170 160 150 140 130 120 110 100 90 80 70 60 50 40 30 20 10 0 ppm

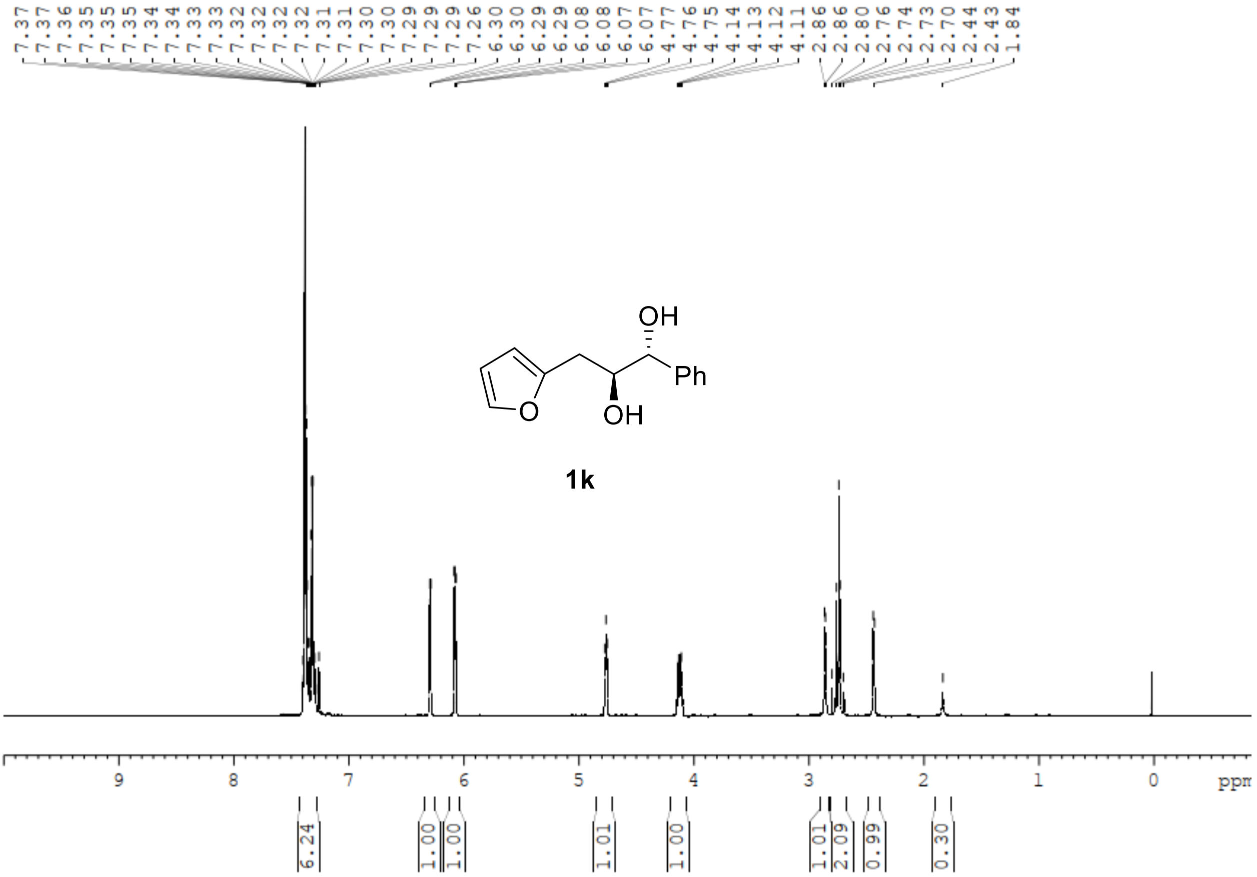
7.37 7.37 7.36 7.35 7.35 7.35 7.34 7.34 7.33 7.33 7.32 7.32 7.32 7.32 7.31 7.31 7.30 7.30 7.29 7.29 7.29 7.26 6.30 6.30 6.29 6.29 6.08 6.08 6.07 6.07 4.77 4.76 4.75 4.14 4.13 4.12 4.11 2.86 2.86 2.80 2.76 2.74 2.73 2.70 2.44 2.43 1.84
OH
Ph
O
OH
1k
6.24
1.00
1.00
1.01
1.00
1.01
2.09
0.99
0.30
ppm

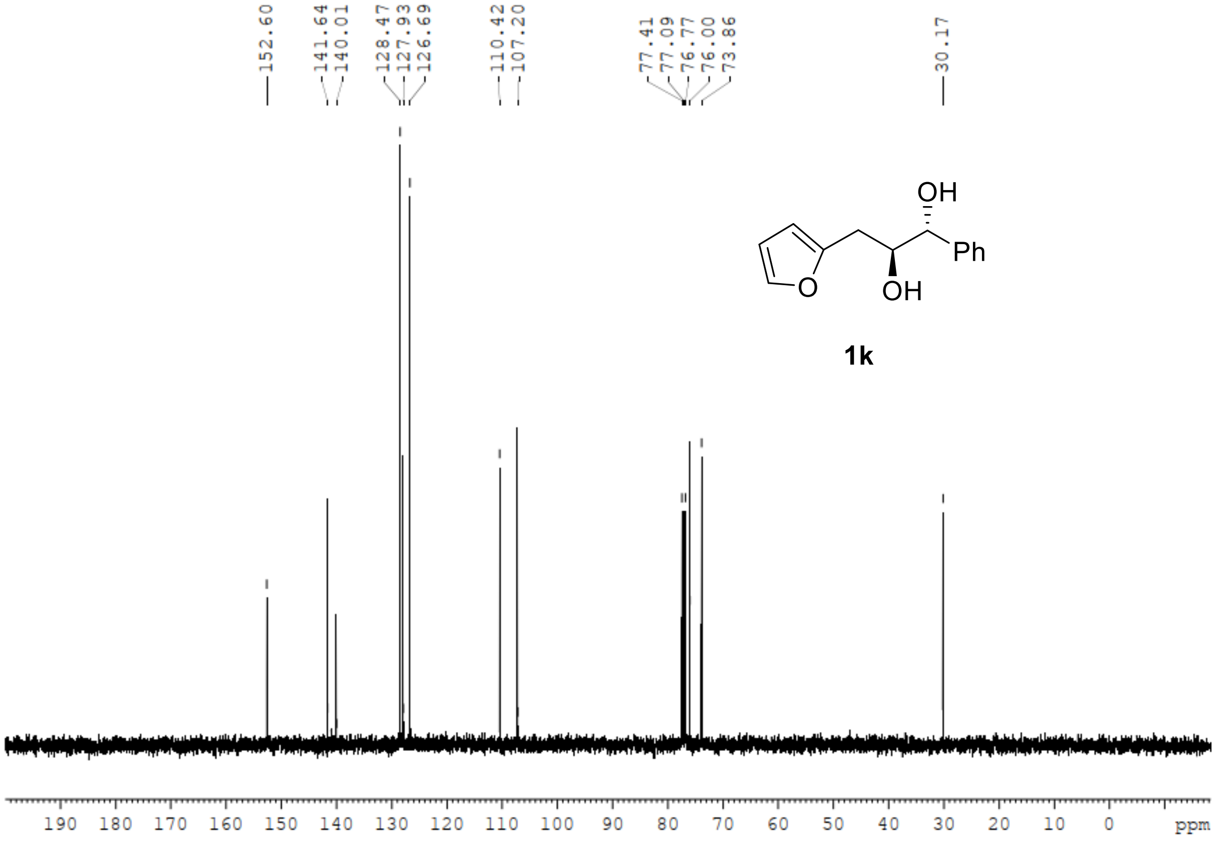
152.60
141.64
140.01
128.47
127.93
126.69
110.42
107.20
77.41
77.09
76.77
76.00
73.86
30.17
OH
Ph
O
OH
1k
ppm

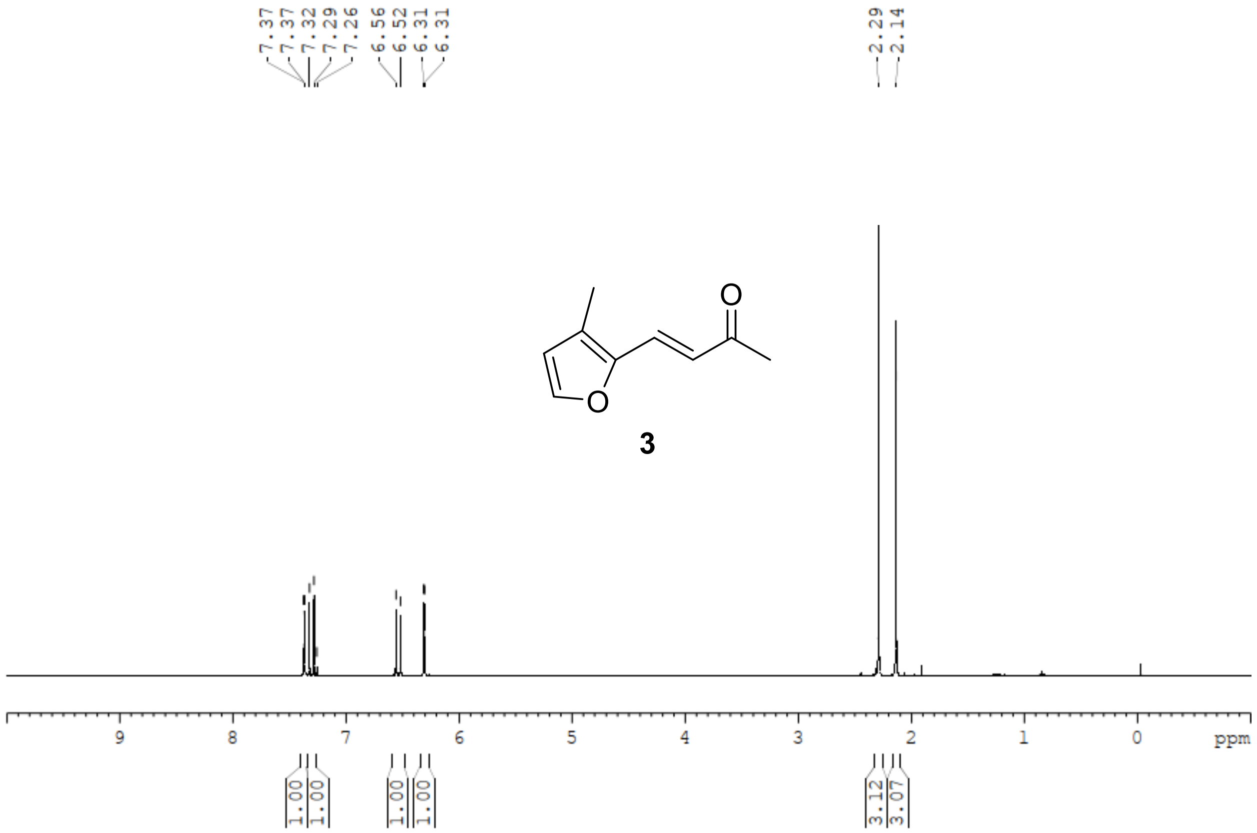
7.37
7.37
7.32
7.29
7.26
6.56
6.52
6.31
6.31
2.29
2.14
O
O
3
9
8
7
6
5
4
3
2
1
0
ppm
1.00
1.00
1.00
1.00
3.12
3.07

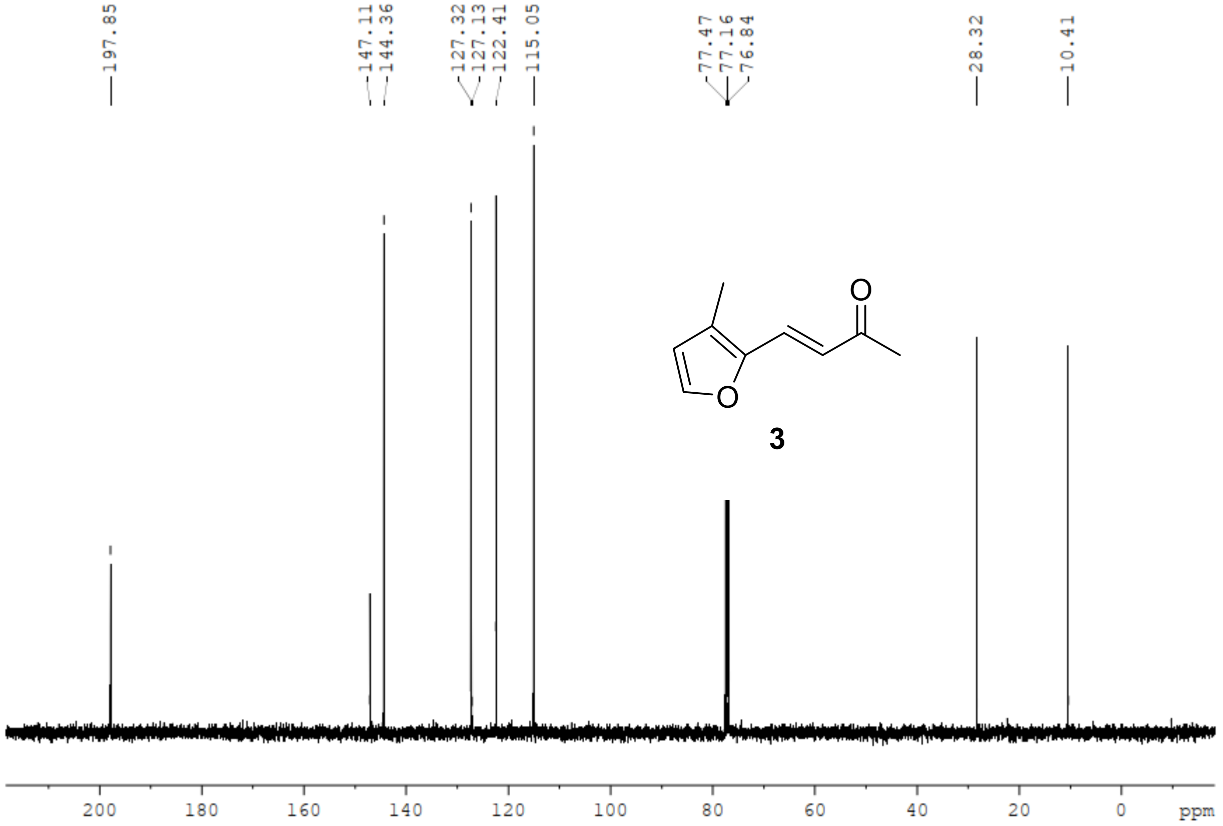
197.85
147.11
144.36
127.32
127.13
122.41
115.05
77.47
77.16
76.84
28.32
10.41
O
O
3
200
180
160
140
120
100
80
60
40
20
0
ppm

6.3 HPLC traces

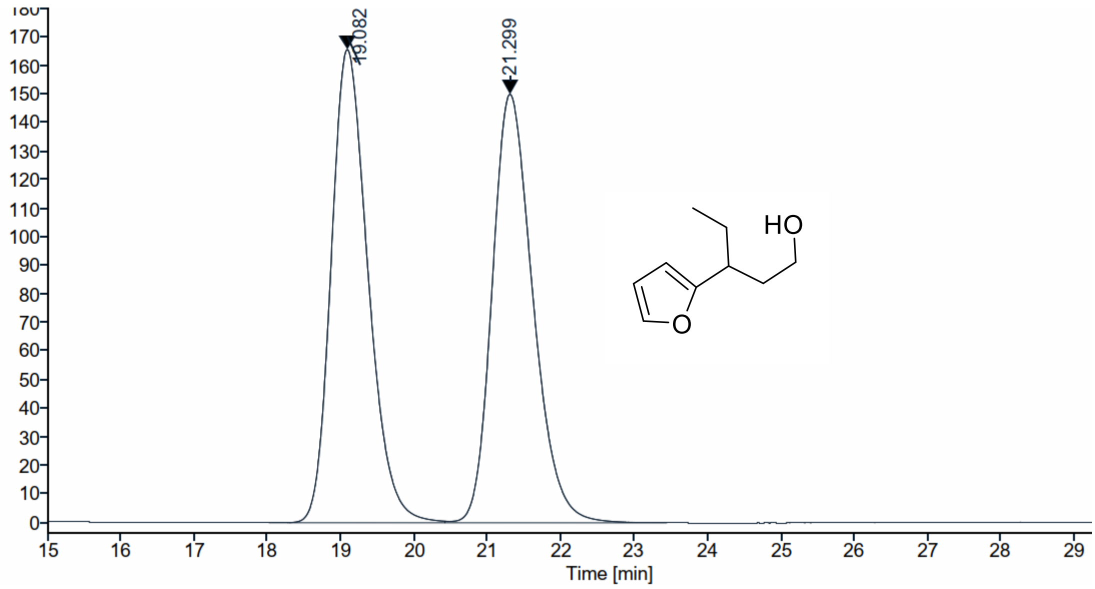

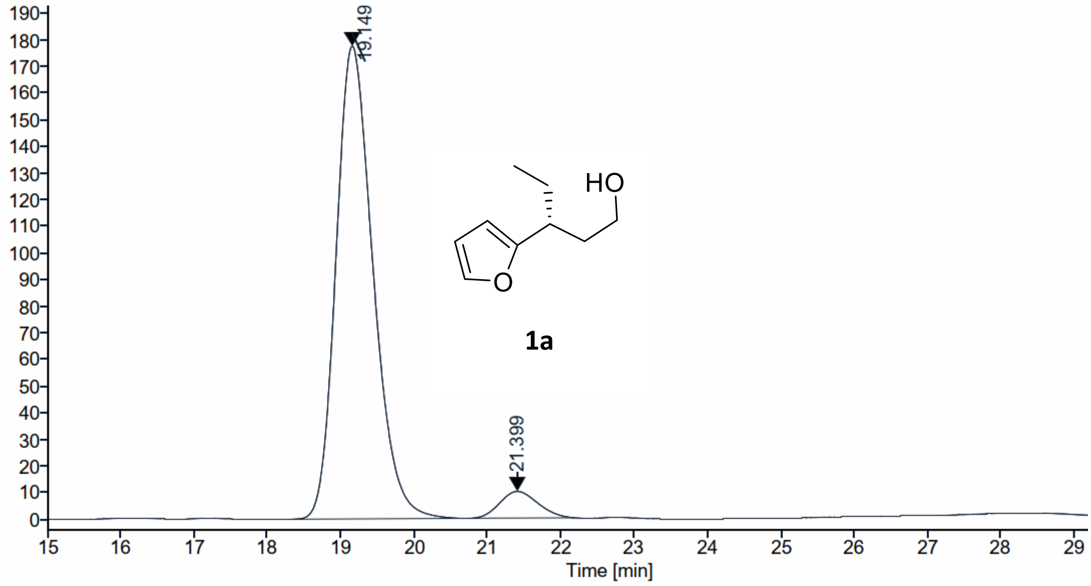

Signal: DAD1D,Sig=230,4 Ref=off

RT [min]	Name	Area	Area%
19.149		6234.3673	94.5322
21.399		360.6026	5.4678

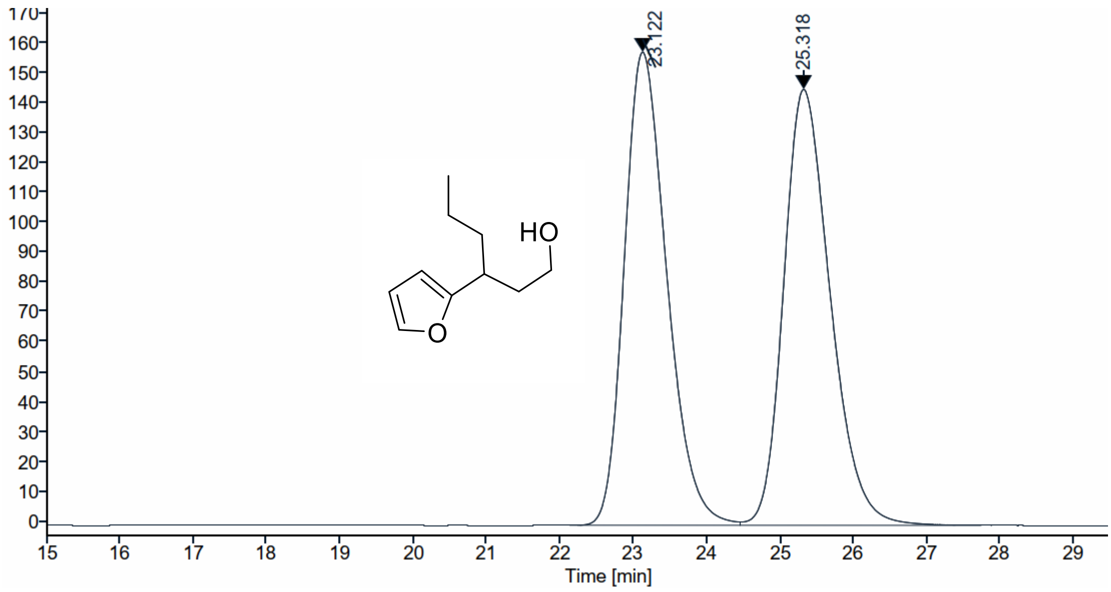

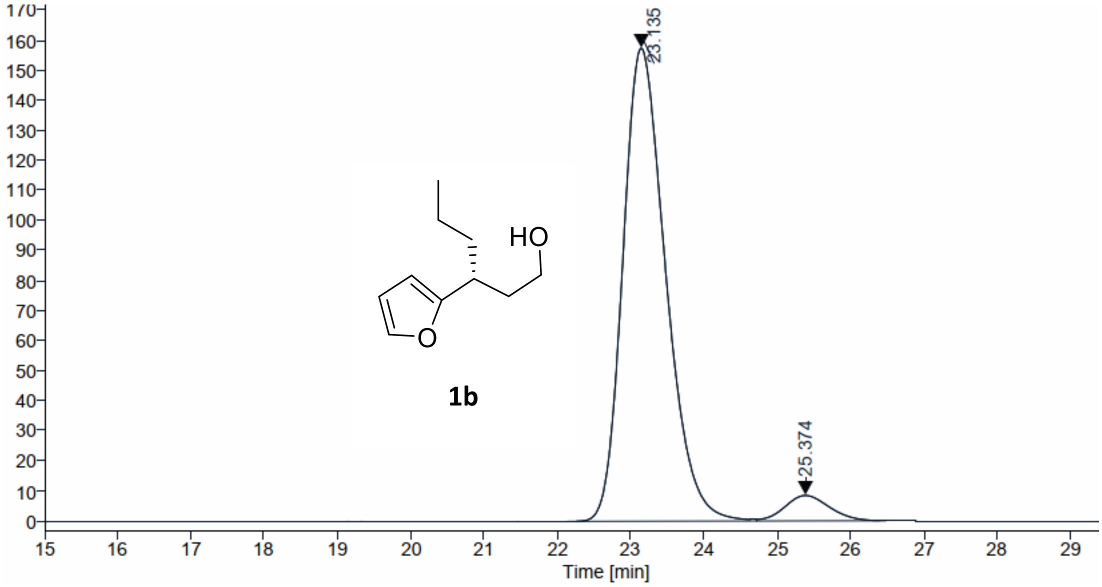

Signal: DAD1D,Sig=230,4 Ref=off

RT [min]	Name	Area	Area%
23.135		6374.3539	94.6998
25.374		356.7658	5.3002

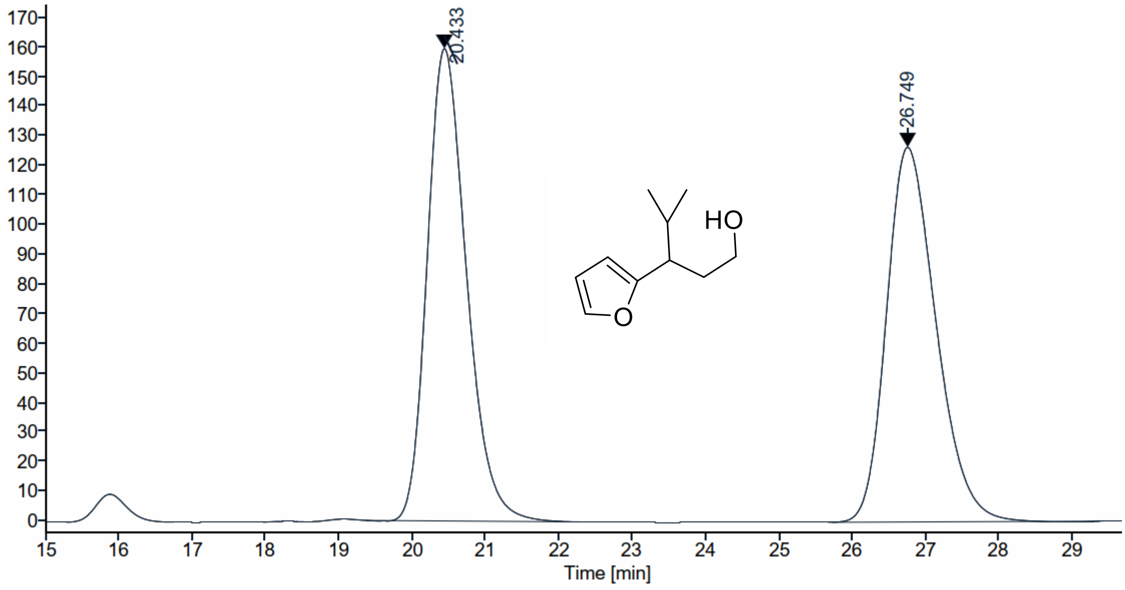

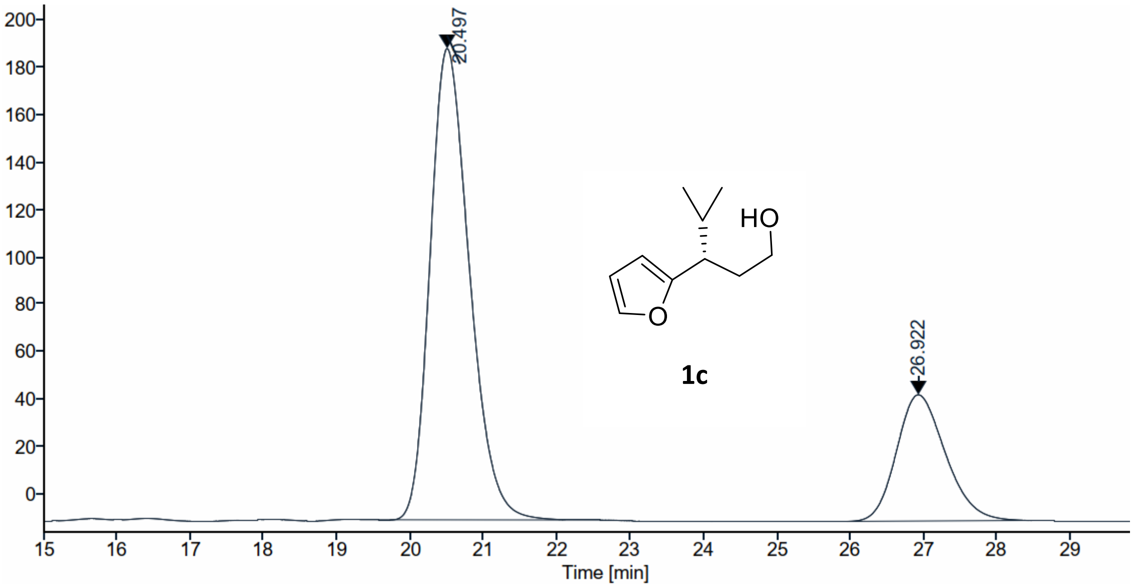

Signal: DAD1D,Sig=230,4 Ref=off

RT [min]	Name	Area	Area%
20.497		7415.2270	75.0349
26.922		2467.1397	24.9651

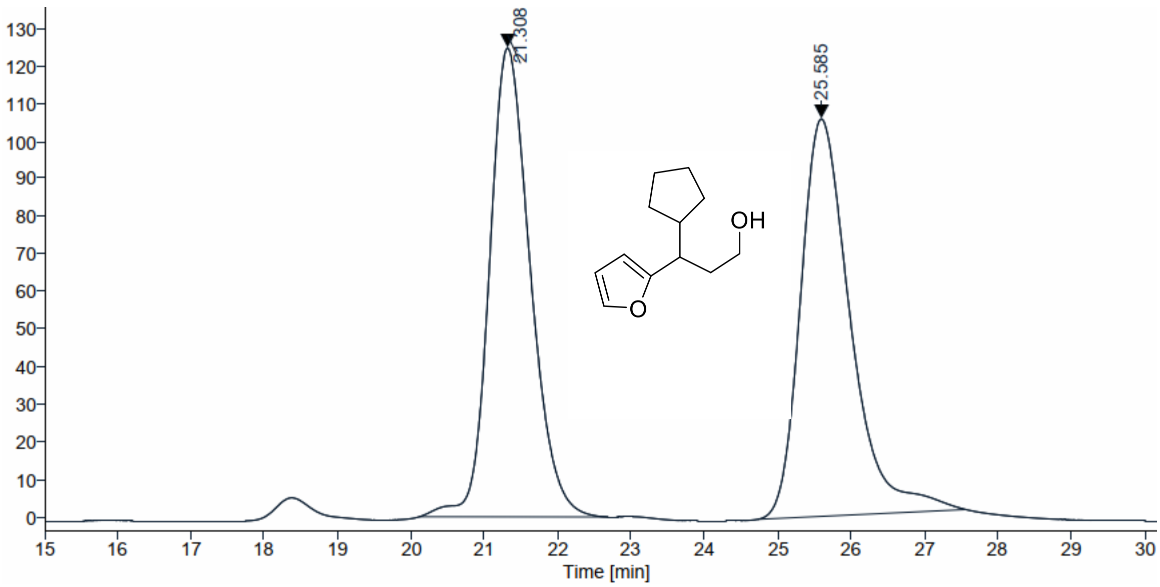

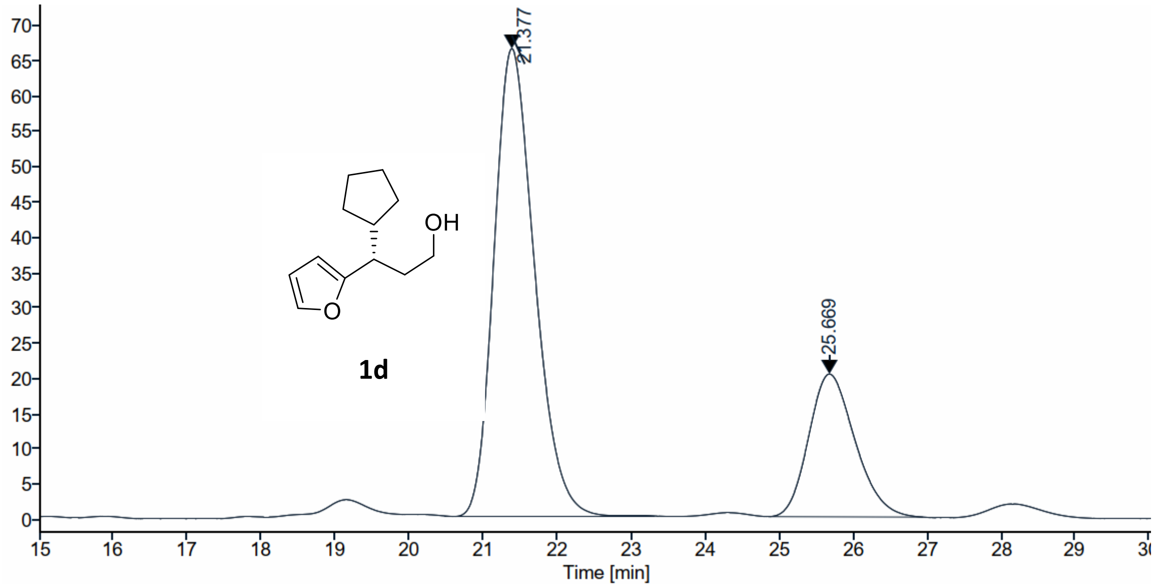

Signal: DAD1D,Sig=230,4 Ref=off

RT [min]	Name	Area	Area%
21.377		2519.4884	74.1217
25.669		879.6349	25.8783

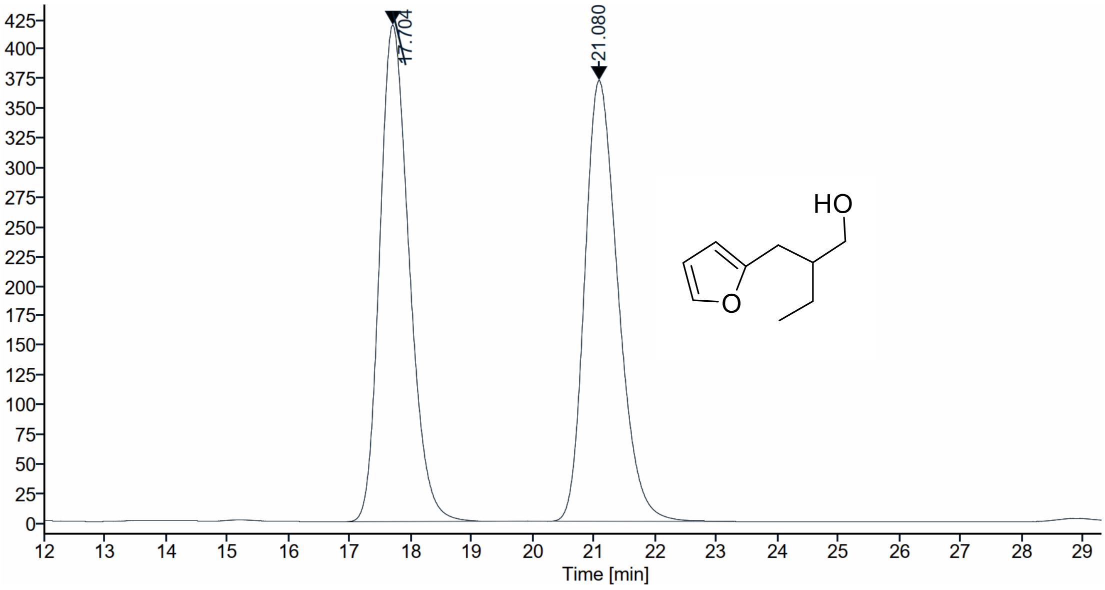

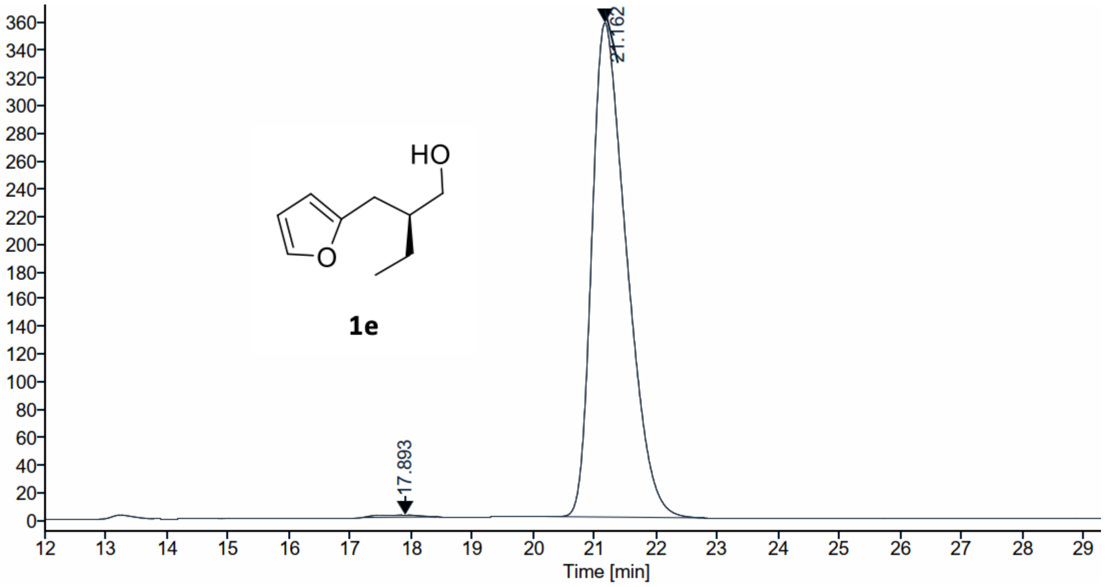

Signal: DAD1D,Sig=230,4 Ref=off

RT [min]	Name	Area	Area%
17.893		76.6257	0.5410
21.162		14087.2788	99.4590

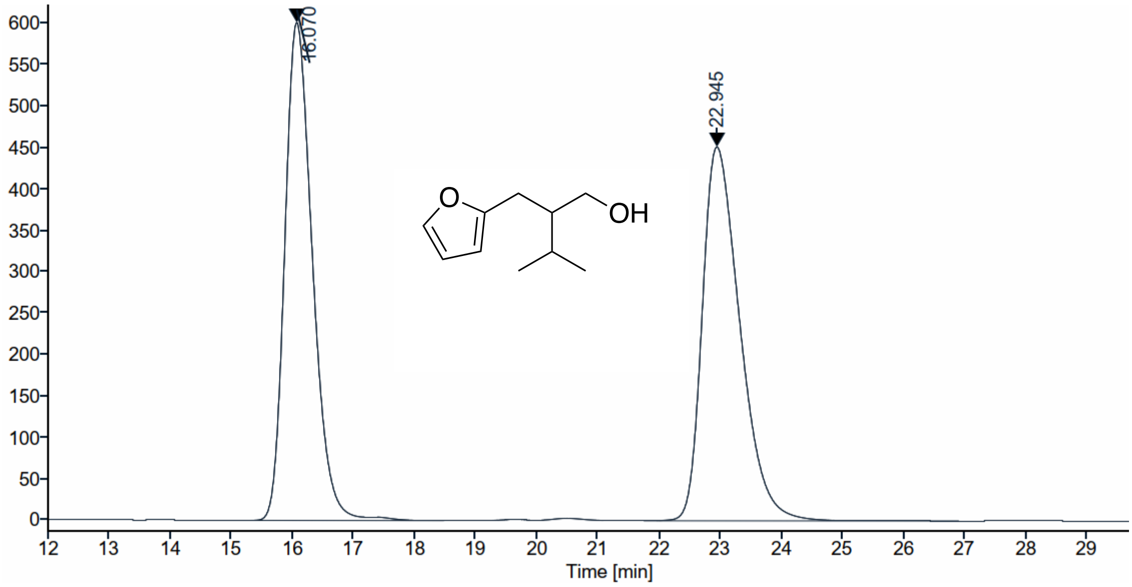

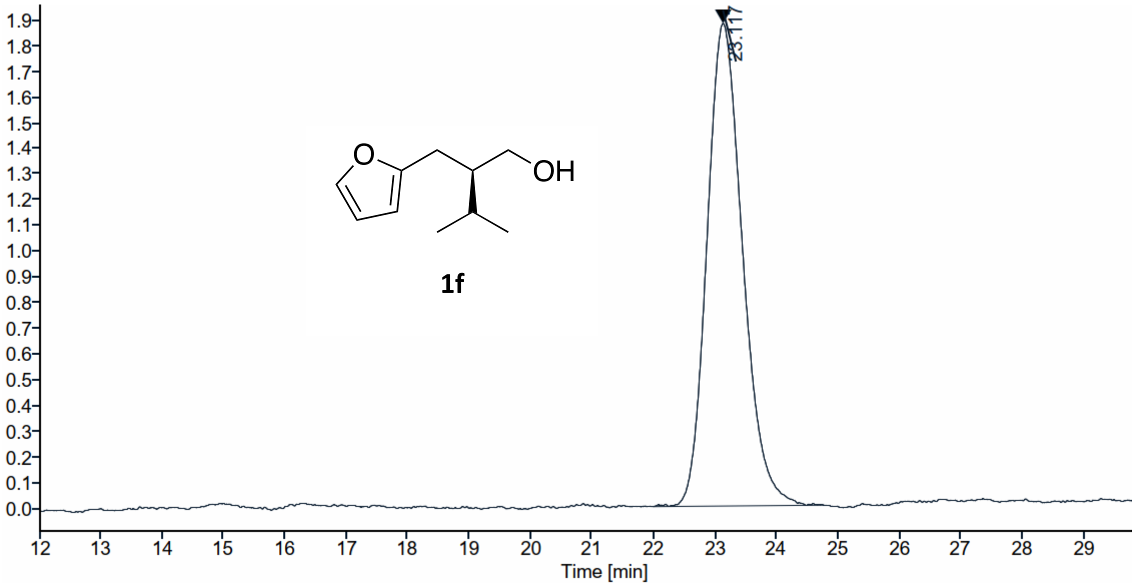

Signal: DAD1D,Sig=230,4 Ref=off

RT [min]	Name	Area	Area%
23.117		75.6126	100.0000

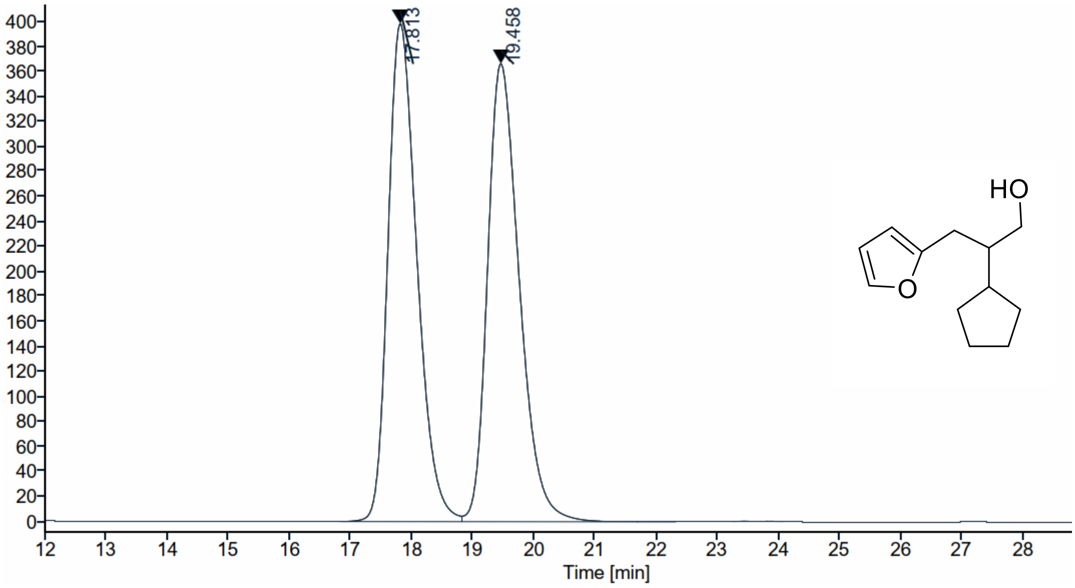

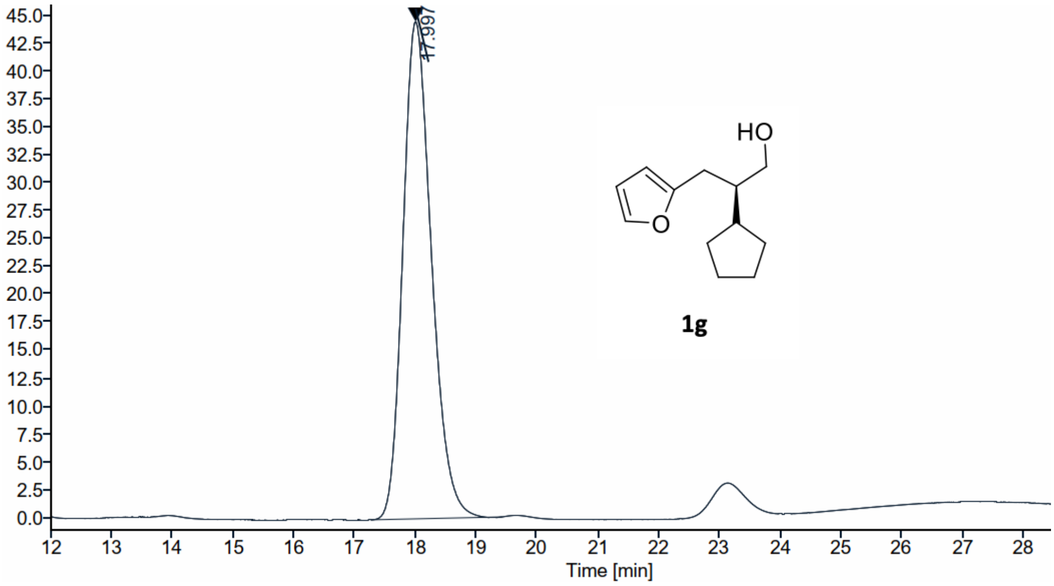

Signal: DAD1D,Sig=230,4 Ref=off

RT [min]	Name	Area	Area%
17.997		1453.3694	100.0000

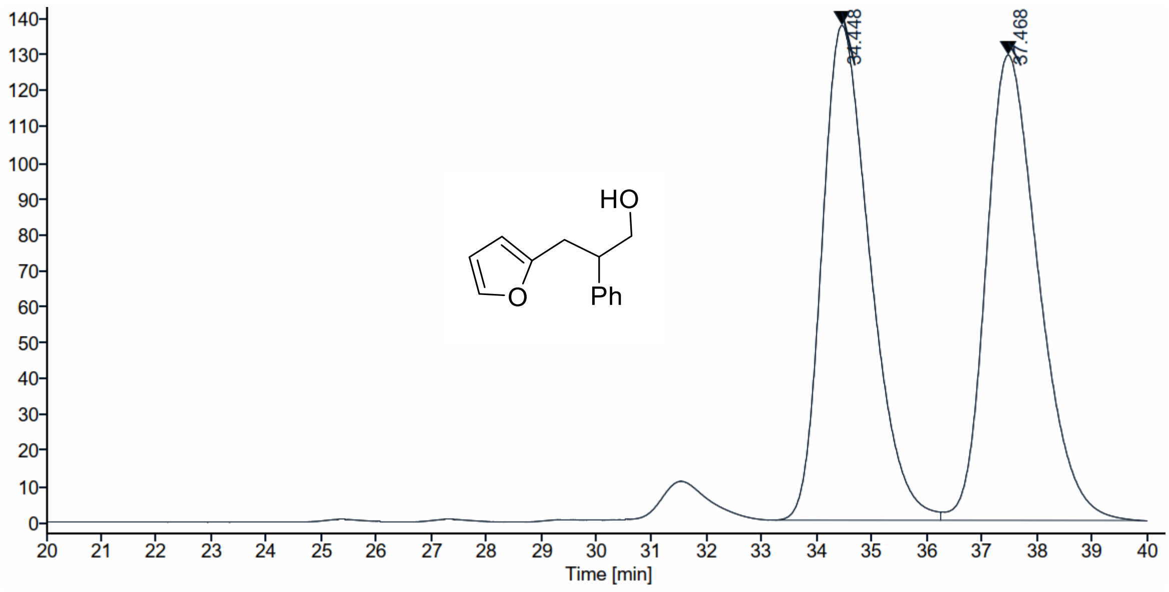

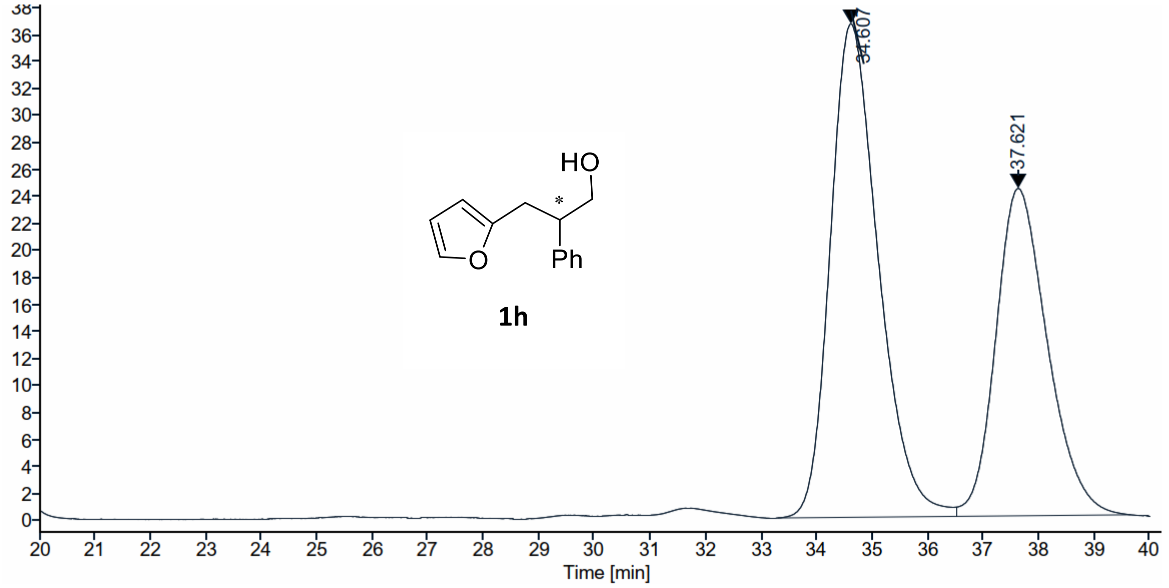

Signal: DAD1D,Sig=230,4 Ref=off

RT [min]	Name	Area	Area%
34.607		2208.0823	58.5095
37.621		1565.8075	41.4905

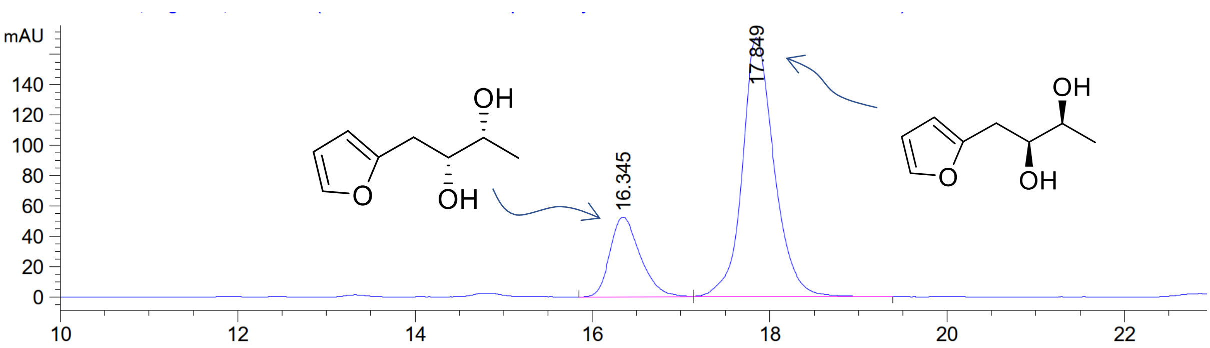
mAU
16.345
17.849
OH
OH

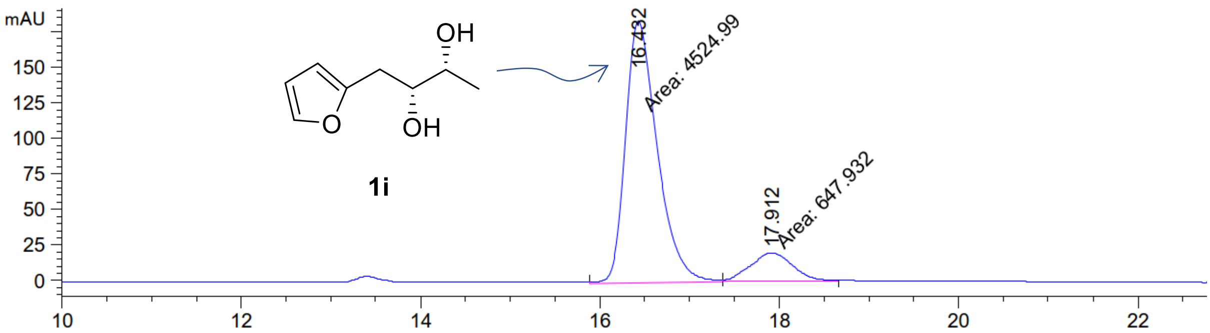
mAU
16.432
Area: 4524.99
17.912
Area: 647.932
OH
OH
1i

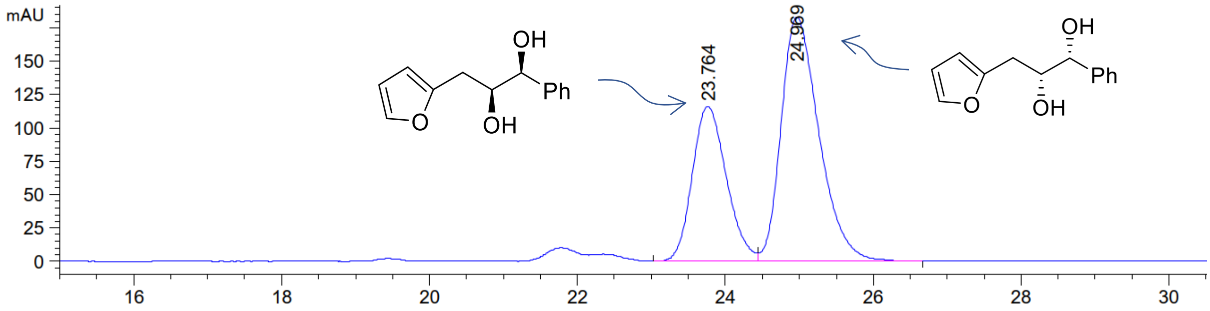
mAU
23.764
24.969
OH
Ph
OH

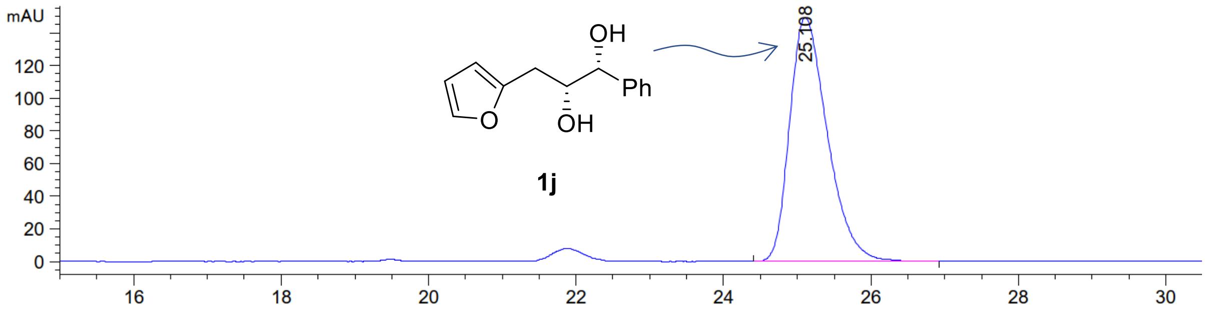
mAU
25.108
OH
Ph
OH
1j

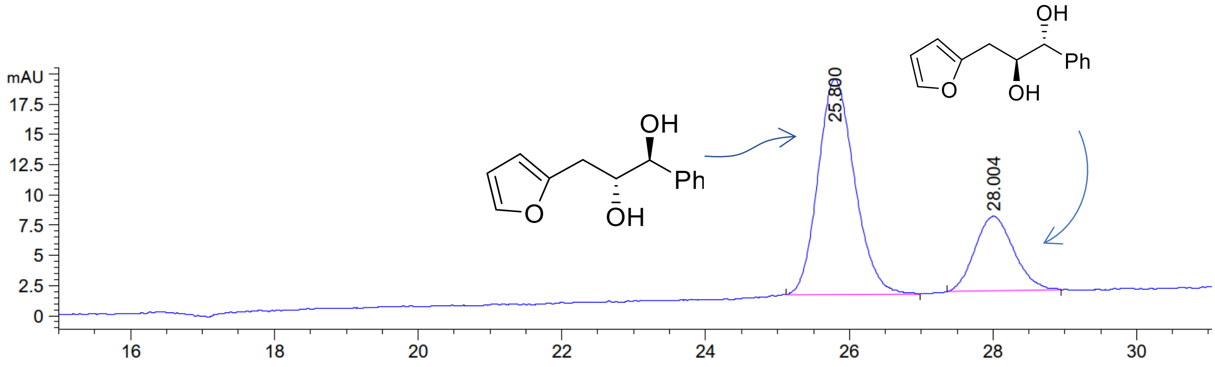
mAU
25.800
28.004
OH
Ph
OH

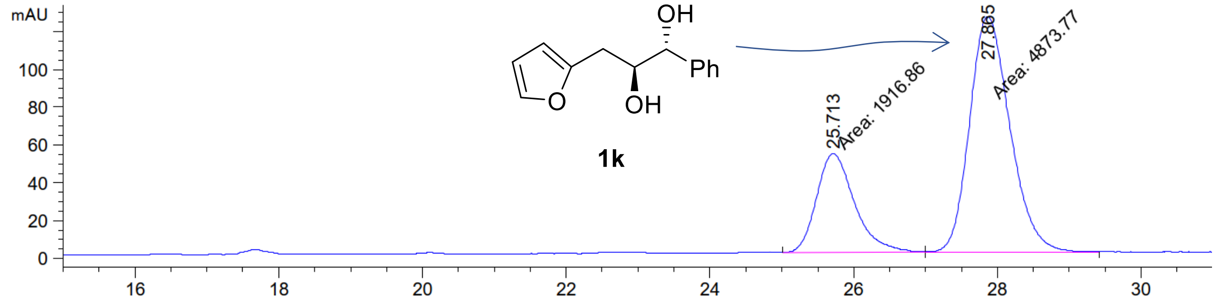
mAU
25.713
Area: 1916.86
27.865
Area: 4873.77
OH
Ph
OH
1k

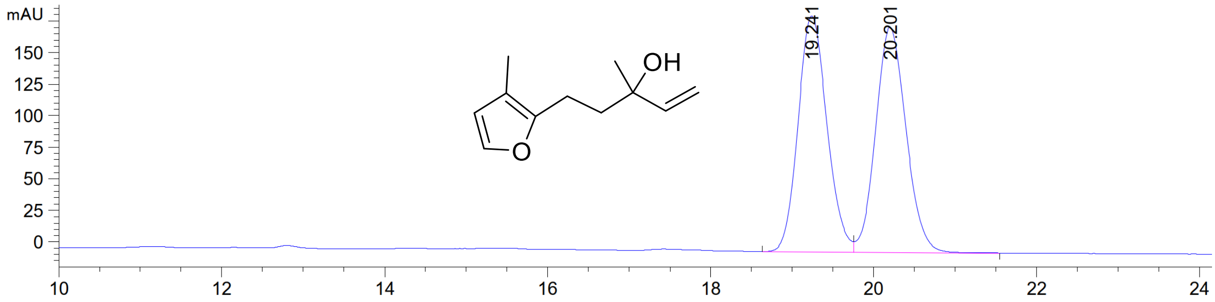
mAU
19.241
20.201
OH

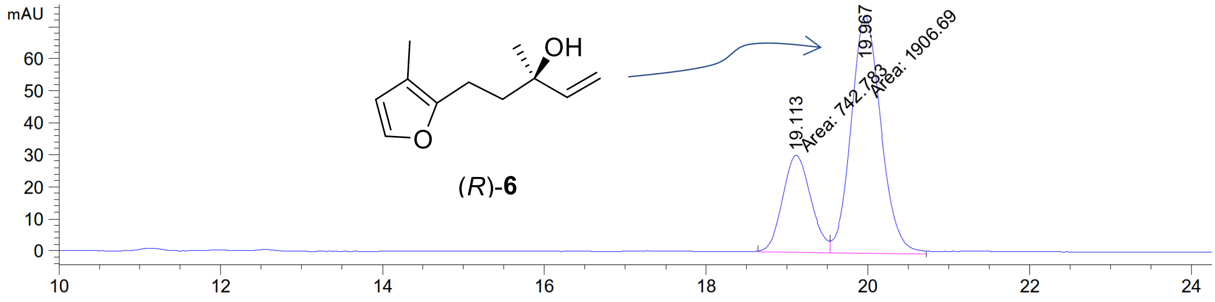
mAU
19.113
Area: 742.783
19.967
Area: 1906.69
OH
(R)-6

Supplementary References

1. Neese, F., The ORCA program system. *Wiley Interdisciplinary Reviews: Computational Molecular Science,* **2012**, *2*, 73-78.

2. Neese, F., Software update: the ORCA program system, version 4.0. *Wiley Interdisciplinary Reviews: Computational Molecular Science*, **2018**, 8.

3. Adamo, C.; V. Barone, Toward reliable density functional methods without adjustable parameters: The PBE0 model. *J. Chem. Phys.*, **1999**, *110*, 6158-6170.

4. Weigend, F.; Ahlrichs R., Balanced basis sets of split valence, triple zeta valence and quadruple zeta valence quality for H to Rn: Design and assessment of accuracy. *Phys. Chem. Chem. Phys.*, **2005**, *7*, 3297-3305.

5. Grimme, S., et al., A consistent and accurate ab initio parametrization of density functional dispersion correction (DFT-D) for the 94 elements H-Pu. *J. Chem. Phys.*, **2010**, *132*, 154104.

6. Grimme, S., S. Ehrlich, and L. Goerigk, Effect of the damping function in dispersion corrected density functional theory. *J. Comput. Chem.*, **2011**, *32*, 1456-1465.

7. Neese, F., An improvement of the resolution of the identity approximation for the formation of the Coulomb matrix. *J. Comput. Chem.*, **2003**, *24*, 1740-1747.

8. Weigend, F., Accurate Coulomb-fitting basis sets for H to Rn. *Phys. Chem. Chem. Phys*. **2006**, *8*, 1057-1065.

9. Neese, F., et al., Efficient, approximate and parallel Hartree–Fock and hybrid DFT calculations. A 'chain-of-spheres' algorithm for the Hartree–Fock exchange. *Chem. Phys.* **2009**, *356*, 98-109.

10. Hanwell, M.D., et al., Avogadro: an advanced semantic chemical editor, visualization, and analysis platform. *J. Cheminform.* **2012**, *4*, 17.

11. Riplinger, C. and F. Neese, An efficient and near linear scaling pair natural orbital based local coupled cluster method. *J. Chem. Phys.* **2013**, *138*, 034106.

12. Riplinger, C., et al., Natural triple excitations in local coupled cluster calculations with pair natural orbitals. *J. Chem. Phys*. **2013**, *139*, 134101.

13. Riplinger, C., et al., Sparse maps--A systematic infrastructure for reduced-scaling electronic structure methods. II. Linear scaling domain based pair natural orbital coupled cluster theory. *J. Chem. Phys*. **2016**, *144*, 024109.

14. Saitow, M., et al., A new near-linear scaling, efficient and accurate, open-shell domain-based local pair natural orbital coupled cluster singles and doubles theory. *J. Chem. Phys*. **2017**, *146*, 164105.

15. Guo, Y., et al., Communication: An improved linear scaling perturbative triples correction for the domain based local pair-natural orbital based singles and doubles coupled cluster method [DLPNO-CCSD(T)]. *J. Chem. Phys*. **2018**, *148*, 011101.

16. Hellweg, A., et al., Optimized accurate auxiliary basis sets for RI-MP2 and RI-CC2 calculations for the atoms Rb to Rn. *Theoretical Chemistry Accounts*, **2007**, *117*, 587-597.

17. Marenich, A.V., C.J. Cramer, and D.G. Truhlar, Universal Solvation Model Based on Solute Electron Density and on a Continuum Model of the Solvent Defined by the Bulk Dielectric Constant and Atomic Surface Tensions. *J. Phys. Chem. B*, **2009**, *113*, 6378-6396.

18. Yang, Z.; Tang, P.; Gauuan, J. F.; Molino, B. F. Asymmetric Total Synthesis of (+)-Crassalactone D. *J. Org. Chem.* **2009**, *74*, 9546.

19. Popsavin, V.; Kovacević, I.; Benedeković, G.; Popsavin, M.; Kojić, V.; Bogdanović, G. Divergent Synthesis of Cytotoxic Styryl Lactones Related to Goniobutenolides A and B, and to Crassalactone D. *Org. Lett.* **2012**, *14*, 5956.

20. Acharyya, R. K.; Nanda, S. Asymmetric Total Synthesis of Naturally Occurring Spirocyclic Tetranorsesquiterpenoid Lanceolactone A. *Org. Biomol. Chem.* **2018**, *16*, 5027.

21. López, F.; Harutyunyan, S. R.; Meetsma, A.; Minnaard, A. J.; Feringa, B. L. Copper-Catalyzed Enantioselective Conjugate Addition of Grignard Reagents to α,β-Unsaturated Esters. *Angew. Chem. Int. Ed.* **2005**, *44*, 2752.

22. Alonso, B.; Ocejo, M.; Carrillo, L.; Vicario, J. L.; Reyes, E.; Uria, U. Using Heteroaryl-Lithium Reagents as Hydroxycarbonyl Anion Equivalents in Conjugate Addition Reactions with (S,S)-(+)-Pseudoephedrine as Chiral Auxiliary; Enantioselective Synthesis of 3-Substituted Pyrrolidines. *J. Org. Chem.* **2013**, *78*, 614.

23. Kawasaki, M.; Kato, D.; Okada, T.; Morita, Y.; Tanaka, Y.; Toyooka, N. Synthesis and Olfactory Evaluation of Optically Active β-Alkyl Substituted γ-Lactones and Whiskey Lactone Analogues. *Tetrahedron.* **2020**, *76*, 130984.

24. Manojveer, S.; Salahi, S.; Wendt, O. F.; Johnson, M. T. Ru-Catalyzed Cross-Dehydrogenative Coupling between Primary Alcohols to Guerbet Alcohol Derivatives: With Relevance for Fragrance Synthesis. *J. Org. Chem.* **2018**, *83*, 10864.
